# Supplementary material for: ATAC-seq footprinting unravels kinetics of transcription factor binding during zygotic genome activation
Source: Nat Commun. 2020 Aug 26;11:4267. doi: 10.1038/s41467-020-18035-1 (PMC7449963; doi:10.1038/s41467-020-18035-1)
Supplement: Supplementary file 3 — Description of Additional Supplementary Files [file 41467_2020_18035_MOESM3_ESM.pdf]

Description of Additional Supplementary Files

File Name: Supplementary Data 1

Description: Visualization of different methods for Tn5 bias correction across all 217 TFs with matched ChIP-seq. Each page contains footprints for a specific TF across all binding sites (in peaks), bound sites (overlapping ChIP-seq) and unbound sites (not overlapping ChIP-seq) for uncorrected/expected/corrected signals from different bias correction methods.
