# Supplementary material for: ATAC-seq footprinting unravels kinetics of transcription factor binding during zygotic genome activation
Source: Nat Commun. 2020 Aug 26;11:4267. doi: 10.1038/s41467-020-18035-1 (PMC7449963; doi:10.1038/s41467-020-18035-1)
Supplement: Supplementary file 1 — Supplementary Information [file 41467_2020_18035_MOESM1_ESM.pdf]

# Supplementary information

## ATAC-seq footprinting unravels kinetics of transcription factor binding during zygotic genome activation

Bentsen et al. 2020

---

## Contents


Supplementary Tables ... 2

Supplementary Table 1: Comparison of features for ATAC-seq footprinting tools ... 2

Supplementary Figures ... 3

Supplementary Figure 1: Overview of the TOBIAS framework tools ... 3

Supplementary Figure 2: Tn5-bias correction is crucial for visualization of footprints from ATAC-seq ... 4

Supplementary Figure 3: Comparison of TOBIAS to existing footprinting methods ... 5

Supplementary Figure 4: Transcription factor activity and expression during mouse and human development ... 6

Supplementary Figure 5: Dux binding is visible as footprints and correlate with ChIP-signal ... 7

Supplementary Figure 6: Predicted Dux binding site correlates with increase in expression ... 8

Supplementary Methods ... 9

1. The TOBIAS framework tools ... 9

Bias correction (TOBIAS ATACorrect module) ... 9

Footprinting (TOBIAS ScoreBigwig module) ... 10

Estimation of transcription factor states and pairwise comparison between conditions (TOBIAS BINDetect module) ... 11

Aggregate plots and calculation of footprint depth (TOBIAS PlotAggregate module) ... 12

Significance of footprint depths calculated by TOBIAS PlotAggregate ... 13

Transcription factor binding network (TOBIAS CreateNetwork module) ... 13

2. TOBIAS pipeline ... 13

3. Validation of TOBIAS and comparison to existing methods ... 13

Datasets used for validation ... 14

Comparison of bias correction tools ... 14

Comparison of footprinting tools ... 14

Supplementary References ... 17

Supplementary Notes ... 18

Supplementary Note 1: BINDetect output for mouse developmental stages ... 18

Supplementary Note 2: BINDetect output for human developmental stages ... 37

# Supplementary Tables

Supplementary Table 1: Comparison of features for ATAC-seq footprinting tools

| | ***Footprinting tools for ATAC-seq*** | | | | |
|---|---|---|---|---|---|
| | **TOBIAS** | **HINT-ATAC** | **MsCentipede** | **PIQ** | **Wellington** |
| **Overview:** | | | | | |
| Year of publication | - | 2019 | 2015 | 2014 | 2013 |
| Tool availability | Github | Github | Github | Bitbucket | Github |
| Programming language | Python | Python | Python | R | Python |
| Type of footprinting<br>(D: De novo, M: Motif-centric) | D | D | M | M | D |
| **Features:** | | | | | |
| Footprinting | ✓ | ✓ | ✓ | ✓ | ✓ |
| Tn5 bias-correction | ✓ | ✓ | × | × | × |
| Size-adjustable footprinting algorithm | ✓ | × | × | × | × |
| Differential footprinting | ✓ | ✓ | × | × | ✓ |
| Time series footprinting<br>(comparison of 2+ conditions) | ✓ | × | × | × | × |
| Calculation of TFBS (from motifs) | ✓ | ✓ | × | ✓ | × |
| Consensus motifs for clustered motifs | ✓ | × | × | × | × |
| TFBS clustering | ✓ | × | × | × | × |
| Output of genomic tracks | ✓ | ✓ | × | × | ✓ |
| Adjustable plotting of aggregate footprints | ✓ | × | × | × | × |
| Visualization of locus footprints | ✓ | × | × | × | × |
| Inference of TF-binding networks | ✓ | × | × | × | × |
| Predict bound/unbound state per TFBS | ✓ | × | ✓ | ✓ | × |
| **Usability:** | | | | | |
| Uses standard file formats | ✓ | ✓ | × | × | ✓ |
| Parallel computing | ✓ | × | × | × | ✓ |
| Complete workflow available<br>- Snakemake<br>- Nextflow | ✓<br>✓<br>✓ | × | × | × | × |
| Cloud computing supported | ✓ | × | × | × | × |
| Parallel computing | ✓ | × | × | × | ✓ |
| Time to execute* (min) | 7.2 | 46 | 2808 | 329 | 8 |
| Package manager/installer | Pypi<br>Bioconda | Pypi<br>Bioconda | - | - | Pypi<br>Bioconda |

* CPU time (using 30 cores if applicable) to perform bias correction (if applicable) and footprinting for GM12878 chromosome 1 using 54 transcription factors matched to ENCODE ChIP-seq.

# Supplementary Figures

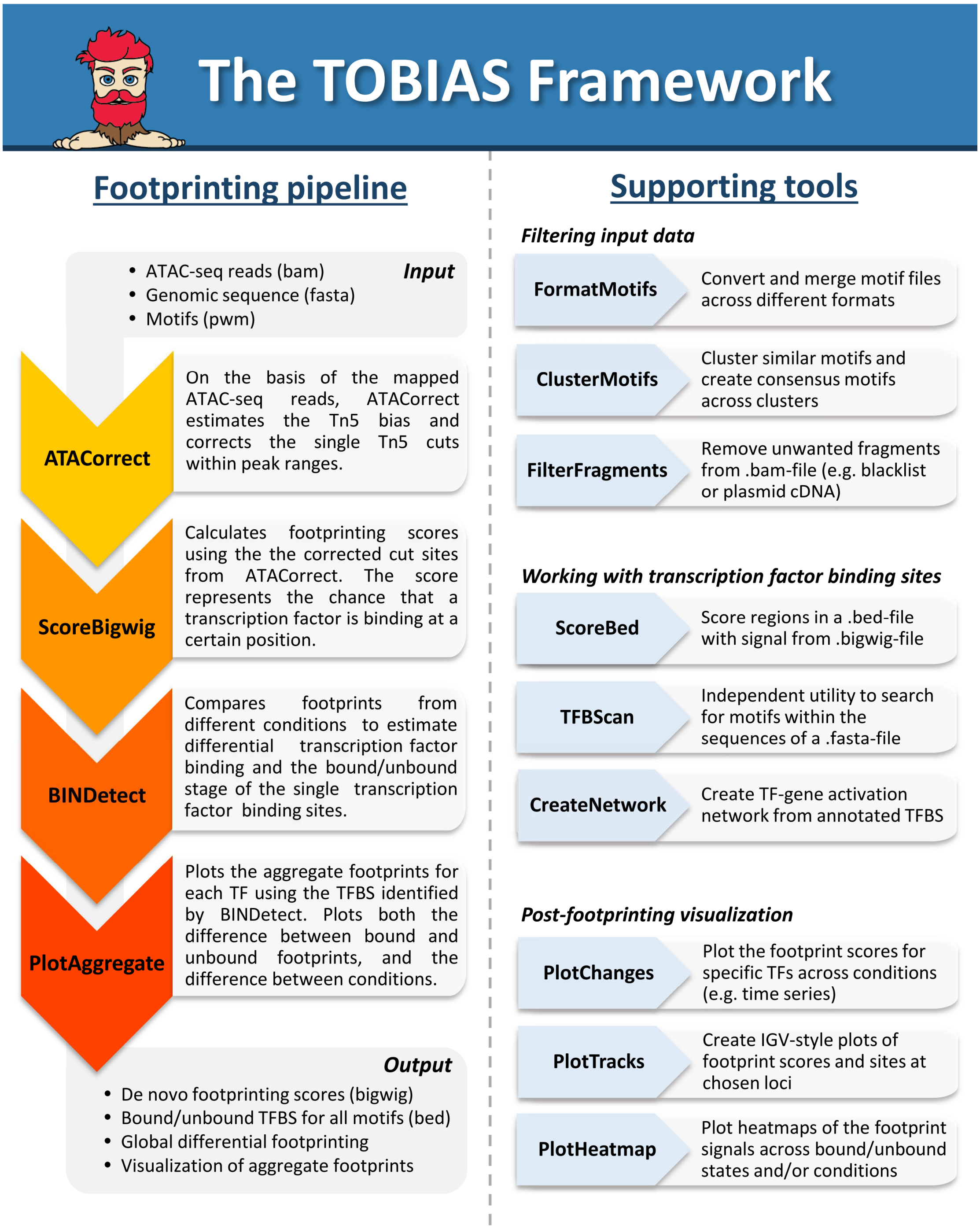


**Supplementary Figure 1: Overview of the TOBIAS framework tools**

The TOBIAS tools are intended for use in a standardized pipeline as shown on the left. ATACorrect and ScoreBigwig corrects Tn5 cuts and calculates footprint scores respectively. Next, BINDetect introduces information from different transcription factor binding motifs to predict binding sites both within and across conditions. PlotAggregate is used to visualize aggregate footprints. Furthermore, a large variety of supporting tools can be used at different stages of the pipeline (right), such as pre-filtering of .bam-files using FilterFragments or plotting of locus-specific footprints using PlotTracks.

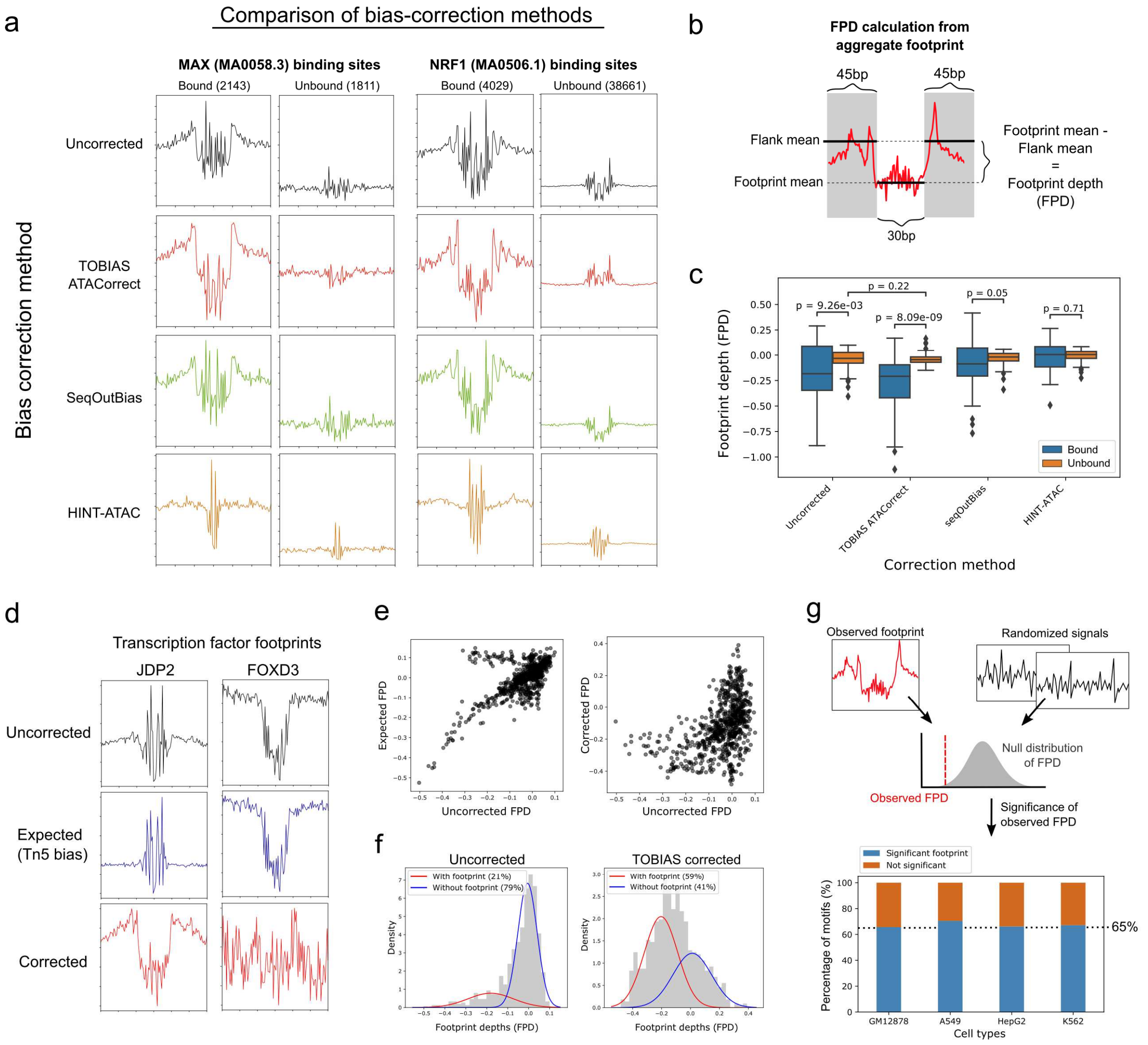


**Supplementary Figure 2: Tn5-bias correction is crucial for visualization of footprints from ATAC-seq**

*(a)* Comparison of aggregate footprints for transcription factors MAX and NRF1 across different bias-correction methods in cell type GM12878. Bound and unbound sites are defined by overlap of motif occurrences with ChIP-seq peaks. The aggregate signals are shown in a 120bp window using uncorrected signals (pileup of Tn5 insertions), TOBIAS ATACorrect, SeqOutBias and HINT-ATAC correction methods. Aggregate signals for all included TFs and cell types are found in the Supplementary Data 1. *(b)* The footprint depth (FPD) of an aggregate footprint is the difference between the mean of the flank signal and the mean of the footprint signal. Negative FPDs represent a stronger footprint. *(c)* Quantification of footprint depths between bound/unbound subsets (as explained in (a)) for different bias correction methods. N=54 transcription factors per boxplot where the bounds of the box are 25th and 75th percentiles (Q1 and Q3), the center is the median, and the whiskers are defined as Q1 - 1.5*(Q3-Q1) and Q3 + 1.5*(Q3-Q1). Significance is calculated by a two-sided Mann-Whitney U-test without adjustments. “Uncorrected” refers to the footprint depths estimated from uncorrected Tn5 signals. *(d)* The aggregate footprints for transcription factors JDP2 and FOXD3 across the uncorrected, expected and corrected Tn5 signals in GM12878. *(e)* Correlation between depth of footprints for uncorrected vs. expected footprints (left) and corrected vs. expected footprints (right). The plot consists of FPD values for N=746 motifs in cell type GM18278. *(f)* Mixture model of N=746 footprint depths in cell type GM12878. For uncorrected signals (left), the mixture model shows that 21% of all motifs generate a footprint. For corrected signals (right), 59% of all motifs to generate a footprint. *(g)* Significance of observed footprint depths. A null distribution of random aggregate footprints is created by shuffling the observed aggregate signal values. The observed footprint depth is compared to the null distribution for each TF, and the significance of the FPD being on the lower tail of the null distribution is calculated (Z-score; one-sided p-value < 0.01). Source data is provided in the Source Data file.

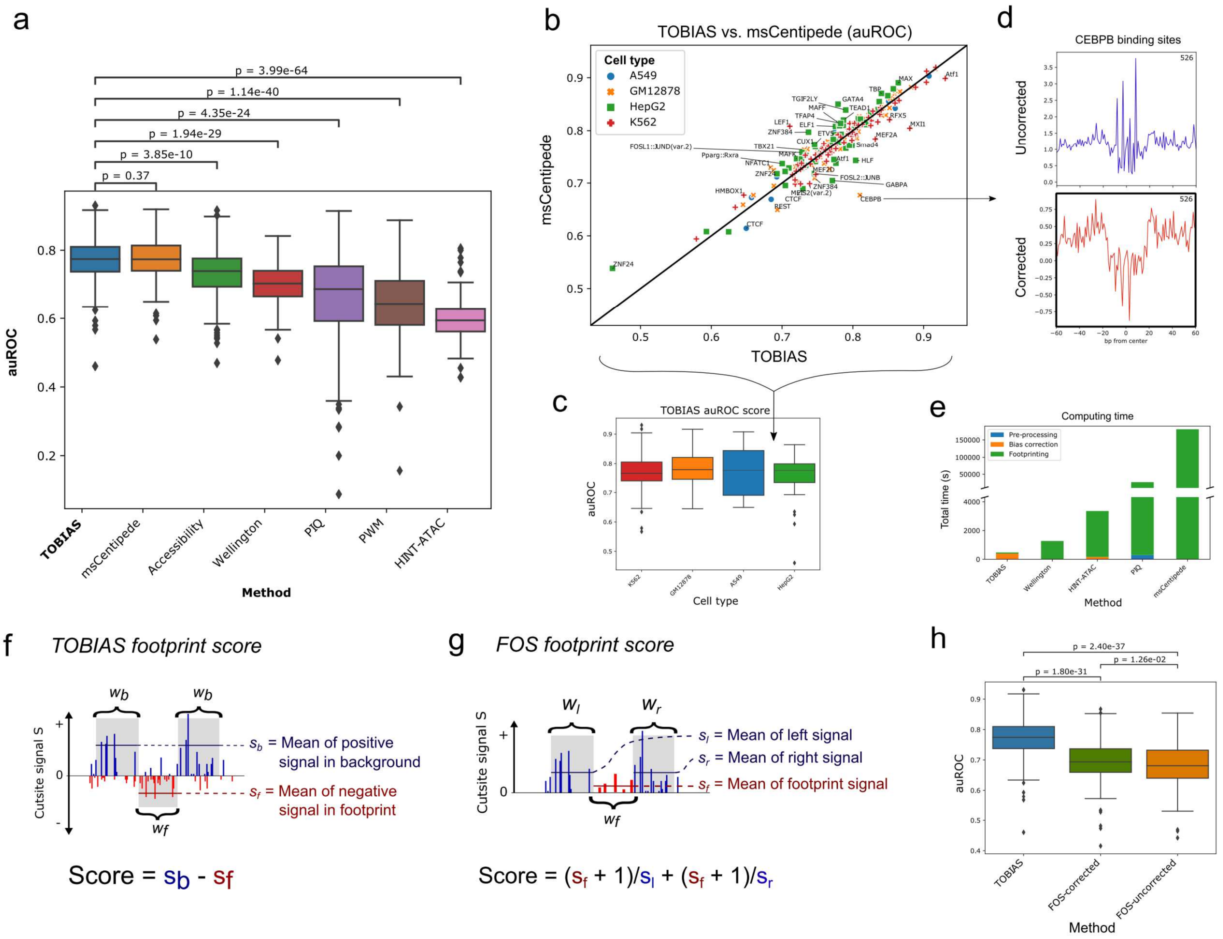


## Supplementary Figure 3: Comparison of TOBIAS to existing footprinting methods

*(a)* Comparison of predictive power across different footprinting methods. The auROC (area under the Receiver Operating Curve) is used as a measure of predictive power for each method and is generated by overlap with ChIP-seq peaks (limited to chromosome 1). N=217 TF experiments across four cell types. Significance is defined by a two-sided Mann-Whitney U test without adjustment. *(b)* Scatterplot comparing the auROC of TOBIAS and msCentipede. Each point represents one TF based on N=217 paired ChIP-seq/ATAC-seq datasets, which are colored and marked dependent on the respective cell type the ChIP-seq was performed on. The diagonal line represents equal auROC between TOBIAS and msCentipede. *(c)* The auROC of TOBIAS predictions across cell types K562 (n=87), GM12878 (n=54), HepG2 (n=64) and A549 (n=12). *(d)* The aggregate footprints for true CEBPB binding sites (bound sites verified by ChIP-seq) in cell line GM12878. The number in the upper-right corner (526) represents the number of CEBPB binding sites included in the aggregate. *(e)* Comparison of computing times for footprinting tools. The CPU run time for each tool is measured across the three tasks of "pre-processing" (only for PIQ), "bias-correction" (only for TOBIAS and HINT-ATAC) and footprinting (all tools). *(f)* Calculation of the TOBIAS footprint score. The term $w_b$ is the width of the flanking background left/right, and $w_f$ is the width of the footprint. The score is calculated as the difference of the background mean (positive values) and the footprint mean (negative values). *(g)* Calculation of the FOS footprint score. The FOS (Footprint Occupancy Score) is calculated on the basis of the mean signals of the left flank (of width $w_l$), the right flank (of width $w_r$) and the footprint (of width $w_f$). *(h)* Comparison of score predictions (N=217 TFs) (as explained in (a)) for TOBIAS, FOS footprint score on corrected data, and FOS footprint score on uncorrected data. Significance is defined by a two-sided Mann-Whitney U test without adjustment. All boxplots are defined with the box being the 25th and 75th percentiles (Q1 and Q3), the center being the median, and the whiskers defined as Q1 - 1.5*(Q3-Q1) and Q3 + 1.5*(Q3-Q1).

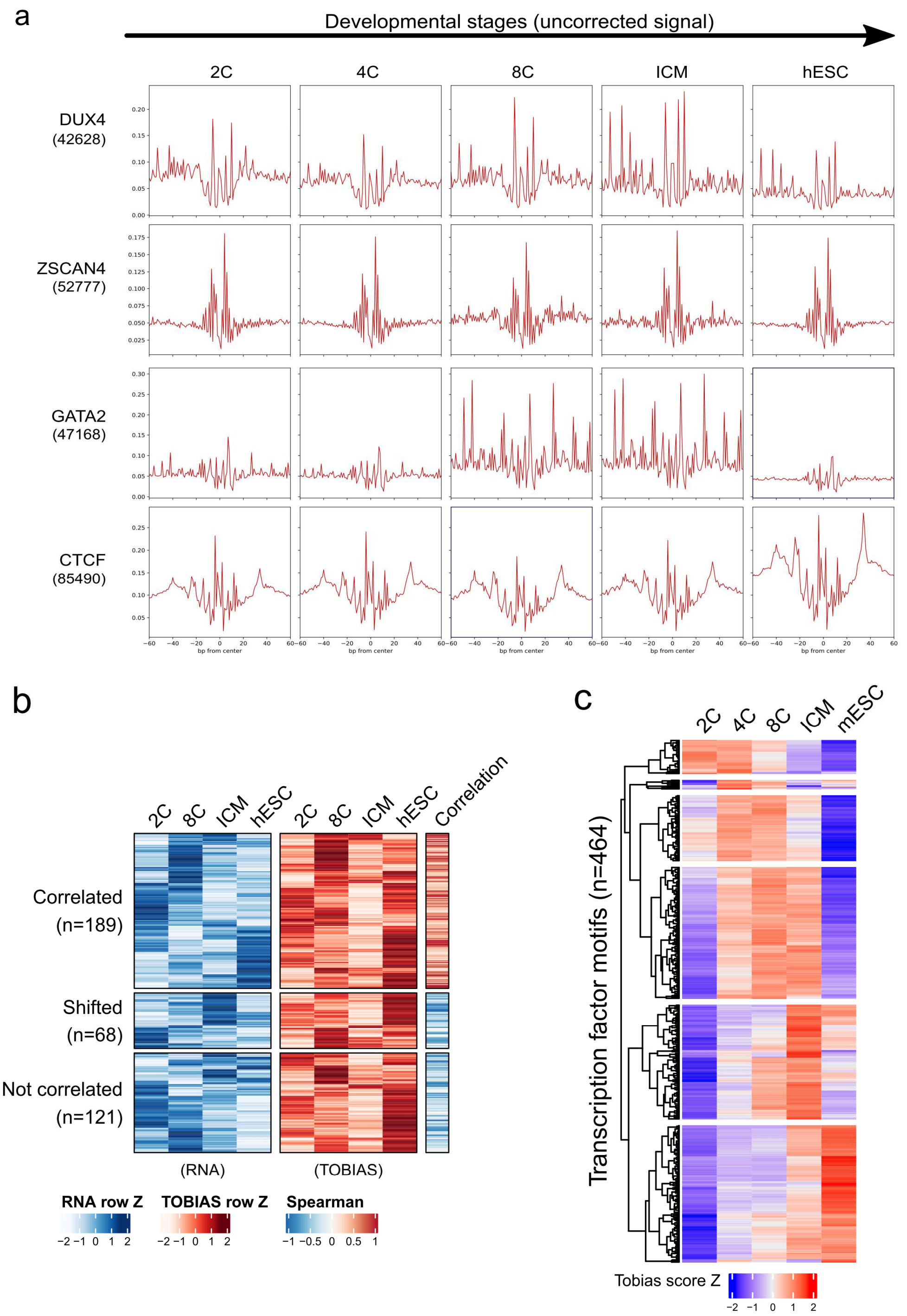


**Supplementary Figure 4: Transcription factor activity and expression during mouse and human development**

*(a)* Depiction of uncorrected footprint aggregates across time points for transcription factors DUX4, ZSCAN4, GATA2 and CTCF. Aggregated Tn5 signals are shown across time points 2C, 4C, 8C, ICM and hESC. The numbers below each TF name represent the number of binding sites included in the plots. *(b)* Correlation of footprints and RNA-seq. The left heatmap (blue) depicts expression of transcription factor clusters in the respective human developmental stages. The left heatmap (red) depicts the corresponding TOBIAS scores. Spearman column represents the spearman correlation between TOBIAS/RNA rows. The TF clusters are grouped into “Correlated” (Spearman≥0.2), “shifted” (RNA max value appears before TOBIAS max value) and “Not correlated” (Spearman<0.2 with no apparent shift in RNA). (c) Dynamic transcription factor binding during mouse embryonic development. The heatmap depicts the TOBIAS-predicted footprint scores for 464 motifs during the time points 2C, 4C, 8C, ICM and mESC. The rows are clustered into 6 clusters using hierarchical clustering. Source data for (b) and (c) is provided in the Source Data file.

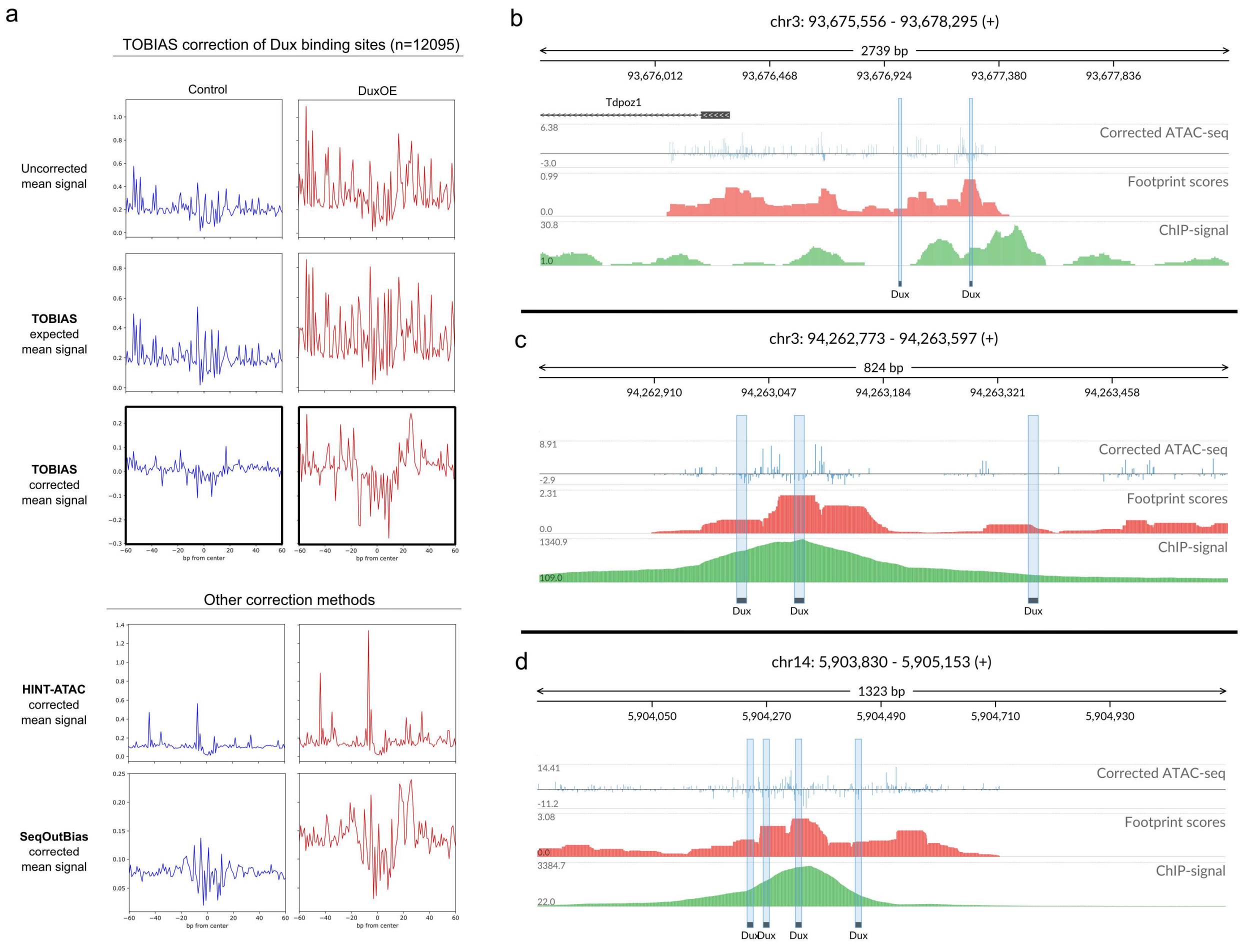


**Supplementary Figure 5: Dux binding is visible as footprints and correlate with ChIP-signal**

*(a)* Correction of the Dux footprint using different bias correction methods. The aggregate footprints for N=12095 Dux binding sites (within ATAC-seq peaks) are shown between Control and *Dux*OE conditions. The top three panels depict the uncorrected, expected and corrected signals as calculated by TOBIAS. The bottom panels depict the same sites corrected by either HINT-ATAC or SeqOutBias methods. *(b)* A view of the footprinting scores in the promoter of *Tdpoz1*. Genomic tracks show corrected ATAC-seq cutsites at 1bp resolution (blue), footprint scores as calculated by TOBIAS (red), and pileup of reads from Dux ChIP-seq from Hendrickson et al. 2017 (green). Potential Dux binding sites are highlighted in blue. *(c-d)* Footprinting correlates with ChIP-signal at multiple genomic loci. Genomic tracks are the same as described for (b).

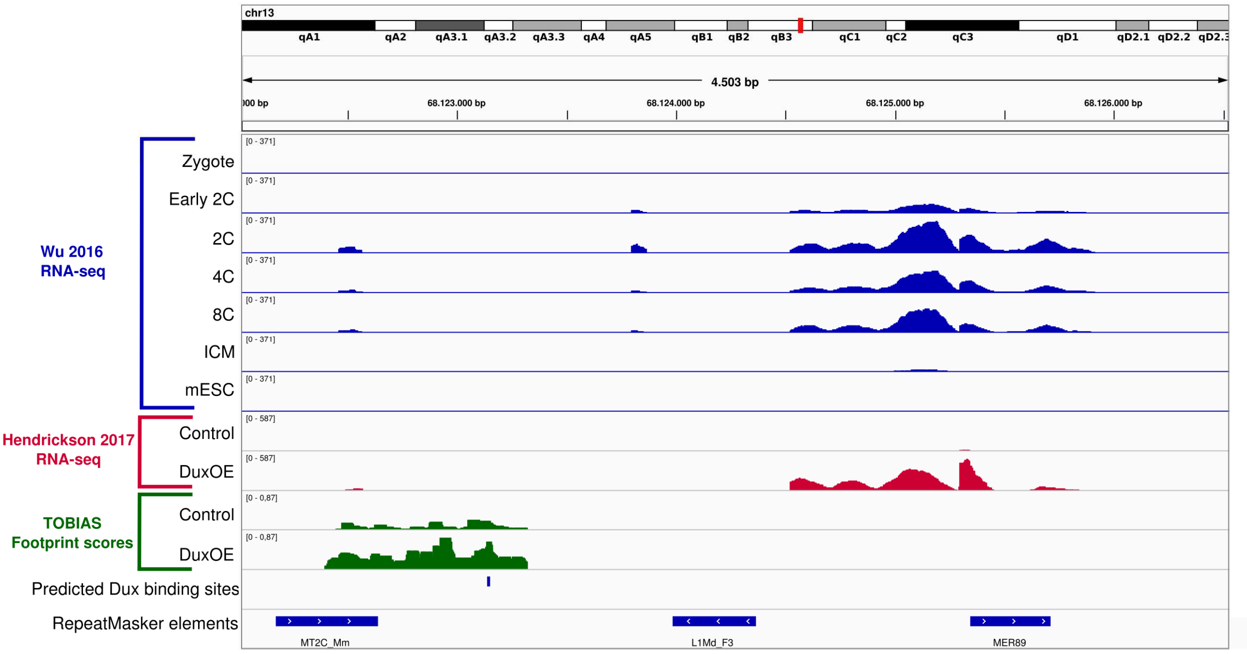


**Supplementary Figure 6: Predicted Dux binding site correlates with increase in expression**

The figure shows genomic tracks of RNA-seq from embryonic cell stages (blue) and *Dux* overexpression (red), TOBIAS footprint scores predicted from *Dux* overexpression ATAC-seq (green), predicted Dux binding site as well as known repeats as annotated by RepeatMasker[1].

# Supplementary Methods

## 1. The TOBIAS framework tools

In developing TOBIAS, we found that there were six main areas of DGF that had not been comprehensively addressed in the context of ATAC-seq footprinting analysis:

1. All-in-one framework including bias correction, footprinting, quantification of protein binding and visualization
2. Investigation of TF binding on a global level (which TFs are more bound globally) as well as the locus-specific level (which TF binds to which genomic locations including statistics on differential binding)
3. Consideration of the redundancy and similarity of known TF binding motifs in the context of footprinting
4. A scoring model for TF-DNA binding taking into account the potential lack of a canonical footprint effect
5. Comparison and quantification of TF binding activity within complex experimental settings (multiple conditions or time series)
6. Automated workflows for recurring analysis tasks

Modules enabling these individual analysis steps are included in the TOBIAS package, which is publicly available at Github (https://github.com/loosolab/TOBIAS) as well as on PyPI and Bioconda. Besides the examples given in the repository README, we provide a Wiki (https://github.com/loosolab/TOBIAS/wiki) which introduces some of the individual software modules. We used the pre-defined workflows in Snakemake and NextFlow to run the full analysis (see section 2). The single modules, and how they were applied in this investigation, are explained in more detail below.

### Bias correction (TOBIAS ATACorrect module)

Each Tn5-cut site is defined as the 5' end of a read shifted by +5 at the plus strand and -4 at the minus strand to center the transposase event. Using the mapped reads from closed chromatin, ATACorrect builds a dinucleotide weight matrix[2] representing the preference of Tn5 insertion. In contrast to the classical position weight matrix (PWM), the dinucleotide weight matrix (DWM) captures the inter-base relationships which arise due to the palindromic nature of the bias. A background model is similarly built by shifting all reads +100bp as described by Koohy et al.[3].

Reads within open chromatin peaks are then corrected by estimating the expected number of cuts per base pair and subtracting this from the observed cut sites as follows (modified from [4]):

$$c_i = x_i - e_i \quad (1)$$

for

$$e_i = \hat{x}_i * \hat{b}_i \quad (2) \quad , \quad \hat{x}_i = \sum_{j=i-50}^{i+50} x_j \quad (3) \quad , \quad \hat{b}_i = \frac{b_j}{\sum_{j=i-50}^{i+50} b_j} \quad (4)$$

Where:

- $c_i$ is the corrected number of cuts
- $x_i$ is the observed number of cuts
- $e_i$ is the expected number of cuts
- $b_i$ is the calculated bias level
- *i* is the respective position in the genome.

To limit the influence of low-bias positions in the calculation of $\hat{b}_i$ , a lower limit is set for $b_i$ by calculating the fit of cutsites vs. bias to a rectified linear unit function (ReLu) in moving 100bp-windows and setting every $b_i$ below the linear fit to 0. This calculation is performed for all base pairs within open chromatin, setting all other positions in closed chromatin to 0. Lastly, each $c_i$ is rescaled to fit the original sum of cuts $\hat{x}_i$ for each window.

### Footprinting (TOBIAS ScoreBigwig module)

TOBIAS estimates footprint scores $F_i$ across open chromatin regions by calculating:

$$F_{[i,i+w_f]} = \bar{c}_b - \bar{c}_f \quad (5)$$

For the following calculations:

- $F$ is the continuous footprint score
- $i$ is a position in the genome
- $w_f$ is the width of the footprint (in base pairs)
- $w_b$ is the width of each background regions upstream/downstream of the footprint (in base pairs)
- $\bar{c}_b$ is the mean of corrected footprint cuts smaller than 0
- $\bar{c}_f$ is the mean of corrected background cuts larger than 0

The footprint score *F* in the positions ranging from *i* to $i + w_f$ (the whole width of the footprint) is given as the difference of $\bar{c}_b$ and $\bar{c}_f$ . The range of the footprint is defined as $[i, i + w_f]$ and the ranges of the background are defined as $[i - w_b, i\ [$ and $]i + w_f, i + w_f + w_b]$. In detail, the calculation of $\bar{c}_b$ and $\bar{c}_f$ are defined as:

$$\bar{c}_b = \frac{\sum_{j=1}^{w_b} c_{[i-j]} + \sum_{j=1}^{w_b} c_{[i+w_b+j]}}{2 * w_b} \text{ for } c_i > 0 \quad (6)$$

$$\bar{c}_f = \frac{\sum_{i=1}^{w_f} c_i}{w_f} \text{ for } c_i < 0 \quad (7)$$

The default ranges for the widths are $w_b = [10,30]$ and $w_f = [20,50]$. For each *i*, multiple footprint scores *F* are calculated for different combinations of $w_b$ and $w_f$. For each *i*, the combination of $w_b$ and $w_f$ with the highest score is taken as $F_i$.

This footprint score is a combination of depletion and accessibility as a measure of protein binding. The term $\bar{c}_f$ will be negative and will therefore raise the score if there is a high depletion of cuts in the footprint. If there is no depletion, the score will simplify to the mean of cuts in the background regions, representing accessibility. It is therefore not essential to visibly see a canonical footprint shape for the footprint score to

be high. The footprint score can be interpreted as higher scores giving more evidence that a protein was bound at a given position.

### Estimation of transcription factor states and pairwise comparison between conditions (TOBIAS BINDetect module)

To match the calculated footprint scores to potential binding sites, TOBIAS BINDetect integrates genomic sequence, footprint scores from several conditions, as well as motifs, to identify up- and down regulated TFs based on footprint scores.

In the first step of the algorithm, the MOODS library (https://github.com/jhkorhonen/MOODS)[5] is used to detect TF binding sites (within peaks) with a p-value threshold of 1e-4. Background base pair probabilities are estimated from the input peak set. Subsequently, each binding site is matched to footprint scores for each condition. Simultaneously, a background distribution of values is built by randomly subsetting peak regions at ~200bp intervals, and the scores from each condition are normalized to each other using quantile normalization. These values are used to calculate a distribution of background log2FCs for each pairwise comparison of conditions.

Overlaps between the TFBS identified in the first step are quantified by creating a distance matrix of TFs. The distance *d* between a TF pair (TF1;TF2) is calculated as:

$$d_{TF1;TF2} = 1 - max(\frac{o_{TF1;TF2}}{n_{TF1}}, \frac{o_{TF1;TF2}}{n_{TF2}}) \quad (8)$$

Where:

- $n_{TF1}$ and $n_{TF2}$ are the number of base pairs covered by all *TF1* and TF2 sites respectively
- $o_{TF1;TF2}$ is the amount of base pairs overlapping between *TF1* and *TF2* sites.

The max-statement ensures that the overlap is calculated with regards to the shortest TF motif. As an example, imagine two TFs have overlapping TFBS defined as:

- TFBS ranges for TF1: [10,20] & [50,60]
- TFBS ranges for TF2: [15,30] & [70,85]

In this example: $o_{TF1;TF2} = 5$, $n_{TF1} = 20$ and $n_{TF1} = 30$. The maximum fraction of overlap is therefore 5/20, making $d_{TF1;TF2} = 1 - 0.25 = 0.75$.

In the second step of the algorithm, every TF binding site found (for each motif given as input) is split into bound and unbound sites based on a score threshold per condition. The threshold is set at the level of significance of a normal-distribution fit to the background distribution of scores (user-defined p-value). As well as the per-condition split, each site is assigned a log2FC (fold change) per comparison, which represents whether the binding site has larger/smaller footprint scores in comparison. The global distribution of log2FC's per TF is compared to the background distributions to calculate a *differential binding score*, which is calculated as:

$$\frac{(\bar{x}_0 - \bar{x}_b)}{((std_o + std_b) / 2)} \quad (9)$$

Where:

- $\bar{x}_0$ and $\bar{x}_0$ are the means of the observed (*o*) and background (b) log2FC distributions respectively.
- $std_o$ and $std_b$ are standard deviations of the observed (*o*) and background (b) log2FC distributions respectively.

A p-value is calculated by subsampling 100 log2FCs from the background and calculating the significance of the observed change. By comparing the observed log2FC distribution to the background log2FC, the effects of any global differences due to sequencing depth, noise etc. are controlled.

The differential binding scores and p-values are visualized as a volcano plot per condition-comparison. All TFs with -log10(p-value) above the 95% quantile or differential binding scores smaller/larger than the 5% and 95% quantiles (top 5% in each direction) are colored and shown with labels. Below the plot, hierarchical clustering of the TFBS-distance matrix is shown and all TFs with distances less than 0.5 (overlap of over 50% of base pairs) are colored as separate clusters.

The result of BINDetect is a folder-structure containing an overview of all potential binding sites (as .bed as well as excel-files), the predicted split into bound and unbound sites, and a global overview of differentially bound TFs per condition-comparison.

### Aggregate plots and calculation of footprint depth (TOBIAS PlotAggregate module)

Footprints are visualized using the subtool "TOBIAS PlotAggregate". Aggregate footprints are created by aligning genomic signals centered on all binding sites (taking into account strandedness), to create a matrix of (*n* sites) x (*m* bp). The aggregate signal is calculated as the mean of each column (each bp). The default of +/- 60bp from the motif center was used throughout this manuscript.

The aggregate footprinting depth (FPD), which is applied in Supplementary Fig. 2b-g, was calculated for each TF as:

$$FPD = \bar{s}_f - \bar{s}_b \qquad (10)$$

Where:

- $\bar{s}_b$ is the mean of the aggregate signal in the background region
- $\bar{s}_f$ is the mean of the aggregate signal in the footprint region

The smaller the FPD, the stronger the depletion of the footprint. For this manuscript, we visualized all footprints +/- 60bp from the motif center, while the width of the footprint region ($w_f$) was set to 30bp. Accordingly, each flanking region ($w_b$) is given with 45bp. Formally written as:

$$FPD = \frac{\sum_{i=1}^{w_f} s_{[45+i]}}{w_f} - \frac{\sum_{i=1}^{w_b} s_i + \sum_{j=i}^{w_b} s_{[45+30+i]}}{2 * w_b} \qquad (11)$$

The aggregate signal *s* can be calculated from any source of signals, but will in most cases be the aggregate of the corrected signal *c* as explained above.

Similarly to the investigations in previous literature[6], we applied a mixture model (Python's sklearn.mixture.GaussianMixture) to estimate the fractions of TFs with/without measurable footprints (Supp. Figure 2e).

**Significance of footprint depths calculated by TOBIAS PlotAggregate**

To calculate the significance of the footprint depth (as presented in Supplementary Fig. 2g), the aggregated signal for each TF was collected and the FPD was calculated for the 30bp footprint as explained above. For each aggregated signal (an array of length 120), 1000 randomized signals were created and the FPDs for these were correspondingly calculated. The observed FPD was compared to this null-distribution of FPDs using a Z-score (significance estimated using Python scipy.stats.norm.sf). All p-values (for 746 motifs from the JASPAR 2020 motif database) were FDR-corrected using the Benjamini-Hochberg procedure. This procedure was repeated for each of the four cell types used in the validation (see Validation-section below).

**Transcription factor binding network (TOBIAS CreateNetwork module)**

The TF-TF network for Dux was built by subsetting all binding sites on the following characteristics: Bound in the promoter of a target gene, labeled "Unbound" in Control, labeled "Bound" in *Dux*OE, and log2FC footprint score increasing for *Dux*OE vs. Control. All targets were further reduced to only include genes encoding TFs with available motifs. Motifs were matched to genes as explained in the methods section "Processing of transcription factor motifs". The network was then created using "TOBIAS CreateNetwork". This method first finds all edges of the graph (the connection between bound TFBS and target gene). Next, the adjacency matrix for each node is built. Subsequently, a recursive function is run to traverse the adjacency graph to find all paths. The result is a network of source and target nodes with directed edges, which in words can be described as: *Source TF* binds in the promoter of *Target TF*.

## 2. TOBIAS pipeline

To couple individual TOBIAS tools, we provide scalable analysis workflows implemented in Snakemake[7] and NextFlow[8], including a cloud computing compatible version making use of the de.NBI cloud (https://cloud.denbi.de). These pipelines enable complete footprinting analysis and comparison of TF binding even for complex experimental designs (e.g. time series).

The output generated by the TOBIAS pipeline (Snakemake or NextFlow) is organized in a hierarchical folder structure, which increases clarity of all steps of the analysis. The folder structure specifically organizes input data, pre-processing output like peak calling and annotation, genomic tracks such as bias correction and footprints, as well as the local and global TF predictions. Particularly, the output for every individual TF investigated is arranged into separate folders containing TF specific plots, annotations and binding predictions. This structure makes it simple to use the output for further downstream analysis, as was showcased in this work. An exemplary output of the complete framework can be found at: https://figshare.com/projects/Digital_Genomic_Footprinting_Analysis_of_ATAC-seq_dataset_from_preimplantation_timepoints_via_TOBIAS/69959

## 3. Validation of TOBIAS and comparison to existing methods

Although footprinting tools for DNase-seq exist[9], not all can be applied to paired-end ATAC-seq data. We have therefore focused our comparison on tools which are easily obtainable and installable, do not require ChIP-seq training-data, and are explicitly supporting ATAC-seq. We have additionally added two metrics for "Accessibility" and "Position weight matrix (PWM) score" to compare TOBIAS to other footprinting-free metrics. The validation datasets and usage of existing tools are described in the following sections.

## Datasets used for validation

The TOBIAS framework was benchmarked using ATAC-seq data from four human cell types: GM12878 (GEO: GSE47753), A549 (GEO: GSE114202), K562 (ENA: PRJNA288801) and HEPG2 (ENA: PRJEB30461). ATAC-seq data was trimmed using cutadapt[10] and mapped using Bowtie2[11]. All reads with a quality score <30 as well as non-proper paired reads were removed. All replicates were merged to one joined .bam-file of reads. Peaks were called using MACS2[12] with parameters "--nomodel --shift -100 --extsize 200 --broad --qvalue 0.01 --broad-cutoff 0.01". ChIP-seq peak regions (narrowPeak format) were downloaded from ENCODE and associated to motifs from JASPAR CORE 2018 using "MEME Centrimo"[13]. Only ChIP-seq experiments with motif enrichment > 1.0e-10 (Centrimo E-value) were kept. In case of more than one ChIP-seq experiment for the same target in the same cell type, the one with the highest motif enrichment was chosen. After filtering, there were 12 TFs for A549, 54 TFs for GM12878, 64 TFs for HepG2, and 87 TFs for K562 for a total of 217 ChIP-seq experiments matched to ATAC-seq. Bound binding sites per TF were defined as any TFBS within +/- 50bp from the paired ChIP-seq peak summit. In case of two or more binding sites per peak, the one closest to the summit was set to bound, and other(s) were excluded from the analysis. Unbound binding sites were defined as any TFBS not overlapping any ChIP-seq peak, as well as not overlapping bound sites from any other factors for this cell type. Bound and unbound sites were further filtered to only include TFBS falling within ATAC-seq peaks for the cell type in question.

## Comparison of bias correction tools

TOBIAS was compared to the existing bias correction methods as follows:

- **seqOutBias ([14])**

  The seqOutBias software was downloaded from GitHub (https://github.com/guertinlab/seqOutBias). Following the vignette for ATAC-seq, mappability files were created and ATAC-seq reads were corrected for plus/minus strand reads separately. After correction, we further shifted the positive and negative tracks +5 and -4bp respectively, as this was not performed by the tool itself.

- **HINT-ATAC ([15])**

  The HINT software was downloaded from PyPI as part of the RGT software suite. Bias correction was performed from the ATAC-seq reads using the command "rgt-hint tracks --bc --bigWig <bam>".

Aggregate footprints for each method across all (within peaks), bound and unbound binding sites were visualized using "TOBIAS PlotAggregate".

## Comparison of footprinting tools

Existing footprinting tools were applied as follows:

- **msCentipede ([16])**

  The msCentipede software was downloaded from GitHub (https://github.com/rajanil/msCentipede). For each TF, the binding model was built using the 5000 TFBS with the highest PWM score genomewide. For model learning, the "--mintol" parameter was

set to 1e-3 as a tradeoff between accuracy and speed. The resulting models were then used to infer the posterior binding-probability of TFBS in peaks.

- **Wellington ([17])**

  The pyDNase software was downloaded from PyPI. Footprints in ATAC-seq peaks were estimated using "wellington_footprints.py" with the "-A" option for ATAC-seq mode.

- **PIQ ([18])**

  The PIQ software was downloaded from Bitbucket (https://bitbucket.org/thashim/piq-single/). The script *bam2rdata.r* was used to bring the input .bam-file into the correct data format. Likewise, the script *pwmmatch.exact.r* was used to predict genomewide TFBS. Finally, footprinting scores for each TF were obtained using the script *pertf.r* for each motif/cell type pair. The purity score was taken as the probability for a certain TFBS to be bound.

- **HINT-ATAC ([15])**

  The HINT software was downloaded from PyPI as part of the RGT software suite. Footprints were identified using the command "rgt-hint footprinting --atac-seq --paired-end --organism=hg38 <bam> <peaks>". The output of HINT-ATAC footprinting is a .bed-file of footprint ranges ranked by tag count. All TFBS overlapping a footprint with more than 2/3 of the TFBS bases were assumed to be bound and scored using the tag count of the footprint. The rest of the TFBS (within peaks) were set to score 0 (low chance of protein binding). The auROC was calculated based on the ability of these scores to predict true protein binding. It should be noted that this affects the shape of the ROC curve, as all TFBS without overlaps are assumed to have the same probability of being bound. However, this is a characteristic of the method, and HINT-ATAC was therefore evaluated on the same premise as other tools.

- **Accessibility**

  The "Accessibility" metric is defined as the sum of Tn5 insertions in a 300 basepair window centered at the binding site. This score represents the accessibility of a certain region not taking into account local footprint information.

- **PWM score**

  The score of the motif-sequence match at the specific TFBS. As this is based on sequence alone, the PWM-score is independent of chromatin accessibility.

- **FOS footprint score**

  The Footprint Occupancy Score (FOS) was calculated as described in [19] and pictured in Supplementary Figure 3g. To enable the calculation of FOS for TOBIAS corrected data, all negative signals were set to 0 before calculation.

Due to high computational times for some tools, the validation was limited to binding sites on human chromosome 1. With increasing footprint score thresholds, each TFBS was labeled as “False positive” and “True positive” on the basis of the respective ChIP-seq labels (as explained above). The area under the ROC curve (auROC) was used to evaluate the predictive power of each method.

# Supplementary References


1. Smit, A.F.A. Repeat-Masker Open-3.0. http://www.repeatmasker.org (2004).
2. Siddharthan, R. Dinucleotide weight matrices for predicting transcription factor binding sites: generalizing the position weight matrix. *PLoS One* **5**, e9722 (2010).
3. Koohy, H., Down, T.A. & Hubbard, T.J. Chromatin accessibility data sets show bias due to sequence specificity of the DNase I enzyme. *PLoS One* **8**, e69853 (2013).
4. Gusmao, E.G., Allhoff, M., Zenke, M. & Costa, I.G. Analysis of computational footprinting methods for DNase sequencing experiments. *Nat Methods* **13**, 303-309 (2016).
5. Korhonen, J.H., Palin, K., Taipale, J. & Ukkonen, E. Fast motif matching revisited: high-order PWMs, SNPs and indels. *Bioinformatics* **33**, 514-521 (2016).
6. Baek, S., Goldstein, I. & Hager, G.L. Bivariate Genomic Footprinting Detects Changes in Transcription Factor Activity. *Cell Rep* **19**, 1710-1722 (2017).
7. Koster, J. & Rahmann, S. Snakemake-a scalable bioinformatics workflow engine. *Bioinformatics* **34**, 3600 (2018).
8. Di Tommaso, P. et al. Nextflow enables reproducible computational workflows. *Nat Biotechnol* **35**, 316-319 (2017).
9. Kahara, J. & Lahdesmaki, H. BinDNase: a discriminatory approach for transcription factor binding prediction using DNase I hypersensitivity data. *Bioinformatics* **31**, 2852-2859 (2015).
10. Martin, M. Cutadapt removes adapter sequences from high-throughput sequencing reads. *2011* **17**, 3 (2011).
11. Langmead, B. & Salzberg, S.L. Fast gapped-read alignment with Bowtie 2. *Nature methods* **9**, 357-359 (2012).
12. Feng, J.X., Liu, T., Qin, B., Zhang, Y. & Liu, X.S. Identifying ChIP-seq enrichment using MACS. *Nat Protoc* **7**, 1728-1740 (2012).
13. Bailey, T.L. & Machanick, P. Inferring direct DNA binding from ChIP-seq. *Nucleic Acids Research* **40**, e128-e128 (2012).
14. Martins, A.L., Walavalkar, N.M., Anderson, W.D., Zang, C. & Guertin, M.J. Universal correction of enzymatic sequence bias reveals molecular signatures of protein/DNA interactions. *Nucleic Acids Res* **46**, e9 (2018).
15. Li, Z. et al. Identification of transcription factor binding sites using ATAC-seq. *Genome biology* **20**, 45-45 (2019).
16. Raj, A., Shim, H., Gilad, Y., Pritchard, J.K. & Stephens, M. msCentipede: Modeling Heterogeneity across Genomic Sites and Replicates Improves Accuracy in the Inference of Transcription Factor Binding. *PLoS One* **10**, e0138030 (2015).
17. Piper, J. et al. Wellington: a novel method for the accurate identification of digital genomic footprints from DNase-seq data. *Nucleic Acids Res* **41**, e201 (2013).
18. Sherwood, R.I. et al. Discovery of directional and nondirectional pioneer transcription factors by modeling DNase profile magnitude and shape. *Nature biotechnology* **32**, 171-178 (2014).
19. Neph, S. et al. An expansive human regulatory lexicon encoded in transcription factor footprints. *Nature* **489**, 83-90 (2012).

# Supplementary Notes

## Supplementary Note 1: BINDetect output for mouse developmental stages

The raw output file of the "TOBIAS BINDetect"-module containing differential binding plots across all pairwise-comparisons of mouse developmental stages.

# BINDETECT FIGURES

Page 2) Raw score distributions
Page 3) Normalized score distributions
Page 4) BINDetect plot (early2C / 2C)
Page 5) BINDetect plot (early2C / 4C)
Page 6) BINDetect plot (early2C / 8C)
Page 7) BINDetect plot (early2C / ICM)
Page 8) BINDetect plot (early2C / mESC)
Page 9) BINDetect plot (2C / 4C)
Page 10) BINDetect plot (2C / 8C)
Page 11) BINDetect plot (2C / ICM)
Page 12) BINDetect plot (2C / mESC)
Page 13) BINDetect plot (4C / 8C)
Page 14) BINDetect plot (4C / ICM)
Page 15) BINDetect plot (4C / mESC)
Page 16) BINDetect plot (8C / ICM)
Page 17) BINDetect plot (8C / mESC)
Page 18) BINDetect plot (ICM / mESC)

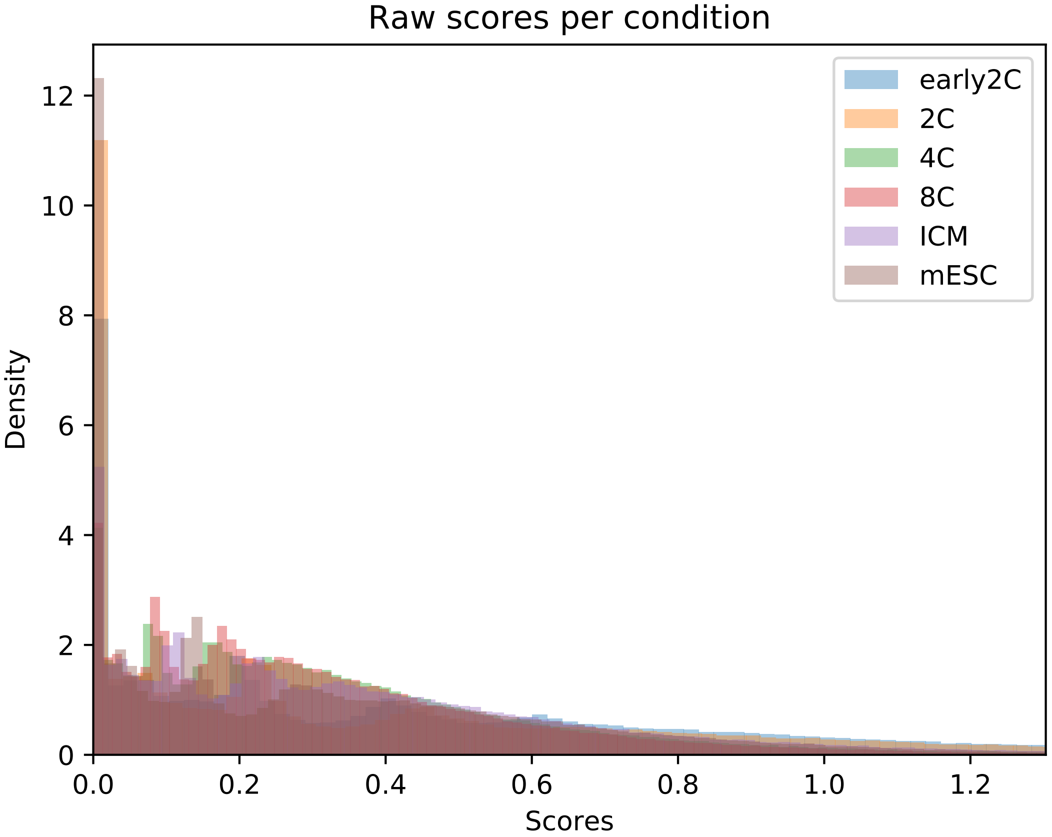
Raw scores per condition
Density
Scores
early2C
2C
4C
8C
ICM
mESC
0
2
4
6
8
10
12
0.0
0.2
0.4
0.6
0.8
1.0
1.2

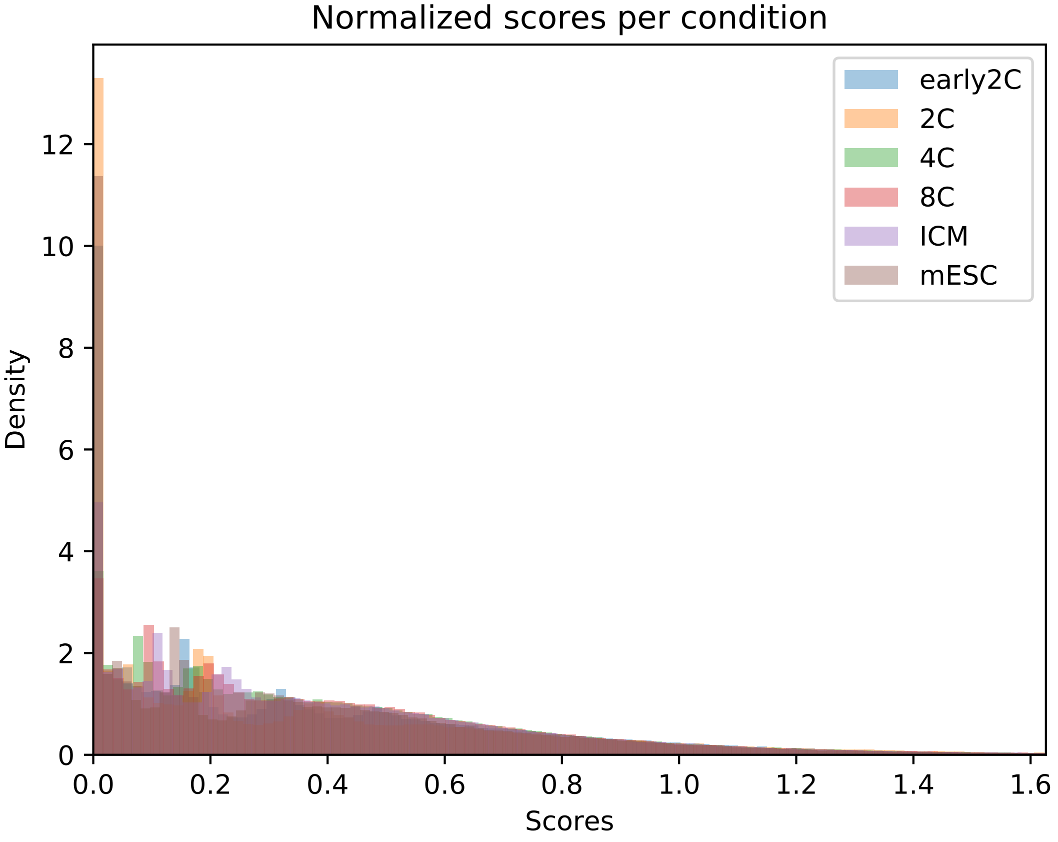

Normalized scores per condition
Density
Scores
early2C
2C
4C
8C
ICM
mESC
0
2
4
6
8
10
12
0.0
0.2
0.4
0.6
0.8
1.0
1.2
1.4
1.6

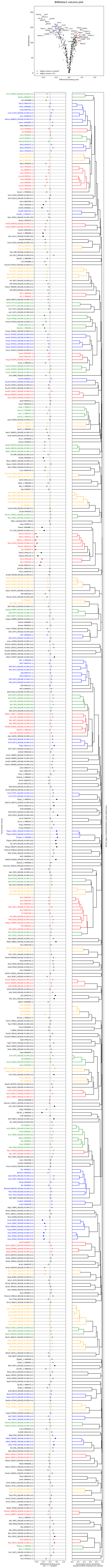
BINDetect volcano plot

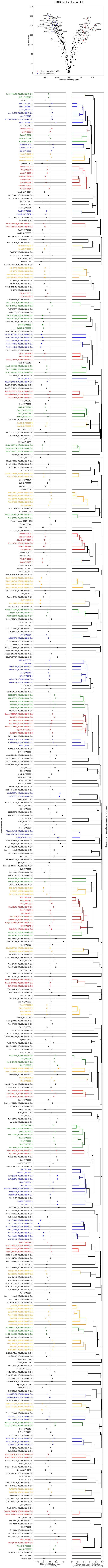
BINDetect volcano plot

BINDetect volcano plot

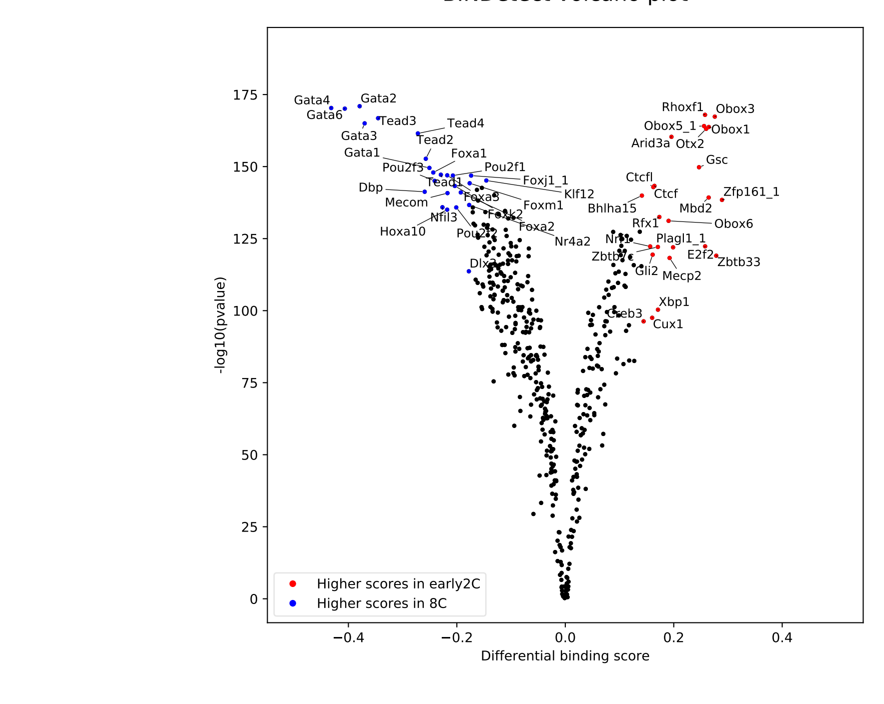

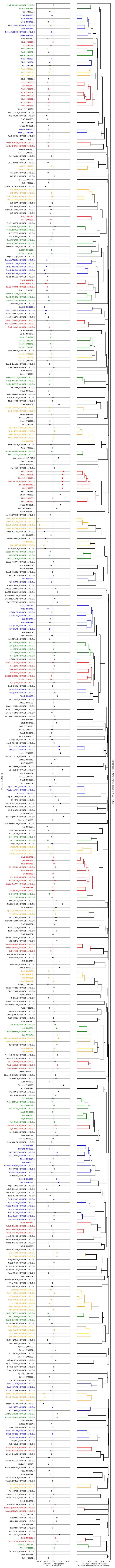

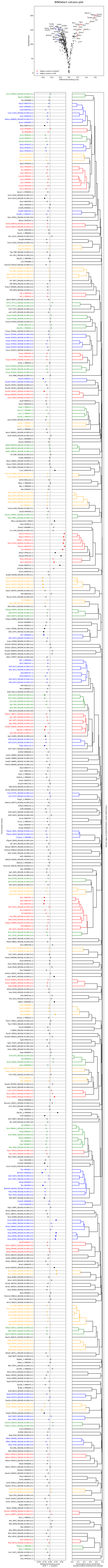
BINDetect volcano plot

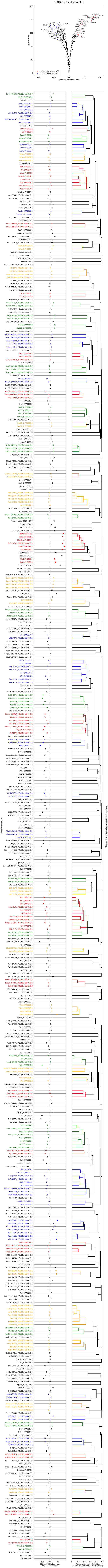BINDetect volcano plot

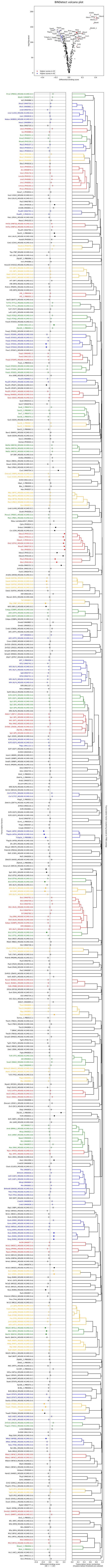
BINDetect volcano plot

BINDetect volcano plot

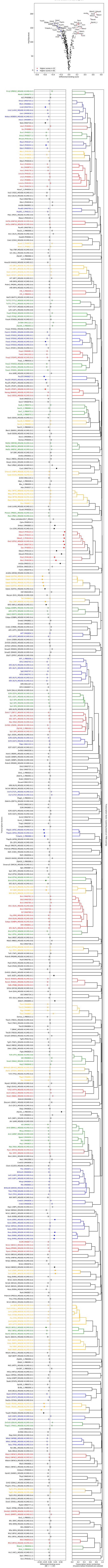

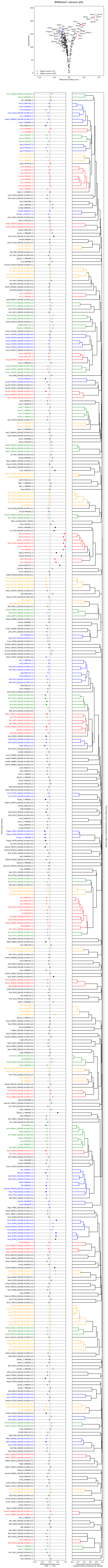
BINDetect volcano plot

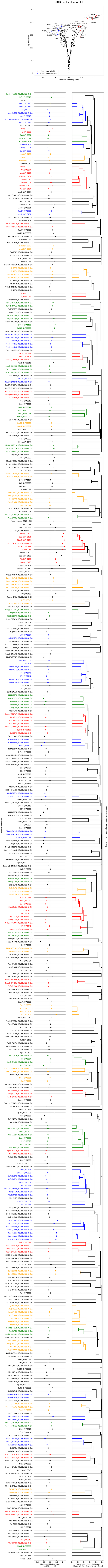
BINDetect volcano plot

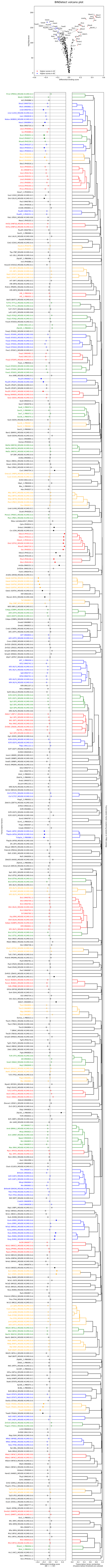
BINDetect volcano plot

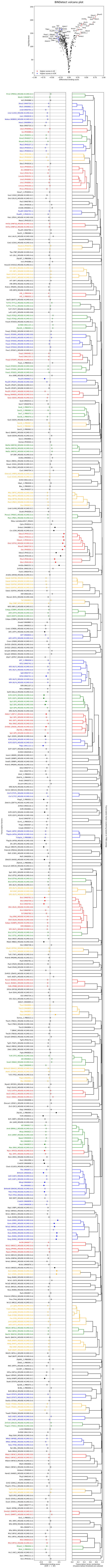
BINDetect volcano plot

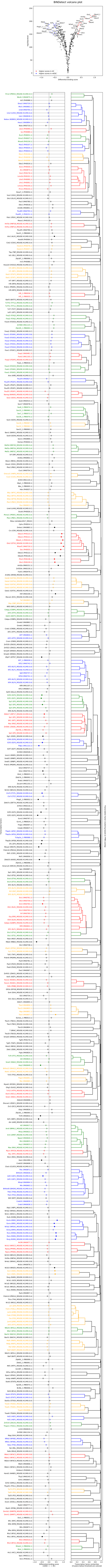
BINDetect volcano plot

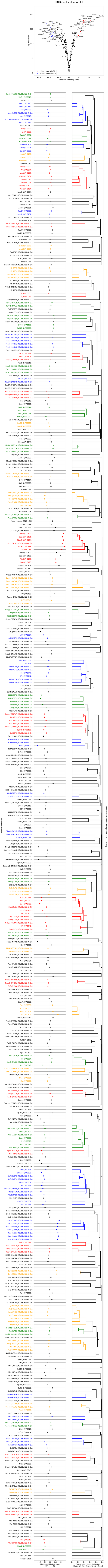

BINDetect volcano plot
-log10(pvalue)
Differential binding score
Higher scores in 8C
Higher scores in ICM
Obox5_1
Rhoxf1
Arid3a
Obox1
Obox3
Esrrb
Otx2
Gsc
Rxra
Rxrg
Rara
Tbx5
Esr2
Nr5a2
Esr1
Obox6
Esrra
Esrrg
Foxj1_1
Obox2
Gm397_1
Otx1
Pitx2
Dmbx1
E2f4
Sp2
Tfdp1
E2f1
Sp1
Mbd2
Egr1
Rfx1
Nrf1
Mecp2
Plagl1_1
Tcfl5
Etv5
Hes1
Mycn
Etv1
E2f5
Elk3
Zbtb33
Tfeb
Zfp57
Atf1
Mlx
Creb1
Bhlhe41
Creb3l2
Hinfp
Differential binding score
(ICM ← → 8C)
Transcription factor similarities
(Clusters below threshold are colored)

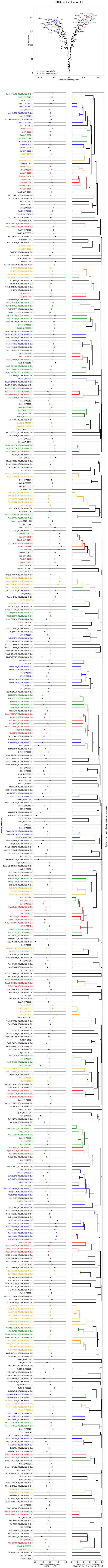
BINDetect volcano plot
-log10(pvalue)
Differential binding score
Higher scores in 8C
Higher scores in mESC

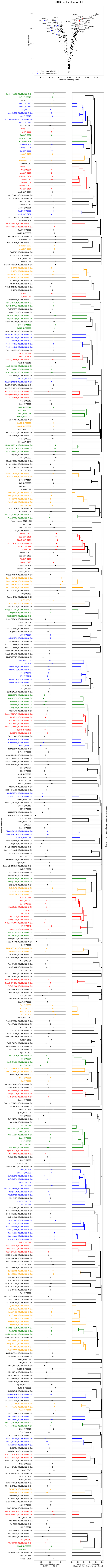
BINDetect volcano plot

## Supplementary Note 2: BINDetect output for human developmental stages

The raw output file of the “TOBIAS BINDetect”-module containing differential binding plots across all pairwise-comparisons of human developmental stages.

# BINDETECT FIGURES

Page 2) Raw score distributions
Page 3) Normalized score distributions
Page 4) BINDetect plot (2C / 4C3PN)
Page 5) BINDetect plot (2C / 8C)
Page 6) BINDetect plot (2C / ICM)
Page 7) BINDetect plot (2C / naive_hESC)
Page 8) BINDetect plot (2C / hESC)
Page 9) BINDetect plot (4C3PN / 8C)
Page 10) BINDetect plot (4C3PN / ICM)
Page 11) BINDetect plot (4C3PN / naive_hESC)
Page 12) BINDetect plot (4C3PN / hESC)
Page 13) BINDetect plot (8C / ICM)
Page 14) BINDetect plot (8C / naive_hESC)
Page 15) BINDetect plot (8C / hESC)
Page 16) BINDetect plot (ICM / naive_hESC)
Page 17) BINDetect plot (ICM / hESC)
Page 18) BINDetect plot (naive_hESC / hESC)

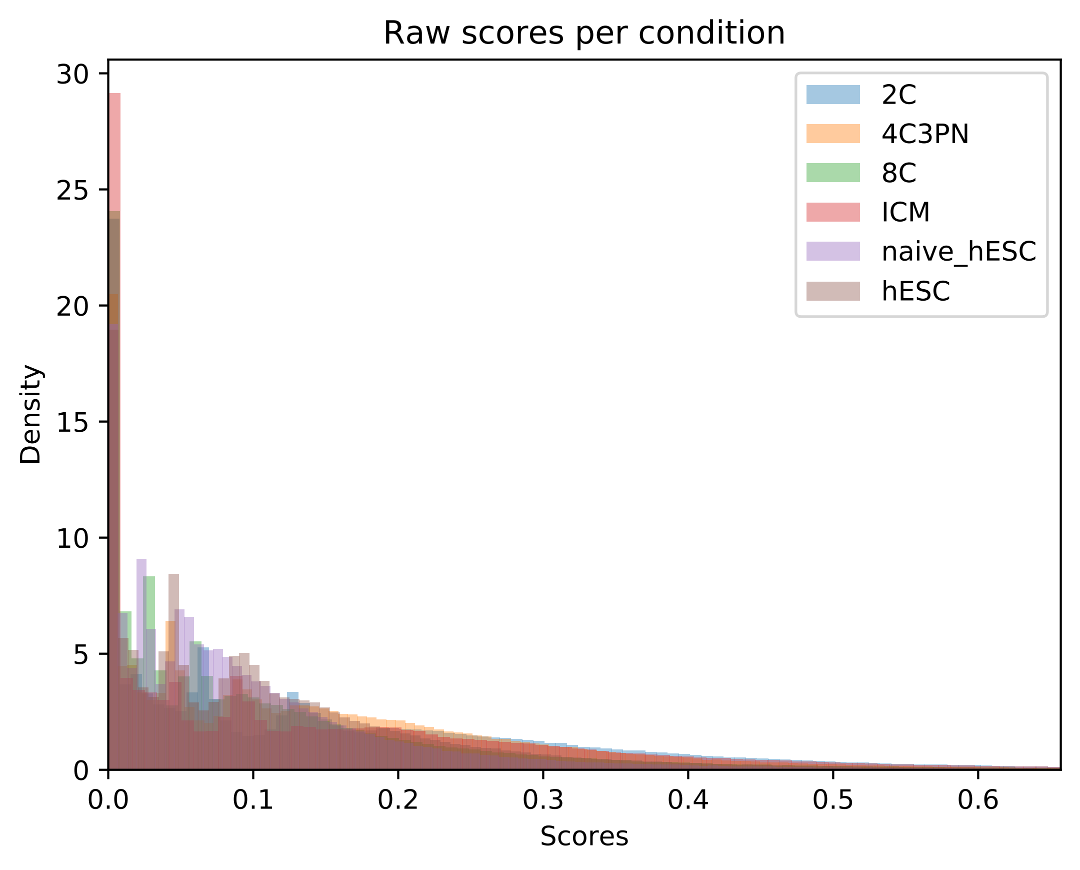
Raw scores per condition
Density
Scores
2C
4C3PN
8C
ICM
naive_hESC
hESC
0
5
10
15
20
25
30
0.0
0.1
0.2
0.3
0.4
0.5
0.6

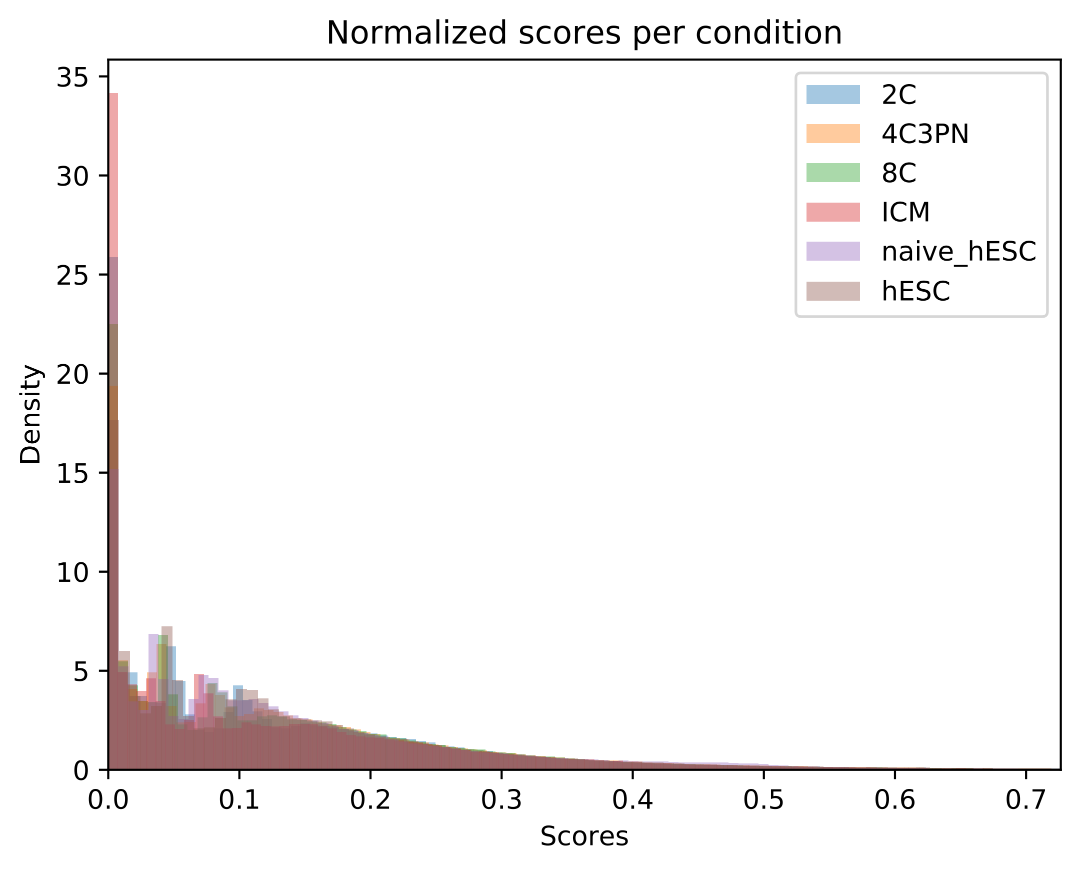

Normalized scores per condition
Density
Scores
0
5
10
15
20
25
30
35
0.0
0.1
0.2
0.3
0.4
0.5
0.6
0.7
2C
4C3PN
8C
ICM
naive_hESC
hESC

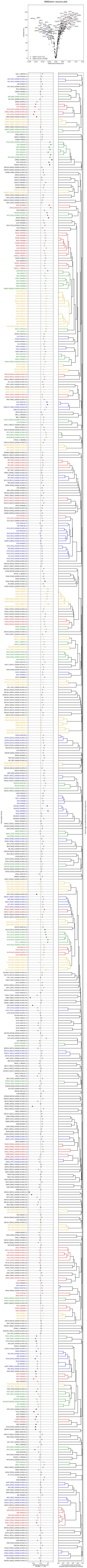

BINDetect volcano plot
-log10(pvalue)
150
125
100
75
50
25
0
−0.20
−0.15
−0.10
−0.05
0.00
0.05
0.10
0.15
0.20
Differential binding score
Higher scores in 2C
Higher scores in 4C3PN
KLF4
KLF5
CRX
DUX4
KLF7_1
PITX2
DMBX1
KLF1
OTX2
PITX1
KLF12
DUXA
GSC
SALL4
FOXK2
GSC2
PITX3
KLF3
FOXG1
RHOXF1
FOXJ2
ALX1
ZNF281
FOXO6
FOXD2
ARGFX
SRY
LHX8
OTX1
LHX2
FOXP2
PHOX2B
FOXD1
LMX1A
OLIG1
BHLHE23
CUX1
CUX2
ZBTB14
MBD2
NRF1
RFX1
MECP2
MYCN
FOSL1
E2F2
ATF6
MXI1
ATF1
HES1
CREB1
CREB3
TCFL5
USF2
ZBTB33
CREB5
HEY1
CREM
ATF7
HEY2
ARNT
ATF3
NPAS2
XBP1
HIF1A
E2F5
PAX2
EPAS1
Transcription factors
SIX6_1 (PB0059.1)
SIX4 (PH0164.1)
SIX2 (SIX2_HUMAN.H11MO.0.A)
SIX1 (SIX1_HUMAN.H11MO.0.A)
HDX (PH0037.1)
GFI1 (GFI1_HUMAN.H11MO.0.C)
NFIL3 (MA0025.1)
GMEB2 (MA0862.1)
TEF (MA0843.1)
DBP (DBP_HUMAN.H11MO.0.B)
HLF (HLF_HUMAN.H11MO.0.C)
GMEB1 (MA0615.1)
CREB5 (MA0840.1)
ATF7 (MA0834.1)
ATF2 (ATF2_HUMAN.H11MO.0.B)
CREB1 (CREB1_HUMAN.H11MO.0.A)
ATF1 (ATF1_HUMAN.H11MO.0.B)
CREM (CREM_HUMAN.H11MO.0.C)
CEBPB (CEBPB_HUMAN.H11MO.0.A)
CEBPA (CEBPA_HUMAN.H11MO.0.A)
CEBPD (CEBPD_HUMAN.H11MO.0.C)
CEBPE (CEBPE_HUMAN.H11MO.0.A)
CEBPG (CEBPG_HUMAN.H11MO.0.B)
ATF4 (ATF4_HUMAN.H11MO.0.A)
MYBL2 (MA0777.1)
BARHL2 (MA0635.1)
HOMEZ (PH0044.1)
OTX2 (OTX2_HUMAN.H11MO.0.A)
GSC (MA0648.1)
GSC2 (MA0891.1)
PITX1 (MA0682.1)
OTX1 (MA0711.1)
PITX3 (MA0714.1)
CRX (CRX_HUMAN.H11MO.0.B)
DMBX1 (MA0883.1)
PITX2 (PH0138.1)
RHOXF1 (MA0719.1)
ZNF329 (ZN329_HUMAN.H11MO.0.C)
ZNF264 (ZN264_HUMAN.H11MO.0.C)
HOXB4 (HXB4_HUMAN.H11MO.0.B)
ARID3A (MA0151.1)
HOXC12 (MA0906.1)
HOXC10 (MA0905.1)
HOXC13 (MA0907.1)
HOXD9 (MA0913.1)
HOXB9 (PH0064.1)
HOXD13 (MA0909.1)
HOXB13 (HXB13_HUMAN.H11MO.0.A)
HOXC9 (HXC9_HUMAN.H11MO.0.C)
CDX2 (CDX2_HUMAN.H11MO.0.A)
HOXA10 (HXA10_HUMAN.H11MO.0.C)
CDX1 (CDX1_HUMAN.H11MO.0.C)
DUXA (MA0884.1)
DUX4 (whiddon2017_DUX4)
CPHX (PH0014.1)
PHOX2B (MA0681.1)
ALX1 (ALX1_HUMAN.H11MO.0.B)
ARGFX (ARGFX_3)
HOXB6 (PH0061.1)
DLX2 (MA0885.1)
DLX3 (DLX3_HUMAN.H11MO.0.C)
DLX5 (PH0024.1)
BARX2 (PH0008.1)
HOXA2 (MA0900.1)
EVX2 (MA0888.1)
HOXB2 (MA0902.1)
EMX2 (MA0886.1)
HOXB3 (MA0903.1)
EMX1 (MA0612.1)
LHX6 (MA0658.1)
MEOX2 (MA0706.1)
HOXB5 (MA0904.1)
HOXA3_1 (PB0031.1)
HOXD1 (PH0074.1)
DLX1 (MA0879.1)
POU6F1 (MA0628.1)
HOXA4 (PH0051.1)
HOXA7_1 (PH0054.1)
LHX8 (MA0705.1)
EN1 (MA0027.2)
LBX1 (MA0618.1)
HESX1 (MA0894.1)
ESX1 (MA0644.1)
GBX2 (MA0890.1)
GBX1 (MA0889.1)
NOBOX (NOBOX_HUMAN.H11MO.0.C)
VAX1 (MA0722.1)
LHX4 (MA0704.1)
LMX1B (MA0703.1)
LMX1A (MA0702.1)
NKX6-2 (MA0675.1)
MNX1 (MA0707.1)
LHX1 (PH0091.1)
SHOX (MA0630.1)
RAX2 (MA0717.1)
UNCX (MA0721.1)
LHX9 (MA0701.1)
MIXL1 (MA0662.1)
LBX2 (MA0699.1)
GSX2 (MA0893.1)
LHX2 (LHX2_HUMAN.H11MO.0.A)
NKX1-2 (PH0110.1)
ARX (MA0874.1)
POU6F2 (MA0793.1)
TLX2 (PH0172.1)
HOXD8 (MA0910.1)
ARID3B (MA0601.1)
HLX (PH0038.1)
HMX2 (MA0897.1)
HMX1 (MA0896.1)
VENTX (MA0724.1)
MSX2 (MA0708.1)
MSX1 (MA0666.1)
BARX1 (MA0875.1)
PRRX2 (PRRX2_HUMAN.H11MO.0.C)
DLX6 (MA0882.1)
DLX4 (MA0881.1)
POU4F2 (MA0683.1)
POU4F1 (MA0790.1)
HNF1B (HNF1B_HUMAN.H11MO.0.A)
HNF1A (HNF1A_HUMAN.H11MO.0.C)
NKX6-1 (NKX61_HUMAN.H11MO.0.B)
ISL1 (ISL1_HUMAN.H11MO.0.A)
PDX1 (PDX1_HUMAN.H11MO.0.A)
HOXA1 (HXA1_HUMAN.H11MO.0.C)
HOXB8 (HXB8_HUMAN.H11MO.0.C)
HOXB7 (HXB7_HUMAN.H11MO.0.C)
MEIS1 (MEIS1_HUMAN.H11MO.0.A)
HOXA9 (HXA9_HUMAN.H11MO.0.B)
PBX2 (PBX2_HUMAN.H11MO.0.C)
CUX2 (MA0755.1)
CUX1 (MA0754.1)
ONECUT1 (HNF6_HUMAN.H11MO.0.B)
ONECUT2 (MA0756.1)
ZNF24 (MA1124.1)
HBP1_1 (PB0028.1)
BBX_1 (PB0005.1)
NKX3-2 (NKX32_HUMAN.H11MO.0.C)
ISL2 (MA0914.1)
NKX3-1 (NKX31_HUMAN.H11MO.0.C)
NKX2-4 (PH0113.1)
NKX2-8 (NKX28_HUMAN.H11MO.0.C)
NFYC (NFYC_HUMAN.H11MO.0.A)
NFYA (NFYA_HUMAN.H11MO.0.A)
NFYB (NFYB_HUMAN.H11MO.0.A)
ZFP187_1 (PB0096.1)
ZNF134 (ZN134_HUMAN.H11MO.0.C)
PKNOX1 (PKNX1_HUMAN.H11MO.0.B)
PBX3 (PBX3_HUMAN.H11MO.0.A)
PBX1 (PBX1_HUMAN.H11MO.0.A)
ZNF680 (ZN680_HUMAN.H11MO.0.C)
SMARCA1 (SMCA1_HUMAN.H11MO.0.C)
ZNF586 (ZN586_HUMAN.H11MO.0.C)
IRF2 (IRF2_HUMAN.H11MO.0.A)
IRF1 (IRF1_HUMAN.H11MO.0.A)
IRF3 (IRF3_HUMAN.H11MO.0.B)
STAT2 (STAT2_HUMAN.H11MO.0.A)
IRF7 (IRF7_HUMAN.H11MO.0.C)
PRDM1 (PRDM1_HUMAN.H11MO.0.A)
IRF8 (IRF8_HUMAN.H11MO.0.B)
IRF4 (IRF4_HUMAN.H11MO.0.A)
BCL11A (BC11A_HUMAN.H11MO.0.A)
IRF9 (IRF9_HUMAN.H11MO.0.C)
ZFP82 (ZFP82_HUMAN.H11MO.0.C)
SPIC (MA0687.1)
ZIM3 (ZIM3_HUMAN.H11MO.0.C)
ZNF350 (ZN350_HUMAN.H11MO.0.C)
ZNF41 (ZNF41_HUMAN.H11MO.0.C)
NFATC3 (NFAC3_HUMAN.H11MO.0.B)
NFATC2 (NFAC2_HUMAN.H11MO.0.B)
NFATC4 (NFAC4_HUMAN.H11MO.0.C)
NFAT5 (MA0606.1)
NFATC1 (NFAC1_HUMAN.H11MO.0.B)
ZNF384 (ZN384_HUMAN.H11MO.0.C)
PRDM6 (PRDM6_HUMAN.H11MO.0.C)
ELF4 (MA0641.1)
ELF1 (ELF1_HUMAN.H11MO.0.A)
ELF3 (ELF3_HUMAN.H11MO.0.A)
ETV3 (MA0763.1)
ERF (MA0760.1)
ETV1 (ETV1_HUMAN.H11MO.0.A)
ELK3 (MA0759.1)
ELK4 (ELK4_HUMAN.H11MO.0.A)
ELK1 (ELK1_HUMAN.H11MO.0.B)
ELF2 (ELF2_HUMAN.H11MO.0.C)
GABPA (GABPA_HUMAN.H11MO.0.A)
ETV6 (MA0645.1)
ETS1 (ETS1_HUMAN.H11MO.0.A)
ERG (ERG_HUMAN.H11MO.0.A)
ETV5 (ETV5_HUMAN.H11MO.0.C)
ETV4 (ETV4_HUMAN.H11MO.0.B)
FLI1 (FLI1_HUMAN.H11MO.0.A)
ETV2 (ETV2_HUMAN.H11MO.0.B)
ETS2 (ETS2_HUMAN.H11MO.0.B)
ZNF418 (ZN418_HUMAN.H11MO.0.C)
ZNF257 (ZN257_HUMAN.H11MO.0.C)
ZNF354A (Z354A_HUMAN.H11MO.0.C)
ZFP28 (ZFP28_HUMAN.H11MO.0.C)
ZFP105_1 (PB0093.1)
FOXJ3 (FOXJ3_HUMAN.H11MO.0.A)
FOXD3 (MA0041.1)
FOXJ2 (FOXJ2_HUMAN.H11MO.0.C)
AR (ANDR_HUMAN.H11MO.0.A)
FOXQ1 (FOXQ1_HUMAN.H11MO.0.C)
FOXO6 (MA0849.1)
FOXD2 (MA0847.1)
FOXD1 (MA0031.1)
FOXK2 (MA1103.1)
FOXP2 (FOXP2_HUMAN.H11MO.0.C)
FOXO3 (FOXO3_HUMAN.H11MO.0.B)
FOXG1 (MA0613.1)
FOXP1 (FOXP1_HUMAN.H11MO.0.A)
FOXK1 (FOXK1_HUMAN.H11MO.0.A)
FOXO1 (FOXO1_HUMAN.H11MO.0.A)
FOXO4 (FOXO4_HUMAN.H11MO.0.C)
FOXC1 (FOXC1_HUMAN.H11MO.0.C)
FOXM1 (FOXM1_HUMAN.H11MO.0.A)
FOXA2 (FOXA2_HUMAN.H11MO.0.A)
FOXA3 (FOXA3_HUMAN.H11MO.0.B)
FOXC2 (MA0846.1)
FOXB1 (MA0845.1)
ZNF250 (ZN250_HUMAN.H11MO.0.C)
FEZF1 (FEZF1_HUMAN.H11MO.0.C)
SOX18_1 (PB0067.1)
SOX12_1 (PB0062.1)
SOX7_1 (PB0073.1)
SOX30_1 (PB0070.1)
SRY (SRY_HUMAN.H11MO.0.B)
SOX5 (SOX5_HUMAN.H11MO.0.C)
SOX3 (SOX3_HUMAN.H11MO.0.B)
SOX2 (SOX2_HUMAN.H11MO.0.A)
SOX4 (SOX4_HUMAN.H11MO.0.B)
SOX6 (MA0515.1)
SOX15 (MA1152.1)
SOX17 (SOX17_HUMAN.H11MO.0.C)
SOX13 (MA1120.1)
SOX9 (SOX9_HUMAN.H11MO.0.B)
SOX21 (MA0866.1)
SOX1 (MA0870.1)
SOX11 (MA0869.1)
TCF7L2 (TF7L2_HUMAN.H11MO.0.A)
TCF7L1 (TF7L1_HUMAN.H11MO.0.B)
TCF7 (TCF7_HUMAN.H11MO.0.A)
LEF1 (LEF1_HUMAN.H11MO.0.A)
ZNF502 (ZN502_HUMAN.H11MO.0.C)
ZNF394 (ZN394_HUMAN.H11MO.0.C)
SMARCA5 (SMCA5_HUMAN.H11MO.0.C)
ZNF582 (ZN582_HUMAN.H11MO.0.C)
POU3F2 (PO3F2_HUMAN.H11MO.0.A)
ZNF140 (ZN140_HUMAN.H11MO.0.C)
ZNF85 (ZNF85_HUMAN.H11MO.0.C)
ZNF214 (ZN214_HUMAN.H11MO.0.C)
ZNF274 (ZN274_HUMAN.H11MO.0.A)
ZNF260 (ZN260_HUMAN.H11MO.0.C)
ZNF146 (OZF_HUMAN.H11MO.0.C)
TEAD4 (TEAD4_HUMAN.H11MO.0.A)
TEAD1 (TEAD1_HUMAN.H11MO.0.A)
TEAD3 (MA0808.1)
TEAD2 (MA1121.1)
HSF4 (MA0771.1)
HSF1 (HSF1_HUMAN.H11MO.0.A)
HSF2 (HSF2_HUMAN.H11MO.0.A)
GATA5 (MA0766.1)
GATA4 (GATA4_HUMAN.H11MO.0.A)
GATA6 (GATA6_HUMAN.H11MO.0.A)
GATA3 (GATA3_HUMAN.H11MO.0.A)
GATA2 (GATA2_HUMAN.H11MO.0.A)
MECOM (EVI1_HUMAN.H11MO.0.B)
HLTF (MA0109.1)
ARID5B (ARI5B_HUMAN.H11MO.0.C)
ARID5A (MA0602.1)
TBP (TBP_HUMAN.H11MO.0.A)
SRF (SRF_HUMAN.H11MO.0.A)
MEF2D (MEF2D_HUMAN.H11MO.0.A)
MEF2A (MEF2A_HUMAN.H11MO.0.A)
ZNF136 (ZN136_HUMAN.H11MO.0.C)
ZNF410 (MA0752.1)
AIRE (AIRE_HUMAN.H11MO.0.C)
POU5F1 (PO5F1_HUMAN.H11MO.0.A)
NANOG (NANOG_HUMAN.H11MO.0.A)
POU3F1 (PO3F1_HUMAN.H11MO.0.C)
POU5F1B (MA0792.1)
POU2F3 (MA0627.1)
POU2F2 (PO2F2_HUMAN.H11MO.0.A)
POU2F1 (PO2F1_HUMAN.H11MO.0.C)
LIN54 (MA0619.1)
IRX4 (PH0085.1)
IRX3_1 (PH0083.1)
IRX5 (PH0086.1)
IRX2 (PH0082.1)
BHLHE23 (MA0817.1)
BHLHE22 (MA0818.1)
OLIG1 (MA0826.1)
OLIG3 (MA0827.1)
NEUROG1 (MA0623.1)
NEUROG2 (MA0669.1)
TWIST2 (MA0633.1)
TWIST1 (TWST1_HUMAN.H11MO.0.A)
ZNF490 (ZN490_HUMAN.H11MO.0.C)
ZNF8 (ZNF8_HUMAN.H11MO.0.C)
NR3C2 (MA0727.1)
NR3C1 (GCR_HUMAN.H11MO.0.A)
PGR (PRGR_HUMAN.H11MO.0.A)
NFKB2 (NFKB2_HUMAN.H11MO.0.B)
NFKB1 (NFKB1_HUMAN.H11MO.1.B)
RELA (TF65_HUMAN.H11MO.0.A)
RELB (RELB_HUMAN.H11MO.0.C)
REL (REL_HUMAN.H11MO.0.B)
ZNF528 (ZN528_HUMAN.H11MO.0.C)
BCL6 (BCL6_HUMAN.H11MO.0.A)
BCL6B (MA0731.1)
STAT3 (STAT3_HUMAN.H11MO.0.A)
STAT1 (STAT1_HUMAN.H11MO.0.A)
STAT5B (STA5B_HUMAN.H11MO.0.A)
STAT5A (STA5A_HUMAN.H11MO.0.A)
ZNF436 (ZN436_HUMAN.H11MO.0.C)
STAT6 (STAT6_HUMAN.H11MO.0.B)
MYBL1 (MA0776.1)
HMBOX1 (MA0895.1)
OVOL1 (OVOL1_HUMAN.H11MO.0.C)
TFDP1 (TFDP1_HUMAN.H11MO.0.C)
E2F1 (E2F1_HUMAN.H11MO.0.A)
E2F7 (E2F7_HUMAN.H11MO.0.B)
E2F6 (E2F6_HUMAN.H11MO.0.A)
E2F3 (E2F3_HUMAN.H11MO.0.A)
E2F5 (E2F5_HUMAN.H11MO.0.B)
ZNF467 (ZN467_HUMAN.H11MO.0.C)
MAZ (MAZ_HUMAN.H11MO.0.A)
VEZF1 (VEZF1_HUMAN.H11MO.0.C)
WT1 (WT1_HUMAN.H11MO.0.C)
PATZ1 (PATZ1_HUMAN.H11MO.0.C)
ZNF281 (ZN281_HUMAN.H11MO.0.A)
ZBTB17 (ZBT17_HUMAN.H11MO.0.A)
ZNF263 (ZN263_HUMAN.H11MO.0.A)
ZNF341 (ZN341_HUMAN.H11MO.0.C)
EGR3 (MA0732.1)
EGR1 (EGR1_HUMAN.H11MO.0.A)
EGR2 (EGR2_HUMAN.H11MO.0.A)
ZNF740 (MA0753.1)
SALL4 (SALL4_HUMAN.H11MO.0.B)
KLF3 (KLF3_HUMAN.H11MO.0.B)
KLF1 (KLF1_HUMAN.H11MO.0.A)
KLF5 (KLF5_HUMAN.H11MO.0.A)
KLF4 (KLF4_HUMAN.H11MO.0.A)
KLF7_1 (PB0039.1)
KLF6 (KLF6_HUMAN.H11MO.0.A)
SP8 (MA0747.1)
KLF16 (MA0741.1)
KLF9 (KLF9_HUMAN.H11MO.0.C)
KLF14 (MA0740.1)
SP3 (SP3_HUMAN.H11MO.0.B)
SP2 (SP2_HUMAN.H11MO.0.A)
E2F4 (E2F4_HUMAN.H11MO.0.A)
SP4 (SP4_HUMAN.H11MO.0.A)
SP1 (SP1_HUMAN.H11MO.0.A)
KLF12 (KLF12_HUMAN.H11MO.0.C)
ZSCAN22 (ZSC22_HUMAN.H11MO.0.C)
ZNF770 (ZN770_HUMAN.H11MO.0.C)
TAF1 (TAF1_HUMAN.H11MO.0.A)
HINFP (HINFP_HUMAN.H11MO.0.C)
KLF13 (MA0657.1)
ZNF816 (ZN816_HUMAN.H11MO.0.C)
MZF1 (MZF1_HUMAN.H11MO.0.B)
ZNF324 (Z324A_HUMAN.H11MO.0.C)
PLAG1 (MA0163.1)
THAP1 (THAP1_HUMAN.H11MO.0.C)
MBD2 (MBD2_HUMAN.H11MO.0.B)
SREBF2 (SRBP2_HUMAN.H11MO.0.B)
SREBF1 (SRBP1_HUMAN.H11MO.0.A)
E2F2 (E2F2_HUMAN.H11MO.0.B)
CTCF (CTCF_HUMAN.H11MO.0.A)
CTCFL (CTCFL_HUMAN.H11MO.0.A)
KLF8 (KLF8_HUMAN.H11MO.0.C)
ZSCAN4 (MA1155.1)
RREB1 (MA0073.1)
GLIS3 (MA0737.1)
GLIS1 (MA0735.1)
GLIS2 (MA0736.1)
GCM2 (MA0767.1)
GCM1 (MA0646.1)
ZNF768 (ZN768_HUMAN.H11MO.0.C)
VDR (VDR_HUMAN.H11MO.0.A)
GLI3 (GLI3_HUMAN.H11MO.0.B)
GLI2 (MA0734.1)
ZBTB7C (MA0695.1)
ZBTB7B (MA0694.1)
ZBTB7A (ZBT7A_HUMAN.H11MO.0.A)
ZNF143 (ZN143_HUMAN.H11MO.0.A)
THAP11 (THA11_HUMAN.H11MO.0.B)
ZNF76 (ZNF76_HUMAN.H11MO.0.C)
IKZF1 (IKZF1_HUMAN.H11MO.0.C)
RBPJ (SUH_HUMAN.H11MO.0.A)
ZBTB3_1 (PB0091.1)
NKX2-1 (NKX21_HUMAN.H11MO.0.A)
NKX2-5 (NKX25_HUMAN.H11MO.0.B)
ZSCAN31 (ZSC31_HUMAN.H11MO.0.C)
ZNF423 (MA0116.1)
ZIC2_1 (PB0102.1)
EBF1 (COE1_HUMAN.H11MO.0.A)
TFAP2C (AP2C_HUMAN.H11MO.0.A)
TFAP2A (AP2A_HUMAN.H11MO.0.A)
TFAP2B (AP2B_HUMAN.H11MO.0.B)
ZFX (ZFX_HUMAN.H11MO.0.A)
ZNF282 (MA1154.1)
E2F8 (MA0865.1)
ZBTB48 (ZBT48_HUMAN.H11MO.0.C)
PRDM14 (PRD14_HUMAN.H11MO.0.A)
NR1H4 (NR1H4_HUMAN.H11MO.0.B)
ZBTB6 (ZBTB6_HUMAN.H11MO.0.C)
MECP2 (MECP2_HUMAN.H11MO.0.C)
NR2E3 (NR2E3_HUMAN.H11MO.0.C)
NR2E1 (MA0676.1)
ZNF317 (ZN317_HUMAN.H11MO.0.C)
RXRB (RXRB_HUMAN.H11MO.0.C)
NR2F2 (COT2_HUMAN.H11MO.0.A)
ESRRG (ERR3_HUMAN.H11MO.0.B)
RARB (MA0857.1)
RARA (RARA_HUMAN.H11MO.0.A)
NR2C1 (NR2C1_HUMAN.H11MO.0.C)
RORA (RORA_HUMAN.H11MO.0.C)
NR1D1 (NR1D1_HUMAN.H11MO.0.B)
RXRA (RXRA_HUMAN.H11MO.0.A)
NR2F1 (COT1_HUMAN.H11MO.0.C)
NR2F6 (MA0677.1)
PPARG (PPARG_HUMAN.H11MO.0.A)
PPARA (PPARA_HUMAN.H11MO.0.B)
NR4A2 (NR4A2_HUMAN.H11MO.0.C)
NR4A1 (NR4A1_HUMAN.H11MO.0.A)
ESRRB (ERR2_HUMAN.H11MO.0.A)
ESRRA (ERR1_HUMAN.H11MO.0.A)
NR5A2 (NR5A2_HUMAN.H11MO.0.B)
NR5A1 (STF1_HUMAN.H11MO.0.B)
NR6A1 (NR6A1_HUMAN.H11MO.0.B)
HNF4A (HNF4A_HUMAN.H11MO.0.A)
NR2C2 (NR2C2_HUMAN.H11MO.0.B)
INSM1 (INSM1_HUMAN.H11MO.0.C)
THRB (THB_HUMAN.H11MO.0.C)
THRA (THA_HUMAN.H11MO.0.C)
NR1H3 (NR1H3_HUMAN.H11MO.0.B)
NR1I3 (NR1I3_HUMAN.H11MO.0.C)
RARG (RARG_HUMAN.H11MO.0.B)
ESR2 (ESR2_HUMAN.H11MO.0.A)
RORB (MA1150.1)
ZBTB14 (ZBT14_HUMAN.H11MO.0.C)
NRF1 (NRF1_HUMAN.H11MO.0.A)
MTF1 (MTF1_HUMAN.H11MO.0.C)
ZNF335 (ZN335_HUMAN.H11MO.0.A)
ZNF322 (ZN322_HUMAN.H11MO.0.B)
REST (REST_HUMAN.H11MO.0.A)
ZNF708 (ZN708_HUMAN.H11MO.0.C)
NFIC (NFIC_HUMAN.H11MO.0.A)
NFIA (NFIA_HUMAN.H11MO.0.C)
NFIX (MA0671.1)
ZNF449 (ZN449_HUMAN.H11MO.0.C)
HIC2 (MA0738.1)
ZNF331 (ZN331_HUMAN.H11MO.0.C)
TP73 (P73_HUMAN.H11MO.0.A)
TP63 (P63_HUMAN.H11MO.0.A)
TP53 (P53_HUMAN.H11MO.0.A)
CENPB (MA0637.1)
OSR2 (OSR2_HUMAN.H11MO.0.C)
OSR1_1 (PB0050.1)
ZNF547 (ZN547_HUMAN.H11MO.0.C)
ZNF563 (ZN563_HUMAN.H11MO.0.C)
MYB (MYB_HUMAN.H11MO.0.A)
ASCL2 (MA0816.1)
ASCL1 (ASCL1_HUMAN.H11MO.0.A)
TCF4 (ITF2_HUMAN.H11MO.0.C)
MYF6 (MYF6_HUMAN.H11MO.0.C)
TCF3 (TFE2_HUMAN.H11MO.0.A)
TCF12 (HTF4_HUMAN.H11MO.0.A)
PTF1A (PTF1A_HUMAN.H11MO.0.B)
ZBTB18 (ZBT18_HUMAN.H11MO.0.C)
TFAP4 (TFAP4_HUMAN.H11MO.0.A)
LYL1 (LYL1_HUMAN.H11MO.0.A)
ZEB1 (ZEB1_HUMAN.H11MO.0.A)
SNAI1 (SNAI1_HUMAN.H11MO.0.C)
SNAI2 (SNAI2_HUMAN.H11MO.0.A)
ID4 (MA0824.1)
FIGLA (MA0820.1)
TGIF2 (MA0797.1)
PKNOX2 (MA0783.1)
MEIS2 (MEIS2_HUMAN.H11MO.0.B)
TGIF1 (PH0169.1)
MEIS3 (MA0775.1)
SMAD4 (SMAD4_HUMAN.H11MO.0.B)
SMAD2 (SMAD2_HUMAN.H11MO.0.A)
SMAD3 (SMAD3_HUMAN.H11MO.0.B)
TBX2 (MA0688.1)
EOMES (MA0800.1)
TBX20 (MA0689.1)
TBX5 (MA0807.1)
TBX1 (MA0805.1)
MGA (MA0801.1)
ZNF18 (ZNF18_HUMAN.H11MO.0.C)
TBX3 (TBX3_HUMAN.H11MO.0.C)
ZNF667 (ZN667_HUMAN.H11MO.0.C)
ZFP691_1 (PB0099.1)
ZKSCAN1 (ZKSC1_HUMAN.H11MO.0.B)
ZIC3 (ZIC3_HUMAN.H11MO.0.B)
EPAS1 (EPAS1_HUMAN.H11MO.0.B)
ARNT (ARNT_HUMAN.H11MO.0.B)
HIF1A (HIF1A_HUMAN.H11MO.0.C)
AHR (AHR_HUMAN.H11MO.0.B)
CREB3L2 (MA0608.1)
CLOCK (CLOCK_HUMAN.H11MO.0.C)
NPAS2 (MA0626.1)
MYC (MYC_HUMAN.H11MO.0.A)
MNT (MA0825.1)
MYCN (MYCN_HUMAN.H11MO.0.A)
MLXIP (MA0622.1)
ID2 (MA0617.1)
ARNTL (BMAL1_HUMAN.H11MO.0.A)
MAX (MAX_HUMAN.H11MO.0.A)
MXI1 (MXI1_HUMAN.H11MO.0.A)
HEY2 (MA0649.1)
HEY1 (MA0823.1)
HES1 (MA1099.1)
HES2 (MA0616.1)
TFE3 (TFE3_HUMAN.H11MO.0.B)
MITF (MITF_HUMAN.H11MO.0.A)
TFEB (TFEB_HUMAN.H11MO.0.C)
USF2 (USF2_HUMAN.H11MO.0.A)
USF1 (USF1_HUMAN.H11MO.0.A)
TFEC (MA0871.1)
BHLHE41 (MA0636.1)
BHLHE40 (BHE40_HUMAN.H11MO.0.A)
MLX (MA0663.1)
MLXIPL (MA0664.1)
ATF3 (ATF3_HUMAN.H11MO.0.A)
TCFL5 (MA0632.1)
HES7 (MA0822.1)
PAX2 (MA0067.1)
CREB3 (MA0638.1)
CREB3L1 (MA0839.1)
XBP1 (MA0844.1)
ATF6 (ATF6A_HUMAN.H11MO.0.B)
ZBTB33 (KAISO_HUMAN.H11MO.0.A)
ZBED1 (MA0749.1)
ZNF121 (ZN121_HUMAN.H11MO.0.C)
PAX5 (PAX5_HUMAN.H11MO.0.A)
SPZ1 (MA0111.1)
HIC1 (HIC1_HUMAN.H11MO.0.C)
IRF6_1 (PB0036.1)
RFX2 (RFX2_HUMAN.H11MO.0.A)
RFX1 (RFX1_HUMAN.H11MO.0.B)
RFX4 (MA0799.1)
RFX3 (RFX3_HUMAN.H11MO.0.B)
RFXDC2_1 (PB0056.1)
RFX5 (RFX5_HUMAN.H11MO.0.A)
TFCP2 (MA0145.3)
GRHL1 (MA0647.1)
GRHL2 (GRHL2_HUMAN.H11MO.0.A)
PROX1 (MA0794.1)
ZFP42 (ZFP42_HUMAN.H11MO.0.A)
YY1 (TYY1_HUMAN.H11MO.0.A)
YY2 (MA0748.1)
SOX8 (MA0868.1)
MAFK (MAFK_HUMAN.H11MO.0.A)
MAFG (MAFG_HUMAN.H11MO.0.A)
MAF (MAF_HUMAN.H11MO.0.A)
MAFF (MAFF_HUMAN.H11MO.0.B)
MAFB (MAFB_HUMAN.H11MO.0.B)
NRL (MA0842.1)
NFE2 (NFE2_HUMAN.H11MO.0.A)
BACH2 (BACH2_HUMAN.H11MO.0.A)
NFE2L2 (NF2L2_HUMAN.H11MO.0.A)
BACH1 (BACH1_HUMAN.H11MO.0.A)
JUN (JUN_HUMAN.H11MO.0.A)
JUND (JUND_HUMAN.H11MO.0.A)
JDP2 (MA0655.1)
FOS (FOS_HUMAN.H11MO.0.A)
FOSB (FOSB_HUMAN.H11MO.0.A)
FOSL2 (FOSL2_HUMAN.H11MO.0.A)
FOSL1 (FOSL1_HUMAN.H11MO.0.A)
JUNB (JUNB_HUMAN.H11MO.0.A)
ZNF554 (ZN554_HUMAN.H11MO.0.C)
BATF (BATF_HUMAN.H11MO.0.A)
BATF3 (BATF3_HUMAN.H11MO.0.B)
NFE2L1 (NF2L1_HUMAN.H11MO.0.C)
ZNF549 (ZN549_HUMAN.H11MO.0.C)
PAX9 (MA0781.1)
PAX6 (PAX6_HUMAN.H11MO.0.C)
ZNF354C (MA0130.1)
FOXH1 (FOXH1_HUMAN.H11MO.0.A)
RUNX3 (RUNX3_HUMAN.H11MO.0.A)
RUNX1 (RUNX1_HUMAN.H11MO.0.A)
RUNX2 (RUNX2_HUMAN.H11MO.0.A)
CBFB (PEBB_HUMAN.H11MO.0.C)
ZNF382 (ZN382_HUMAN.H11MO.0.C)
−0.1
0.0
0.1
Differential binding score
(4C3PN ← → 2C)
0.0
0.2
0.4
0.6
0.8
1.0
Transcription factor similarities
(Clusters below threshold are colored)
Transcription factor clustering based on TFBS overlap

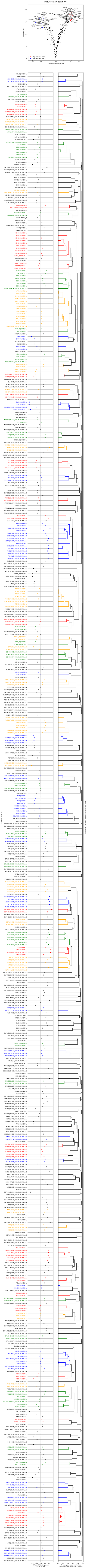
BINDetect volcano plot
-log10(pvalue)
Differential binding score
Higher scores in 2C
Higher scores in 8C
ZNF467
VEZF1
PATZ1
KLF6
WT1
KLF1
SP4
SP3
ZNF281
MAZ
SALL4
EGR1
KLF5
FLI1
KLF9
ZNF341
ZNF436
ZBTB17
TCF4
ZIC3
ZNF740
ETV5
EGR2
SP1
ETV4
ZNF331
PTF1A
ERG
EBF1
INSM1
SREBF2
KLF16
ASCL1
ETV1
ETS1
TCF3
ELF2
PBX3
FOXJ3
ZFP105_1
ZNF354A
MEF2D
SRY
MEF2A
AR
TLX2
FOXD3
PAX5
GATA6
ONECUT2
GATA3
HNF1A
RARB
ESRRG
CUX2
RHOXF1
ZBTB14
GATA5
CUX1
ARID5A
ZFP187_1
HLX
TCFL5
BHLHE23
HOMEZ
YY2
HES1
IRF6_1
SIX6_1 (PB0059.1)
SIX4 (PH0164.1)
SIX2 (SIX2_HUMAN.H11MO.0.A)
SIX1 (SIX1_HUMAN.H11MO.0.A)
HDX (PH0037.1)
GFI1 (GFI1_HUMAN.H11MO.0.C)
NFIL3 (MA0025.1)
GMEB2 (MA0862.1)
TEF (MA0843.1)
DBP (DBP_HUMAN.H11MO.0.B)
HLF (HLF_HUMAN.H11MO.0.C)
GMEB1 (MA0615.1)
CREB5 (MA0840.1)
ATF7 (MA0834.1)
ATF2 (ATF2_HUMAN.H11MO.0.B)
CREB1 (CREB1_HUMAN.H11MO.0.A)
ATF1 (ATF1_HUMAN.H11MO.0.B)
CREM (CREM_HUMAN.H11MO.0.C)
CEBPB (CEBPB_HUMAN.H11MO.0.A)
CEBPA (CEBPA_HUMAN.H11MO.0.A)
CEBPD (CEBPD_HUMAN.H11MO.0.C)
CEBPE (CEBPE_HUMAN.H11MO.0.A)
CEBPG (CEBPG_HUMAN.H11MO.0.B)
ATF4 (ATF4_HUMAN.H11MO.0.A)
MYBL2 (MA0777.1)
BARHL2 (MA0635.1)
HOMEZ (PH0044.1)
OTX2 (OTX2_HUMAN.H11MO.0.A)
GSC (MA0648.1)
GSC2 (MA0891.1)
PITX1 (MA0682.1)
OTX1 (MA0711.1)
PITX3 (MA0714.1)
CRX (CRX_HUMAN.H11MO.0.B)
DMBX1 (MA0883.1)
PITX2 (PH0138.1)

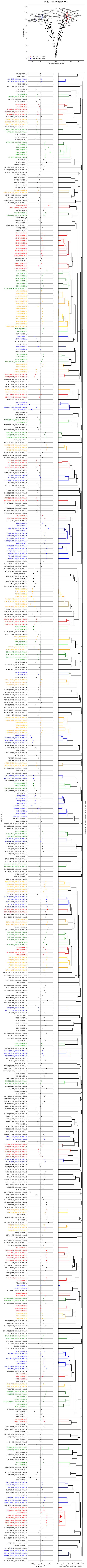
BINDetect volcano plot
Higher scores in 2C
Higher scores in ICM
Differential binding score
-log10(pvalue)

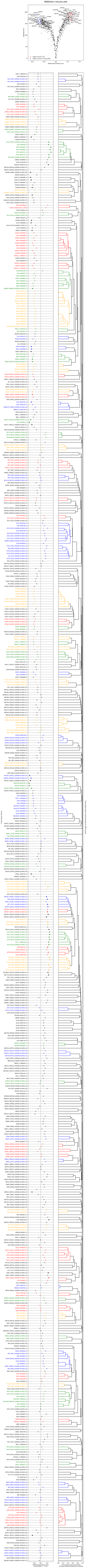

BINDetect volcano plot
-log10(pvalue)
Differential binding score
Higher scores in 2C
Higher scores in naive_hESC
NANOG
POU5F1
POU5F1B
POU3F1
POU2F3
POU2F2
HOXB9
NKX3-2
NKX3-1
HOXB4
ISL2
HOXC10
HMX2
HMX1
VENTX
HOXB6
HOXB7
NOBOX
HOXC12
DLX2
BARX1
BARX2
BARHL2
CUX2
MYBL1
ZNF263
ZNF770
ZSCAN22
ZBTB17
SP3
KLF6
MAZ
SP1
KLF3
WT1
SP4
KLF5
SP2
KLF4
ZNF467
EGR2
KLF1
ZNF341
EGR1
KLF12
SP8
PITX1
KLF9
KLF7_1
SNAI2
ZEB1
E2F7
CRX
GSC2
GSC
PATZ1
PITX3
HIC2
OTX1
RHOXF1
SIX6_1 (PB0059.1)
SIX4 (PH0164.1)
SIX2 (SIX2_HUMAN.H11MO.0.A)
SIX1 (SIX1_HUMAN.H11MO.0.A)
HDX (PH0037.1)
GFI1 (GFI1_HUMAN.H11MO.0.C)
NFIL3 (MA0025.1)
GMEB2 (MA0862.1)
TEF (MA0843.1)
DBP (DBP_HUMAN.H11MO.0.B)
HLF (HLF_HUMAN.H11MO.0.C)
GMEB1 (MA0615.1)
CREB5 (MA0840.1)
ATF7 (MA0834.1)
ATF2 (ATF2_HUMAN.H11MO.0.B)
CREB1 (CREB1_HUMAN.H11MO.0.A)
ATF1 (ATF1_HUMAN.H11MO.0.B)
CREM (CREM_HUMAN.H11MO.0.C)
CEBPB (CEBPB_HUMAN.H11MO.0.A)
CEBPA (CEBPA_HUMAN.H11MO.0.A)
CEBPD (CEBPD_HUMAN.H11MO.0.C)
CEBPE (CEBPE_HUMAN.H11MO.0.A)
CEBPG (CEBPG_HUMAN.H11MO.0.B)
ATF4 (ATF4_HUMAN.H11MO.0.A)

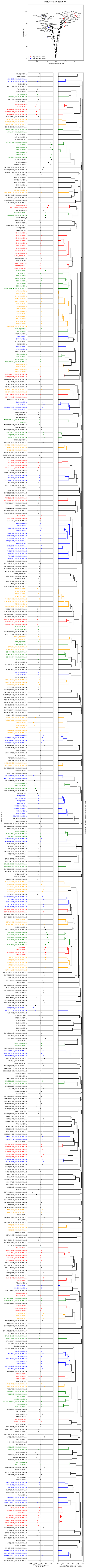

BINDetect volcano plot
-log10(pvalue)
Differential binding score
Higher scores in 2C
Higher scores in hESC
CTCF
POU5F1
NANOG
TEAD3
POU2F3
TEAD1
CTCFL
TEAD2
POU2F2
TEAD4
POU3F1
POU5F1B
ZBTB14
POU2F1
FOSL1
SIX2
LIN54
SIX1
RFX4
NRF1
E2F2
NFE2L1
MECP2
RFXDC2_1
ATF7
CREB5
CREB1
GMEB2
SALL4
KLF5
KLF4
CRX
KLF12
KLF1
OTX2
PITX1
KLF3
GSC2
KLF6
KLF7
PITX3
SP4
SP1
RHOXF1
SP3
PITX2
DMBX1
GSC
IKZF1
SP2
SP8
DUXA
OTX1
KLF9
KLF14
DUX4
PHOX2B
ALX1
LHX8

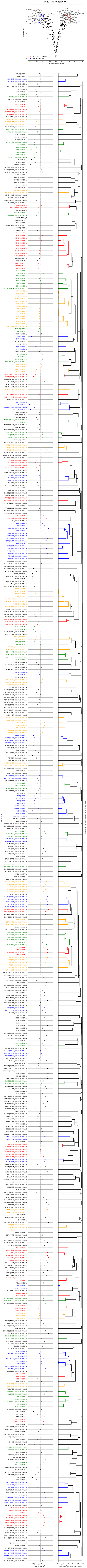
BINDetect volcano plot
-log10(pvalue)
Differential binding score
Higher scores in 4C3PN
Higher scores in 8C
−0.4
−0.3
−0.2
−0.1
0.0
0.1
0.2
0.3
0.4
0
50
100
150
200

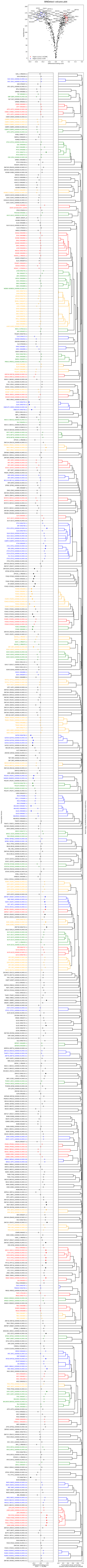
BINDetect volcano plot
-log10(pvalue)
Differential binding score
Higher scores in 4C3PN
Higher scores in ICM

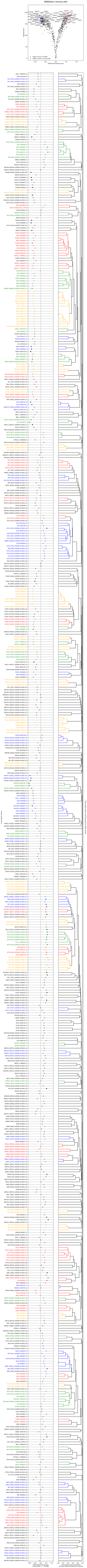
BINDetect volcano plot
-log10(pvalue)
Differential binding score
Higher scores in 4C3PN
Higher scores in naive_hESC
POU5F1
POU5F1B
NANOG
POU3F1
HMX1
SOX15
POU2F3
POU2F2
FOXB1
FOXJ2
NKX3-2
DLX2
NKX3-1
ISL2
FOXG1
HOXB9
HMX2
POU4F2
HOXC9
HOXB4
BARX2
HOXB3
HOXB7
HOXB6
BARX1
VENTX
CUX2
PRRX2
HOXC12
BHLHE23
BARHL2
CUX1
PATZ1
ZNF770
SP2
ZSCAN22
KLF5
SP4
SP3
SP1
SALL4
E2F7
KLF6
KLF1
ZBTB17
MAZ
PAX5
KLF3
EGR2
PLAG1
WT1
ZNF263
KLF12
ZEB1
SREBF2
THRB
SNAI2
ID4
HIC2
ZNF449
GSC2
MAX
OTX1
ETV1
SIX6_1 (PB0059.1)
SIX4 (PH0164.1)
SIX2 (SIX2_HUMAN.H11MO.0.A)
SIX1 (SIX1_HUMAN.H11MO.0.A)
HDX (PH0037.1)
GFI1 (GFI1_HUMAN.H11MO.0.C)
NFIL3 (MA0025.1)
GMEB2 (MA0862.1)
TEF (MA0843.1)
DBP (DBP_HUMAN.H11MO.0.B)
HLF (HLF_HUMAN.H11MO.0.C)
GMEB1 (MA0615.1)
CREB5 (MA0840.1)
ATF7 (MA0834.1)
ATF2 (ATF2_HUMAN.H11MO.0.B)
CREB1 (CREB1_HUMAN.H11MO.0.A)
ATF1 (ATF1_HUMAN.H11MO.0.B)
CREM (CREM_HUMAN.H11MO.0.C)
CEBPB (CEBPB_HUMAN.H11MO.0.A)
CEBPA (CEBPA_HUMAN.H11MO.0.A)
CEBPD (CEBPD_HUMAN.H11MO.0.C)
CEBPE (CEBPE_HUMAN.H11MO.0.A)
CEBPG (CEBPG_HUMAN.H11MO.0.B)
ATF4 (ATF4_HUMAN.H11MO.0.A)
MYBL2 (MA0777.1)
BARHL2 (MA0635.1)
HOMEZ (PH0044.1)
OTX2 (OTX2_HUMAN.H11MO.0.A)
GSC (MA0648.1)
GSC2 (MA0891.1)
PITX1 (MA0682.1)

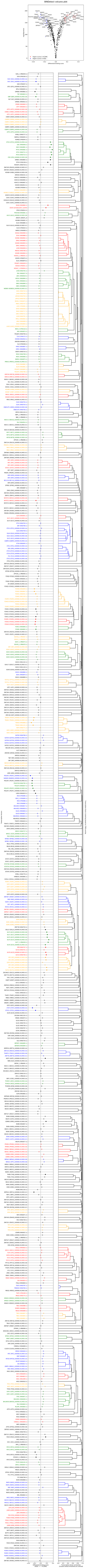
BINDetect volcano plot
-log10(pvalue)
Differential binding score
Higher scores in 4C3PN
Higher scores in hESC
POU5F1
NANOG
CTCF
TEAD3
POU2F3
TEAD4
POU5F1B
POU3F1
POU2F2
POU2F1
TEAD2
TEAD1
FOXA3
CTCFL
FOXK1
FOXA2
FOXM1
NFATC2
FOXD2
FOXC1
FOXO6
LIN54
FOXB1
FOXP1
ZBTB14
SIX1
DBP
RFX4
GMEB2
KLF4
KLF7_1
PITX1
KLF5
OTX2
KLF3
KLF1
SP1
CRX
RHOXF1
KLF6
SP2
KLF12
GSC
PITX3
SP3
DMBX1
KLF14
SP8
KLF9
GSC2
OTX1
SALL4
PAX5
PITX2
DUXA
IKZF1
DUX4
THRB
ESRRG
SIX6_1 (PB0059.1)
SIX4 (PH0164.1)
SIX2 (SIX2_HUMAN.H11MO.0.A)
SIX1 (SIX1_HUMAN.H11MO.0.A)
HDX (PH0037.1)
GFI1 (GFI1_HUMAN.H11MO.0.C)
NFIL3 (MA0025.1)
GMEB2 (MA0862.1)
TEF (MA0843.1)
DBP (DBP_HUMAN.H11MO.0.B)
HLF (HLF_HUMAN.H11MO.0.C)
GMEB1 (MA0615.1)
CREB5 (MA0840.1)
ATF7 (MA0834.1)
ATF2 (ATF2_HUMAN.H11MO.0.B)
CREB1 (CREB1_HUMAN.H11MO.0.A)
ATF1 (ATF1_HUMAN.H11MO.0.B)
CREM (CREM_HUMAN.H11MO.0.C)
CEBPB (CEBPB_HUMAN.H11MO.0.A)
CEBPA (CEBPA_HUMAN.H11MO.0.A)
CEBPD (CEBPD_HUMAN.H11MO.0.C)
CEBPE (CEBPE_HUMAN.H11MO.0.A)
CEBPG (CEBPG_HUMAN.H11MO.0.B)
ATF4 (ATF4_HUMAN.H11MO.0.A)
MYBL2 (MA0777.1)
BARHL2 (MA0635.1)
HOMEZ (PH0044.1)
OTX2 (OTX2_HUMAN.H11MO.0.A)
GSC (MA0648.1)
GSC2 (MA0891.1)
PITX1 (MA0682.1)

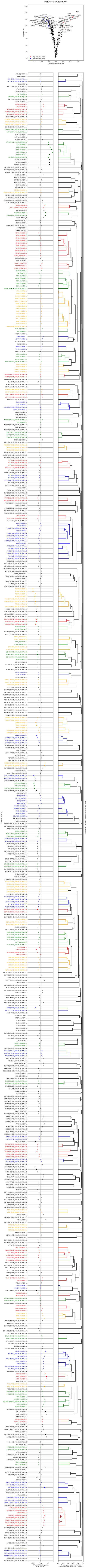
BINDetect volcano plot
-log10(pvalue)
Differential binding score
Higher scores in 8C
Higher scores in ICM
PITX1
OTX2
CRX
DMBX1
OTX1
GSC2
PAX5
PITX3
RHOXF1
PITX2
ZNF121
MEF2D
GSC
MAF
MEF2A
FOS
ALX1
RARB
HIC2
GRHL2
PHOX2B
HES1
HEY1
HES7
NPAS2
HEY2
TCFL5
HINFP
POU5F1
FOXP2
NANOG
POU5F1B
FOXO3
ZNF281
POU2F2
CTCF
POU3F1
FOXA3
KLF16
POU2F3
HNF4A
SOX2
FOXK2
TEAD2
FOXO6
FOXD1
FOXK1
NR2F6
FOXA2
FOXO1
FOXG1
FOXM1
FOXD2
TEAD3
TEAD1
POU2F1
GATA5
NR2C2
SIX6_1 (PB0059.1)
SIX4 (PH0164.1)
SIX2 (SIX2_HUMAN.H11MO.0.A)
SIX1 (SIX1_HUMAN.H11MO.0.A)
HDX (PH0037.1)
GFI1 (GFI1_HUMAN.H11MO.0.C)
NFIL3 (MA0025.1)
GMEB2 (MA0862.1)
TEF (MA0843.1)
DBP (DBP_HUMAN.H11MO.0.B)
HLF (HLF_HUMAN.H11MO.0.C)
GMEB1 (MA0615.1)
CREB5 (MA0840.1)
ATF7 (MA0834.1)
ATF2 (ATF2_HUMAN.H11MO.0.B)
CREB1 (CREB1_HUMAN.H11MO.0.A)
ATF1 (ATF1_HUMAN.H11MO.0.B)
CREM (CREM_HUMAN.H11MO.0.C)
CEBPB (CEBPB_HUMAN.H11MO.0.A)
CEBPA (CEBPA_HUMAN.H11MO.0.A)
CEBPD (CEBPD_HUMAN.H11MO.0.C)

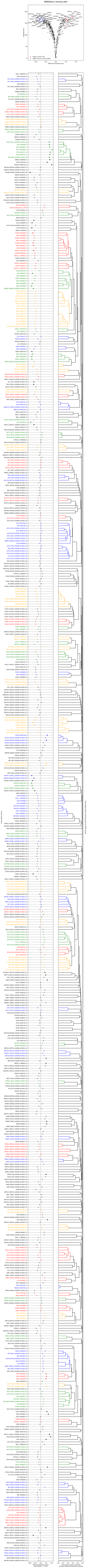

BINDetect volcano plot
-log10(pvalue)
Differential binding score
Higher scores in 8C
Higher scores in naive_hESC
SIX6_1 (PB0059.1)
SIX4 (PH0164.1)
SIX2 (SIX2_HUMAN.H11MO.0.A)
SIX1 (SIX1_HUMAN.H11MO.0.A)
HDX (PH0037.1)
GFI1 (GFI1_HUMAN.H11MO.0.C)
NFIL3 (MA0025.1)
GMEB2 (MA0862.1)
TEF (MA0843.1)
DBP (DBP_HUMAN.H11MO.0.B)
HLF (HLF_HUMAN.H11MO.0.C)
GMEB1 (MA0615.1)
CREB5 (MA0840.1)
ATF7 (MA0834.1)
ATF2 (ATF2_HUMAN.H11MO.0.B)
CREB1 (CREB1_HUMAN.H11MO.0.A)
ATF1 (ATF1_HUMAN.H11MO.0.B)
CREM (CREM_HUMAN.H11MO.0.C)
CEBPB (CEBPB_HUMAN.H11MO.0.A)
CEBPA (CEBPA_HUMAN.H11MO.0.A)
CEBPD (CEBPD_HUMAN.H11MO.0.C)
CEBPE (CEBPE_HUMAN.H11MO.0.A)
CEBPG (CEBPG_HUMAN.H11MO.0.B)
ATF4 (ATF4_HUMAN.H11MO.0.A)
MYBL2 (MA0777.1)
BARHL2 (MA0635.1)
HOMEZ (PH0044.1)
OTX2 (OTX2_HUMAN.H11MO.0.A)
GSC (MA0648.1)

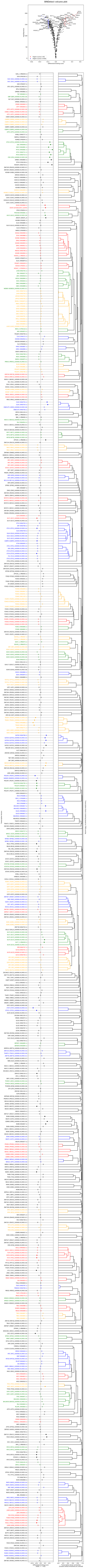

BINDetect volcano plot
-log10(pvalue)
Differential binding score
Higher scores in 8C
Higher scores in hESC
PITX1
RHOXF1
OTX2
CRX
GSC
DMBX1
PAX5
ZNF121
GSC2
PITX2
PITX3
KLF7_1
KLF4
MEF2A
RARB
ESRRG
RXRB
GATA6
MEF2D
OTX1
ALX1
DUX4
ARGFX
GATA5
GATA2
CUX2
MITF
CUX1
ZFP187_1
DUXA
OLIG1
BHLHE23
CTCF
TEAD2
ZIC3
POU5F1
ETV4
TEAD1
CTCFL
TEAD3
TEAD4
TCF3
ZNF528
ZNF436
POU5F1B
POU3F1
ELF1
POU2F2
INSM1
FOSL2
FOSL1
GABPA
RELA
RFX3
RFX4
MECP2
RFXDC2_1
SIX6_1 (PB0059.1)
SIX4 (PH0164.1)
SIX2 (SIX2_HUMAN.H11MO.0.A)
SIX1 (SIX1_HUMAN.H11MO.0.A)
HDX (PH0037.1)
GFI1 (GFI1_HUMAN.H11MO.0.C)
NFIL3 (MA0025.1)
GMEB2 (MA0862.1)
TEF (MA0843.1)
DBP (DBP_HUMAN.H11MO.0.B)
HLF (HLF_HUMAN.H11MO.0.C)
GMEB1 (MA0615.1)
CREB5 (MA0840.1)
ATF7 (MA0834.1)
ATF2 (ATF2_HUMAN.H11MO.0.B)
CREB1 (CREB1_HUMAN.H11MO.0.A)
ATF1 (ATF1_HUMAN.H11MO.0.B)
CREM (CREM_HUMAN.H11MO.0.C)
CEBPB (CEBPB_HUMAN.H11MO.0.A)
CEBPA (CEBPA_HUMAN.H11MO.0.A)
CEBPD (CEBPD_HUMAN.H11MO.0.C)
CEBPE (CEBPE_HUMAN.H11MO.0.A)
CEBPG (CEBPG_HUMAN.H11MO.0.B)
ATF4 (ATF4_HUMAN.H11MO.0.A)
MYBL2 (MA0777.1)
BARHL2 (MA0635.1)
HOMEZ (PH0044.1)
OTX2 (OTX2_HUMAN.H11MO.0.A)
GSC (MA0648.1)
GSC2 (MA0891.1)
PITX1 (MA0682.1)
OTX1 (MA0711.1)
PITX3 (MA0714.1)
CRX (CRX_HUMAN.H11MO.0.B)
DMBX1 (MA0883.1)
PITX2 (PH0138.1)
RHOXF1 (MA0719.1)
ZNF329 (ZN329_HUMAN.H11MO.0.C)

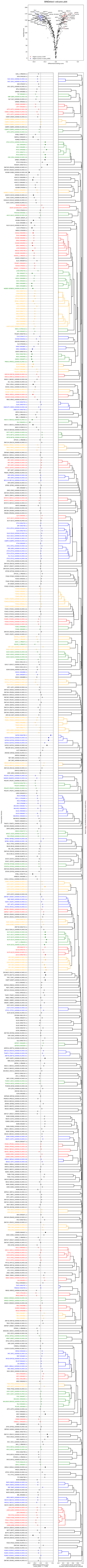
BINDetect volcano plot
-log10(pvalue)
Differential binding score
Higher scores in ICM
Higher scores in naive_hESC
SIX6_1 (PB0059.1)
SIX4 (PH0164.1)
SIX2 (SIX2_HUMAN.H11MO.0.A)
SIX1 (SIX1_HUMAN.H11MO.0.A)
HDX (PH0037.1)
GFI1 (GFI1_HUMAN.H11MO.0.C)
NFIL3 (MA0025.1)
GMEB2 (MA0862.1)
TEF (MA0843.1)
DBP (DBP_HUMAN.H11MO.0.B)
HLF (HLF_HUMAN.H11MO.0.C)
GMEB1 (MA0615.1)
CREB5 (MA0840.1)
ATF7 (MA0834.1)
ATF2 (ATF2_HUMAN.H11MO.0.B)
CREB1 (CREB1_HUMAN.H11MO.0.A)
ATF1 (ATF1_HUMAN.H11MO.0.B)
CREM (CREM_HUMAN.H11MO.0.C)
CEBPB (CEBPB_HUMAN.H11MO.0.A)
CEBPA (CEBPA_HUMAN.H11MO.0.A)
CEBPD (CEBPD_HUMAN.H11MO.0.C)
CEBPE (CEBPE_HUMAN.H11MO.0.A)
CEBPG (CEBPG_HUMAN.H11MO.0.B)
ATF4 (ATF4_HUMAN.H11MO.0.A)
MYBL2 (MA0777.1)
BARHL2 (MA0635.1)
HOMEZ (PH0044.1)
OTX2 (OTX2_HUMAN.H11MO.0.A)
GSC (MA0648.1)
GSC2 (MA0891.1)
PITX1 (MA0682.1)

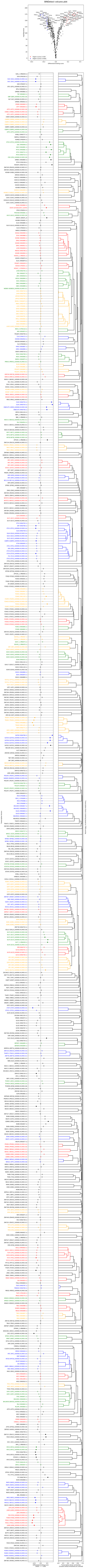
BINDetect volcano plot

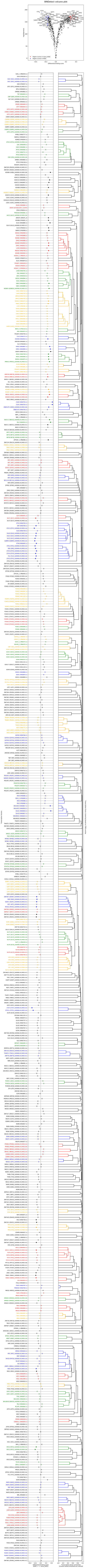
BINDetect volcano plot
-log10(pvalue)
Differential binding score
Higher scores in naive_hESC
Higher scores in hESC
