# Supplementary material for: ATAC-seq footprinting unravels kinetics of transcription factor binding during zygotic genome activation
Source: Nat Commun. 2020 Aug 26;11:4267. doi: 10.1038/s41467-020-18035-1 (PMC7449963; doi:10.1038/s41467-020-18035-1)

Aggregate footprints in A549 for TF CEBPB (ChIP-seq ENCFF047UIF)

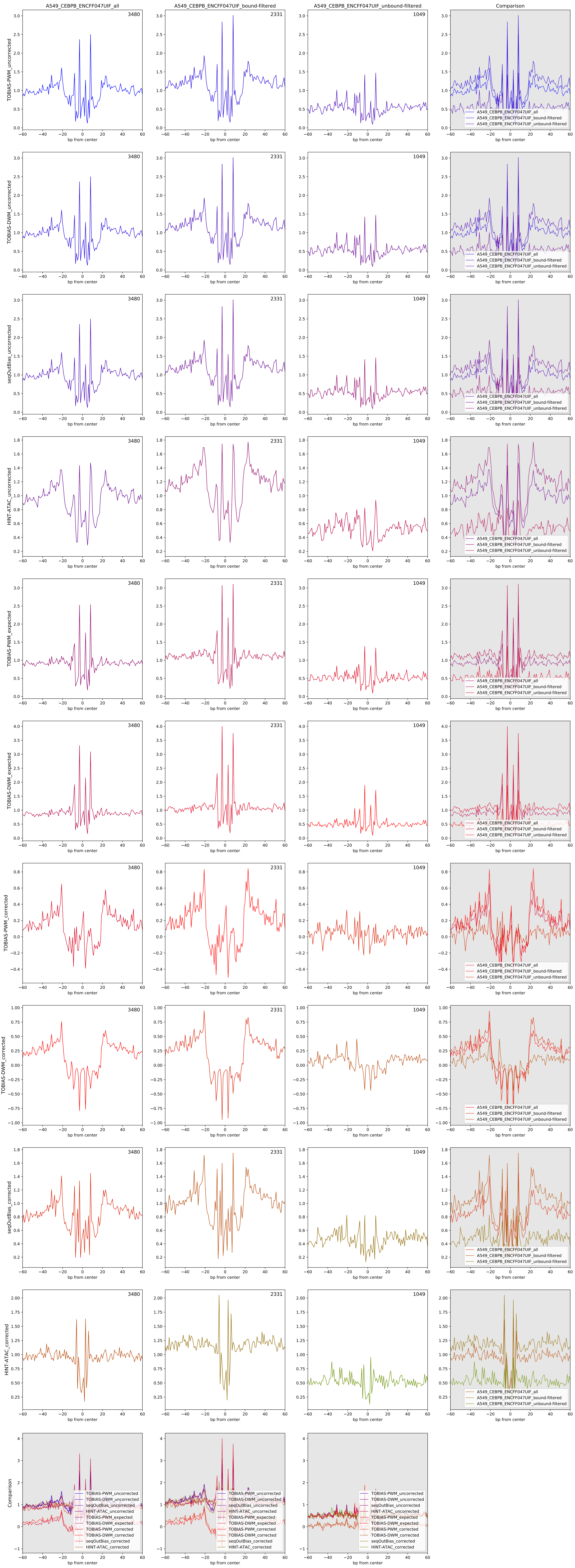

Aggregate footprints in A549 for TF CTCF (ChIP-seq ENCFF646TUX)

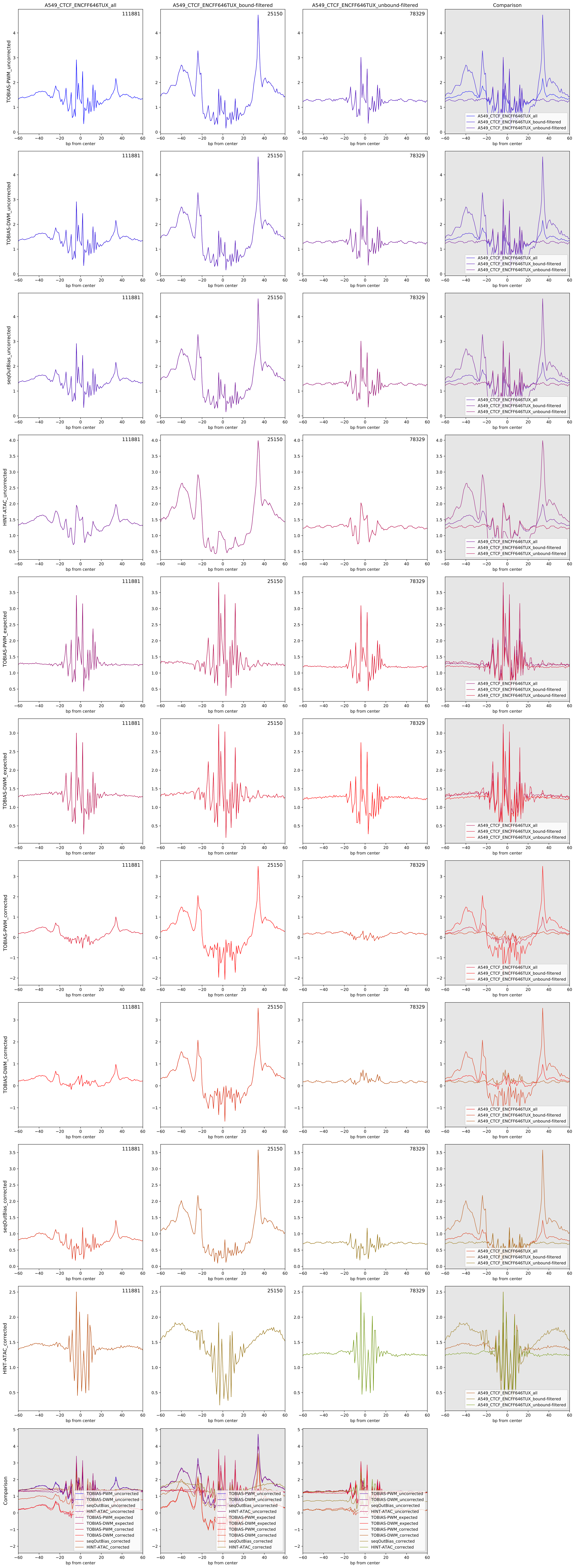

Aggregate footprints in A549 for TF ELK1 (ChIP-seq ENCFF605JXG)

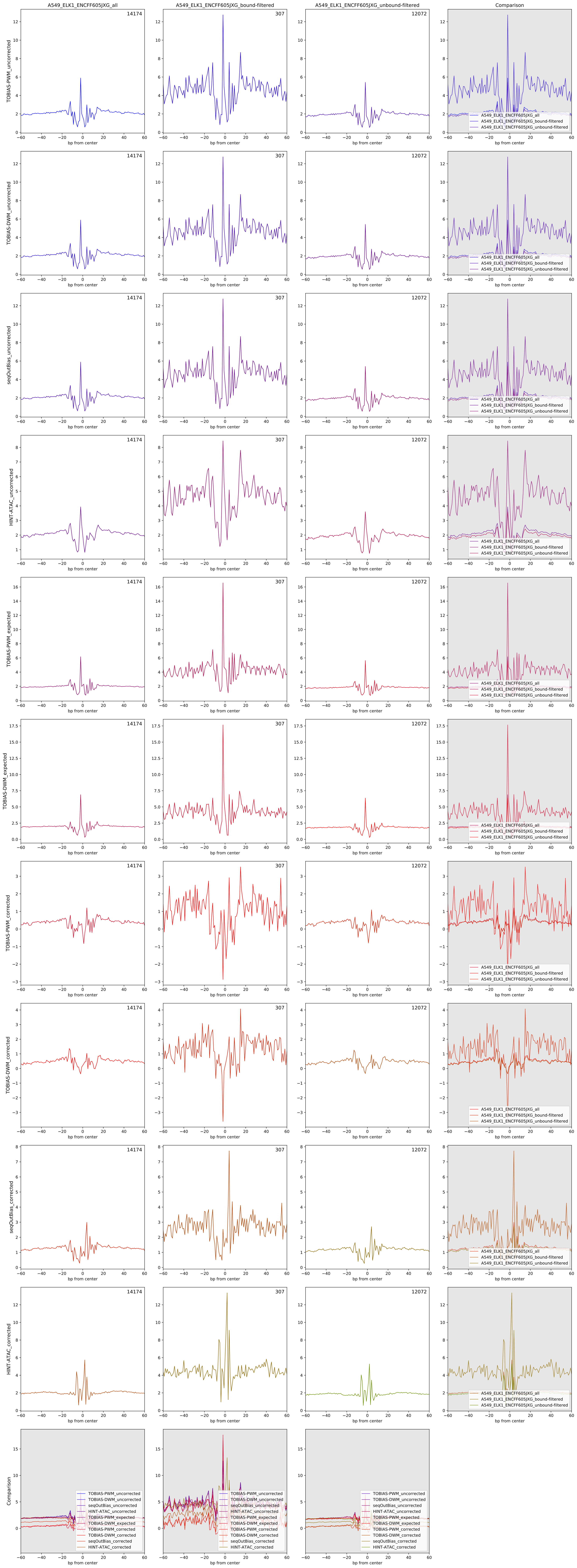

Aggregate footprints in A549 for TF ESRRA (ChIP-seq ENCFF558UWY)

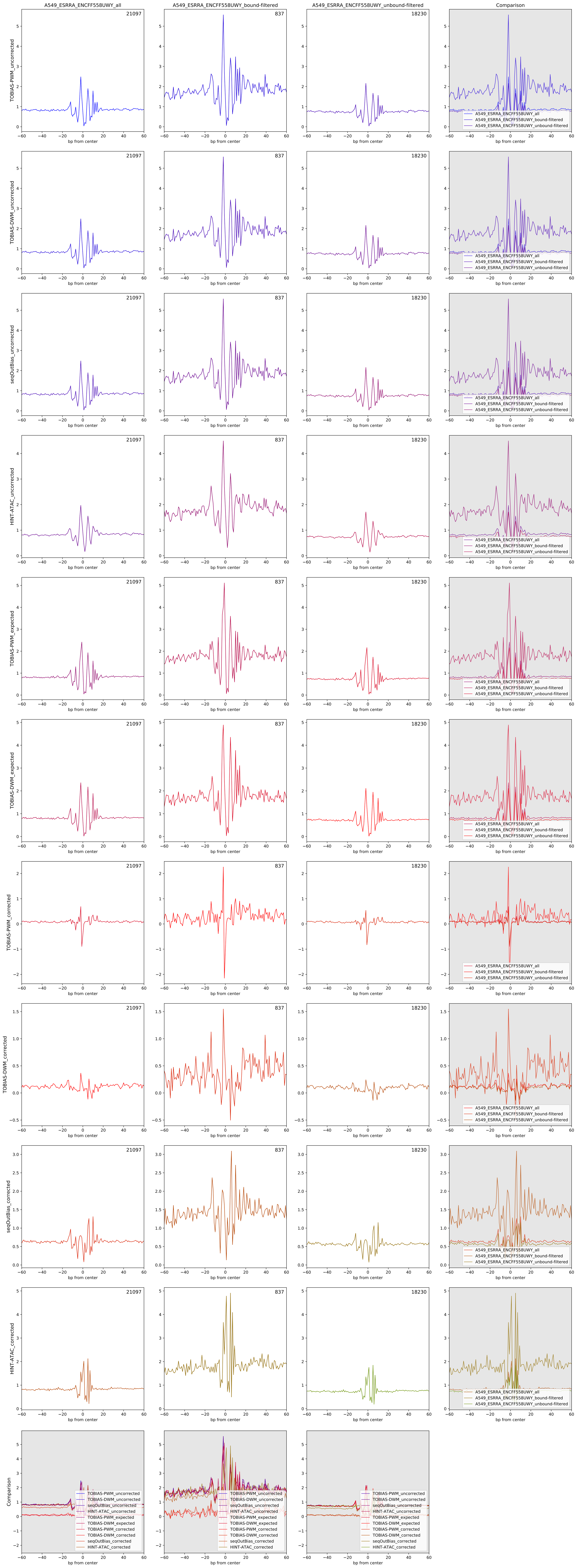

Aggregate footprints in A549 for TF JUN (ChIP-seq ENCFF127HJG)

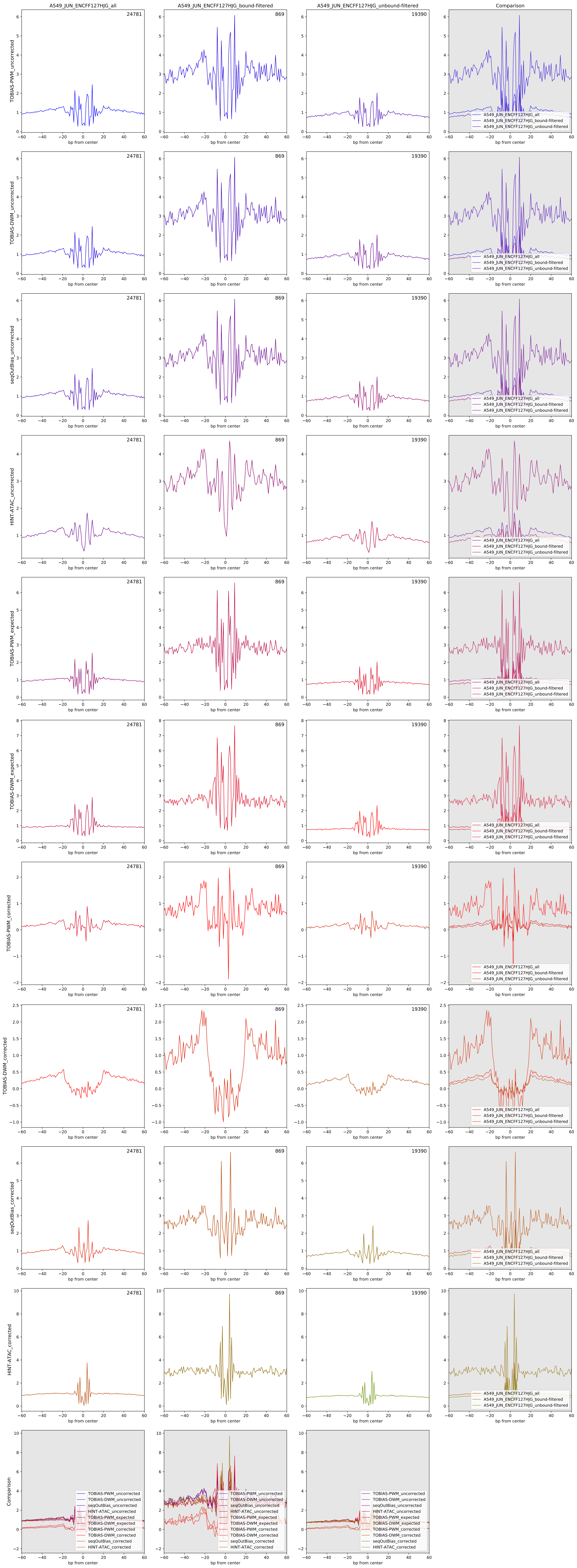

Aggregate footprints in A549 for TF MAFK (ChIP-seq ENCFF813WJW)

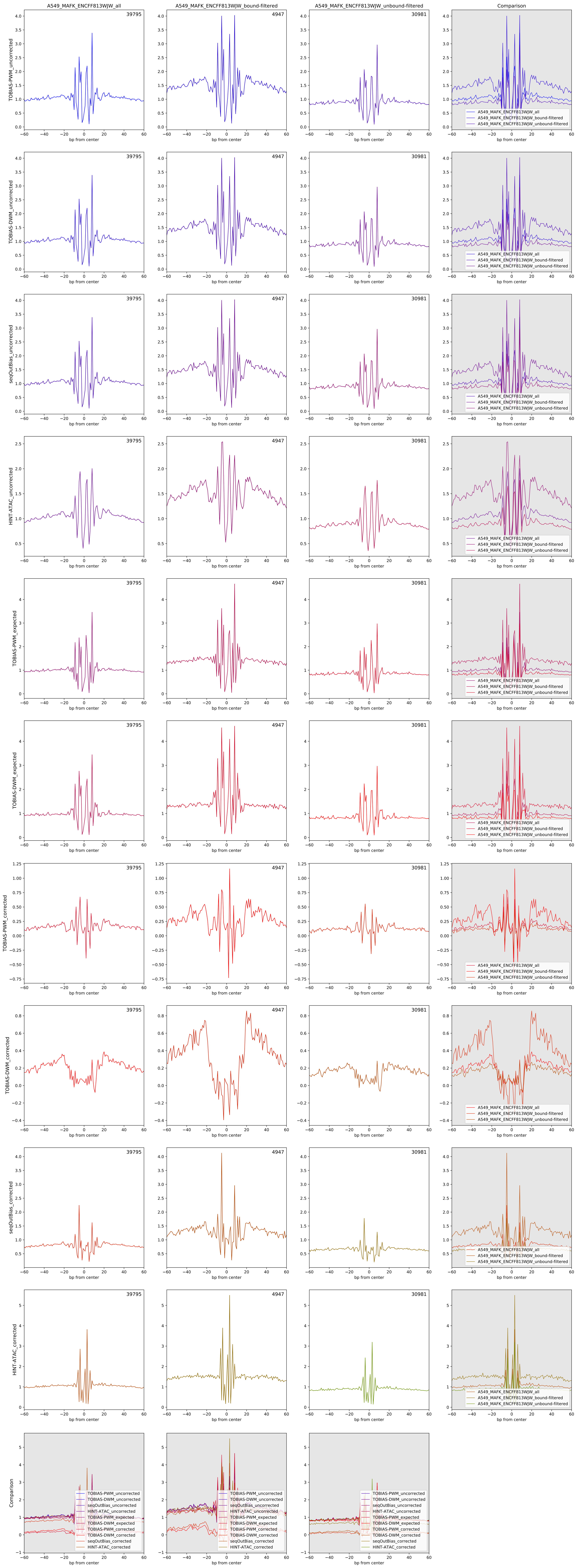

Aggregate footprints in A549 for TF MYC (ChIP-seq ENCFF542GMN)

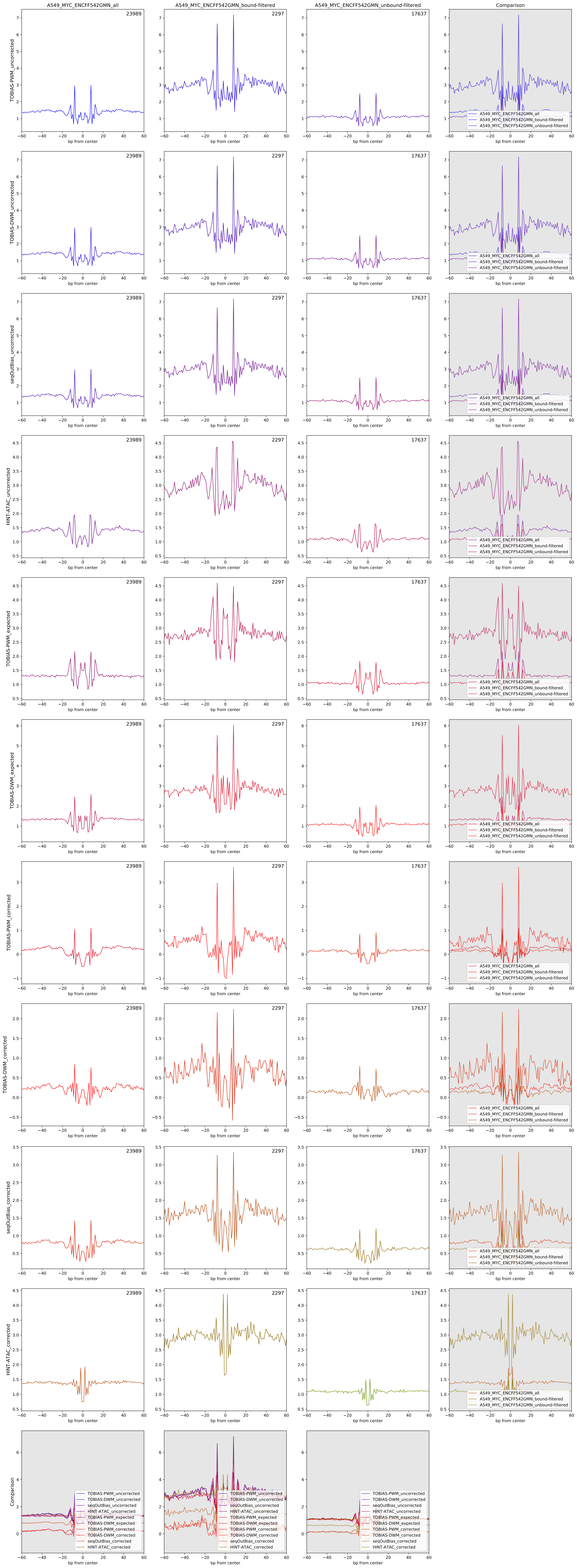

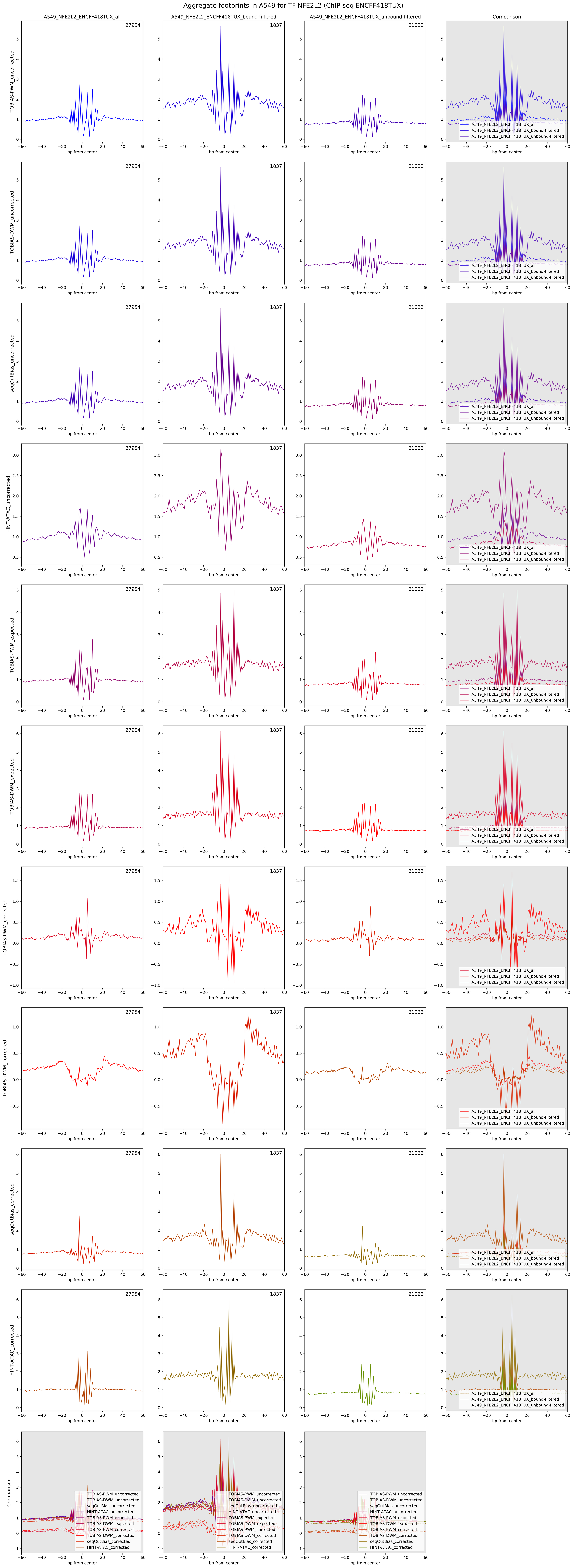
Aggregate footprints in A549 for TF NFE2L2 (ChIP-seq ENCFF418TUX)
A549_NFE2L2_ENCFF418TUX_all
A549_NFE2L2_ENCFF418TUX_bound-filtered
A549_NFE2L2_ENCFF418TUX_unbound-filtered
Comparison
TOBIAS-PWM_uncorrected
TOBIAS-DWM_uncorrected
seqOutBias_uncorrected
HINT-ATAC_uncorrected
TOBIAS-PWM_expected
TOBIAS-DWM_expected
TOBIAS-PWM_corrected
TOBIAS-DWM_corrected
seqOutBias_corrected
HINT-ATAC_corrected
27954
1837
21022
bp from center

Aggregate footprints in A549 for TF REST (ChIP-seq ENCFF706DRE)

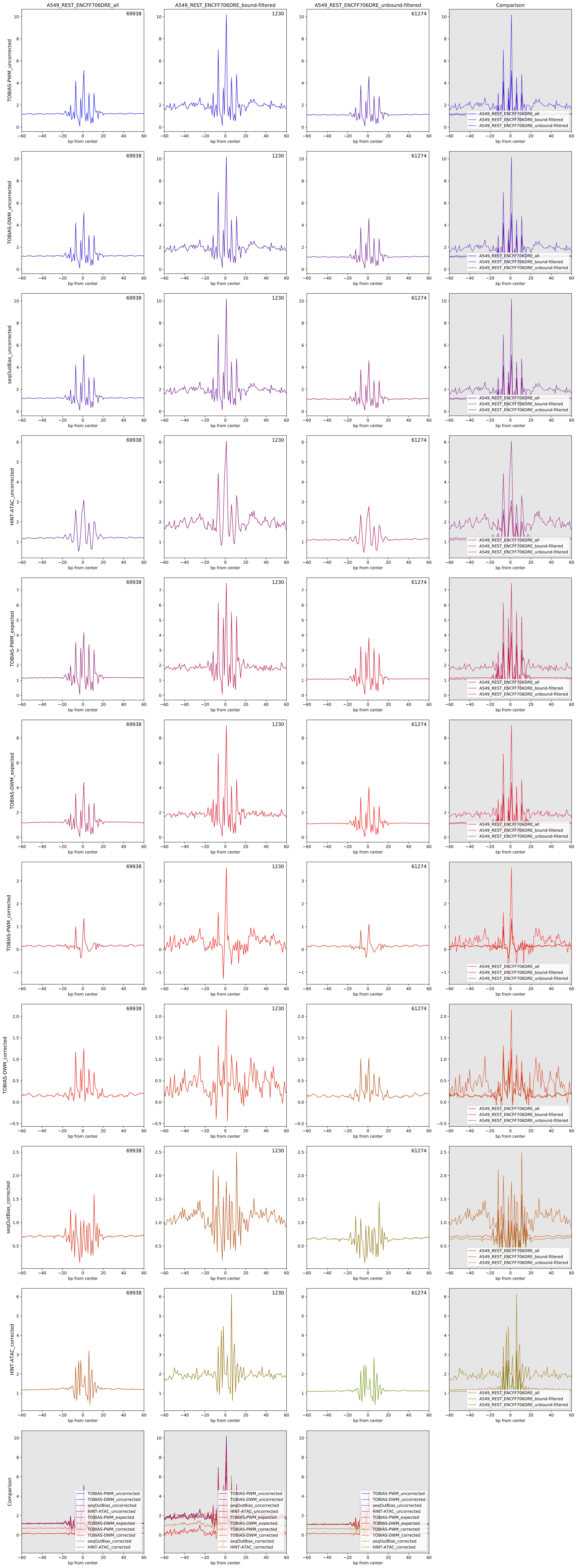

Aggregate footprints in A549 for TF RFX5 (ChIP-seq ENCFF179WDI)

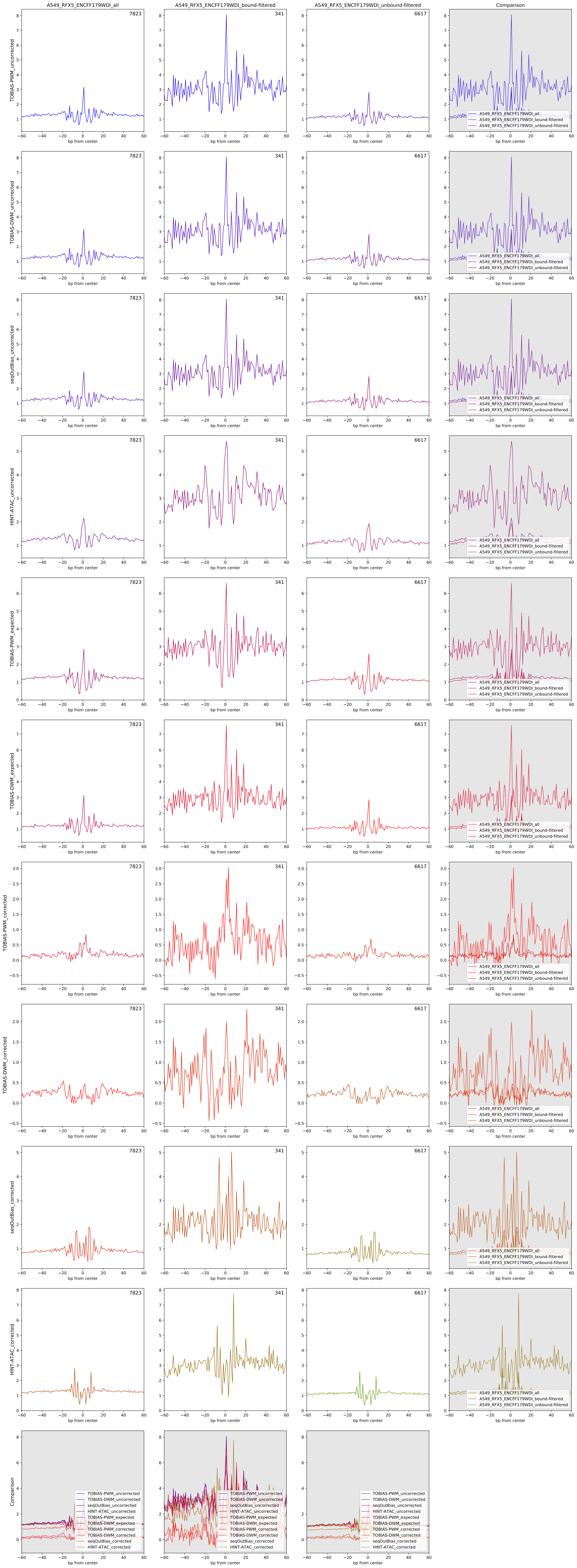

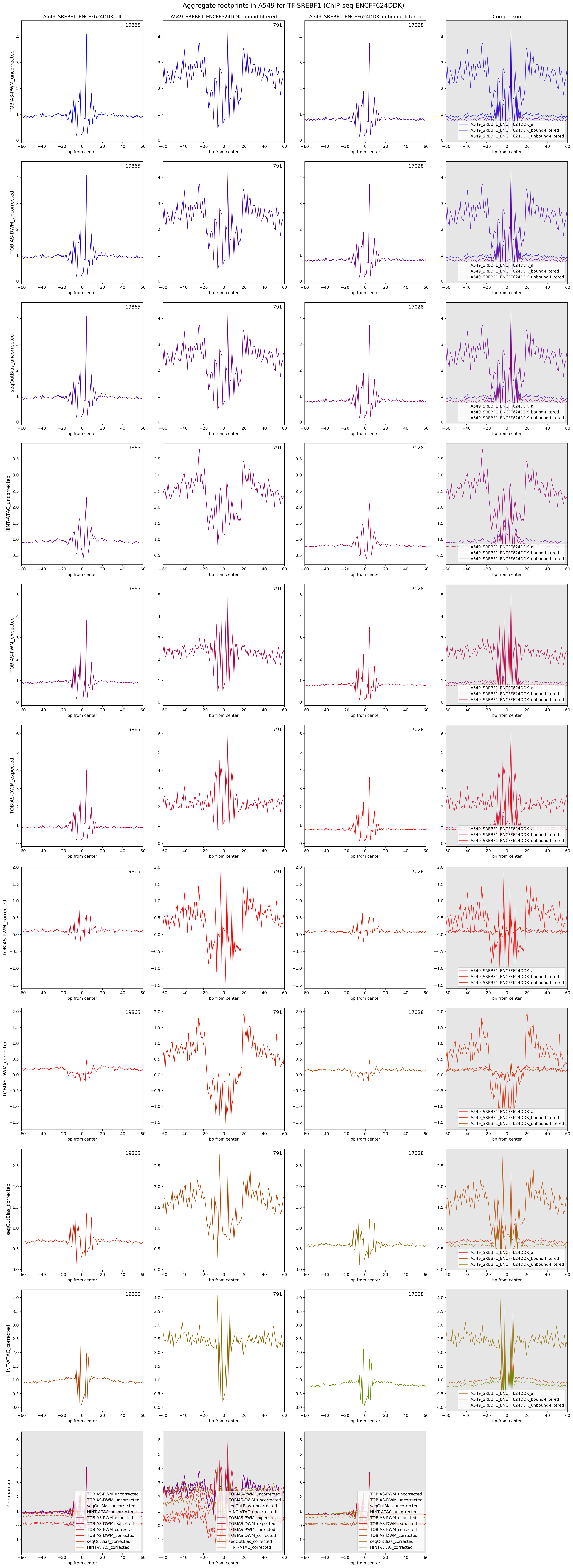
Aggregate footprints in A549 for TF SREBF1 (ChIP-seq ENCFF624DDK)
A549_SREBF1_ENCFF624DDK_all
A549_SREBF1_ENCFF624DDK_bound-filtered
A549_SREBF1_ENCFF624DDK_unbound-filtered
Comparison
TOBIAS-PWM_uncorrected
TOBIAS-DWM_uncorrected
seqOutBias_uncorrected
HINT-ATAC_uncorrected
TOBIAS-PWM_expected
TOBIAS-DWM_expected
TOBIAS-PWM_corrected
TOBIAS-DWM_corrected
seqOutBias_corrected
HINT-ATAC_corrected
19865
791
17028
bp from center

Aggregate footprints in A549 for TF USF2 (ChIP-seq ENCFF593EOW)

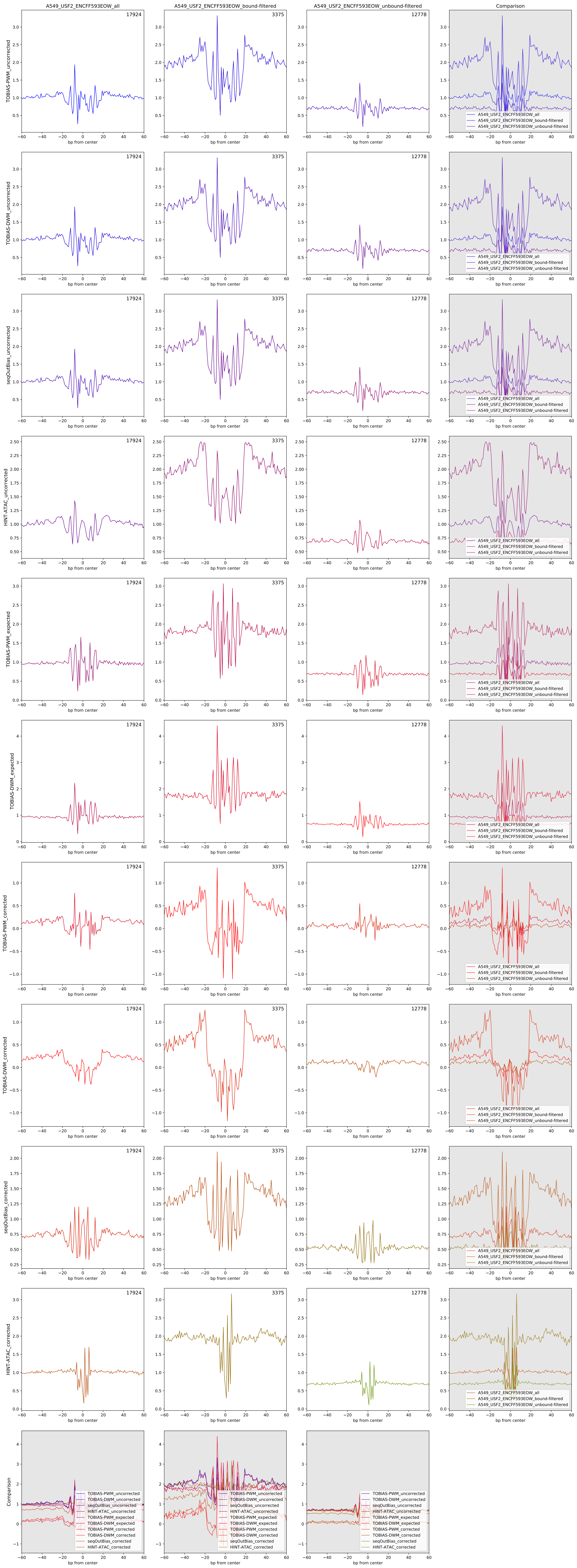

Aggregate footprints in GM12878 for TF ARID3A (ChIP-seq ENCFF003VDB)

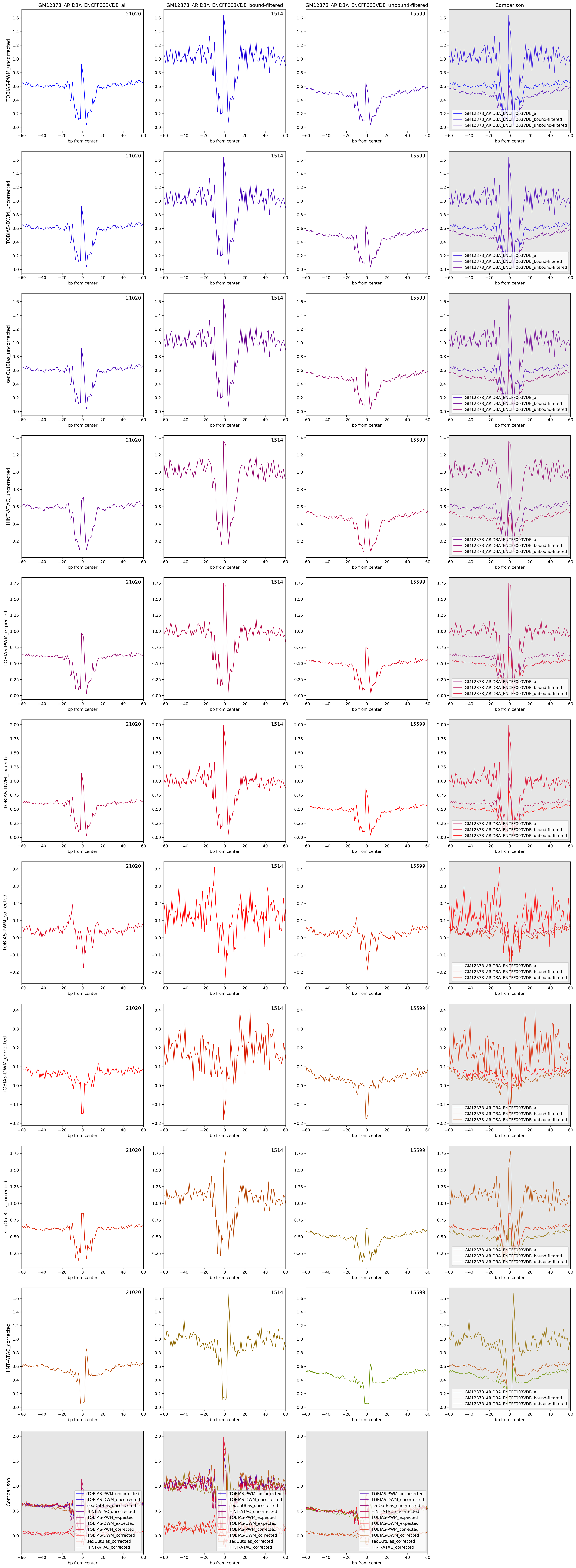

Aggregate footprints in GM12878 for TF ARNT (ChIP-seq ENCFF758RQJ)

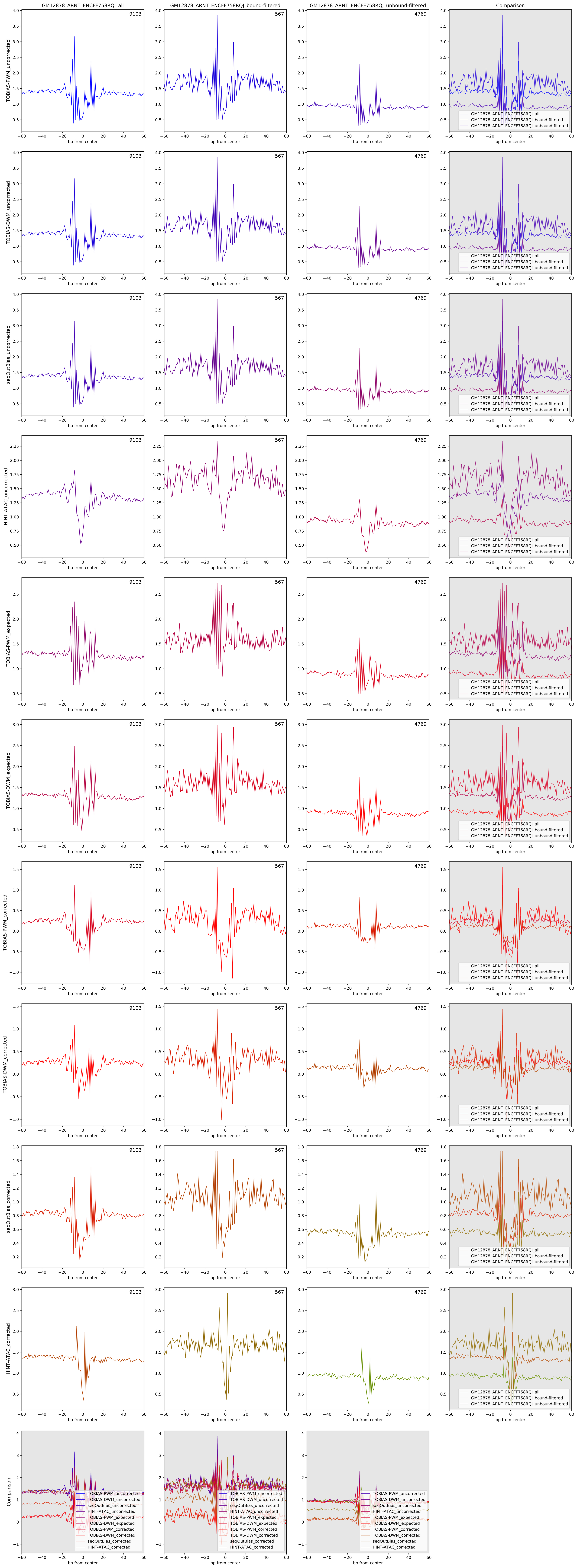

Aggregate footprints in GM12878 for TF BACH1 (ChIP-seq ENCFF725YZH)

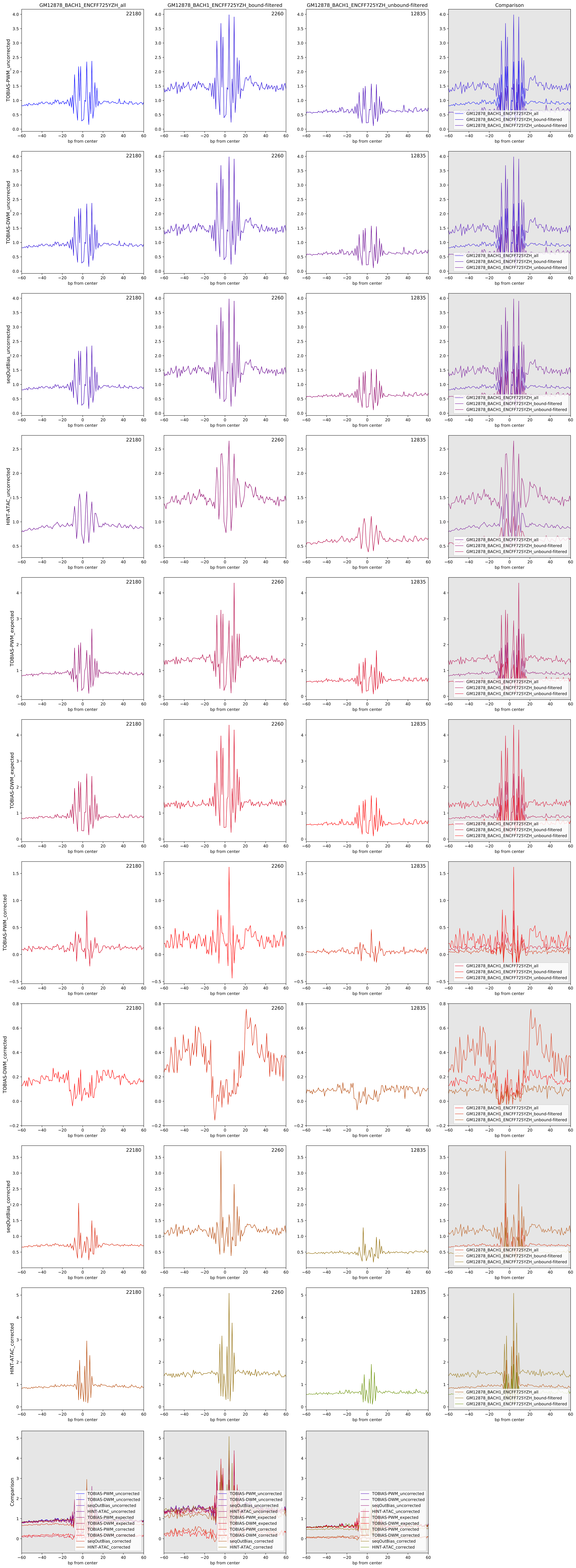

Aggregate footprints in GM12878 for TF BATF (ChIP-seq ENCFF832YIE)

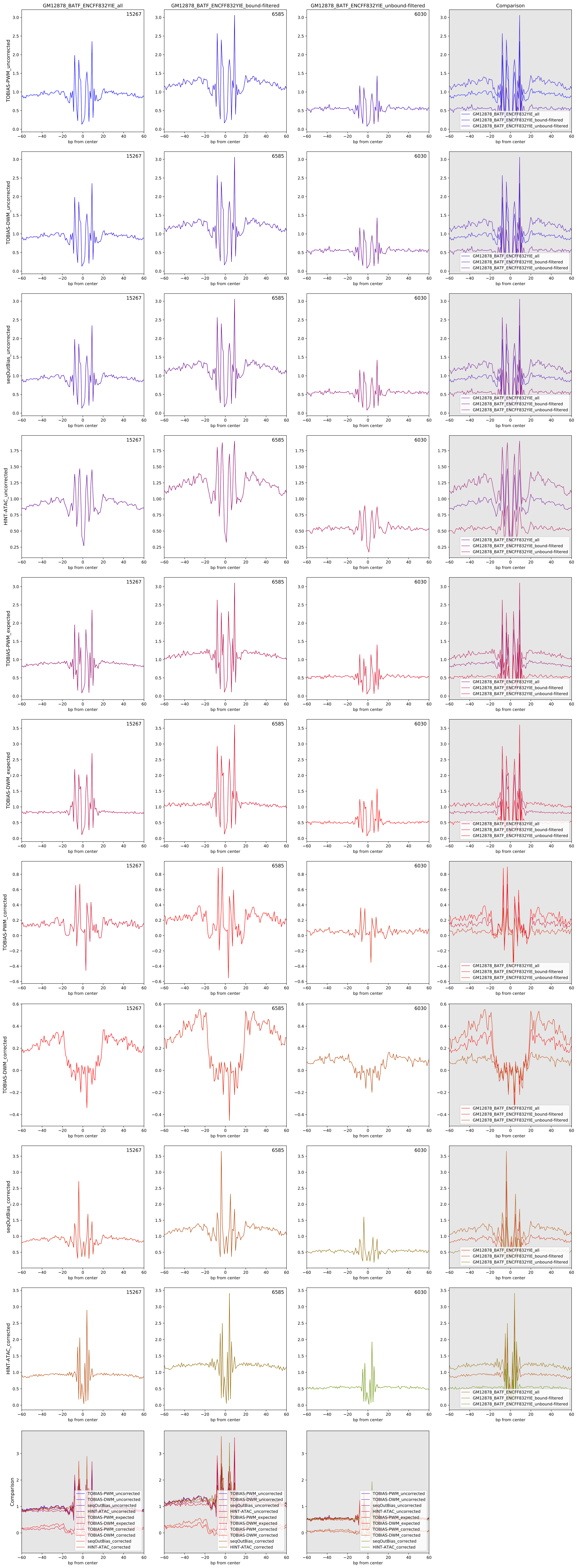

Aggregate footprints in GM12878 for TF BHLHE40 (ChIP-seq ENCFF370ZNL)

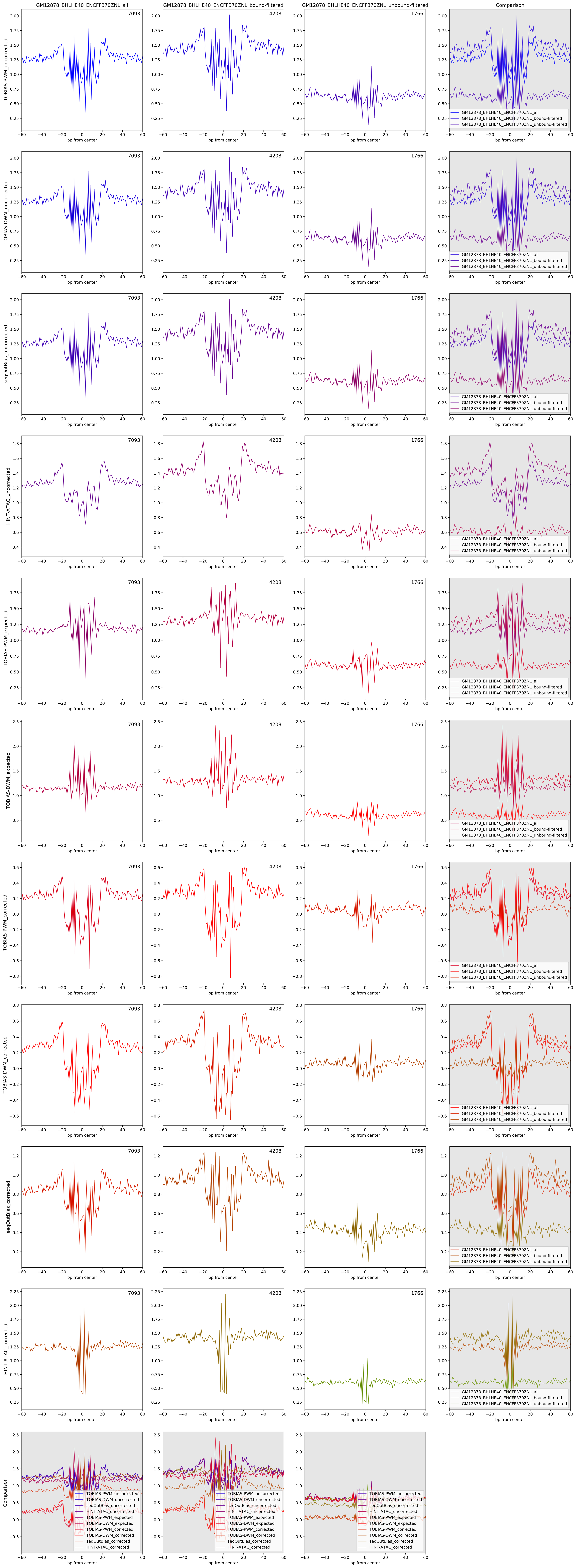

Aggregate footprints in GM12878 for TF CEBPB (ChIP-seq ENCFF786YYI)

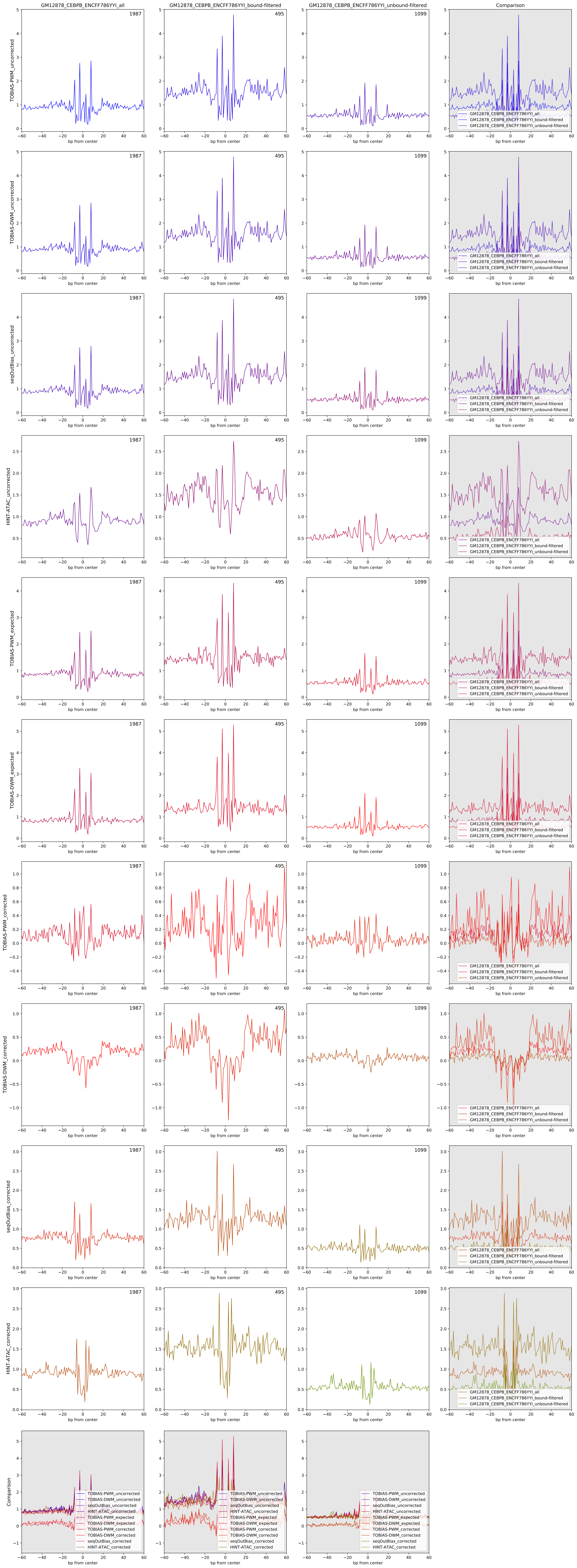

Aggregate footprints in GM12878 for TF CREM (ChIP-seq ENCFF091YID)

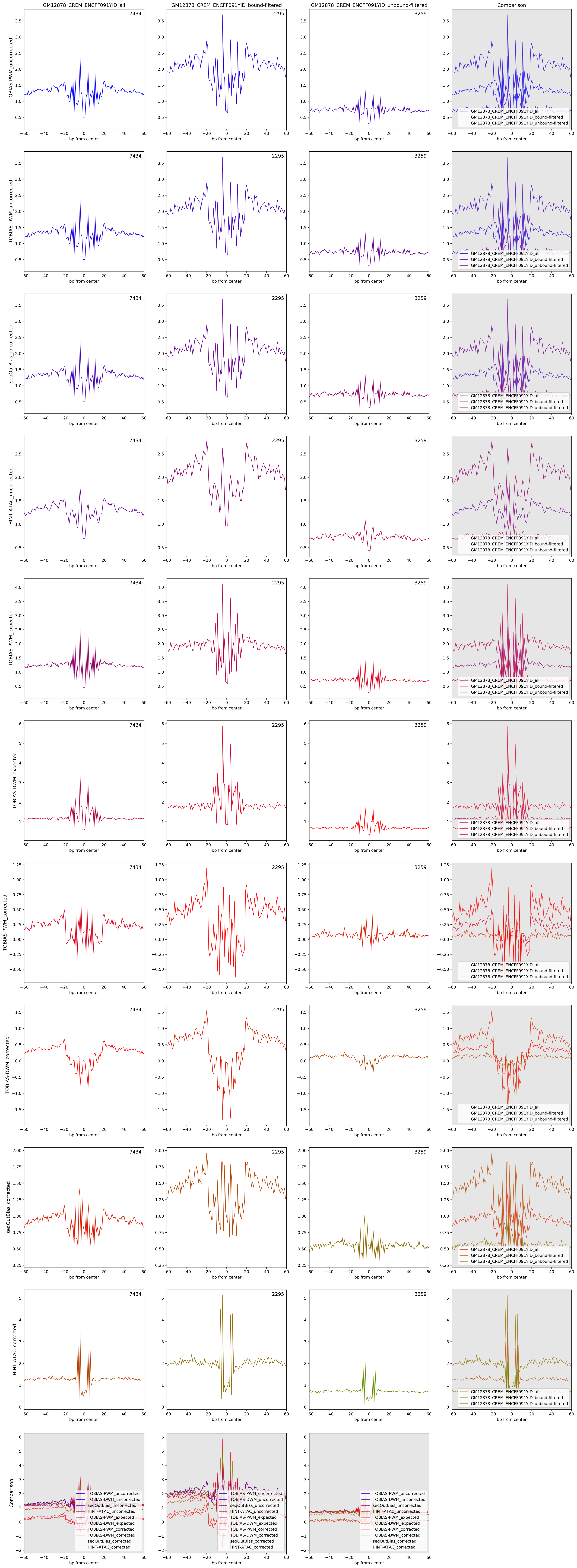

Aggregate footprints in GM12878 for TF CTCF (ChIP-seq ENCFF960ZGP)

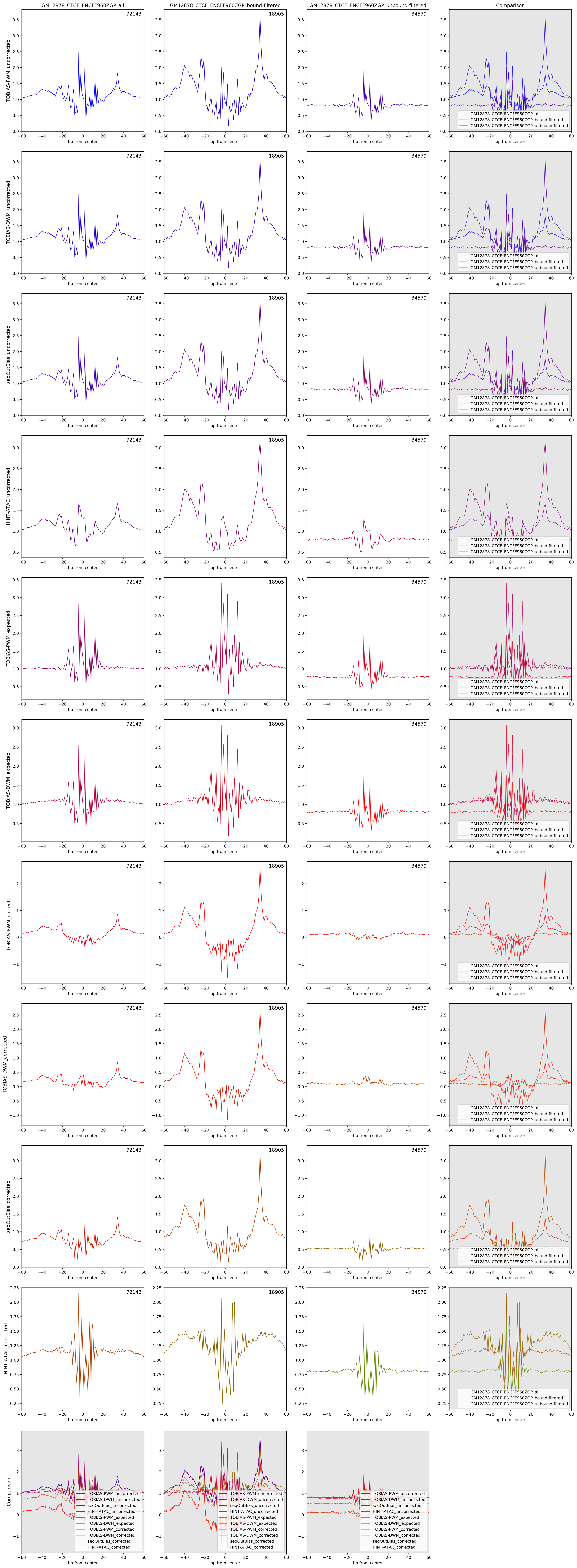

Aggregate footprints in GM12878 for TF CUX1 (ChIP-seq ENCFF567NFS)

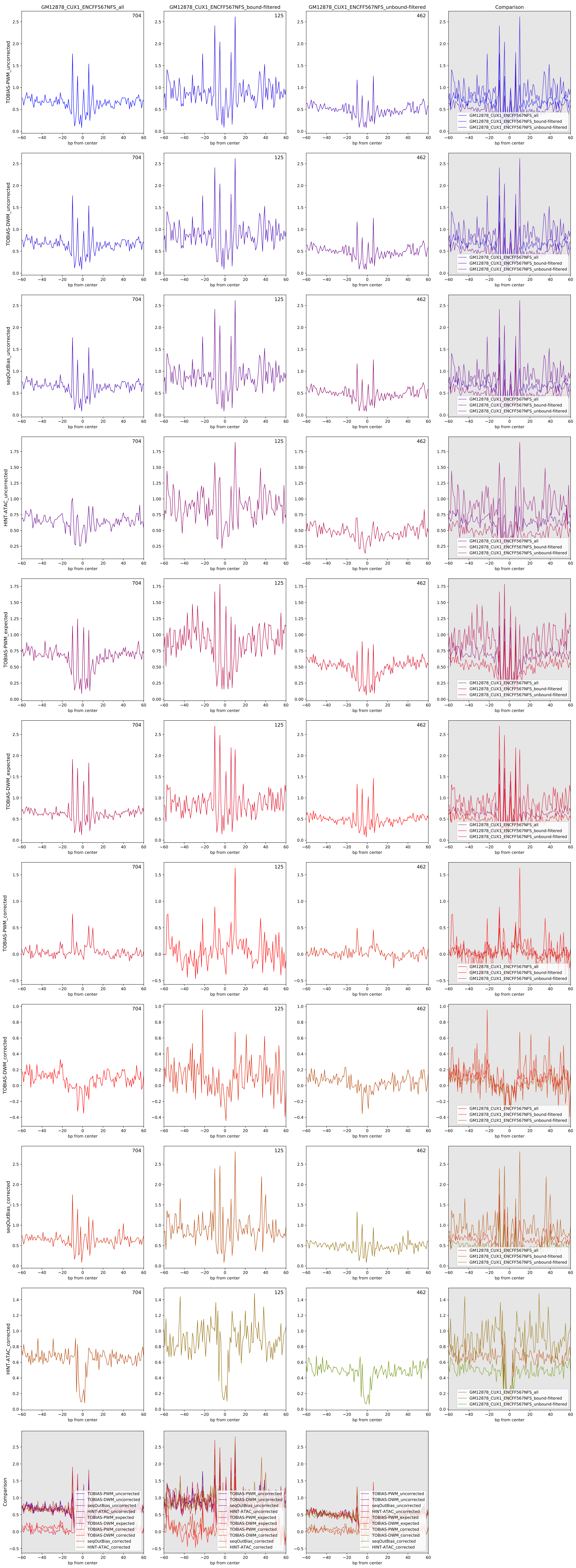

Aggregate footprints in GM12878 for TF E2F4 (ChIP-seq ENCFF687SFB)

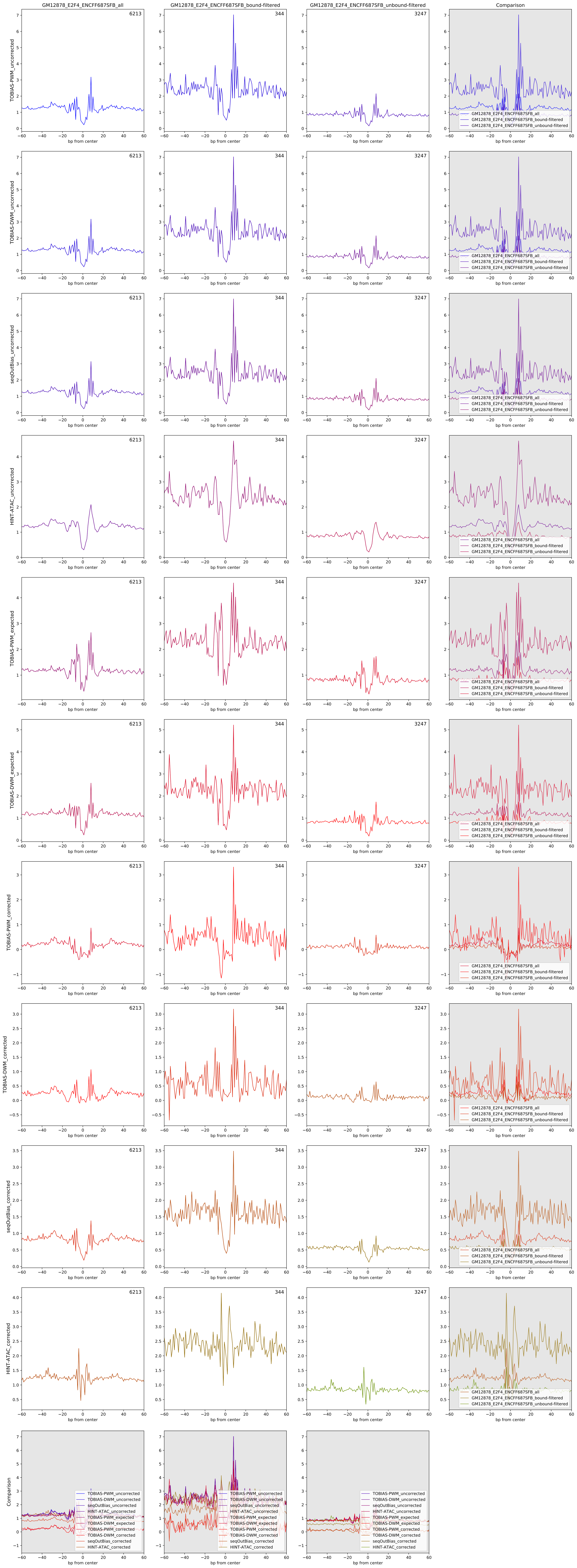

Aggregate footprints in GM12878 for TF EBF1 (ChIP-seq ENCFF249SVT)

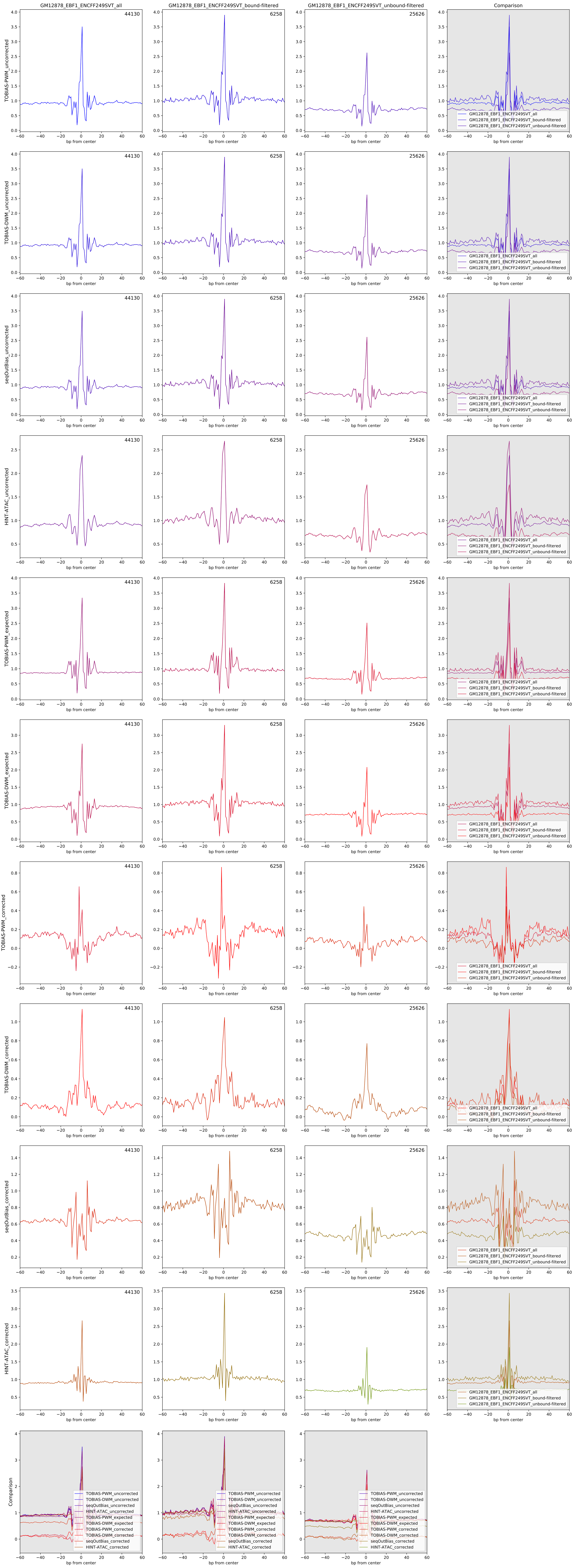

Aggregate footprints in GM12878 for TF ELF1 (ChIP-seq ENCFF948CPI)

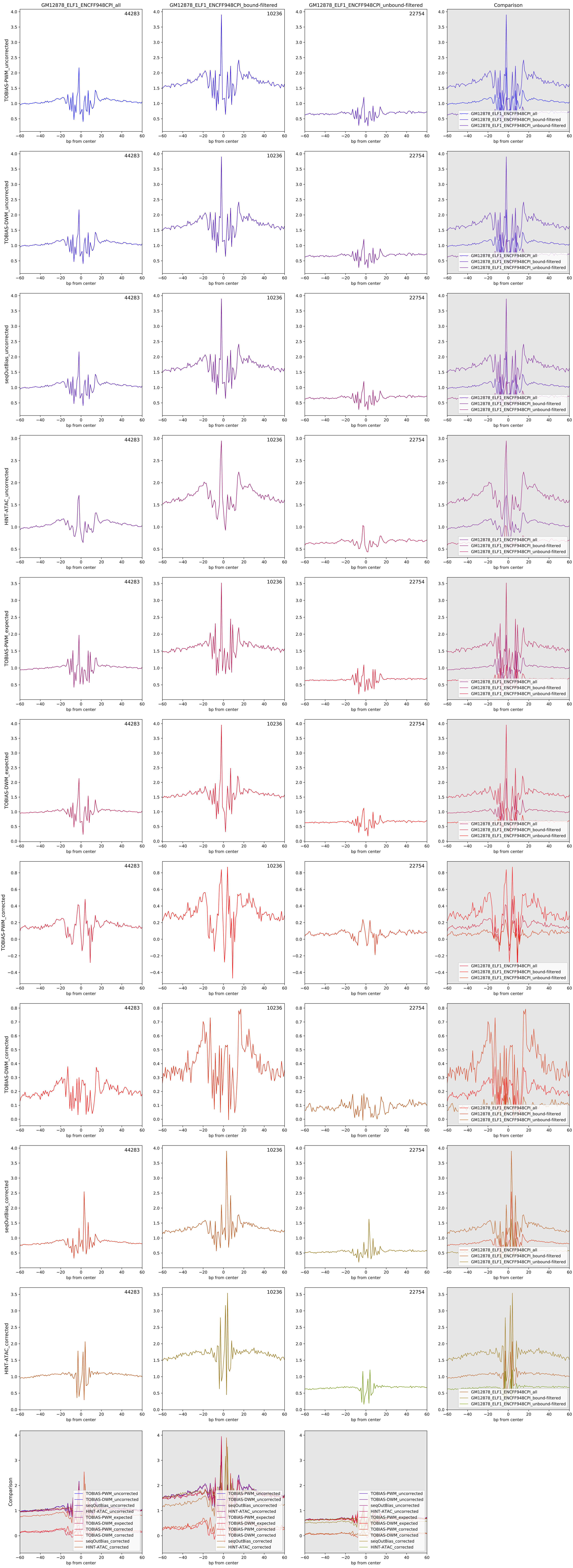

Aggregate footprints in GM12878 for TF ELK1 (ChIP-seq ENCFF432AQP)

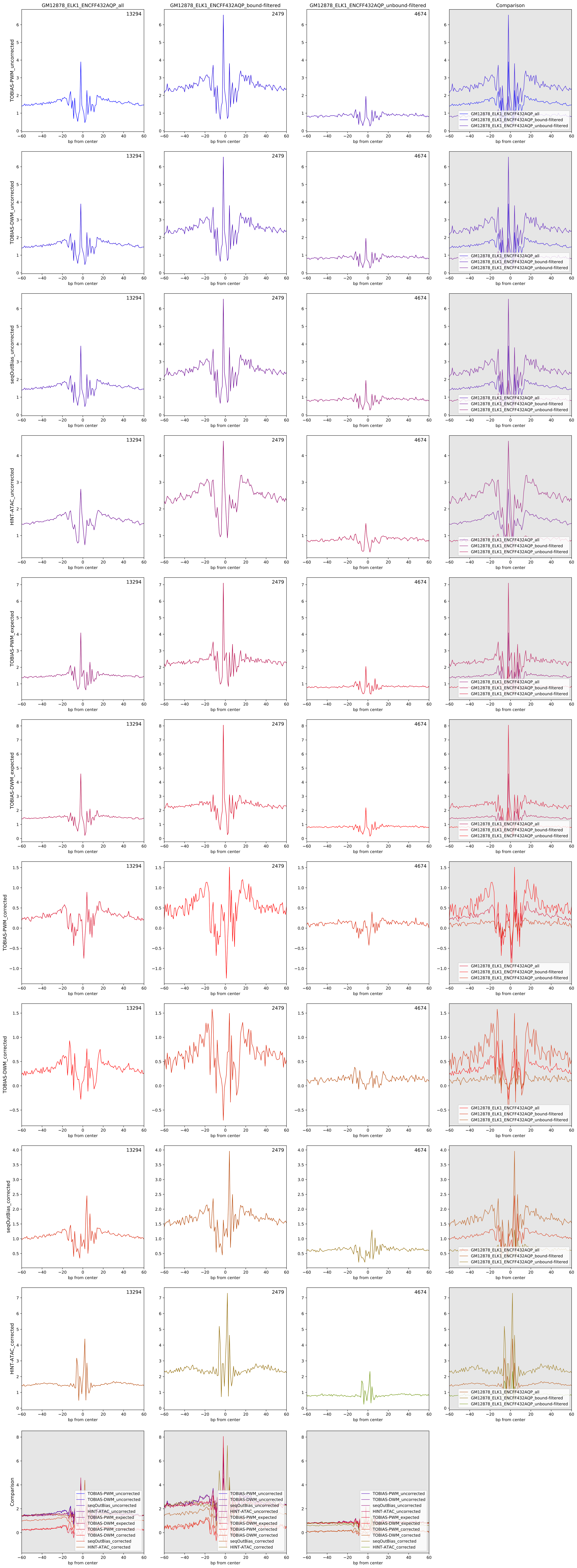

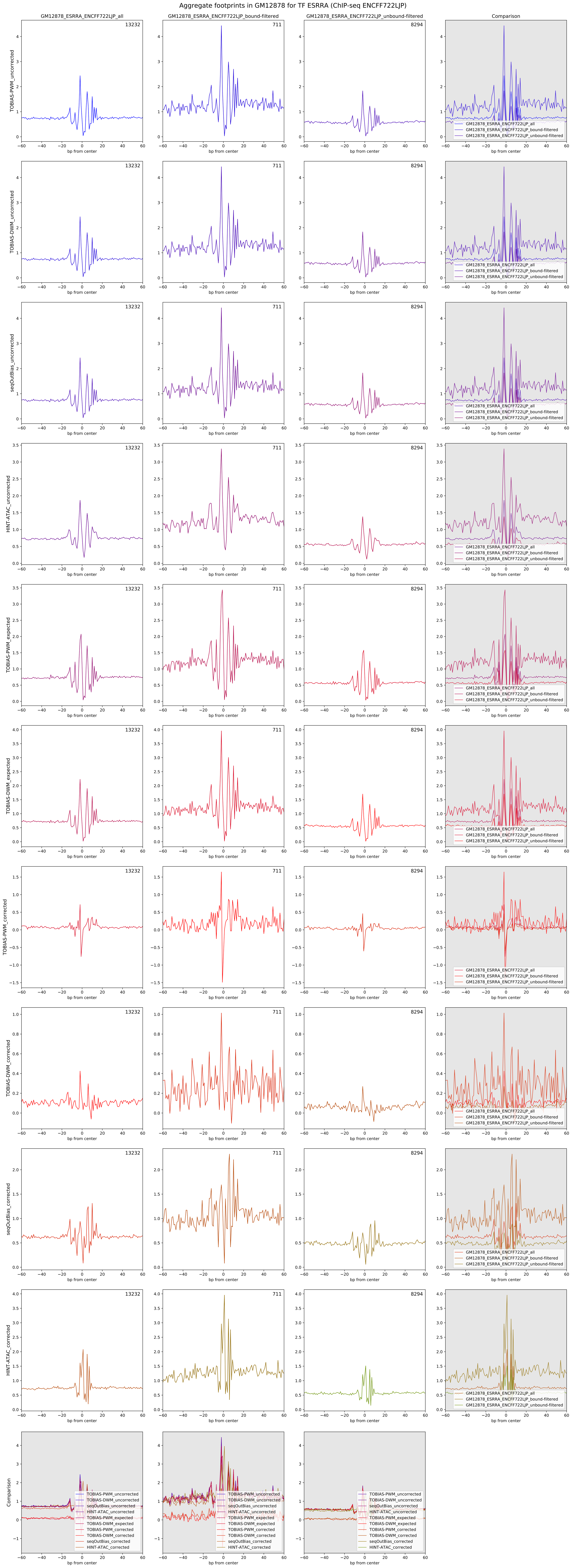

Aggregate footprints in GM12878 for TF ESRRA (ChIP-seq ENCFF722LJP)
GM12878_ESRRA_ENCFF722LJP_all
GM12878_ESRRA_ENCFF722LJP_bound-filtered
GM12878_ESRRA_ENCFF722LJP_unbound-filtered
Comparison
13232
711
8294
TOBIAS-PWM_uncorrected
TOBIAS-DWM_uncorrected
seqOutBias_uncorrected
HINT-ATAC_uncorrected
TOBIAS-PWM_expected
TOBIAS-DWM_expected
TOBIAS-PWM_corrected
TOBIAS-DWM_corrected
seqOutBias_corrected
HINT-ATAC_corrected
bp from center

Aggregate footprints in GM12878 for TF ETS1 (ChIP-seq ENCFF980VOD)

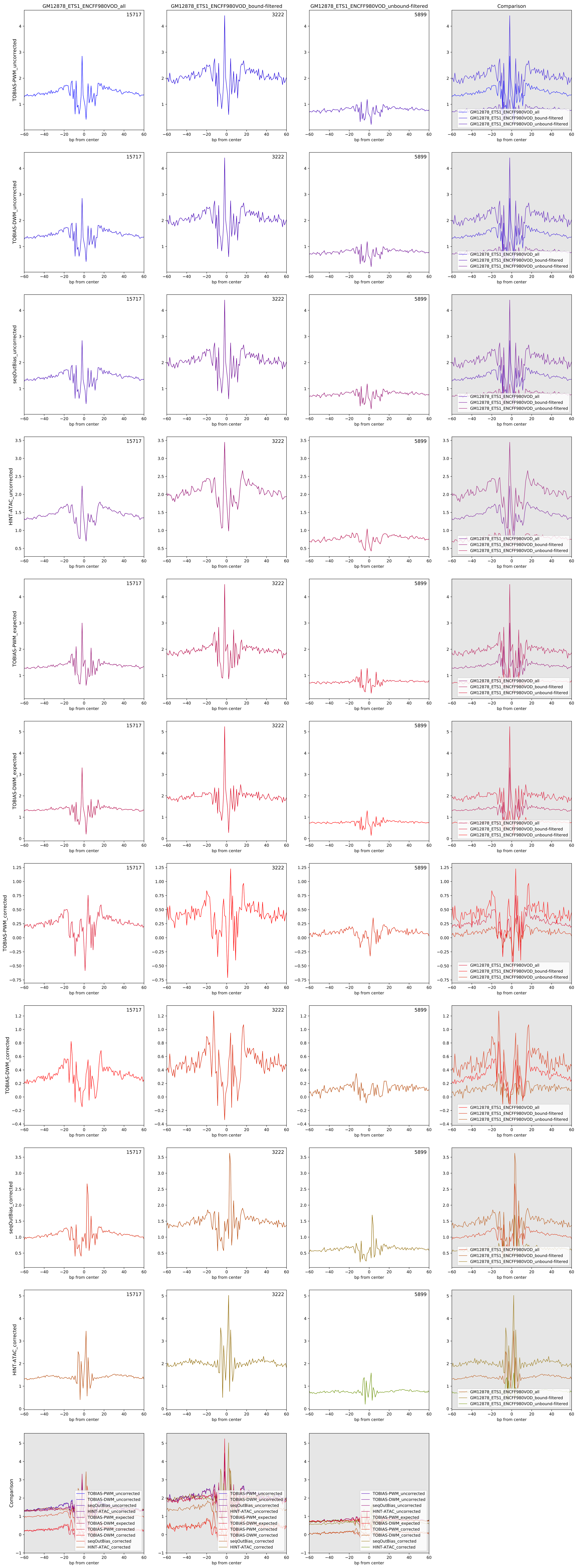

Aggregate footprints in GM12878 for TF ETV6 (ChIP-seq ENCFF745ANU)

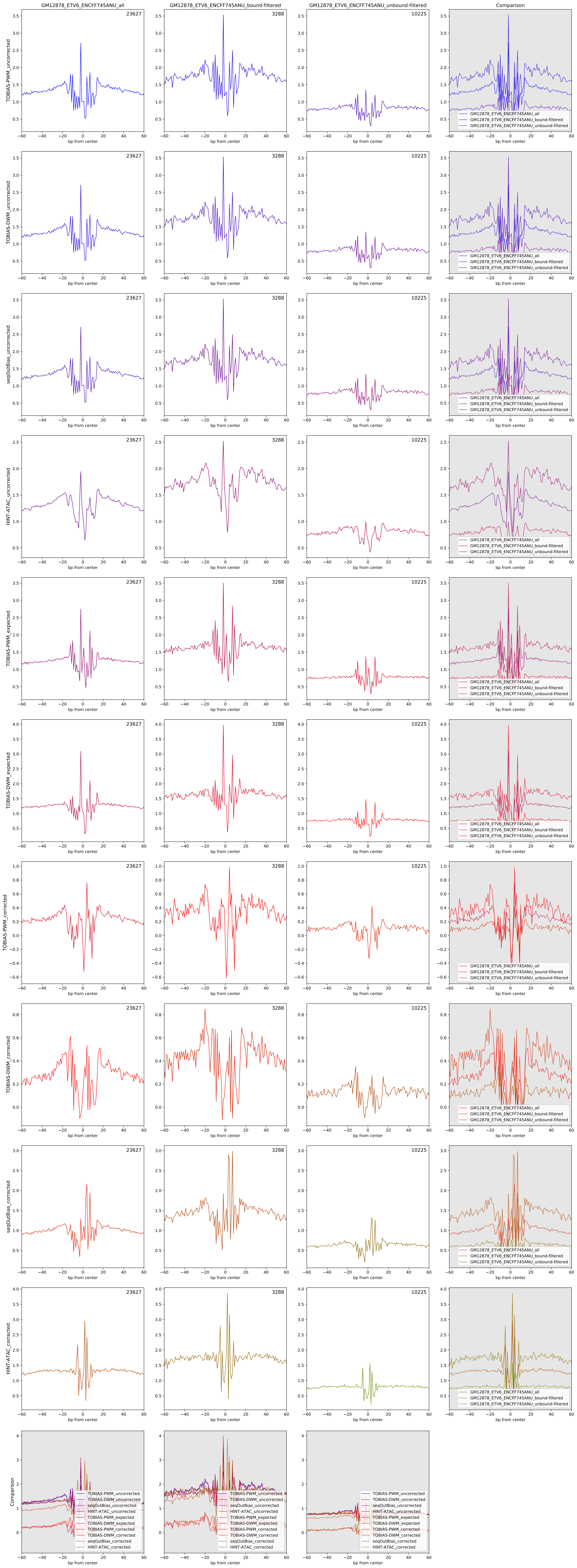

Aggregate footprints in GM12878 for TF FOXK2 (ChIP-seq ENCFF990MTR)

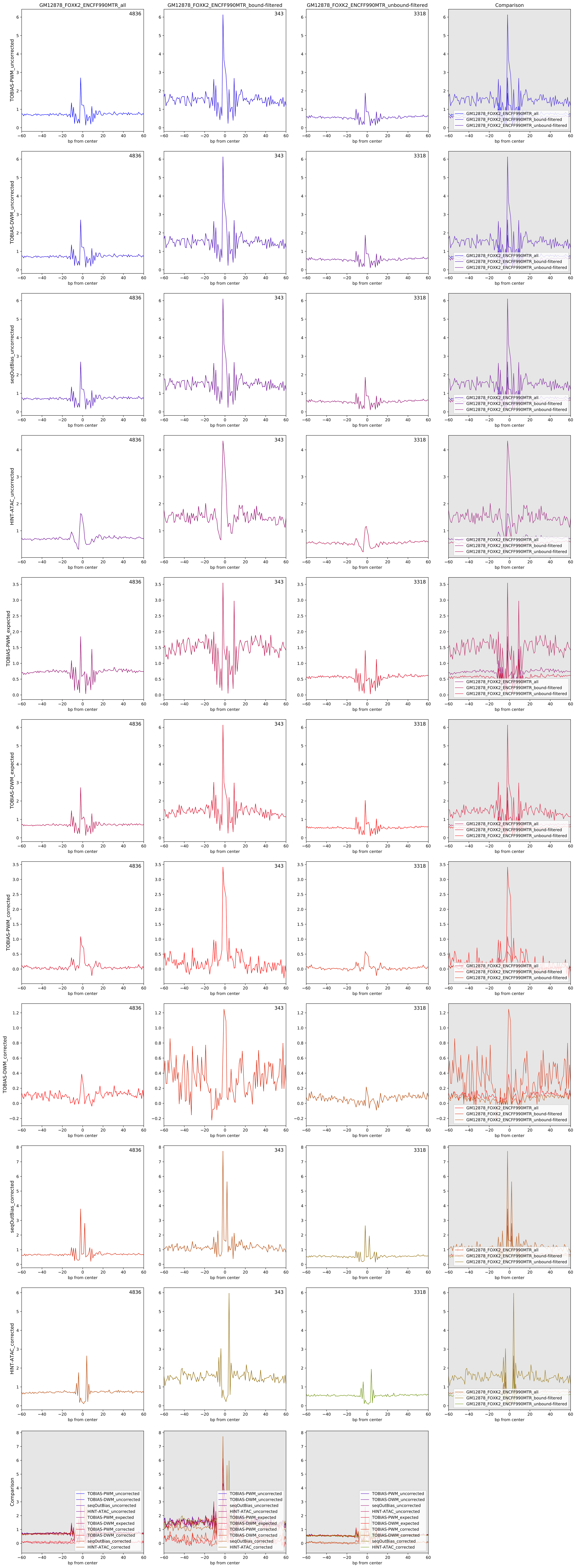

Aggregate footprints in GM12878 for TF GABPA (ChIP-seq ENCFF946ACA)

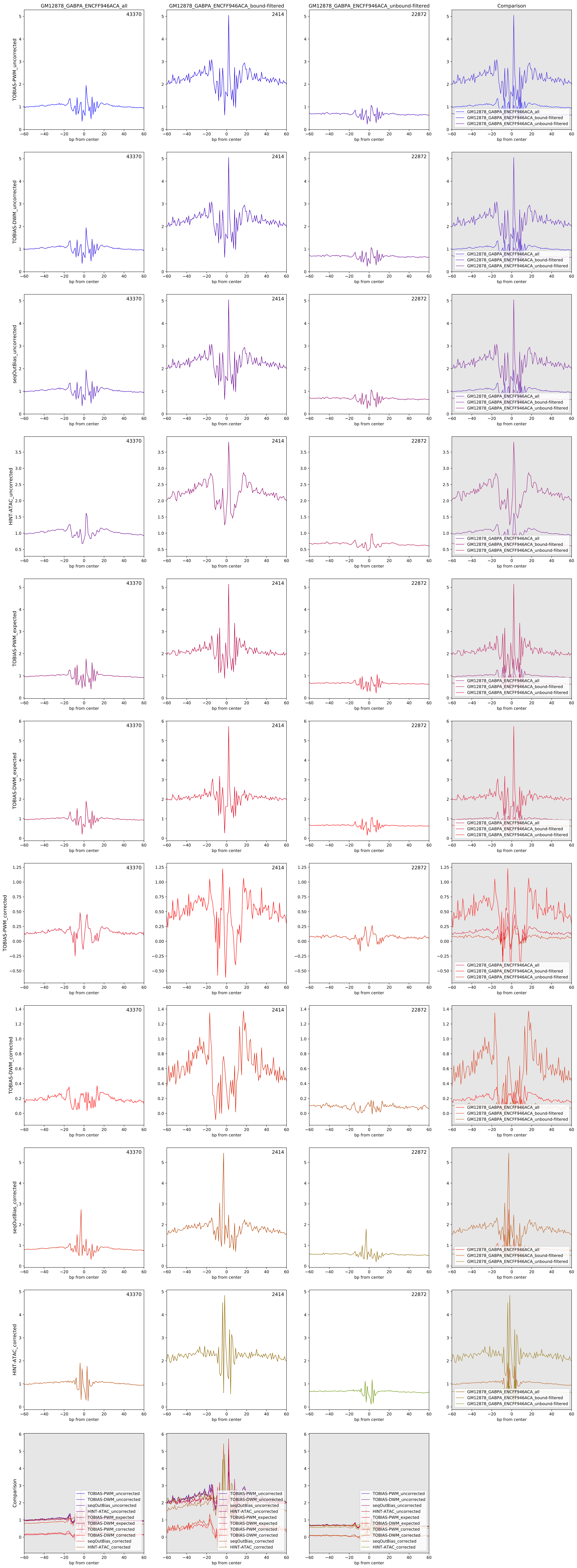

Aggregate footprints in GM12878 for TF HSF1 (ChIP-seq ENCFF603BID)

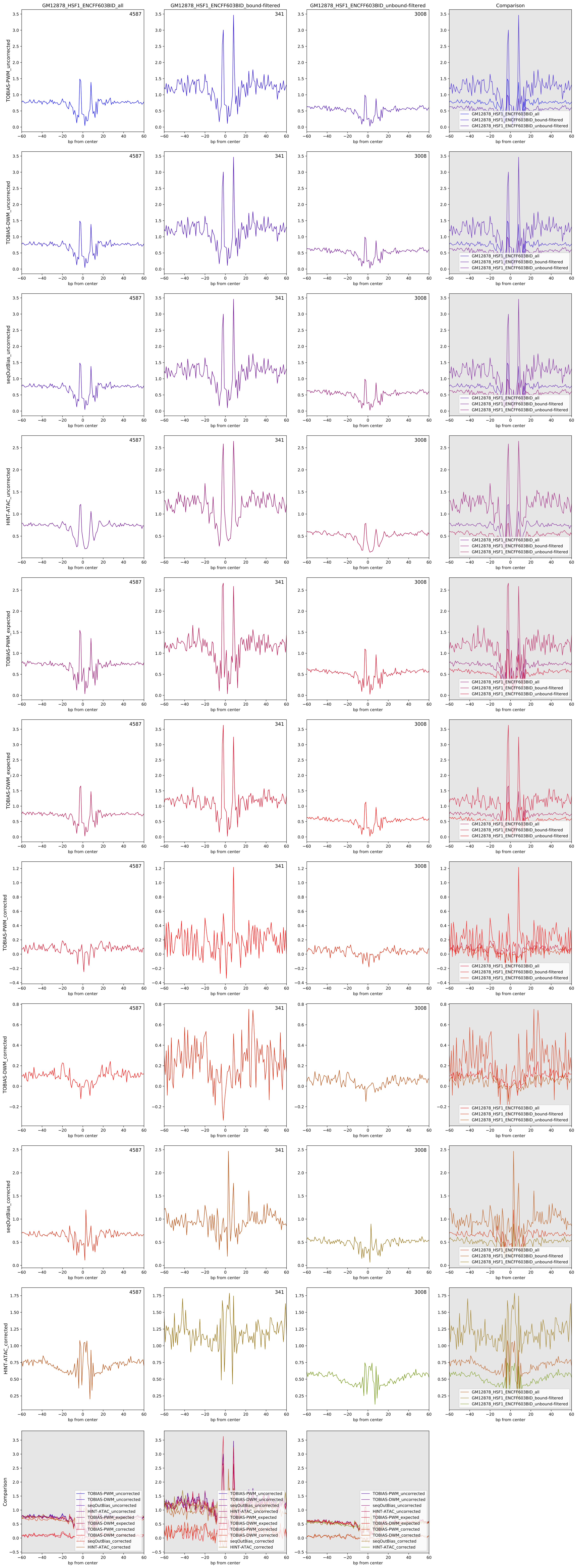

Aggregate footprints in GM12878 for TF IRF4 (ChIP-seq ENCFF720YMW)

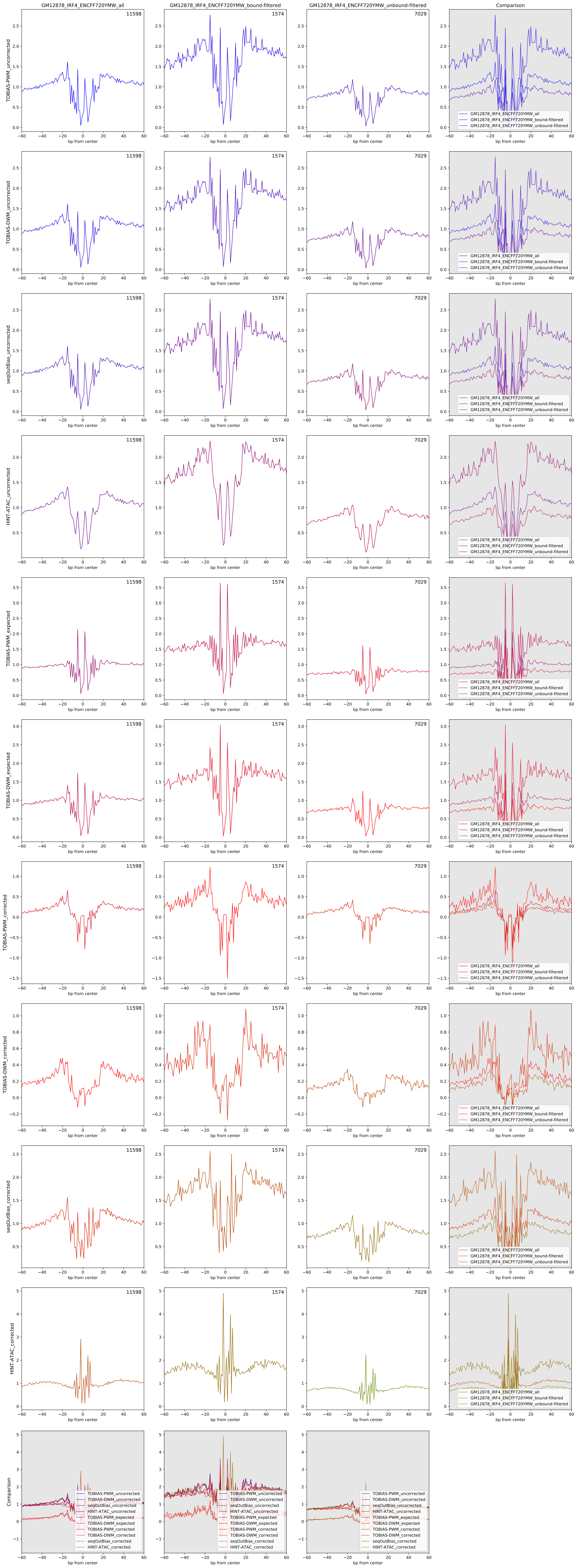

Aggregate footprints in GM12878 for TF JUND (ChIP-seq ENCFF873DJD)

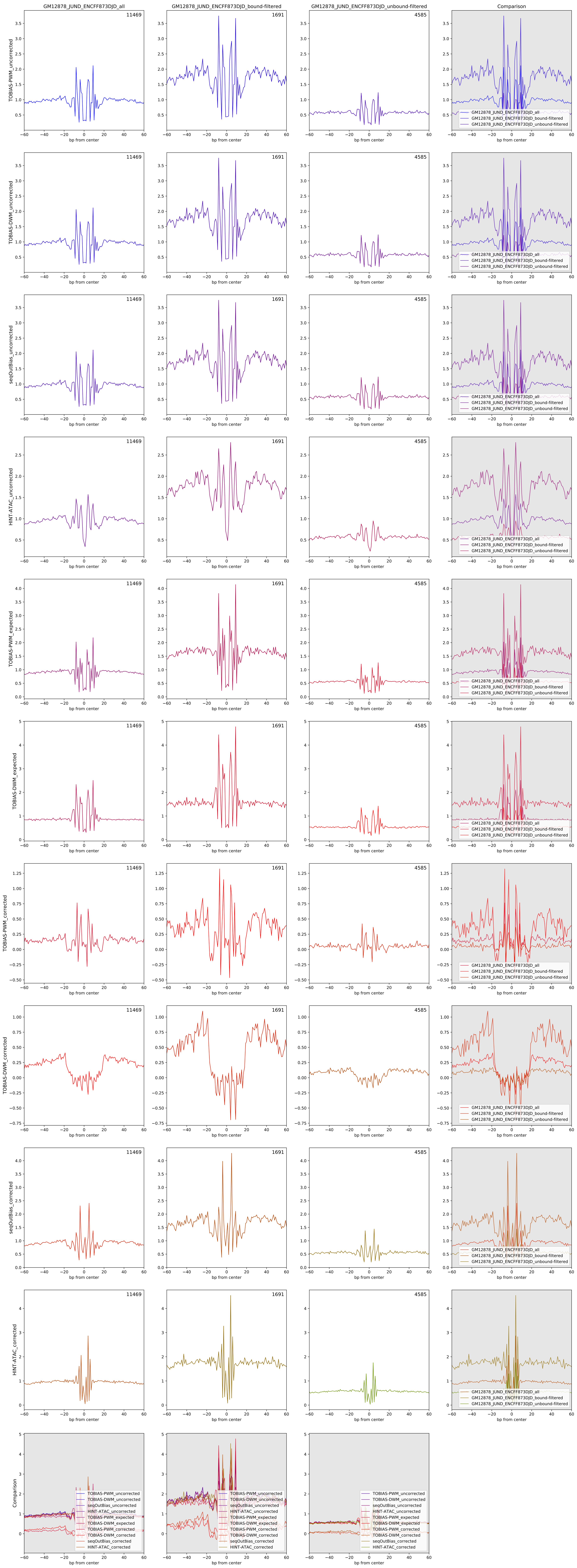

Aggregate footprints in GM12878 for TF MAFK (ChIP-seq ENCFF186AWV)

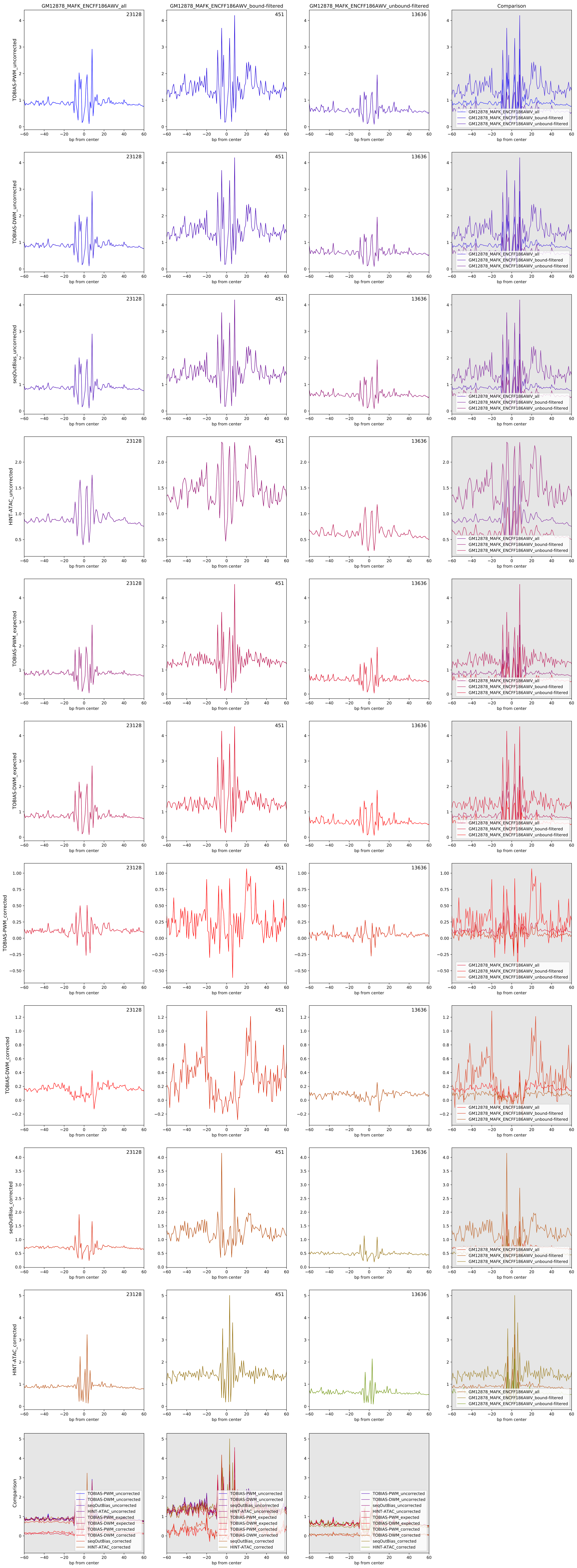

Aggregate footprints in GM12878 for TF MAX (ChIP-seq ENCFF270NAL)

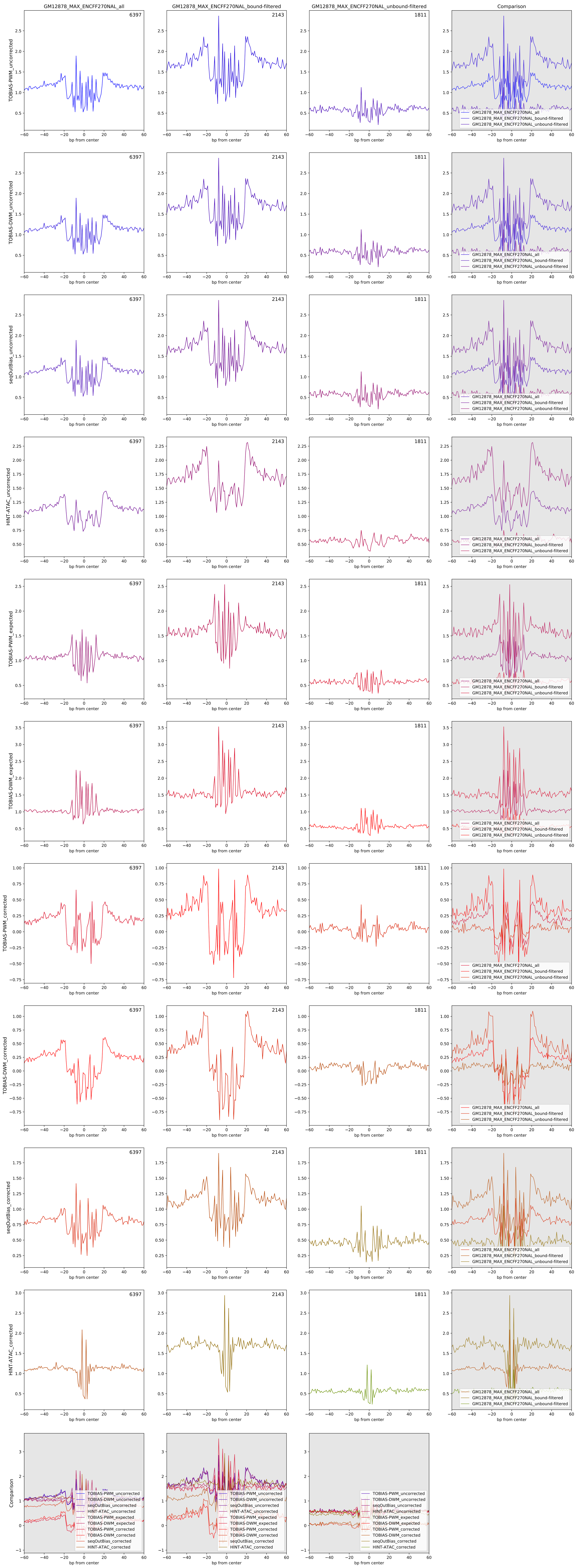

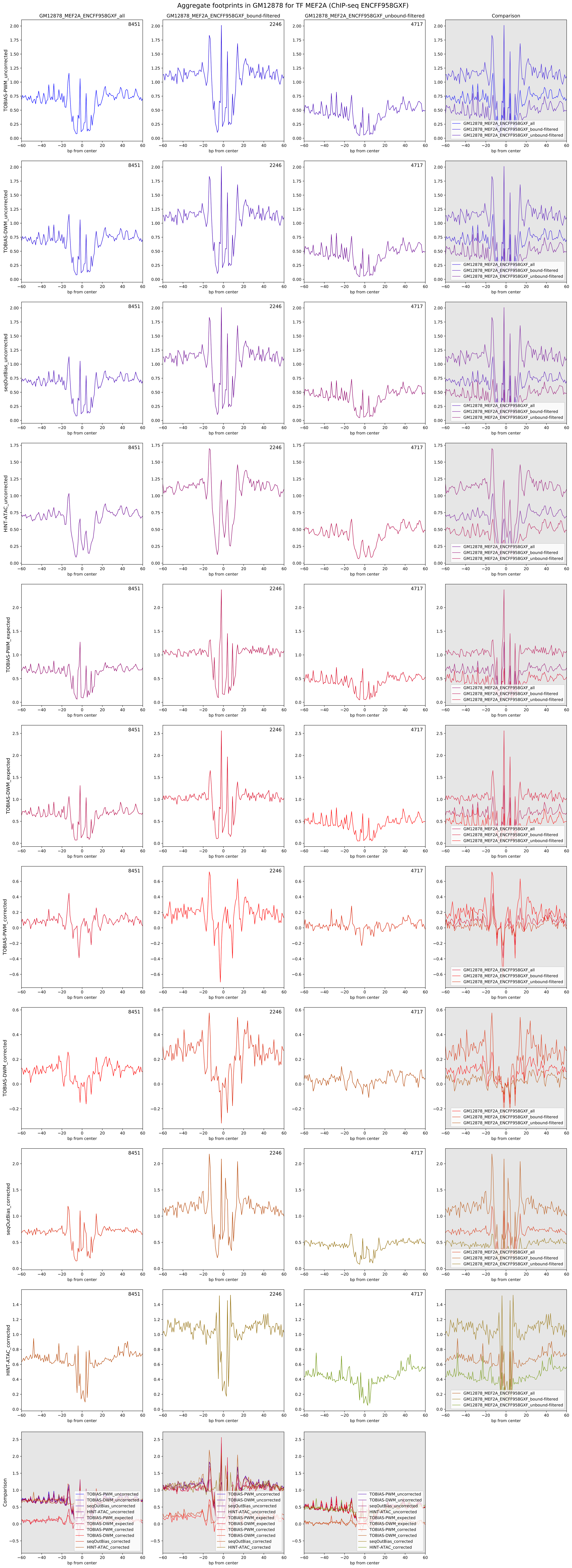
Aggregate footprints in GM12878 for TF MEF2A (ChIP-seq ENCFF958GXF)
GM12878_MEF2A_ENCFF958GXF_all
GM12878_MEF2A_ENCFF958GXF_bound-filtered
GM12878_MEF2A_ENCFF958GXF_unbound-filtered
Comparison
TOBIAS-PWM_uncorrected
TOBIAS-DWM_uncorrected
seqOutBias_uncorrected
HINT-ATAC_uncorrected
TOBIAS-PWM_expected
TOBIAS-DWM_expected
TOBIAS-PWM_corrected
TOBIAS-DWM_corrected
seqOutBias_corrected
HINT-ATAC_corrected
8451
2246
4717
bp from center

Aggregate footprints in GM12878 for TF MEF2B (ChIP-seq ENCFF623FAW)

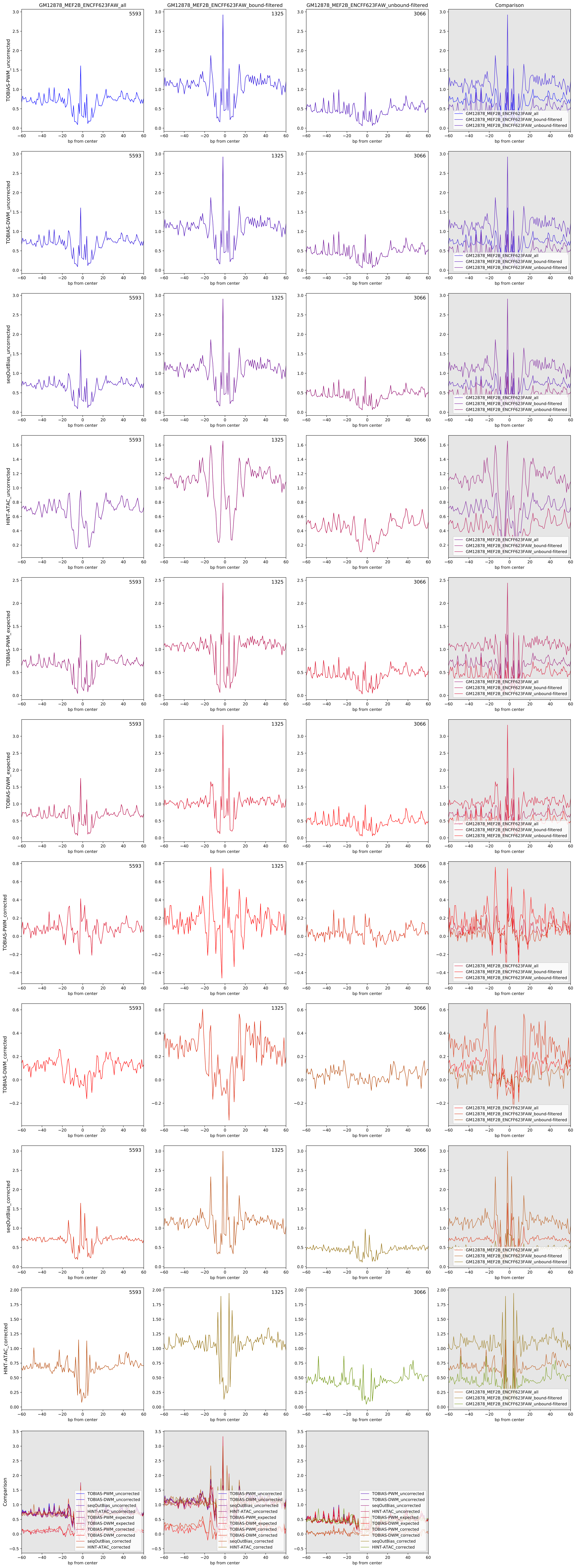

Aggregate footprints in GM12878 for TF MEF2C (ChIP-seq ENCFF830BRO)

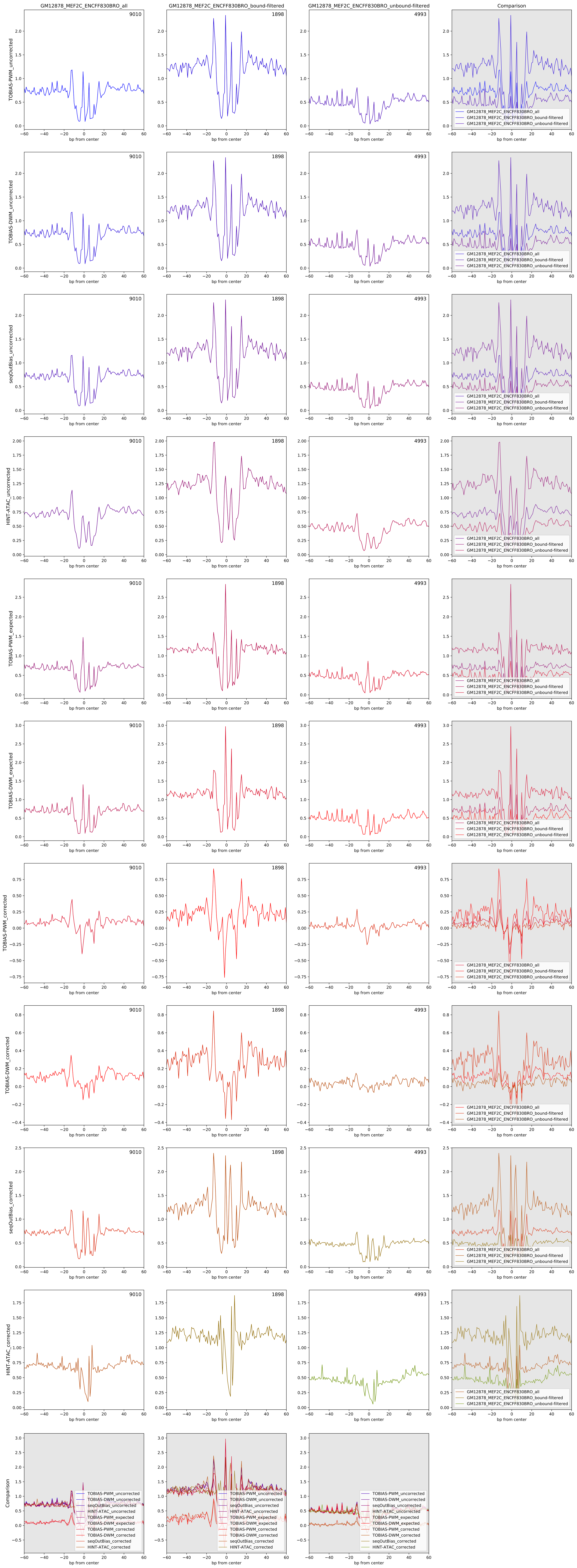

Aggregate footprints in GM12878 for TF MXI1 (ChIP-seq ENCFF199HGX)

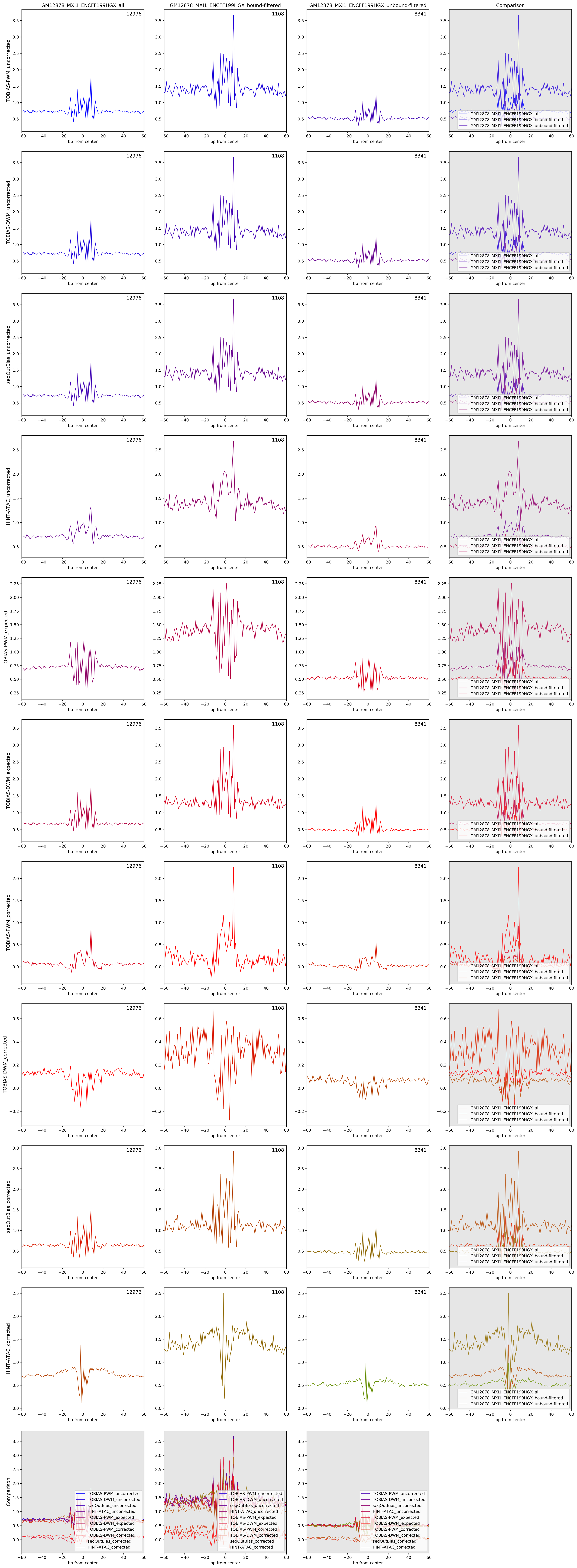

Aggregate footprints in GM12878 for TF NFATC1 (ChIP-seq ENCFF138ZBJ)

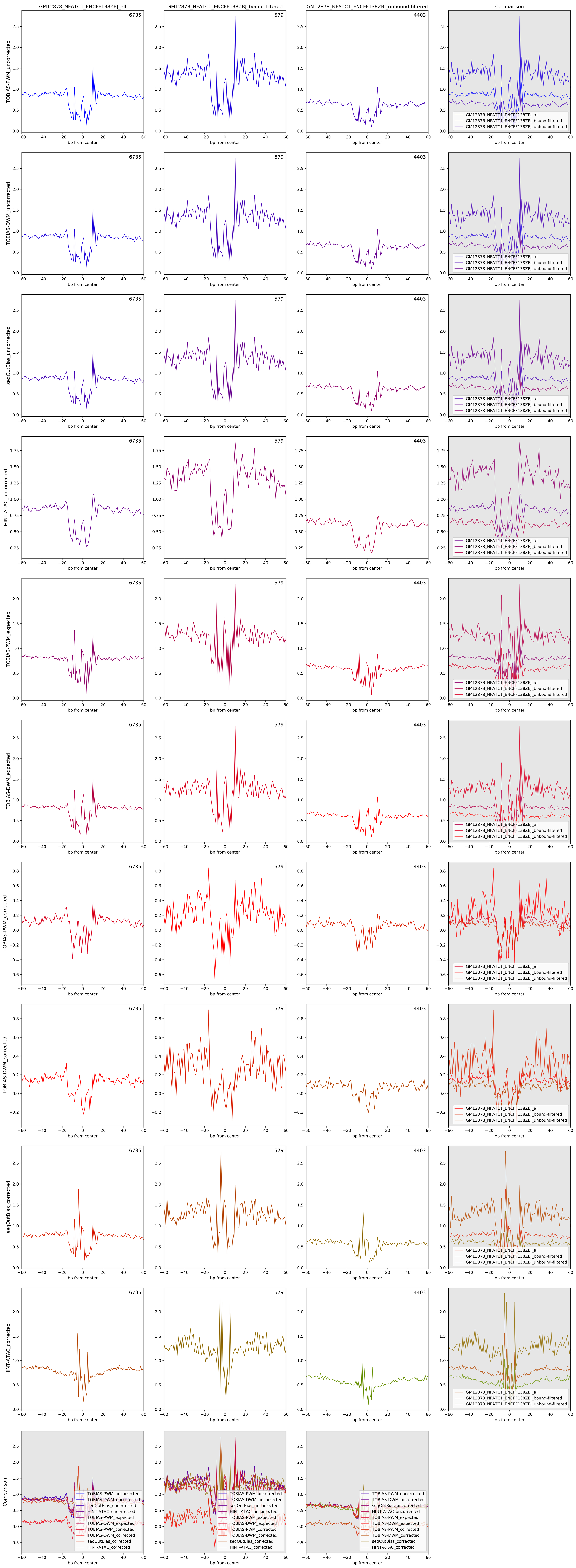

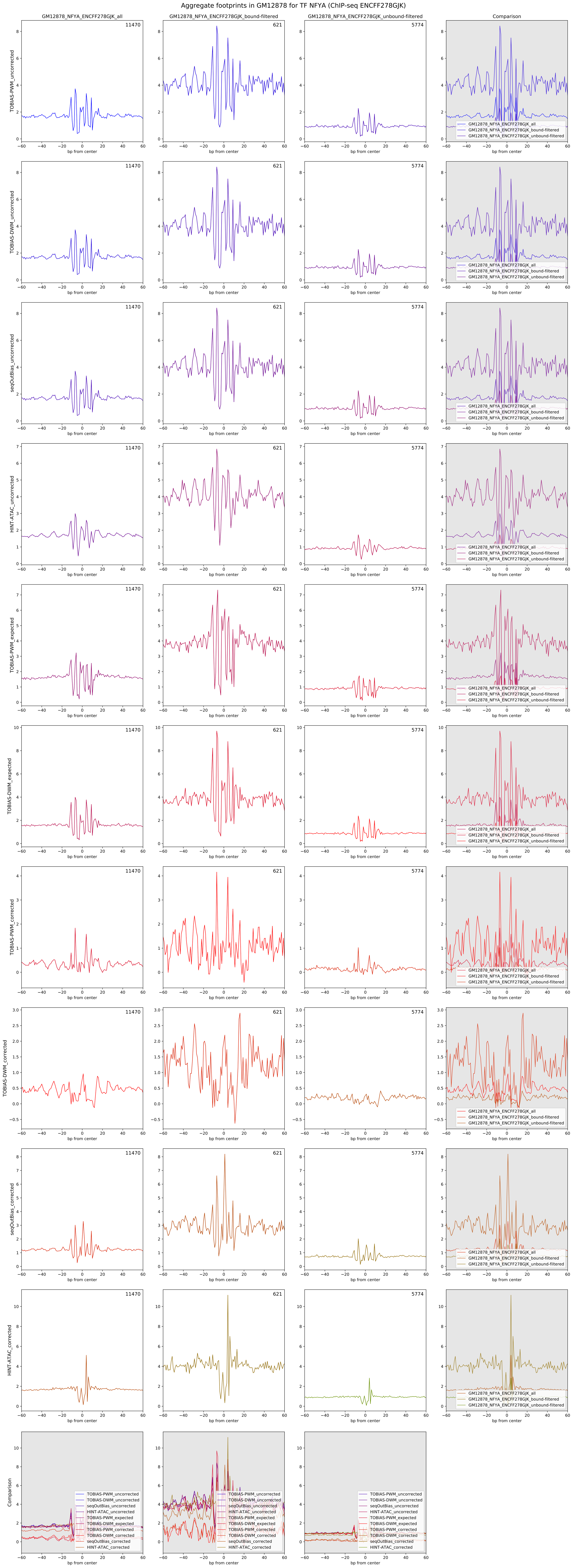
Aggregate footprints in GM12878 for TF NFYA (ChIP-seq ENCFF278GJK)
GM12878_NFYA_ENCFF278GJK_all
GM12878_NFYA_ENCFF278GJK_bound-filtered
GM12878_NFYA_ENCFF278GJK_unbound-filtered
Comparison
11470
621
5774
TOBIAS-PWM_uncorrected
TOBIAS-DWM_uncorrected
seqOutBias_uncorrected
HINT-ATAC_uncorrected
TOBIAS-PWM_expected
TOBIAS-DWM_expected
TOBIAS-PWM_corrected
TOBIAS-DWM_corrected
seqOutBias_corrected
HINT-ATAC_corrected
bp from center

Aggregate footprints in GM12878 for TF NFYB (ChIP-seq ENCFF510NDO)

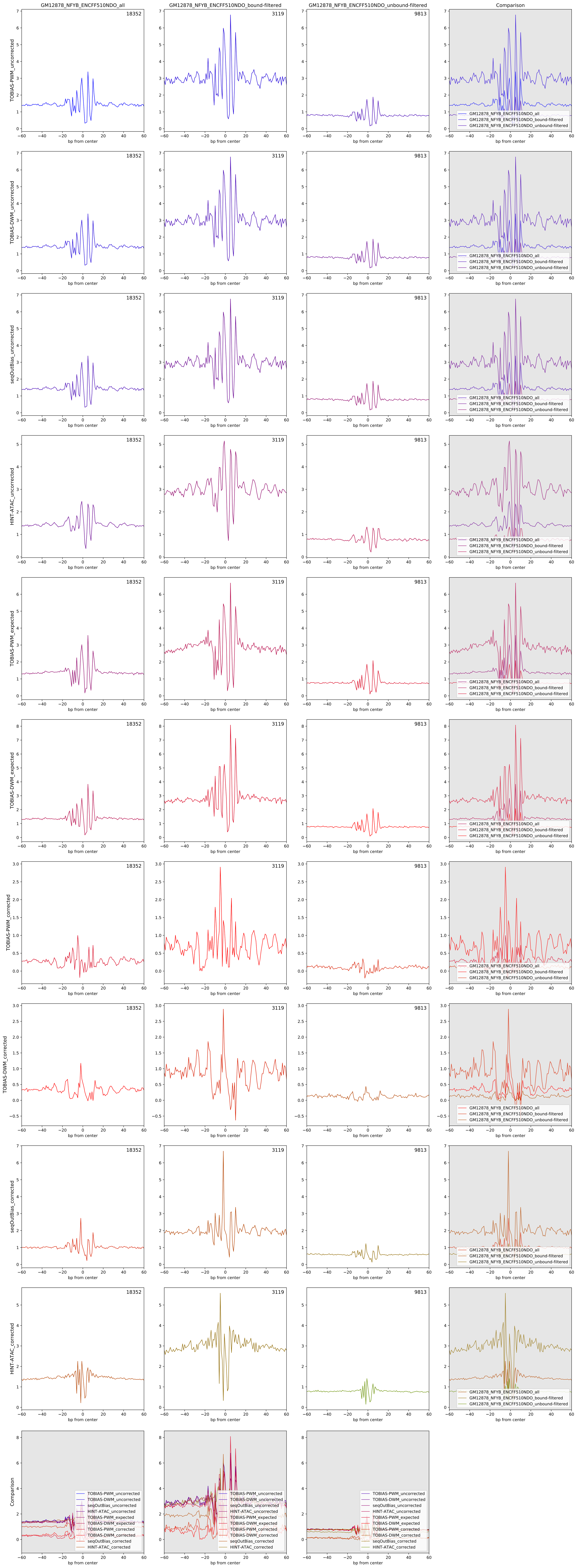

Aggregate footprints in GM12878 for TF NR2C2 (ChIP-seq ENCFF434HVY)

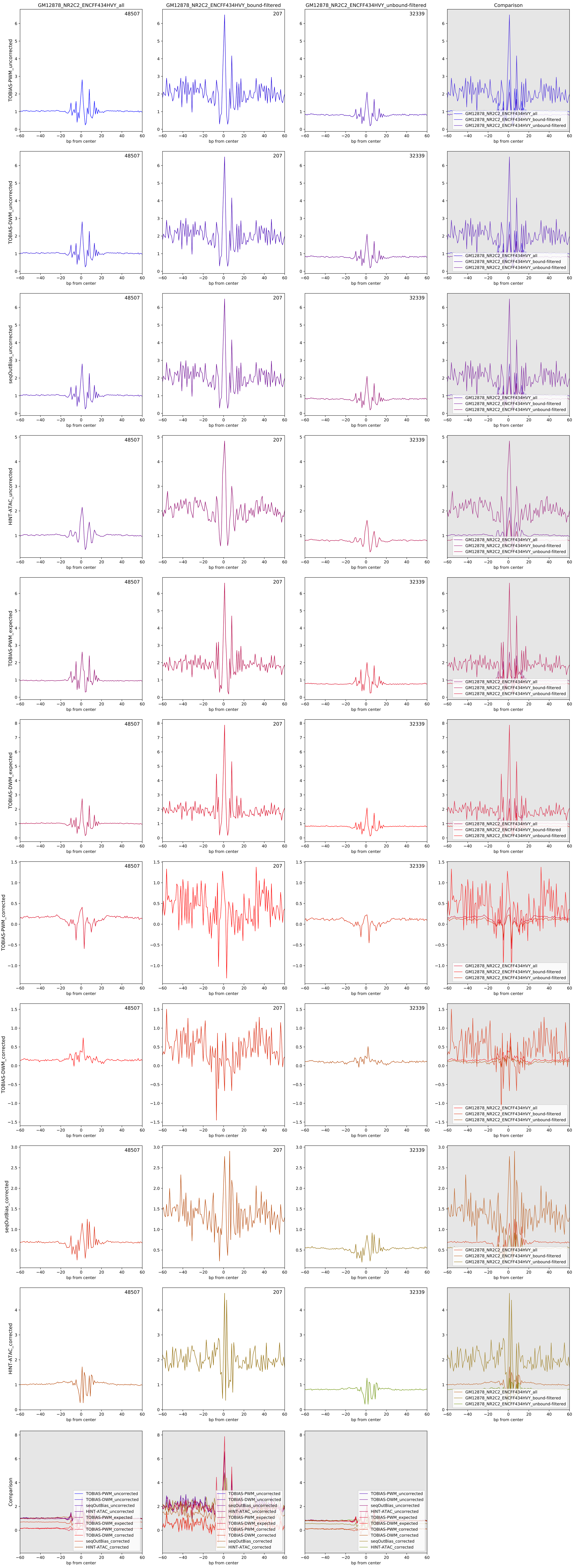

Aggregate footprints in GM12878 for TF NR2F1 (ChIP-seq ENCFF531KOV)

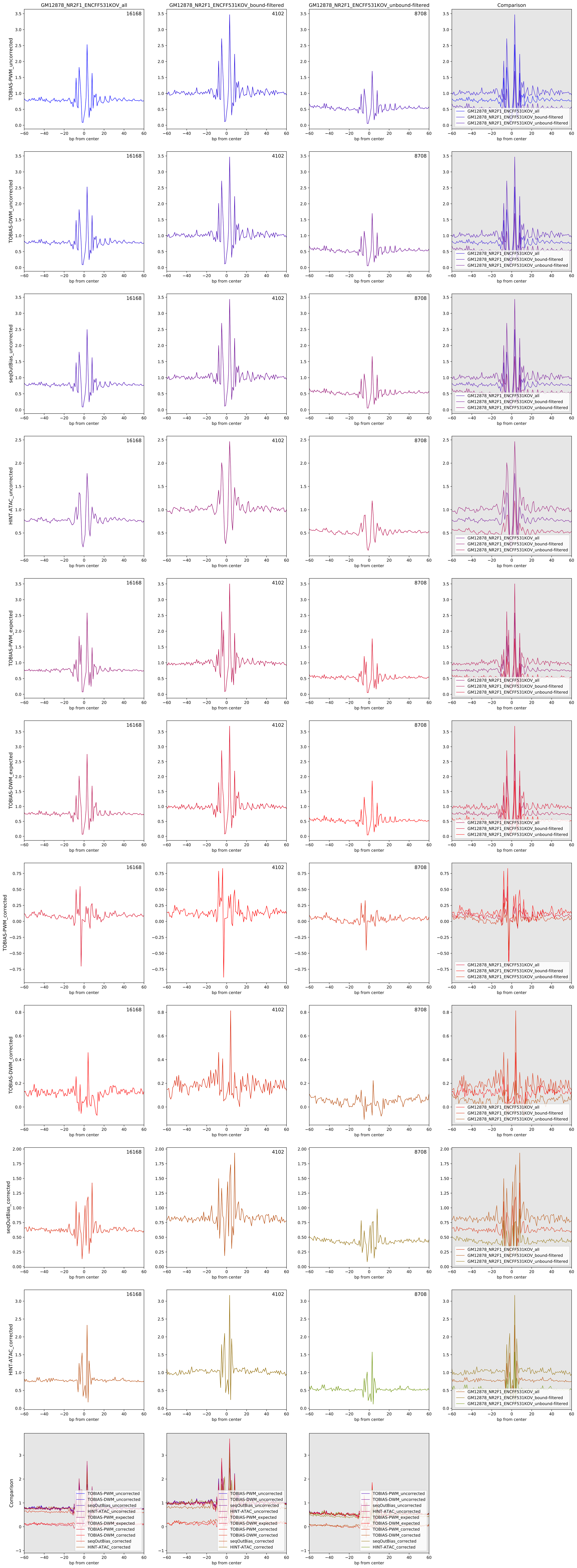

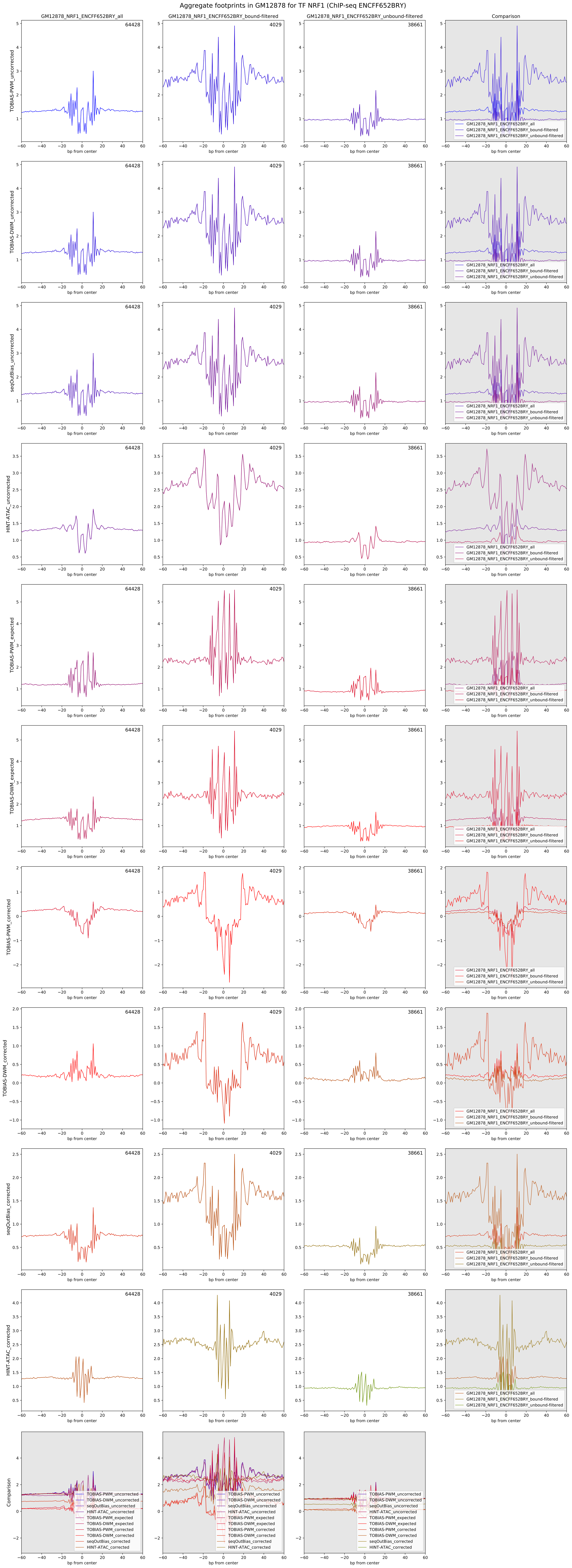

Aggregate footprints in GM12878 for TF NRF1 (ChIP-seq ENCFF652BRY)
GM12878_NRF1_ENCFF652BRY_all
GM12878_NRF1_ENCFF652BRY_bound-filtered
GM12878_NRF1_ENCFF652BRY_unbound-filtered
Comparison
64428
4029
38661
TOBIAS-PWM_uncorrected
TOBIAS-DWM_uncorrected
seqOutBias_uncorrected
HINT-ATAC_uncorrected
TOBIAS-PWM_expected
TOBIAS-DWM_expected
TOBIAS-PWM_corrected
TOBIAS-DWM_corrected
seqOutBias_corrected
HINT-ATAC_corrected
bp from center

Aggregate footprints in GM12878 for TF PAX5 (ChIP-seq ENCFF196JGP)

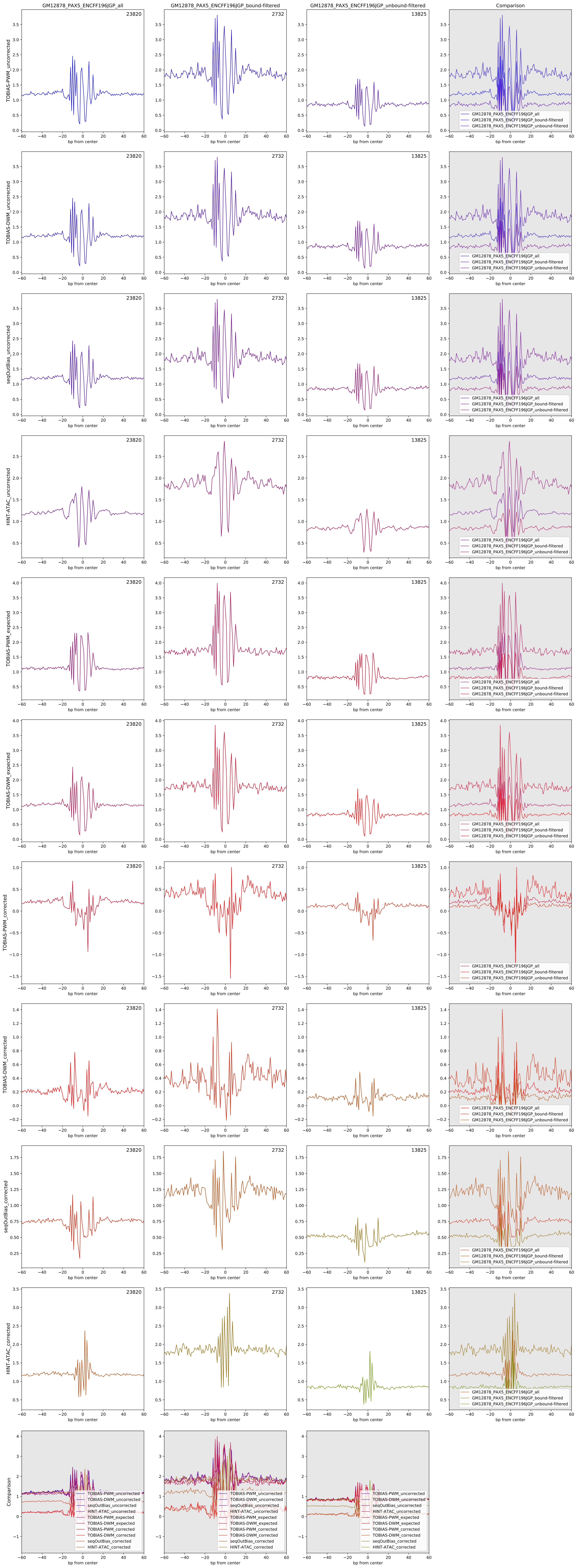

Aggregate footprints in GM12878 for TF PBX3 (ChIP-seq ENCFF926LHG)

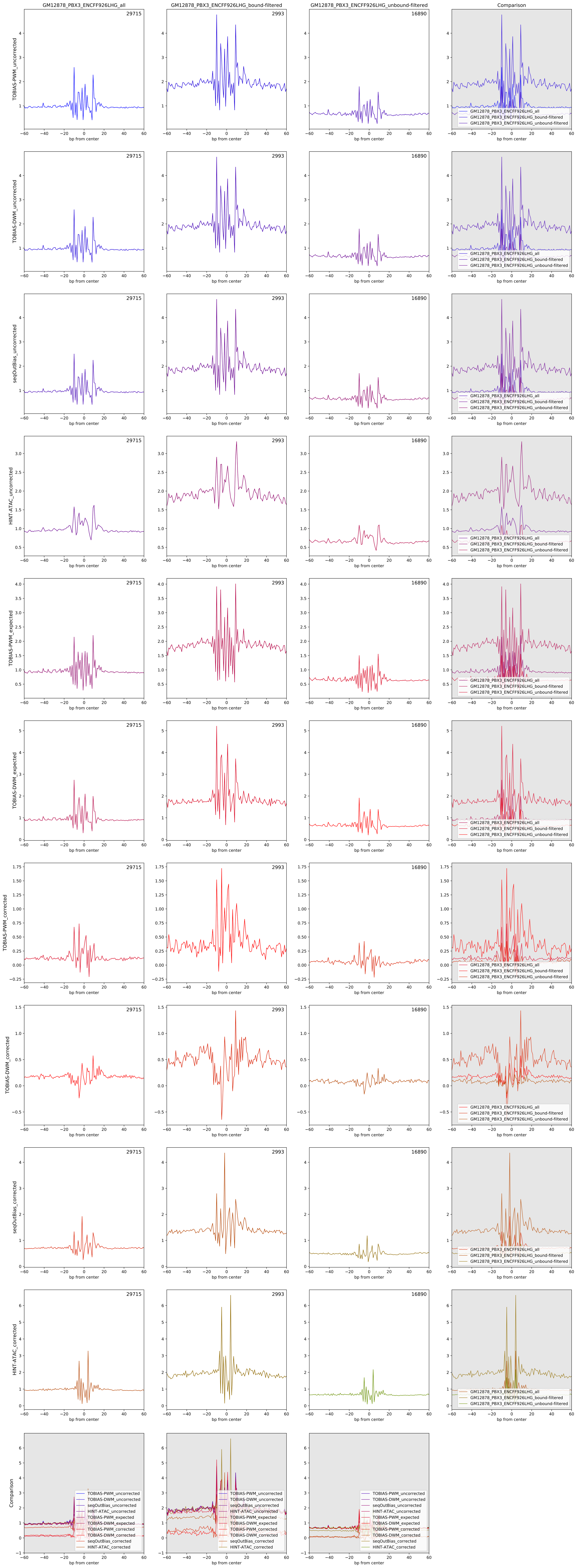

Aggregate footprints in GM12878 for TF PKNOX1 (ChIP-seq ENCFF335ADU)

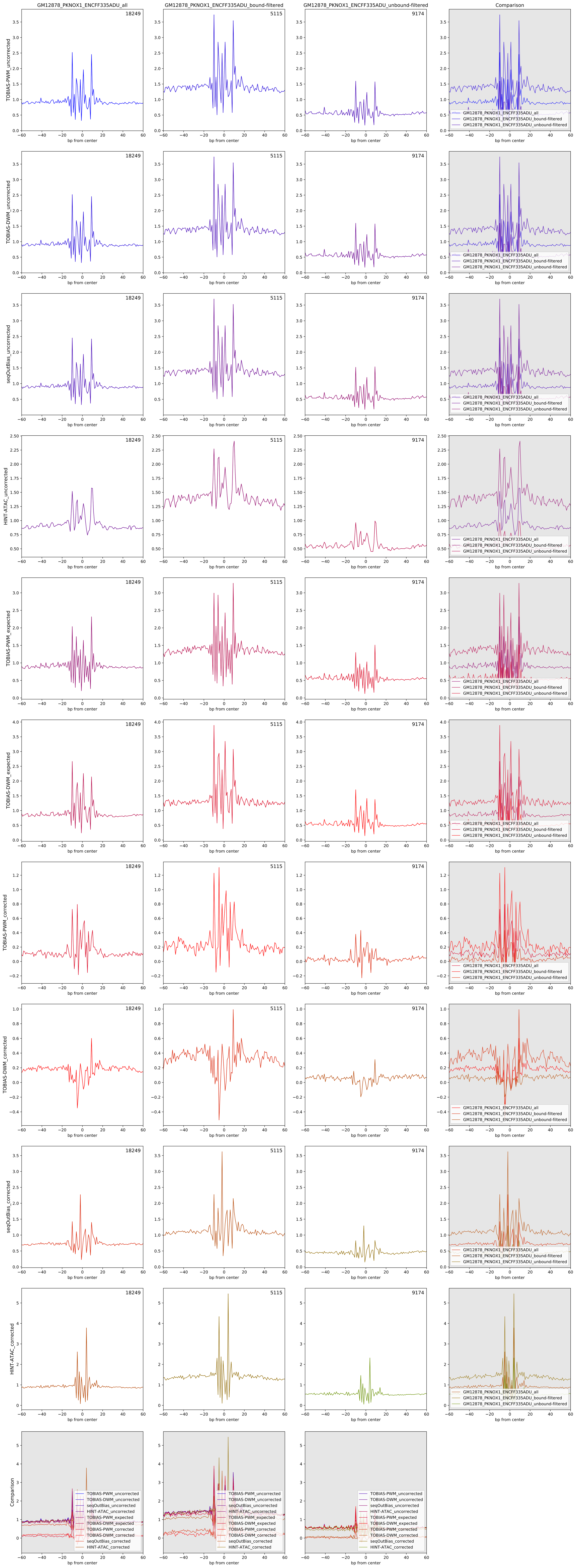

Aggregate footprints in GM12878 for TF RELB (ChIP-seq ENCFF105YDI)

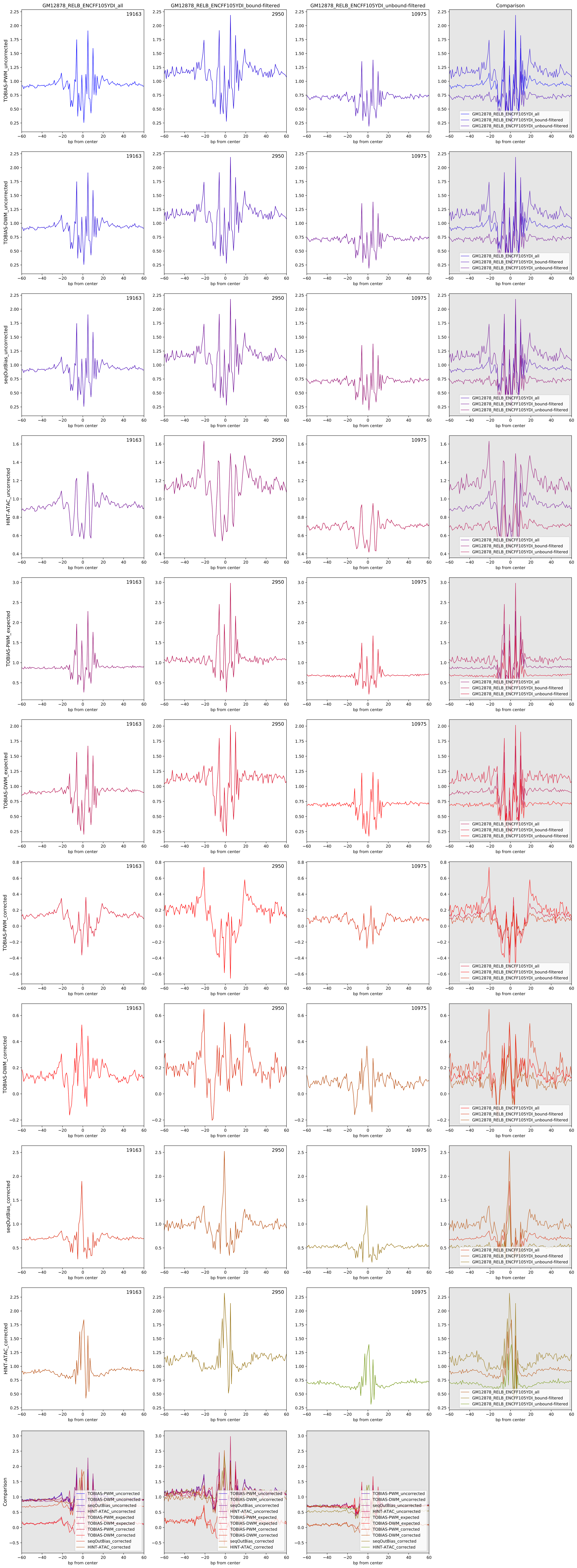

Aggregate footprints in GM12878 for TF REST (ChIP-seq ENCFF274BBE)

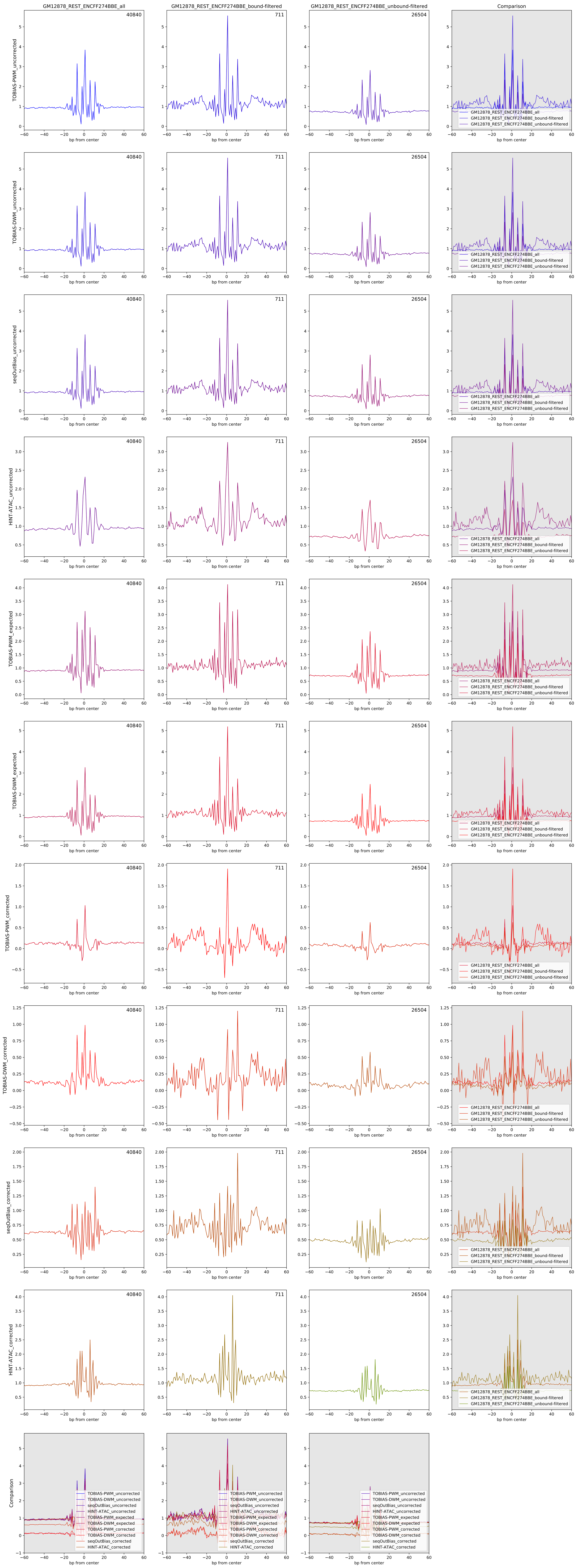

Aggregate footprints in GM12878 for TF RFX5 (ChIP-seq ENCFF259LNG)

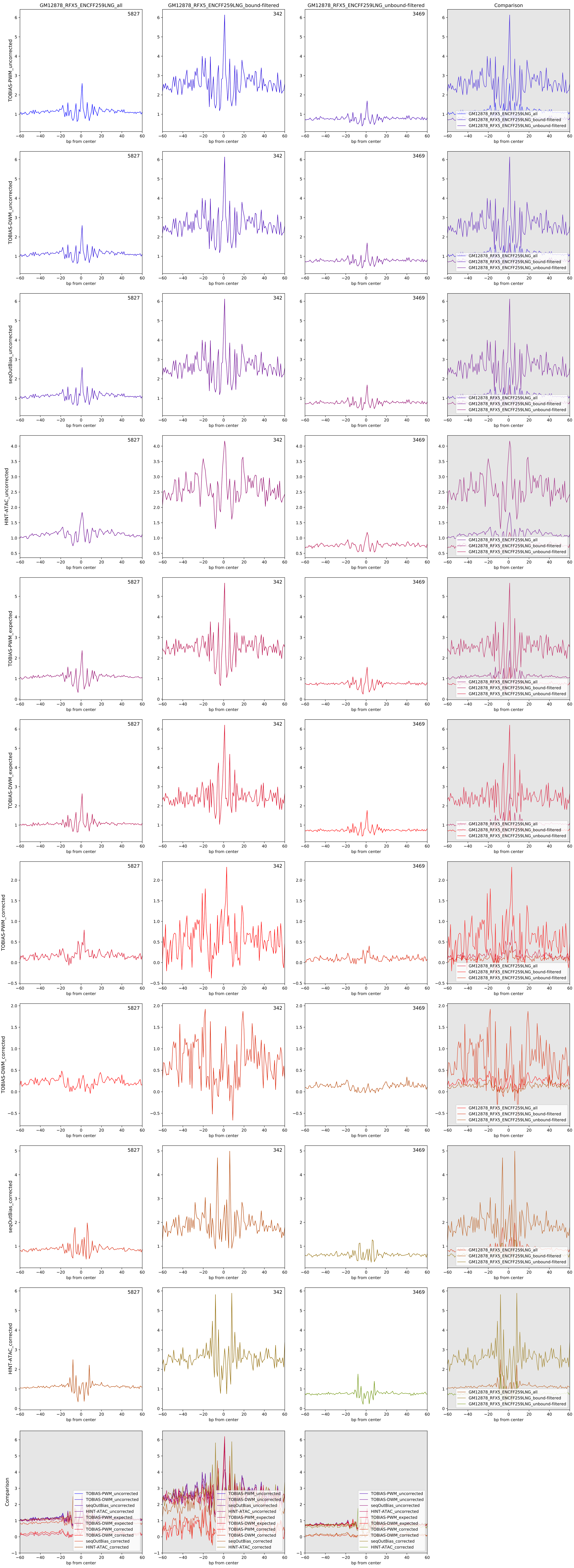

Aggregate footprints in GM12878 for TF RUNX3 (ChIP-seq ENCFF677QUK)

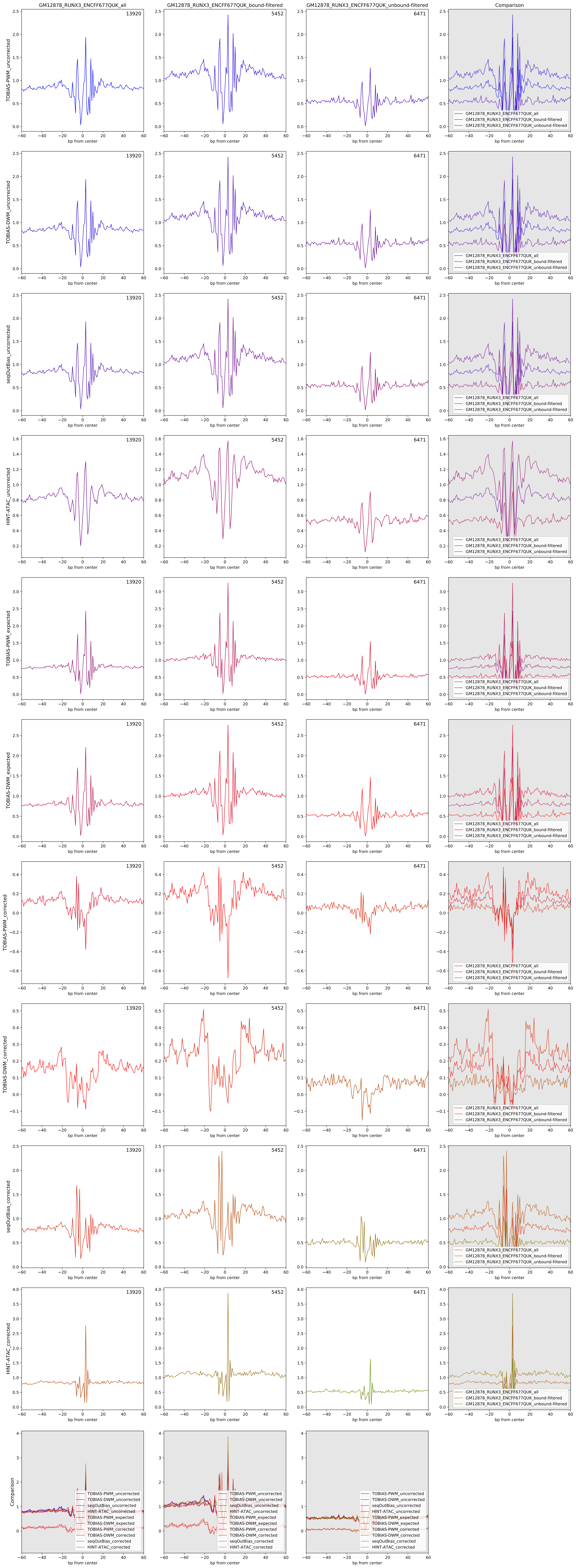

Aggregate footprints in GM12878 for TF SRF (ChIP-seq ENCFF766WWB)

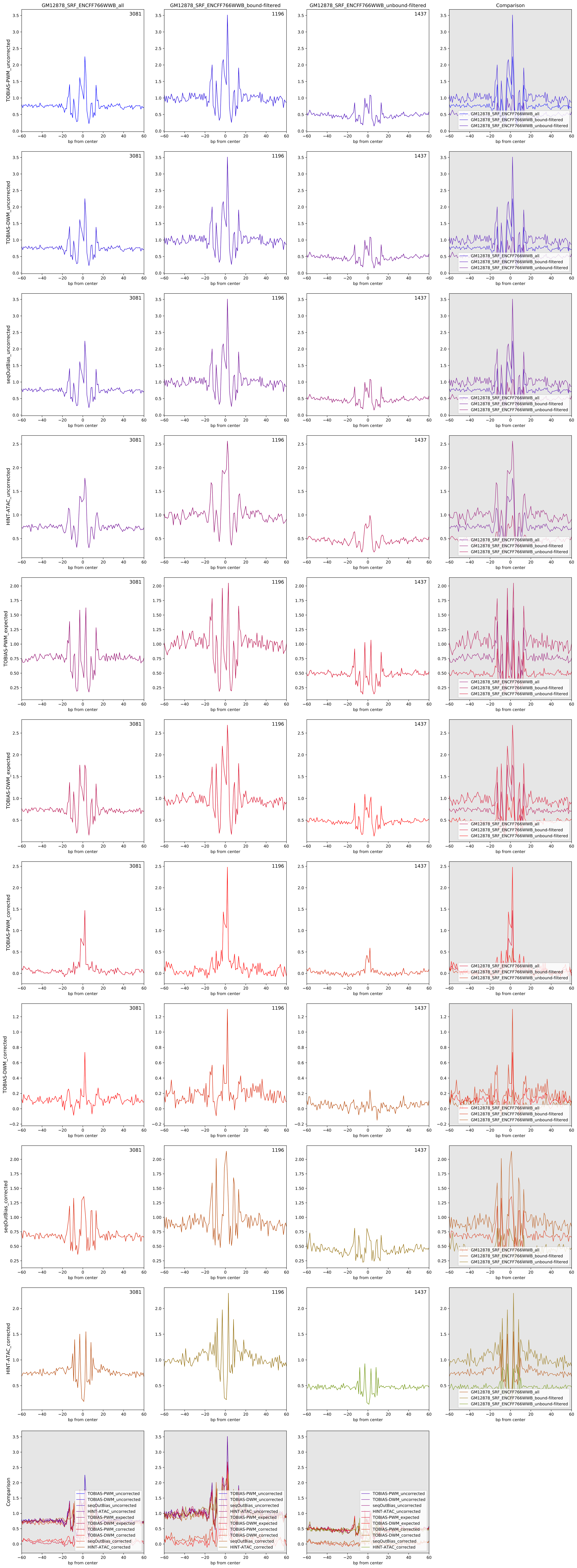

Aggregate footprints in GM12878 for TF TBP (ChIP-seq ENCFF896UZB)

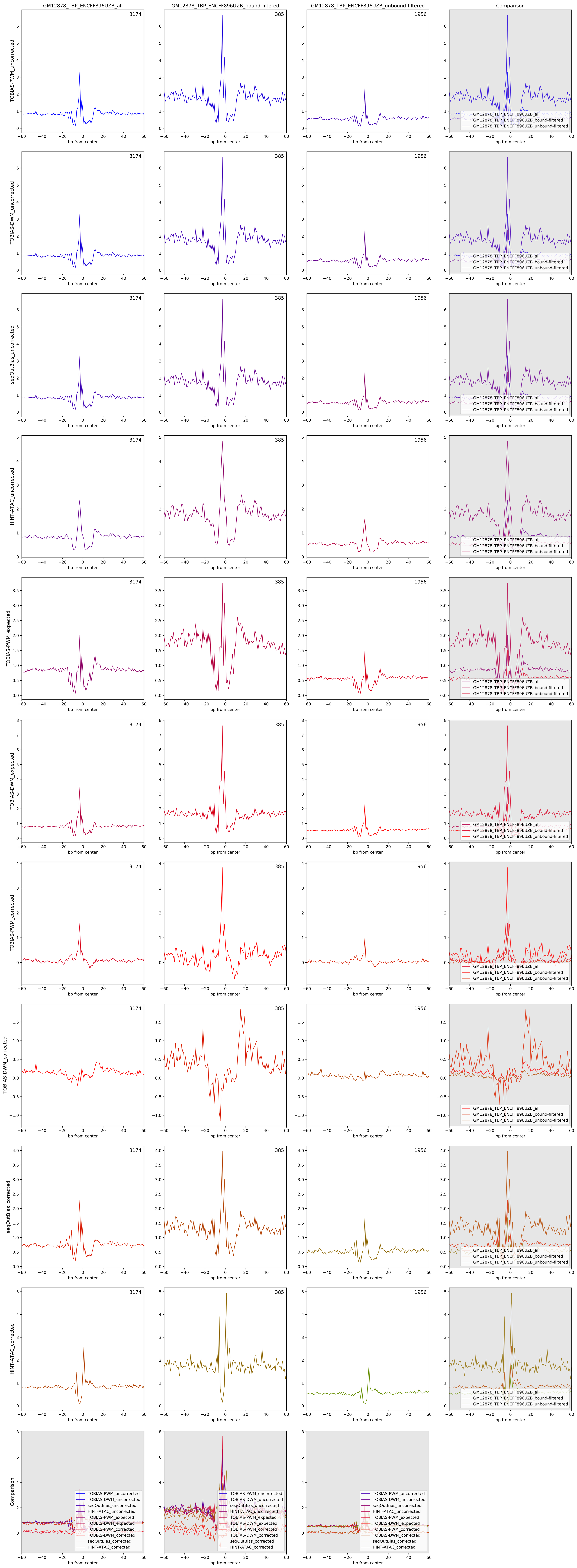

Aggregate footprints in GM12878 for TF TBX21 (ChIP-seq ENCFF971VHK)

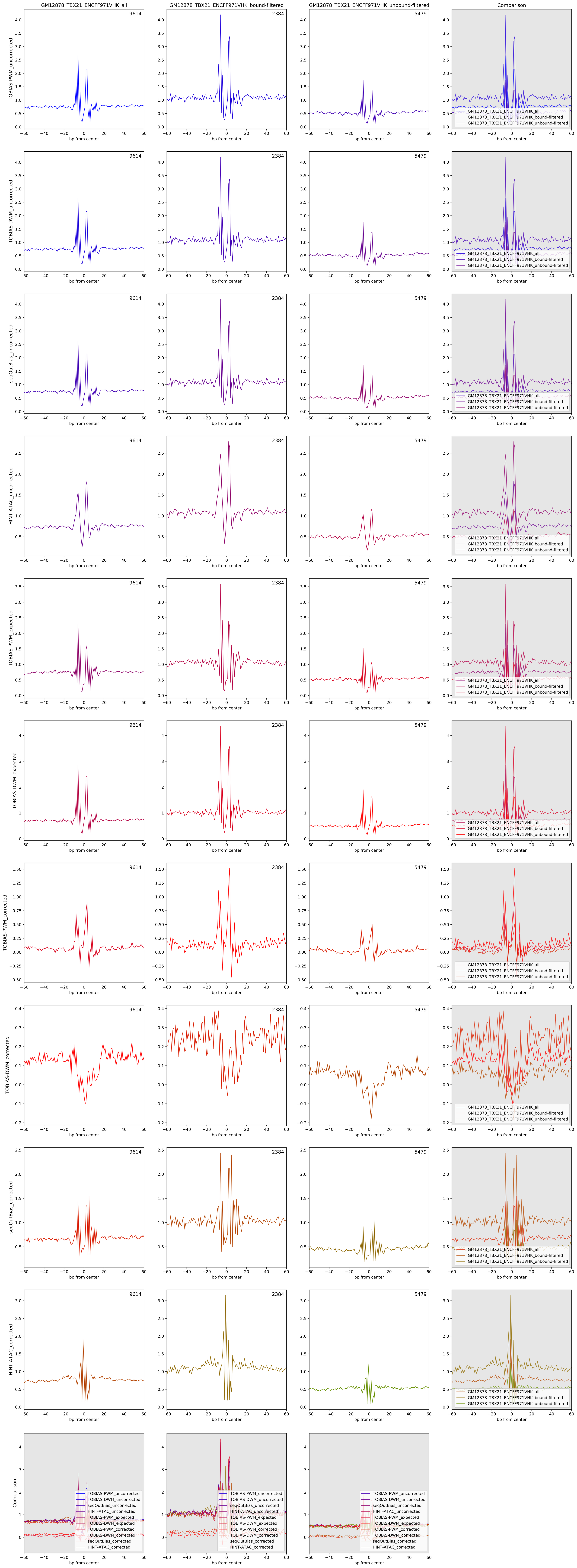

Aggregate footprints in GM12878 for TF TCF12 (ChIP-seq ENCFF768VSH)

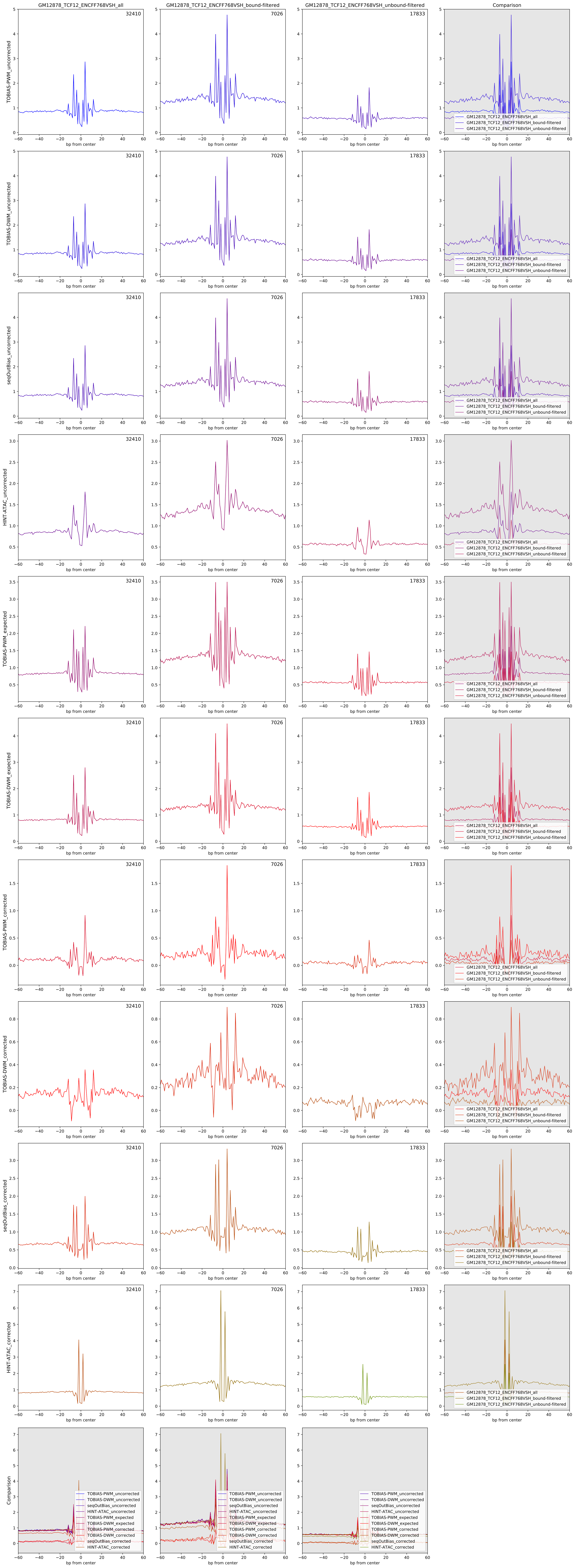

Aggregate footprints in GM12878 for TF TCF7 (ChIP-seq ENCFF152RNE)

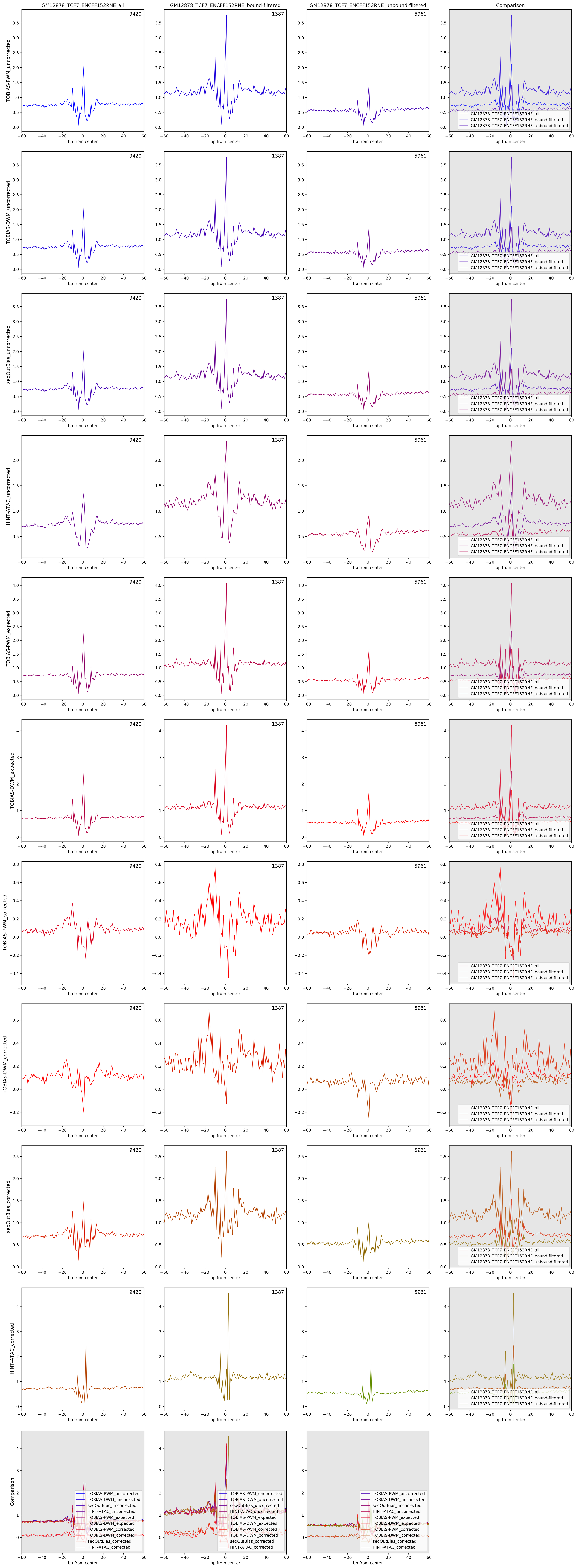

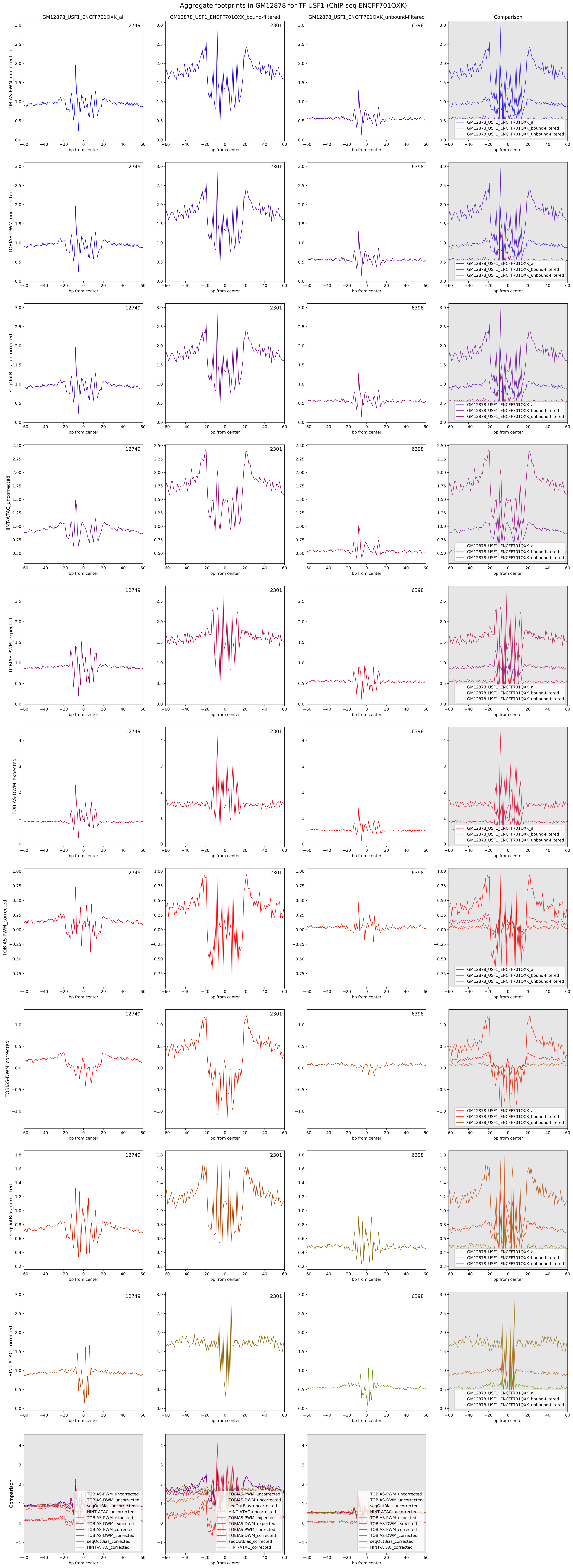

Aggregate footprints in GM12878 for TF USF1 (ChIP-seq ENCFF701QXK)
GM12878_USF1_ENCFF701QXK_all
GM12878_USF1_ENCFF701QXK_bound-filtered
GM12878_USF1_ENCFF701QXK_unbound-filtered
Comparison
TOBIAS-PWM_uncorrected
TOBIAS-DWM_uncorrected
seqOutBias_uncorrected
HINT-ATAC_uncorrected
TOBIAS-PWM_expected
TOBIAS-DWM_expected
TOBIAS-PWM_corrected
TOBIAS-DWM_corrected
seqOutBias_corrected
HINT-ATAC_corrected
12749
2301
6398
bp from center

Aggregate footprints in GM12878 for TF USF2 (ChIP-seq ENCFF514SWA)

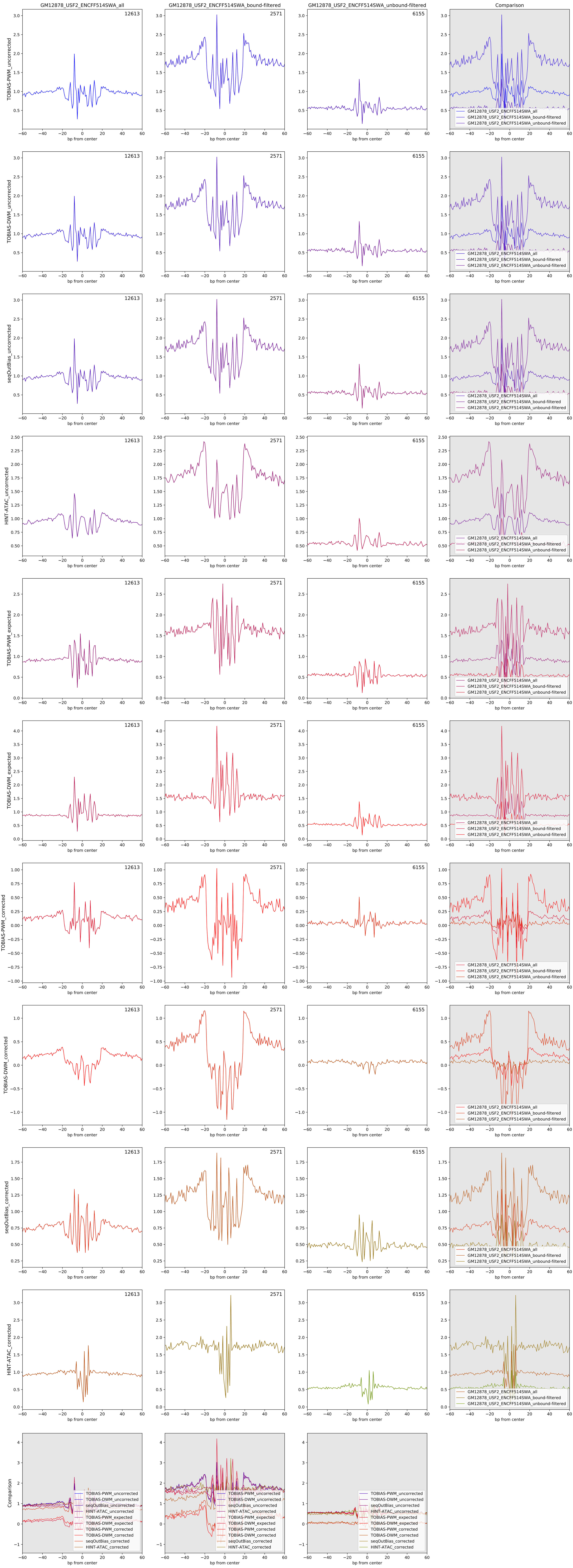

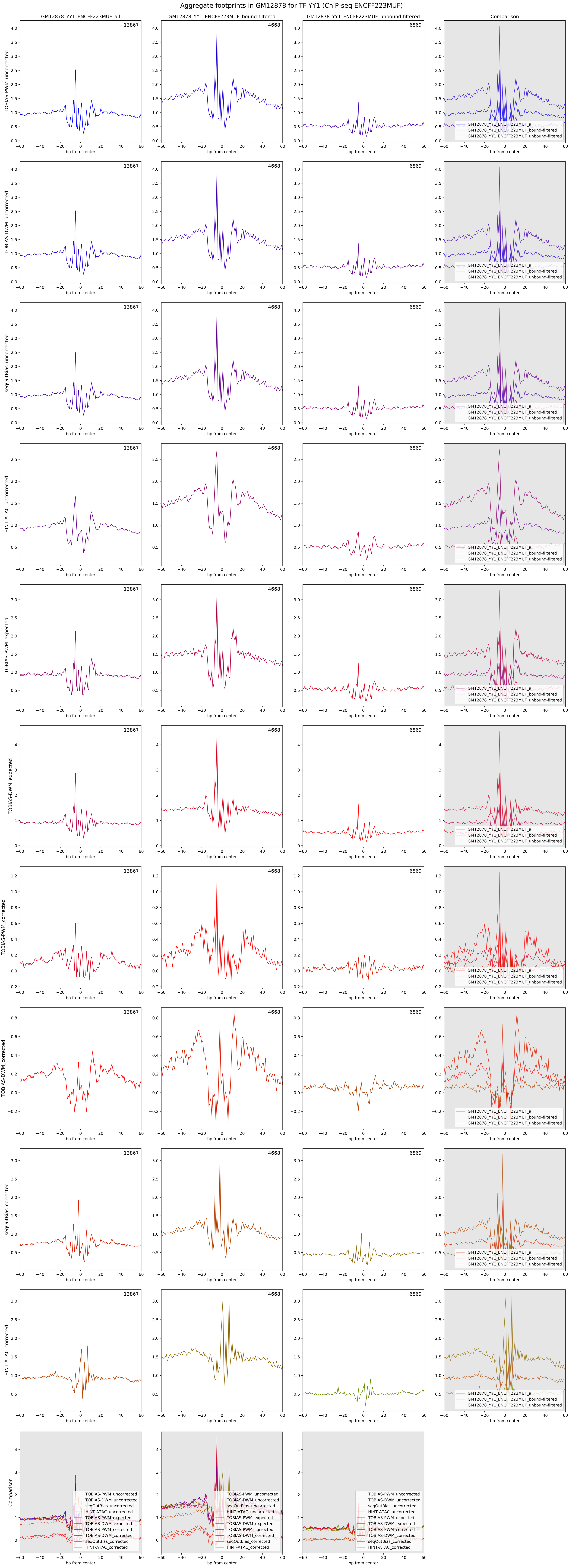
Aggregate footprints in GM12878 for TF YY1 (ChIP-seq ENCFF223MUF)
GM12878_YY1_ENCFF223MUF_all
GM12878_YY1_ENCFF223MUF_bound-filtered
GM12878_YY1_ENCFF223MUF_unbound-filtered
Comparison
TOBIAS-PWM_uncorrected
TOBIAS-DWM_uncorrected
seqOutBias_uncorrected
HINT-ATAC_uncorrected
TOBIAS-PWM_expected
TOBIAS-DWM_expected
TOBIAS-PWM_corrected
TOBIAS-DWM_corrected
seqOutBias_corrected
HINT-ATAC_corrected
Comparison
13867
4668
6869
bp from center
GM12878_YY1_ENCFF223MUF_all
GM12878_YY1_ENCFF223MUF_bound-filtered
GM12878_YY1_ENCFF223MUF_unbound-filtered
TOBIAS-PWM_uncorrected
TOBIAS-DWM_uncorrected
seqOutBias_uncorrected
HINT-ATAC_uncorrected
TOBIAS-PWM_expected
TOBIAS-DWM_expected
TOBIAS-PWM_corrected
TOBIAS-DWM_corrected
seqOutBias_corrected
HINT-ATAC_corrected

Aggregate footprints in GM12878 for TF ZBED1 (ChIP-seq ENCFF630FLK)

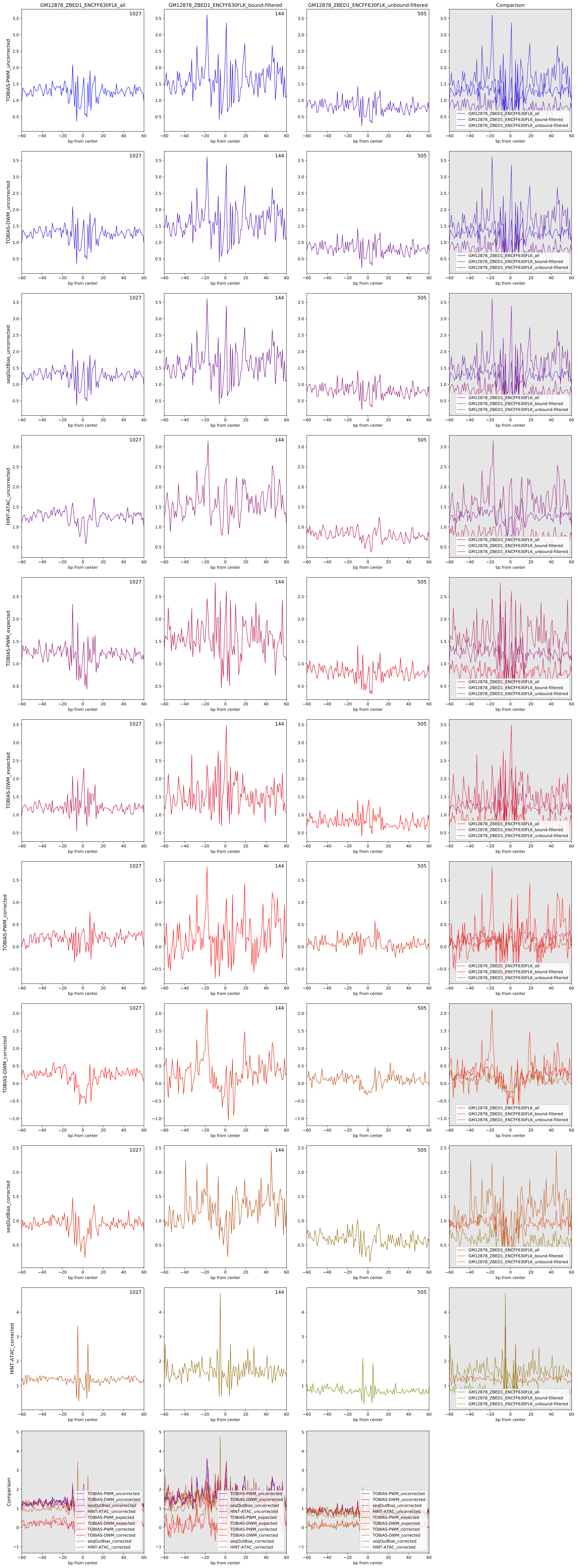

Aggregate footprints in GM12878 for TF ZBTB33 (ChIP-seq ENCFF773OQL)

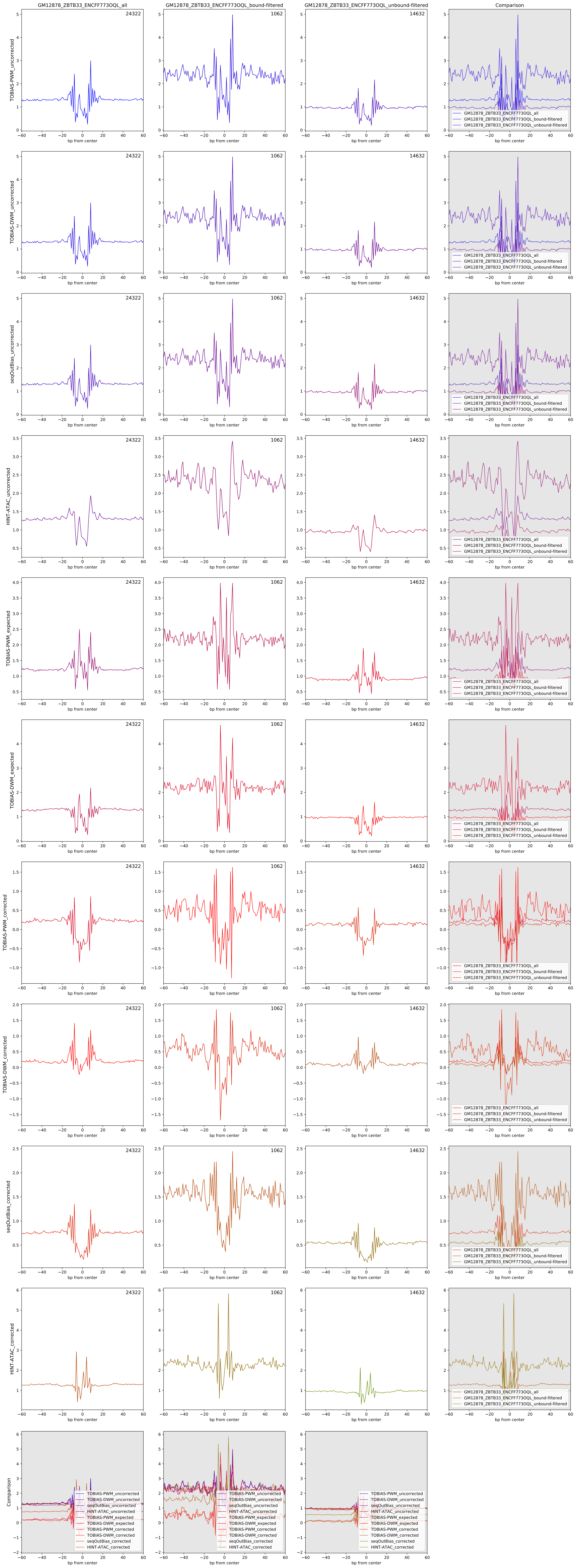

Aggregate footprints in GM12878 for TF ZEB1 (ChIP-seq ENCFF204LCG)

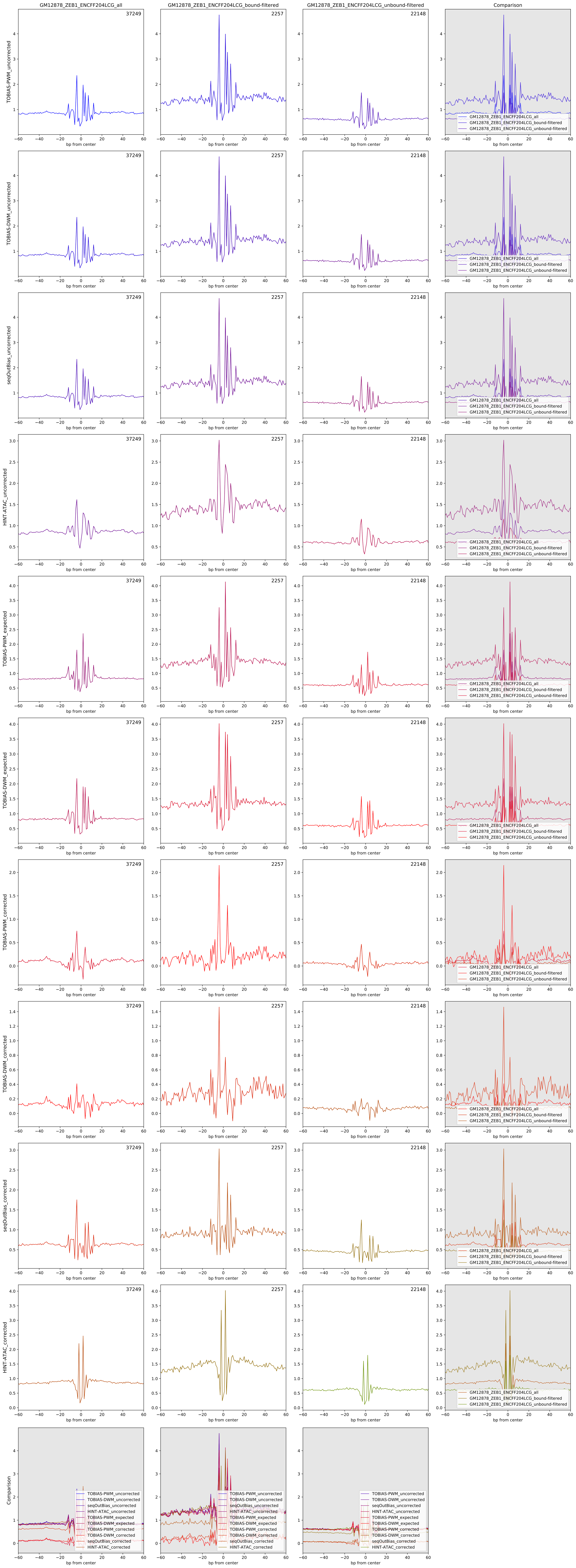

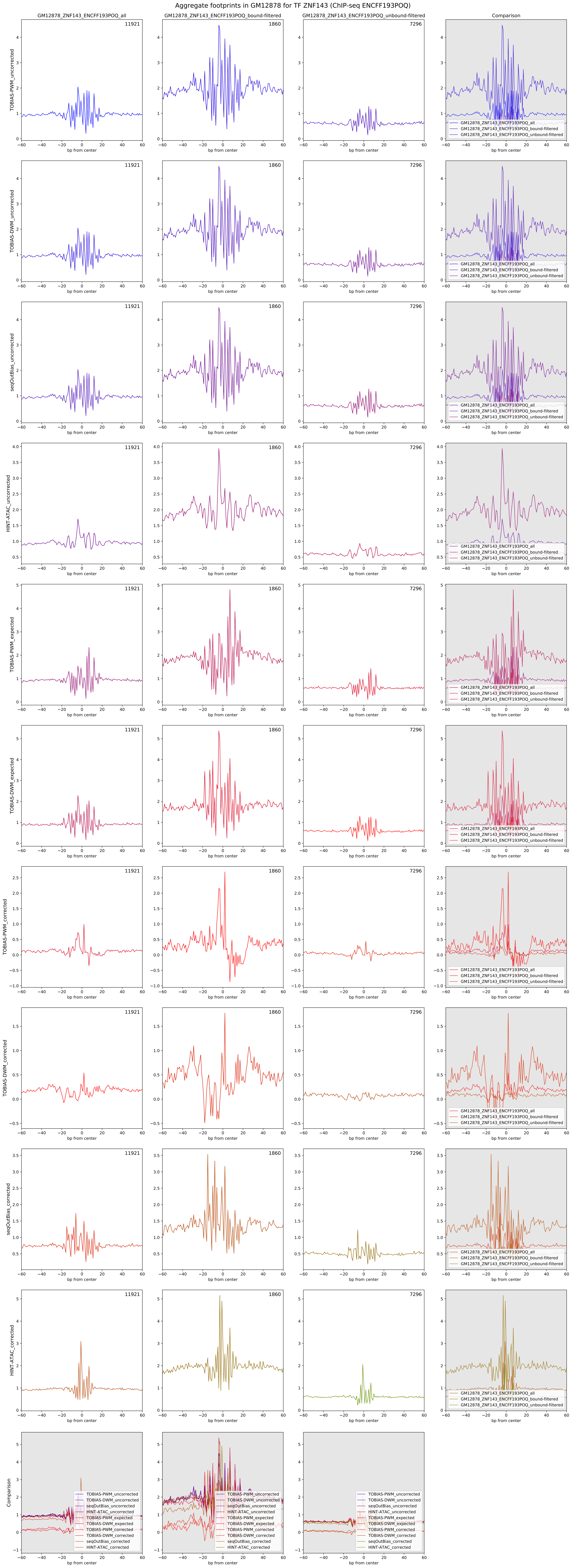

Aggregate footprints in GM12878 for TF ZNF143 (ChIP-seq ENCFF193POQ)
GM12878_ZNF143_ENCFF193POQ_all
GM12878_ZNF143_ENCFF193POQ_bound-filtered
GM12878_ZNF143_ENCFF193POQ_unbound-filtered
Comparison
TOBIAS-PWM_uncorrected
TOBIAS-DWM_uncorrected
seqOutBias_uncorrected
HINT-ATAC_uncorrected
TOBIAS-PWM_expected
TOBIAS-DWM_expected
TOBIAS-PWM_corrected
TOBIAS-DWM_corrected
seqOutBias_corrected
HINT-ATAC_corrected
11921
1860
7296
bp from center

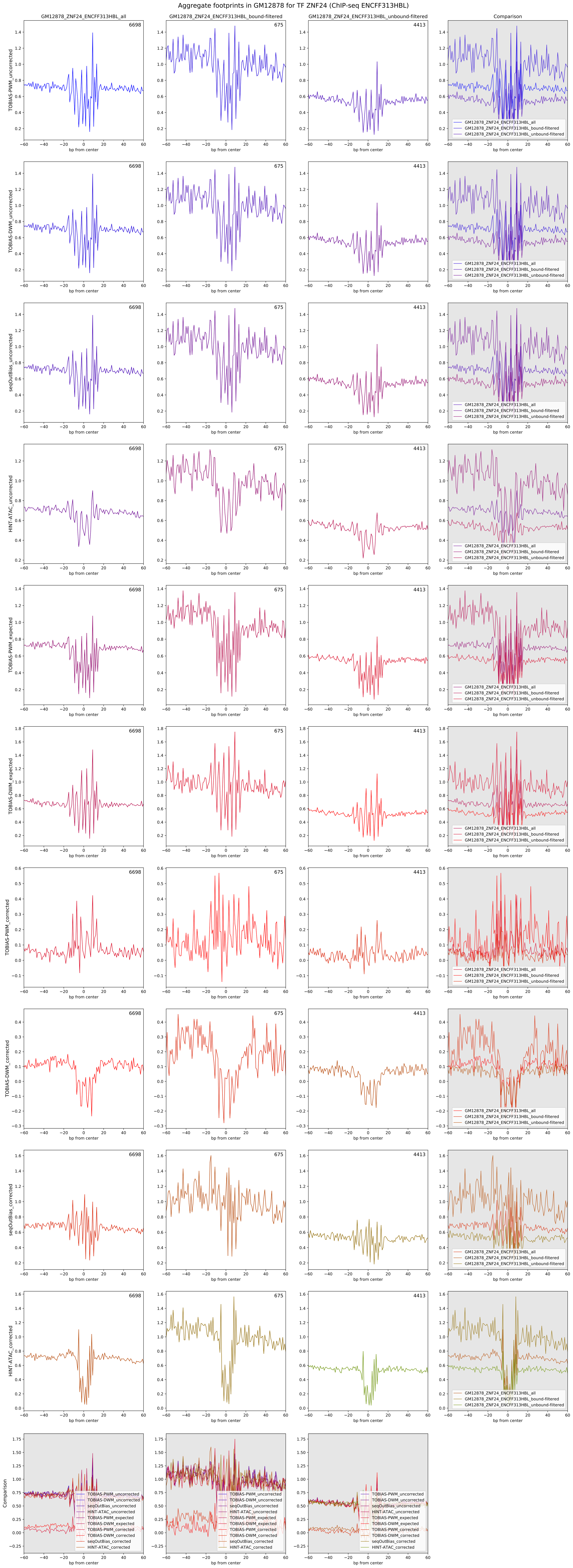
Aggregate footprints in GM12878 for TF ZNF24 (ChIP-seq ENCFF313HBL)
GM12878_ZNF24_ENCFF313HBL_all
GM12878_ZNF24_ENCFF313HBL_bound-filtered
GM12878_ZNF24_ENCFF313HBL_unbound-filtered
Comparison
TOBIAS-PWM_uncorrected
TOBIAS-DWM_uncorrected
seqOutBias_uncorrected
HINT-ATAC_uncorrected
TOBIAS-PWM_expected
TOBIAS-DWM_expected
TOBIAS-PWM_corrected
TOBIAS-DWM_corrected
seqOutBias_corrected
HINT-ATAC_corrected
6698
675
4413
bp from center

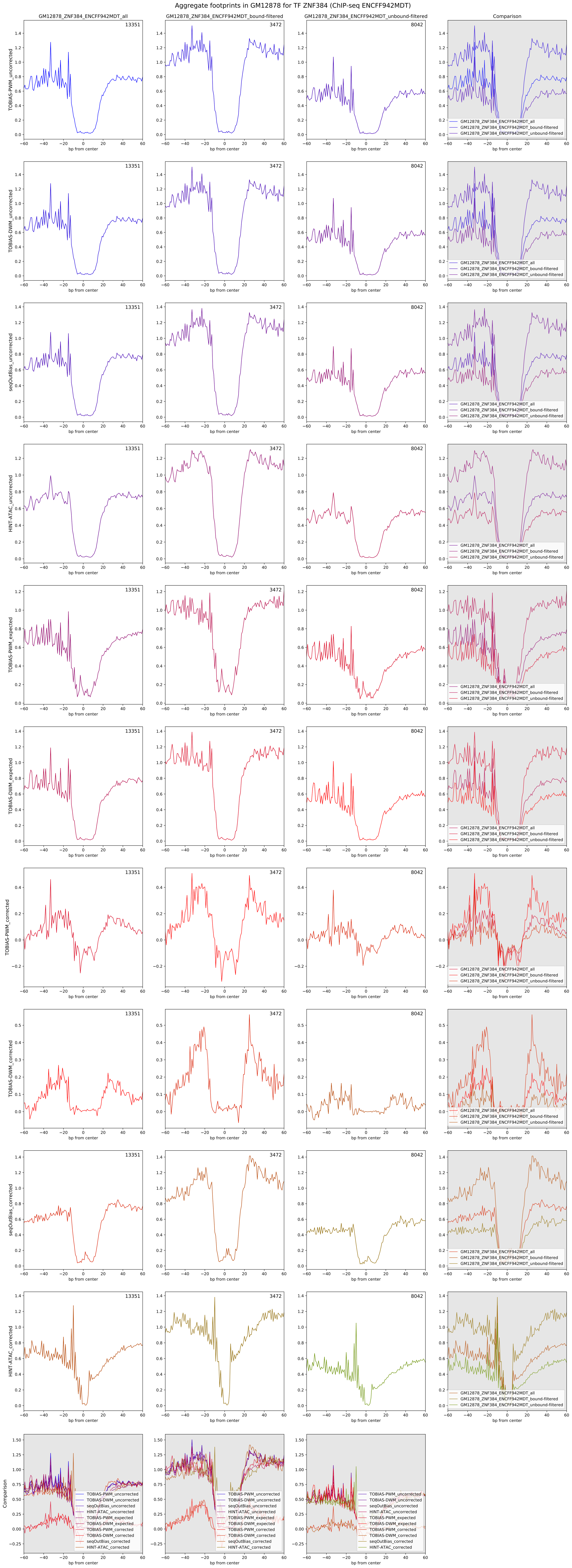
Aggregate footprints in GM12878 for TF ZNF384 (ChIP-seq ENCFF942MDT)
GM12878_ZNF384_ENCFF942MDT_all
GM12878_ZNF384_ENCFF942MDT_bound-filtered
GM12878_ZNF384_ENCFF942MDT_unbound-filtered
Comparison
TOBIAS-PWM_uncorrected
TOBIAS-DWM_uncorrected
seqOutBias_uncorrected
HINT-ATAC_uncorrected
TOBIAS-PWM_expected
TOBIAS-DWM_expected
TOBIAS-PWM_corrected
TOBIAS-DWM_corrected
seqOutBias_corrected
HINT-ATAC_corrected
Comparison
13351
3472
8042
bp from center

Aggregate footprints in HepG2 for TF ARID3A (ChIP-seq ENCFF247GXE)

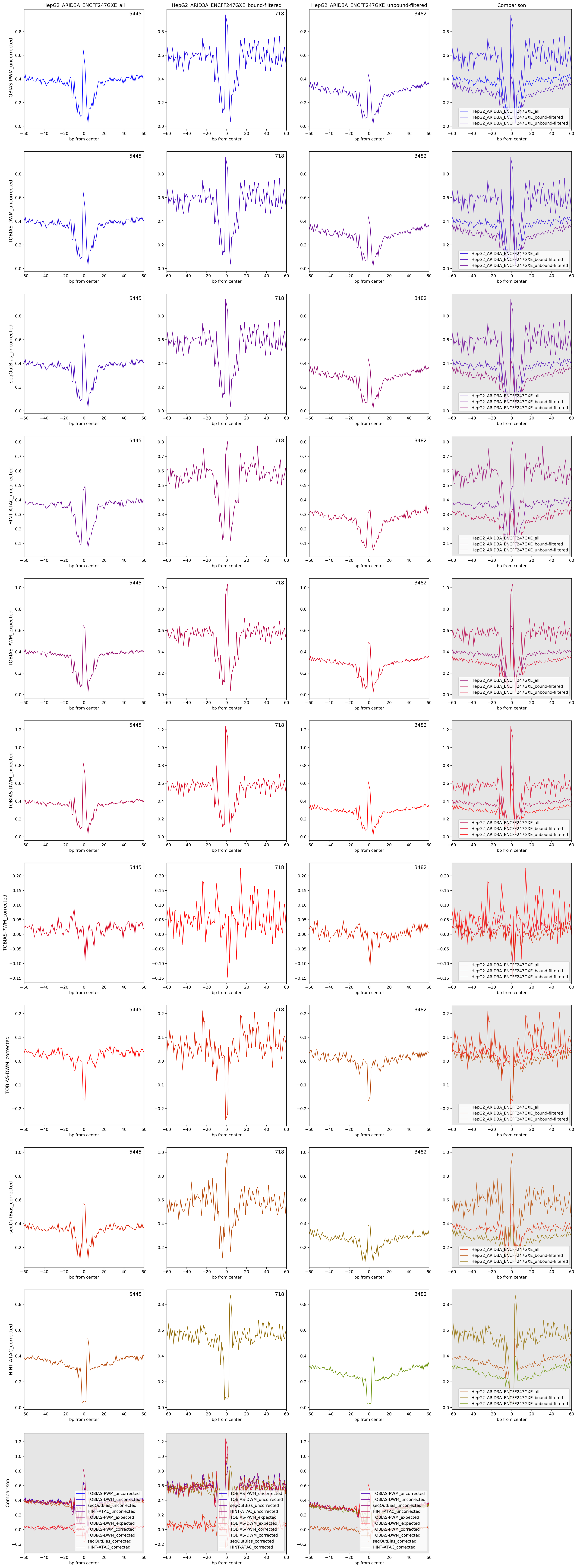

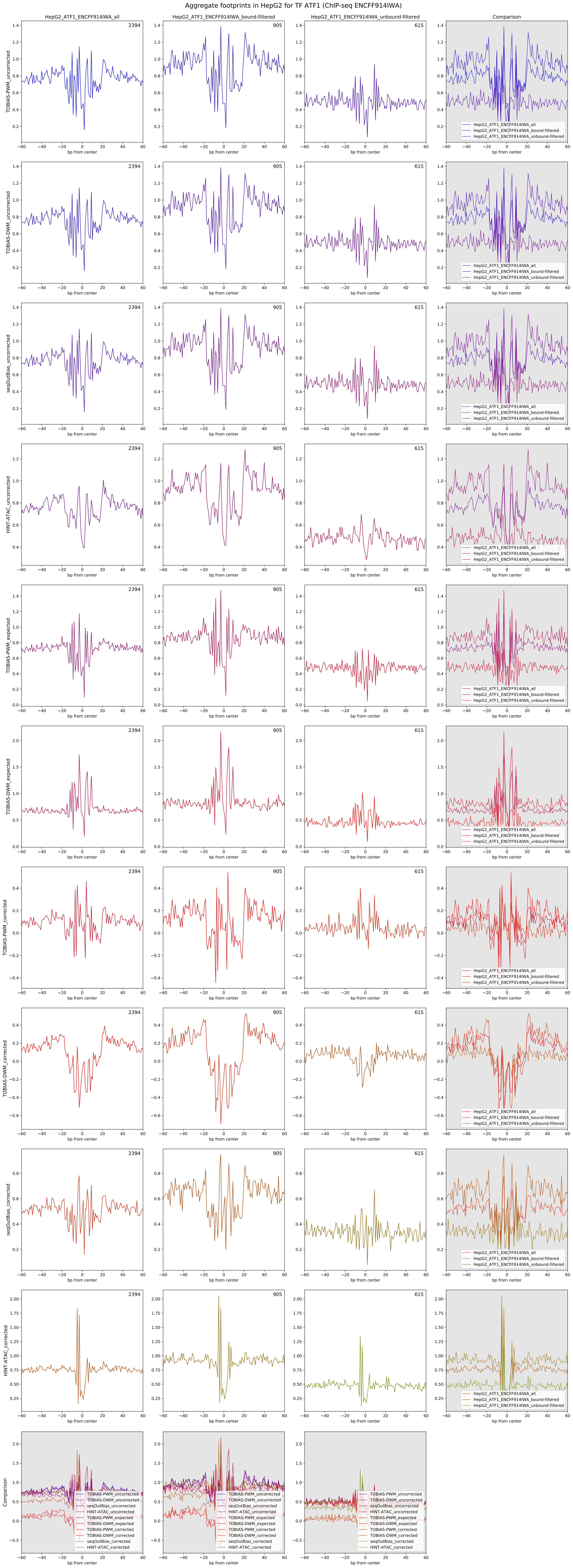

Aggregate footprints in HepG2 for TF ATF1 (ChIP-seq ENCFF914IWA)
HepG2_ATF1_ENCFF914IWA_all
HepG2_ATF1_ENCFF914IWA_bound-filtered
HepG2_ATF1_ENCFF914IWA_unbound-filtered
Comparison
TOBIAS-PWM_uncorrected
TOBIAS-DWM_uncorrected
seqOutBias_uncorrected
HINT-ATAC_uncorrected
TOBIAS-PWM_expected
TOBIAS-DWM_expected
TOBIAS-PWM_corrected
TOBIAS-DWM_corrected
seqOutBias_corrected
HINT-ATAC_corrected
2394
905
615
bp from center

Aggregate footprints in HepG2 for TF ATF3 (ChIP-seq ENCFF137OEY)

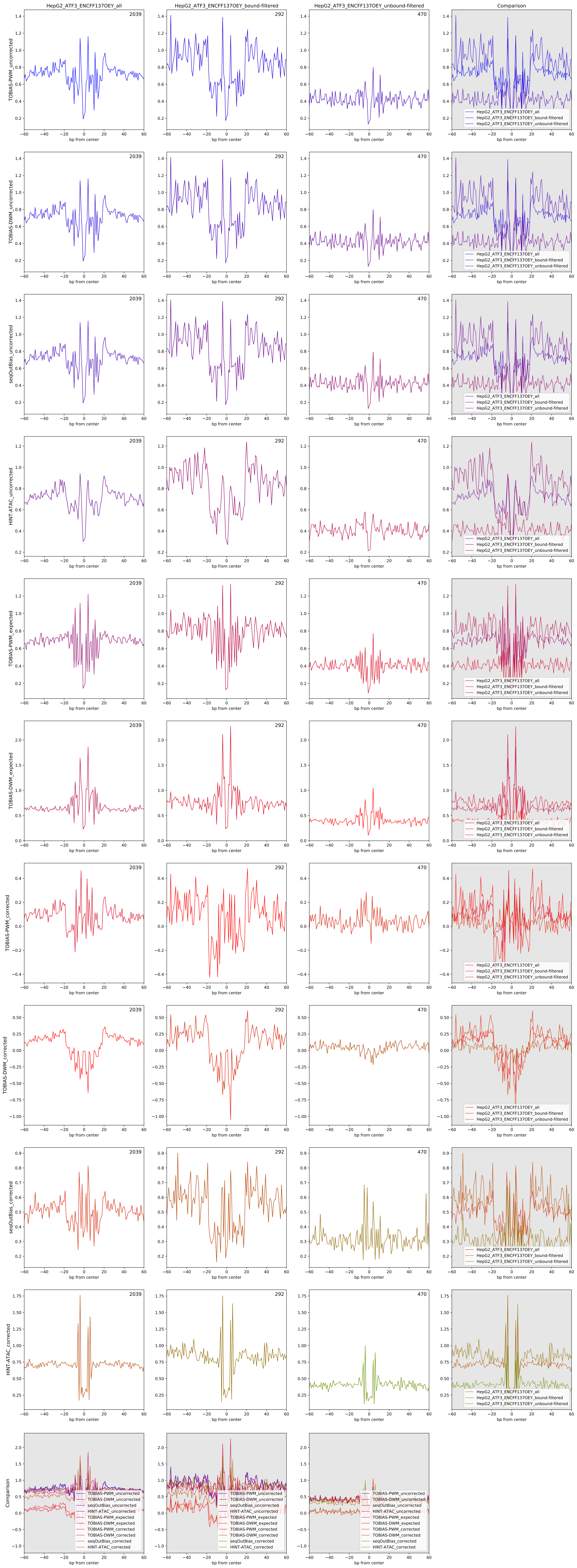

Aggregate footprints in HepG2 for TF BCL6 (ChIP-seq ENCFF014UTU)

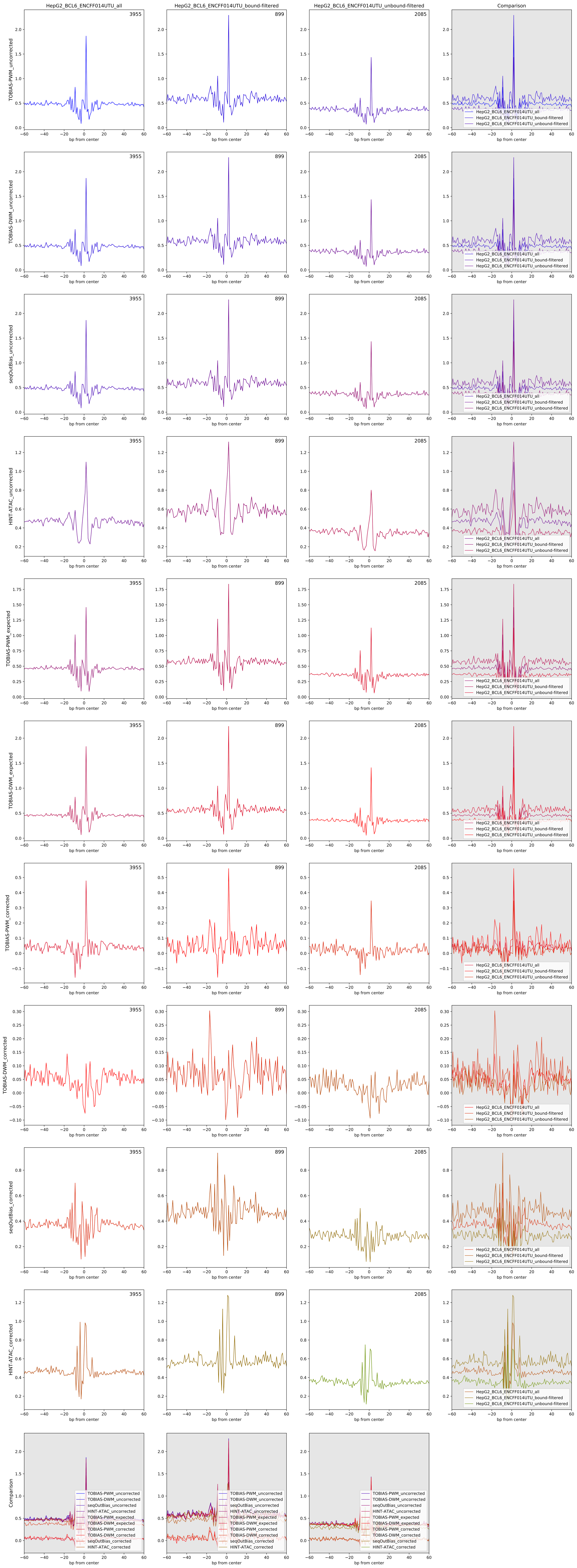

Aggregate footprints in HepG2 for TF BHLHE40 (ChIP-seq ENCFF863ATX)

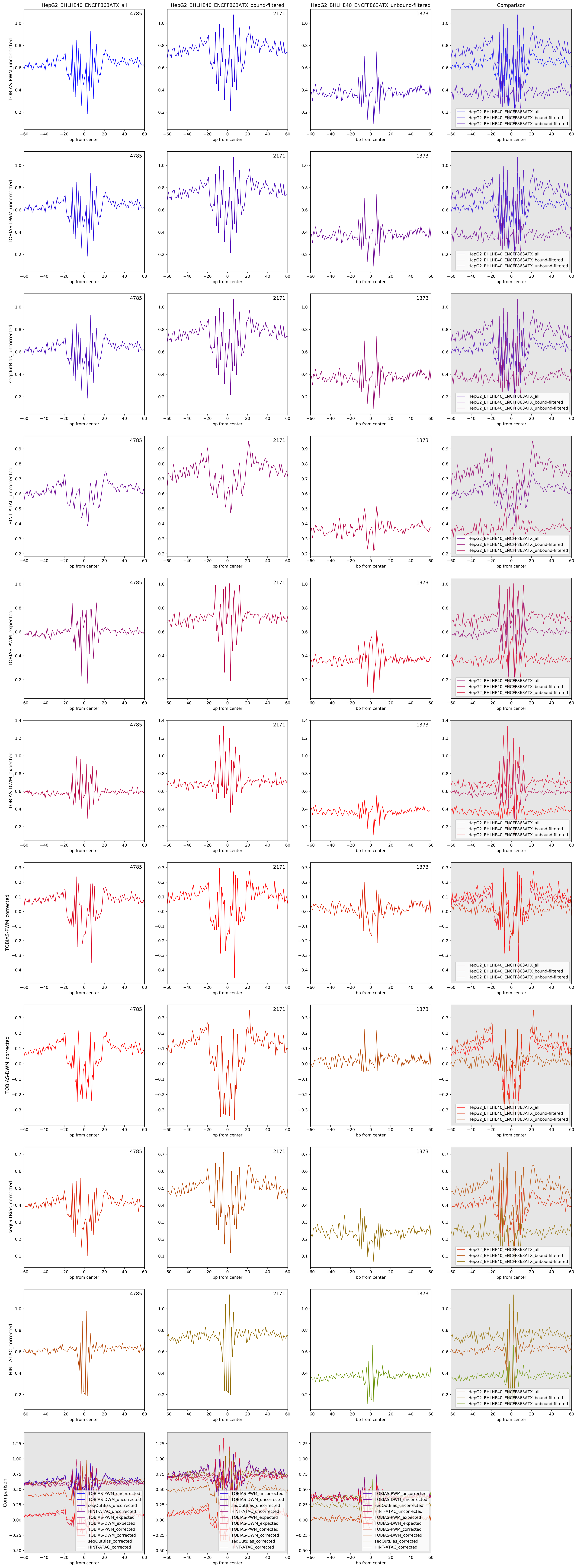

Aggregate footprints in HepG2 for TF CEBPA (ChIP-seq ENCFF874PIO)

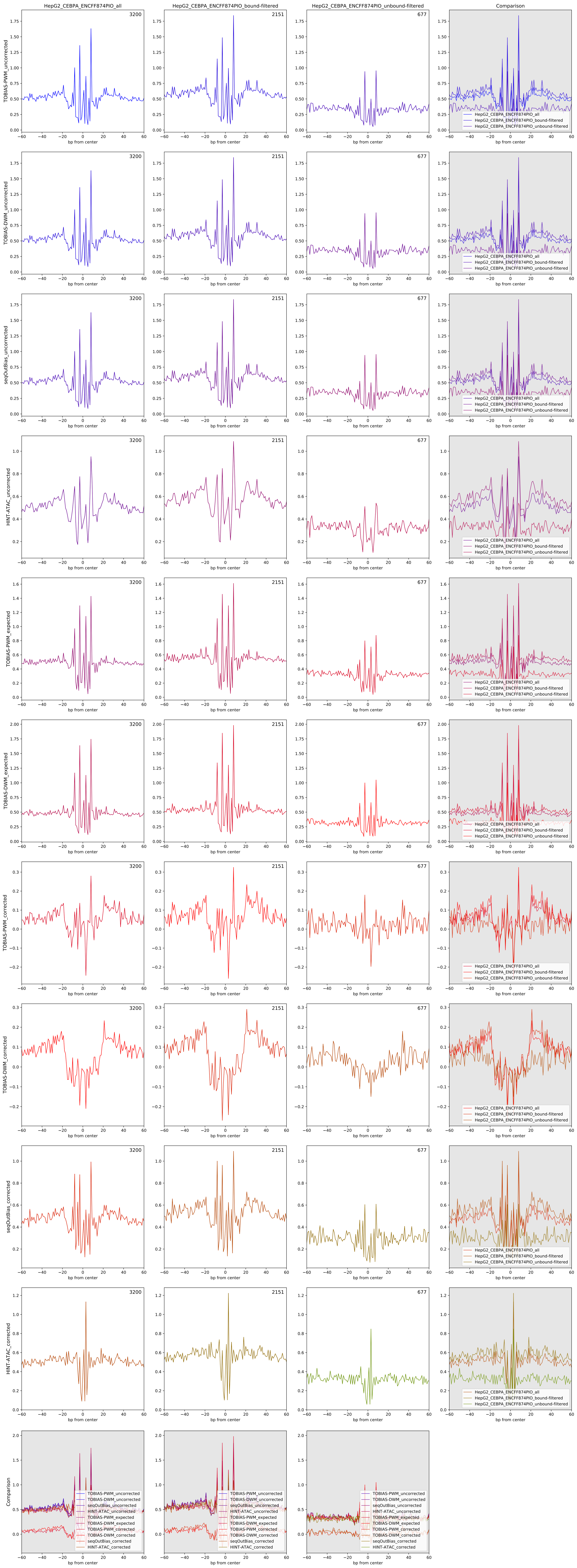

Aggregate footprints in HepG2 for TF CEBPB (ChIP-seq ENCFF915ZYE)

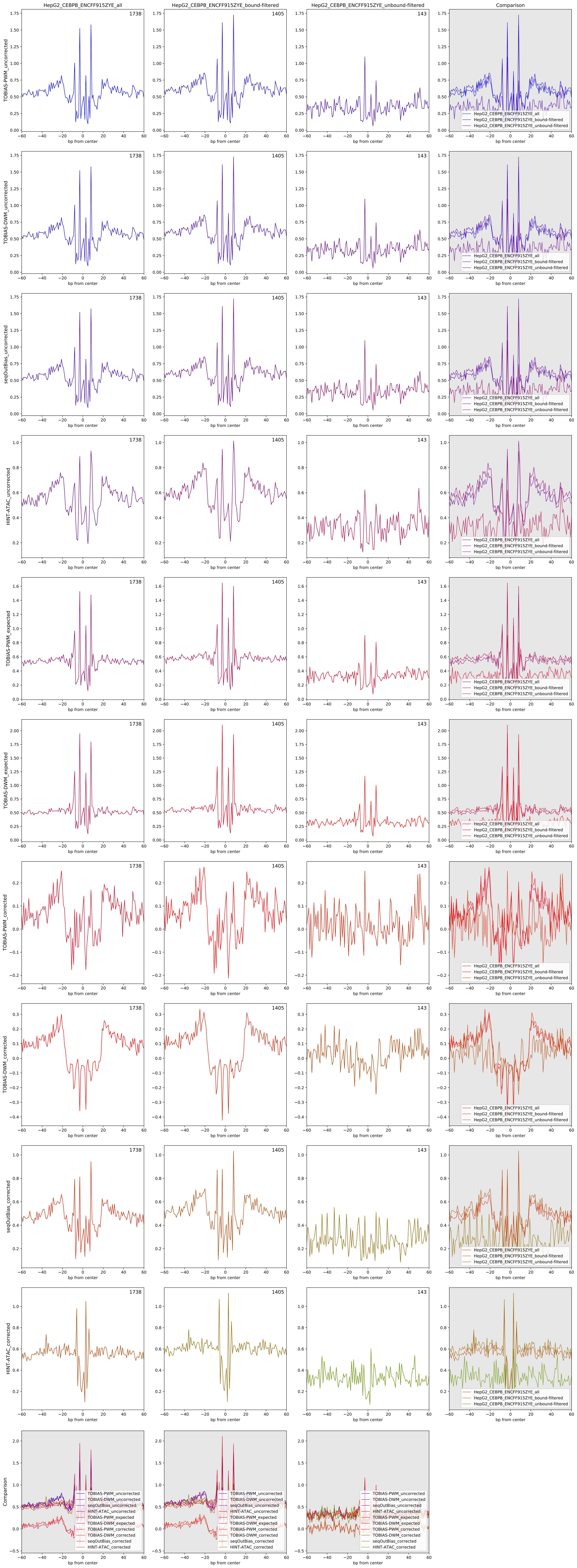

Aggregate footprints in HepG2 for TF CEBPG (ChIP-seq ENCFF094SJQ)

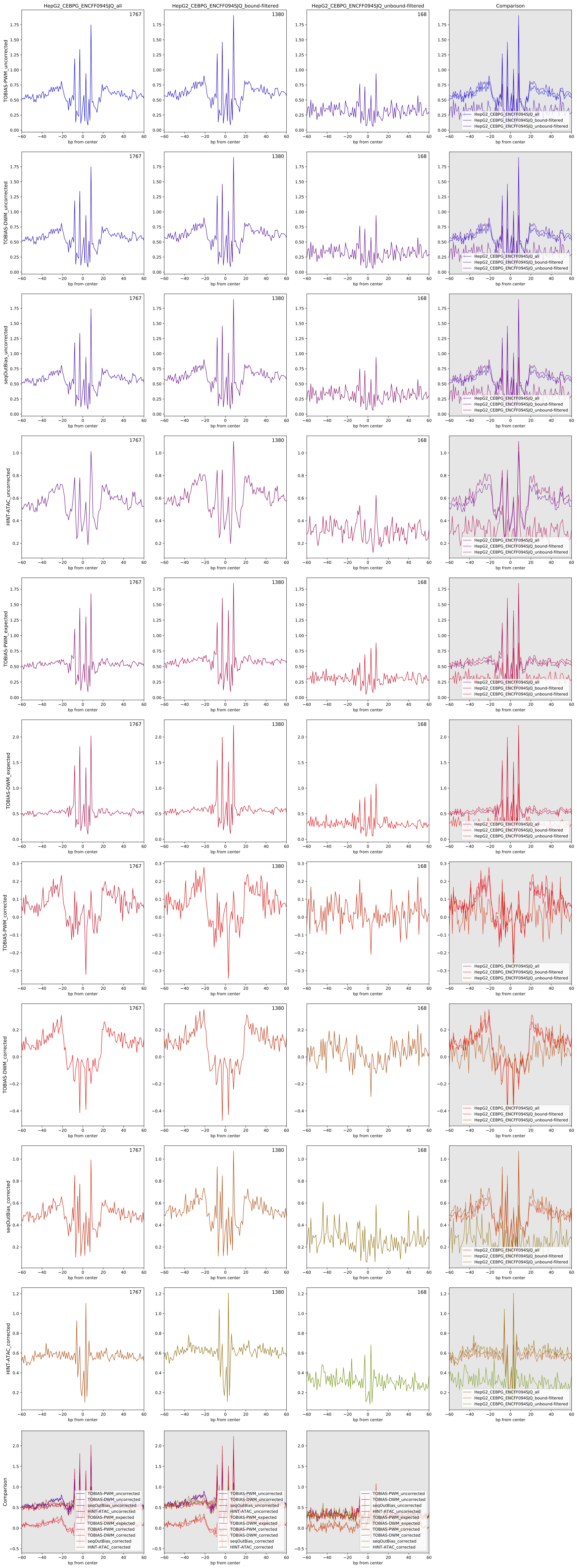

Aggregate footprints in HepG2 for TF CREB1 (ChIP-seq ENCFF550TXR)

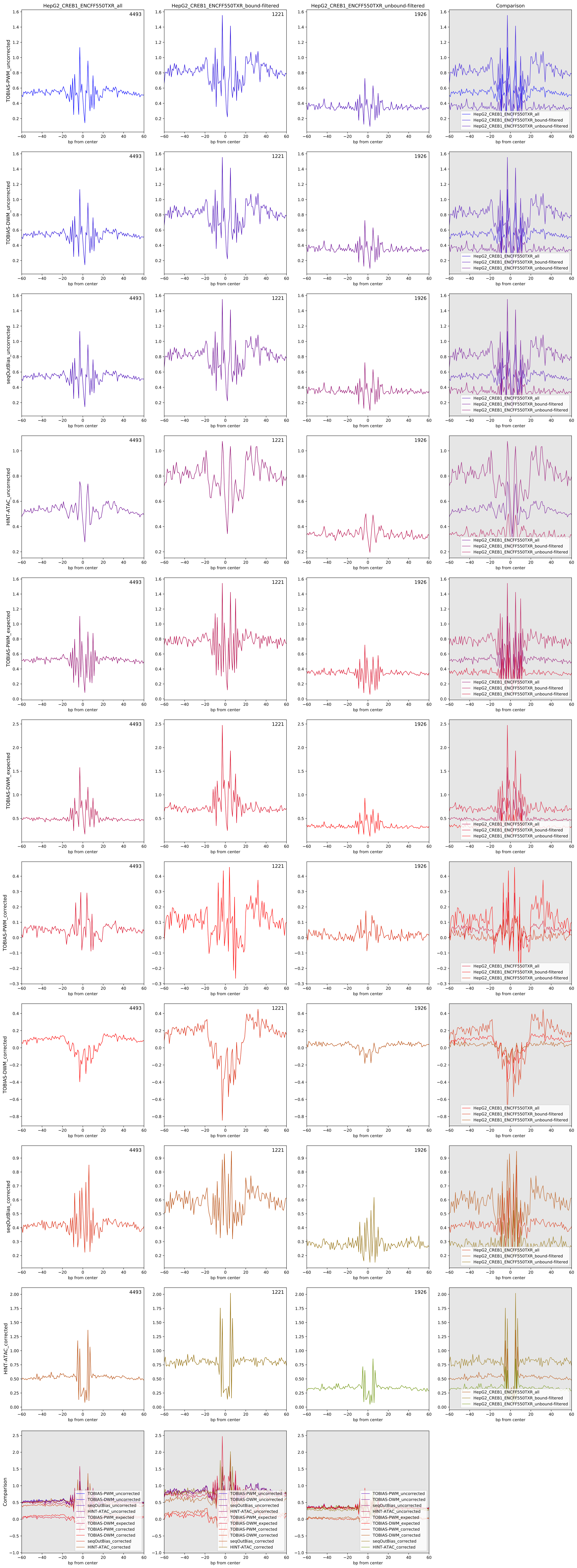

Aggregate footprints in HepG2 for TF CREM (ChIP-seq ENCFF290UGF)

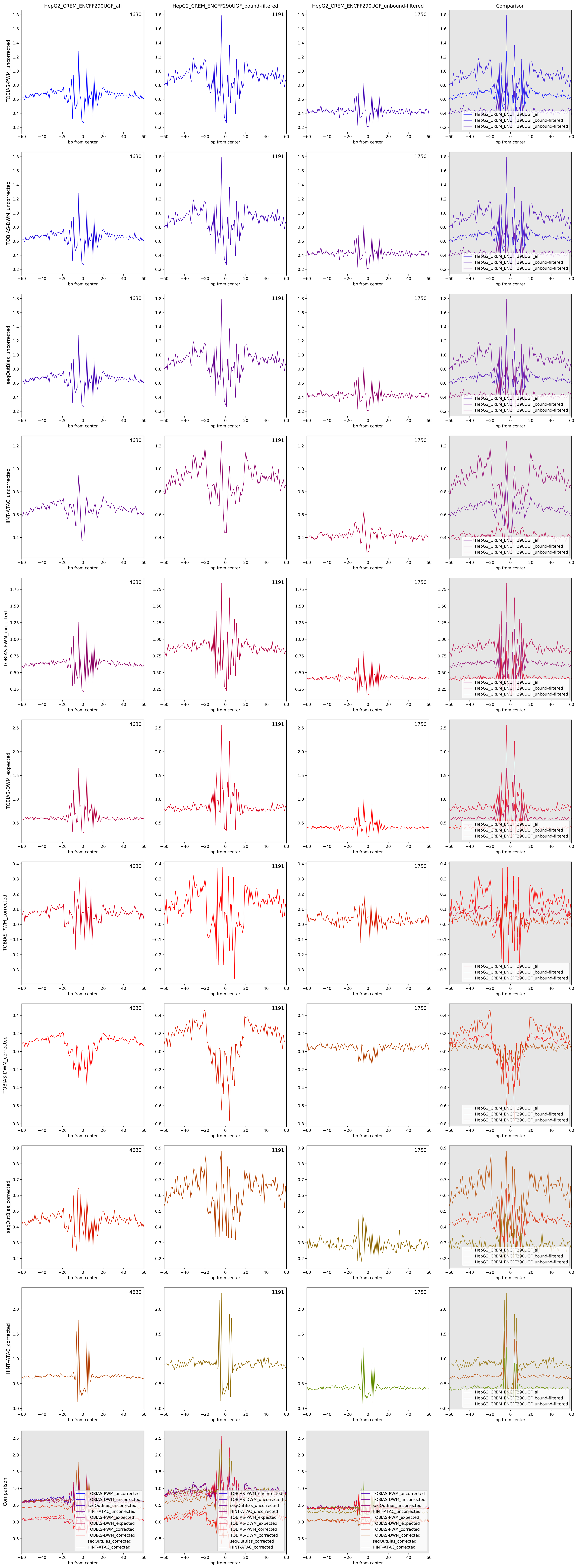

Aggregate footprints in HepG2 for TF CTCF (ChIP-seq ENCFF543WTP)

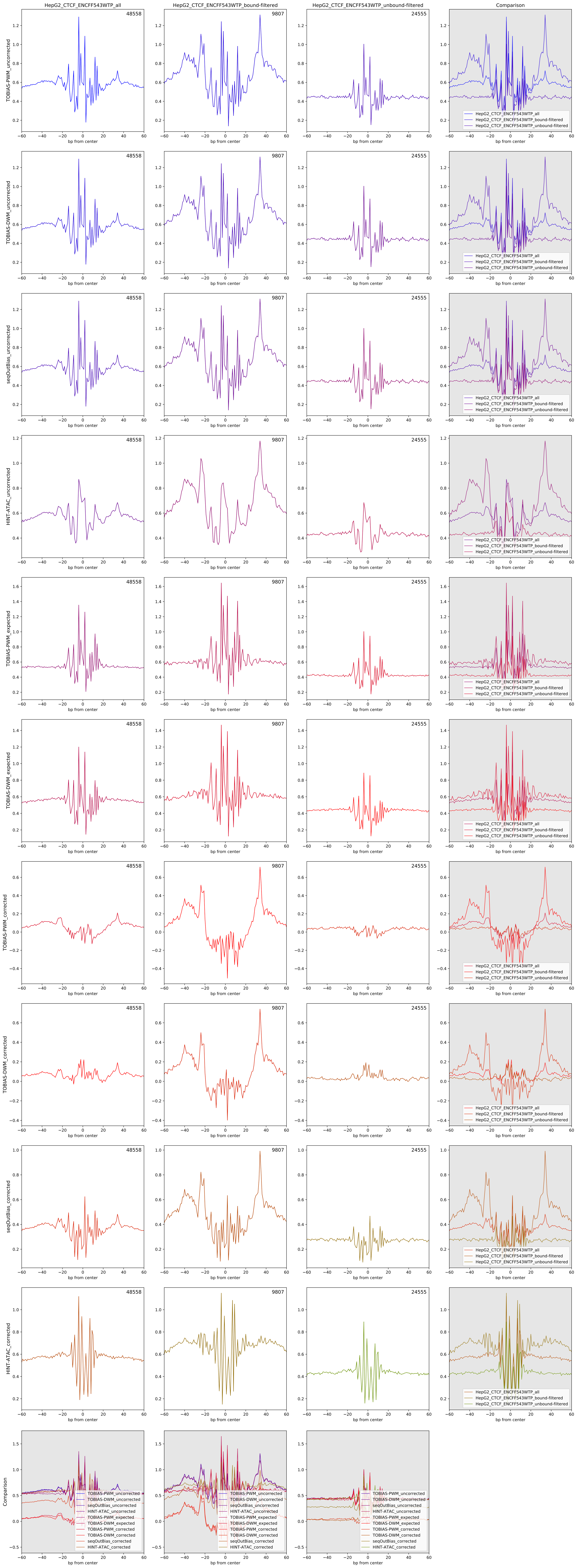

Aggregate footprints in HepG2 for TF ELF1 (ChIP-seq ENCFF840RWO)

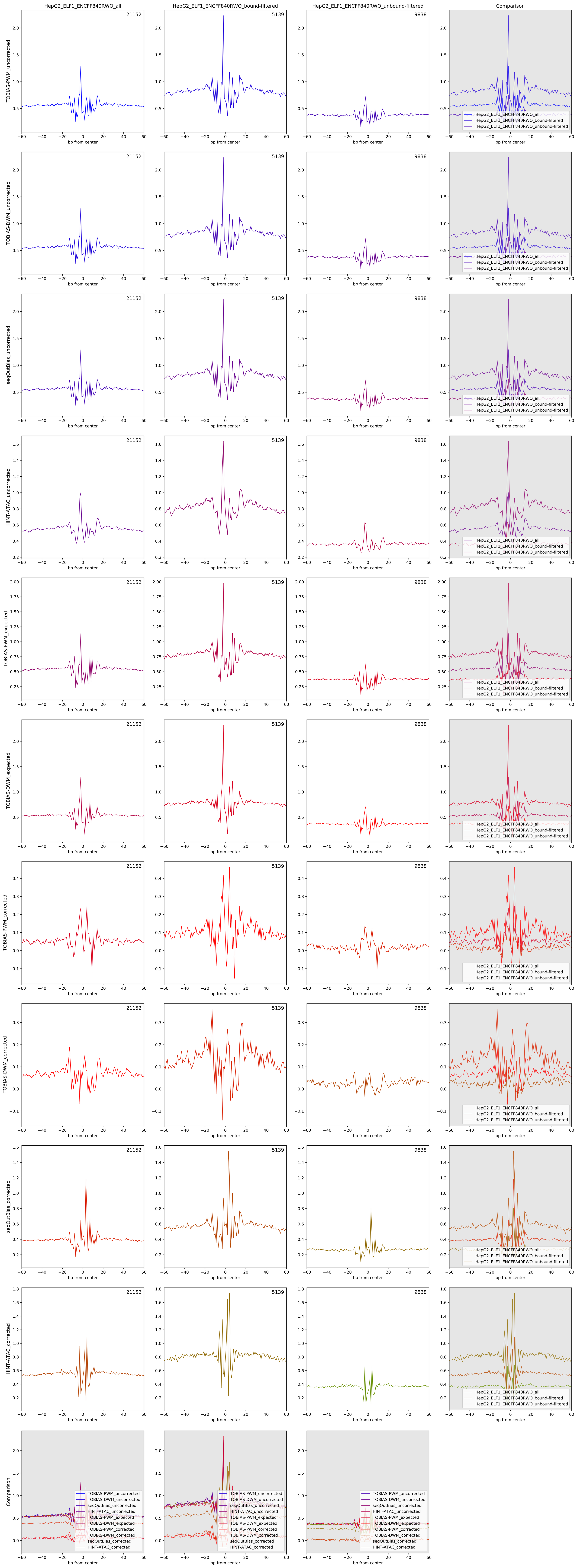

Aggregate footprints in HepG2 for TF ETV4 (ChIP-seq ENCFF710CRT)

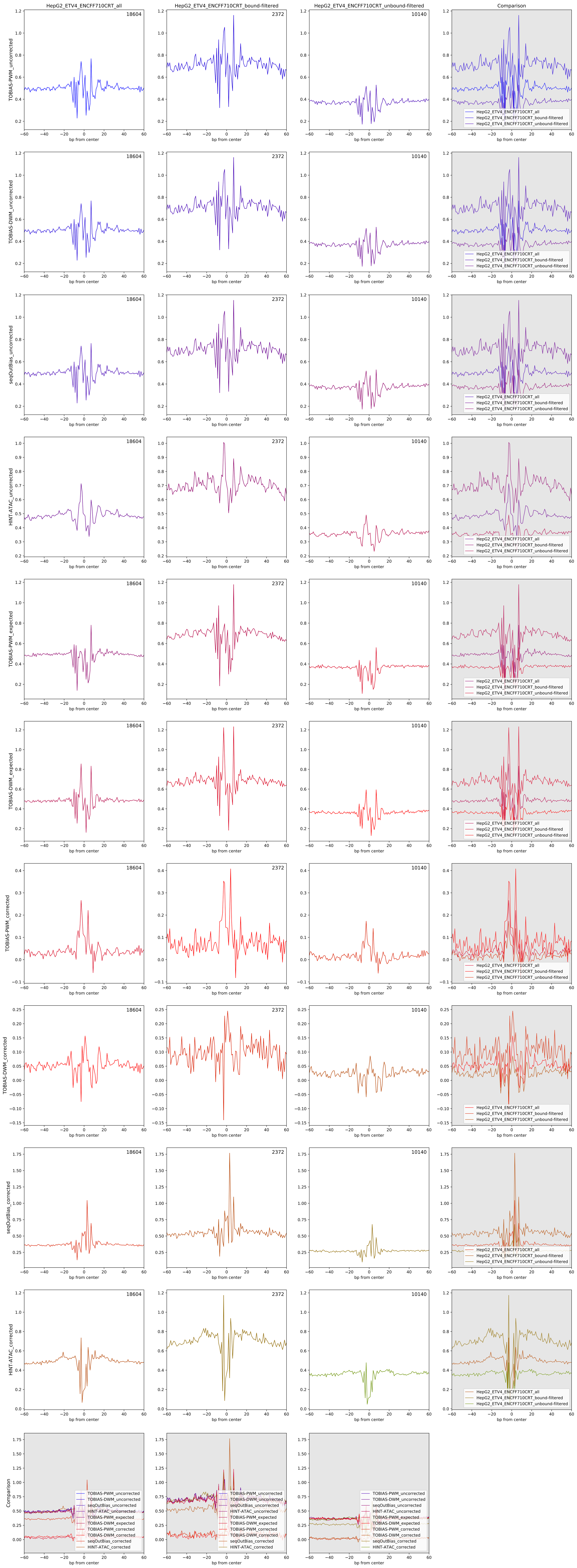

Aggregate footprints in HepG2 for TF ETV5 (ChIP-seq ENCFF923NCW)

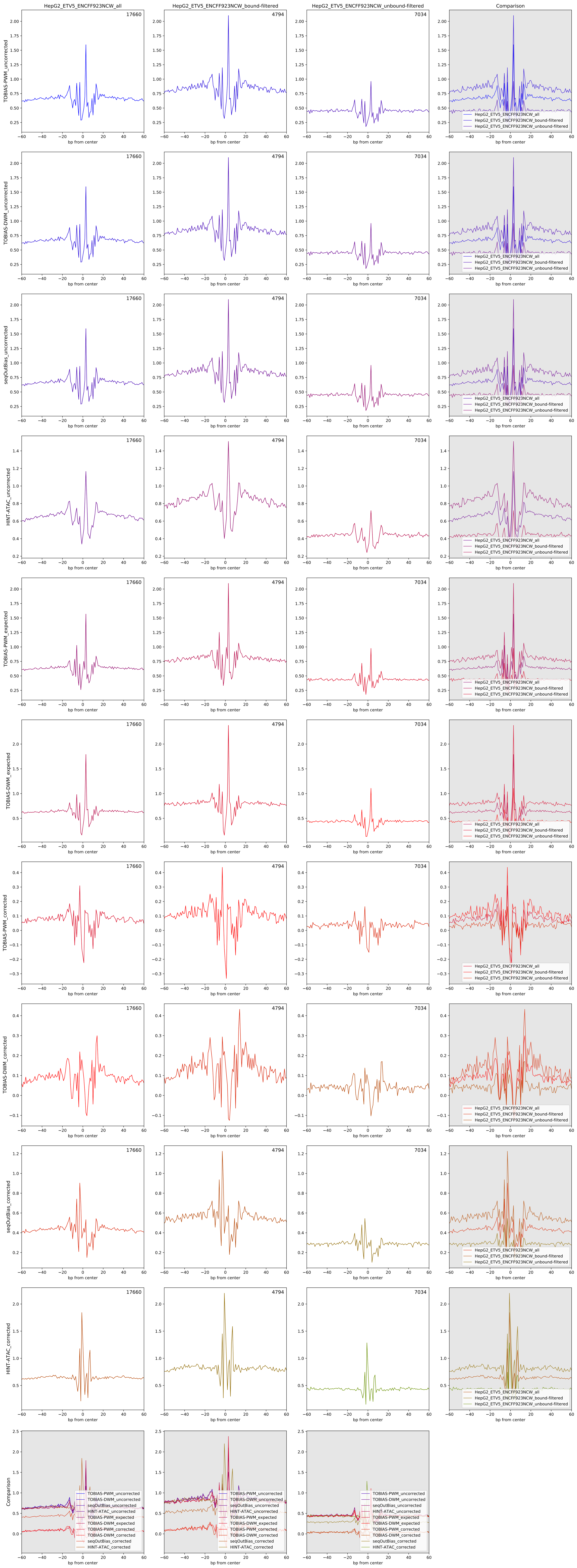

Aggregate footprints in HepG2 for TF FOSL2 (ChIP-seq ENCFF054ESU)

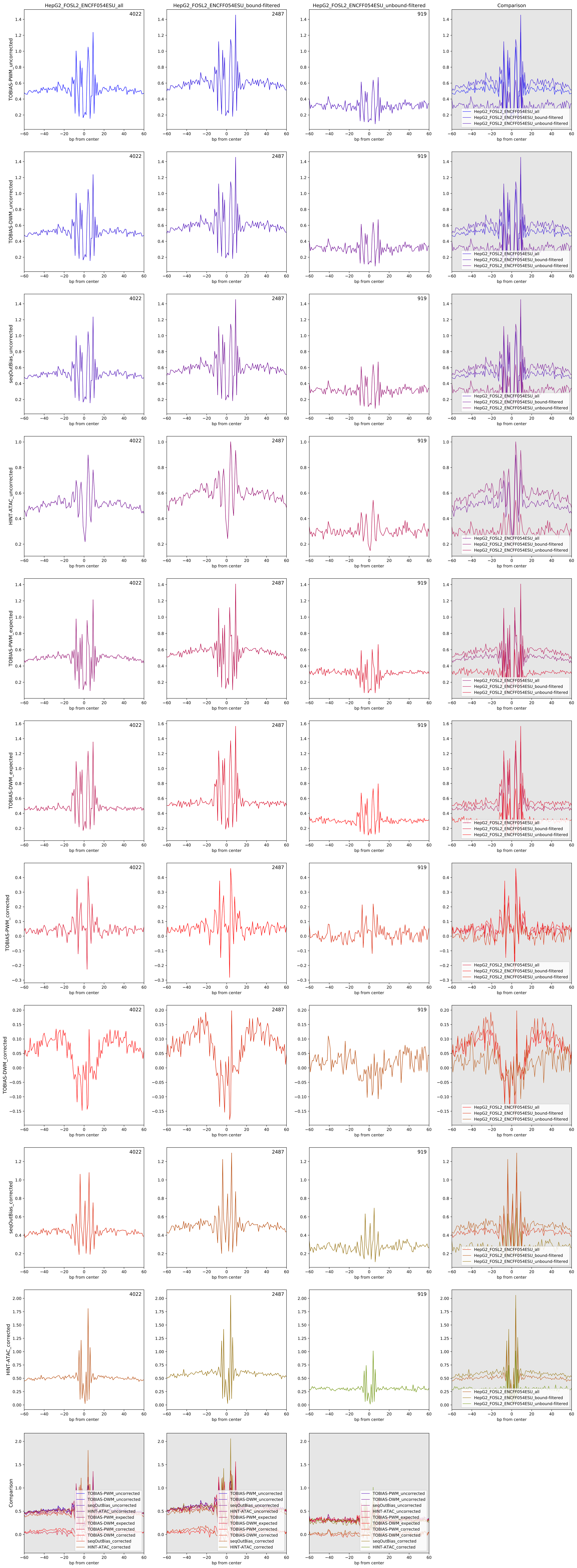

Aggregate footprints in HepG2 for TF FOXA1 (ChIP-seq ENCFF872MGU)

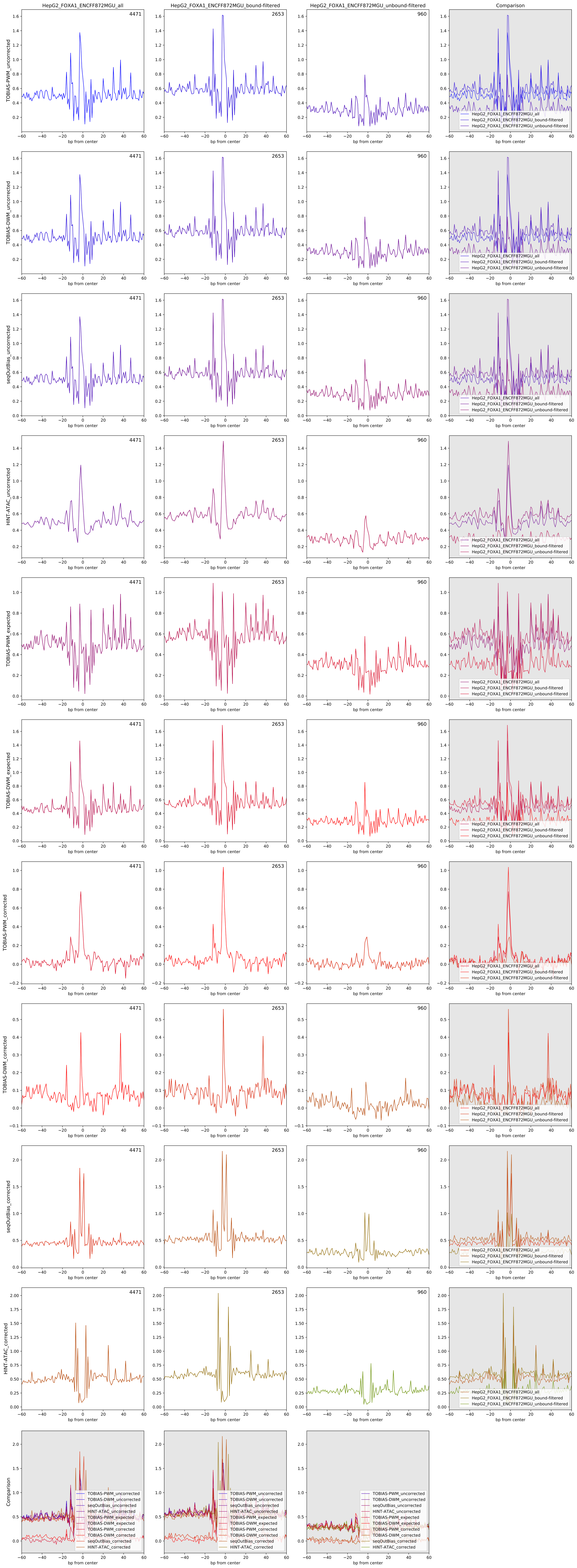

Aggregate footprints in HepG2 for TF FOXA2 (ChIP-seq ENCFF259BJR)

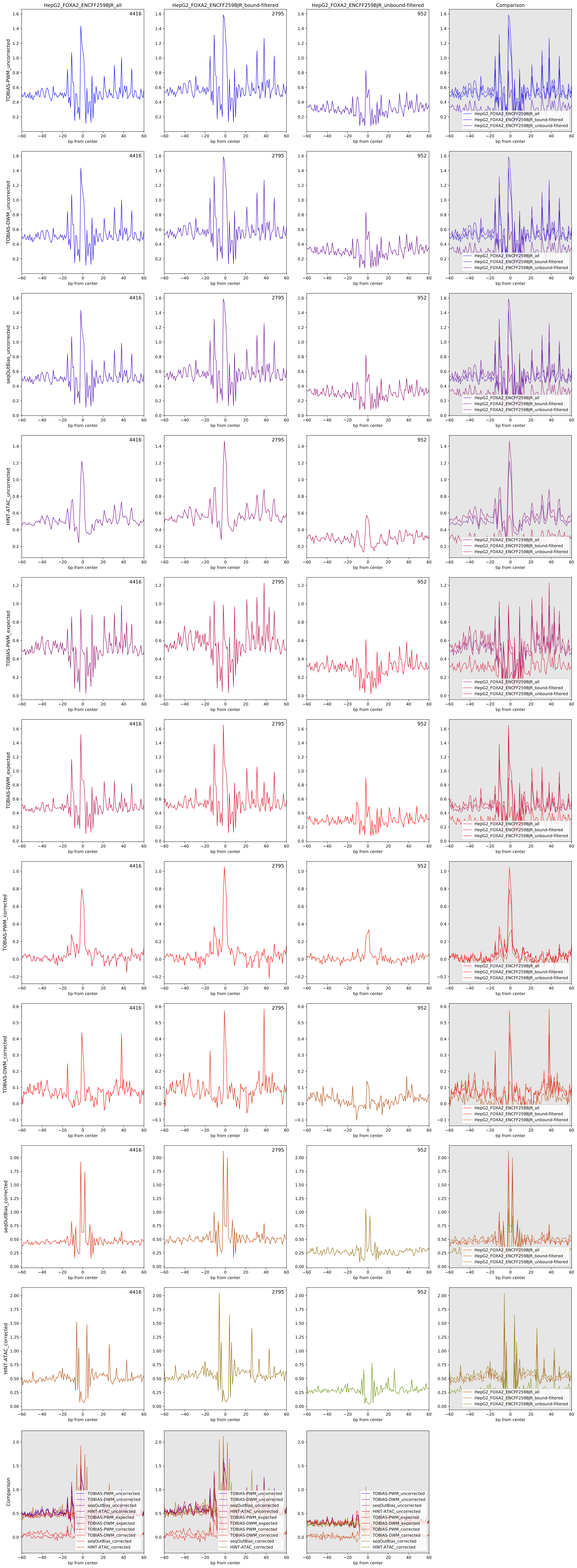

Aggregate footprints in HepG2 for TF FOXK2 (ChIP-seq ENCFF315CHX)

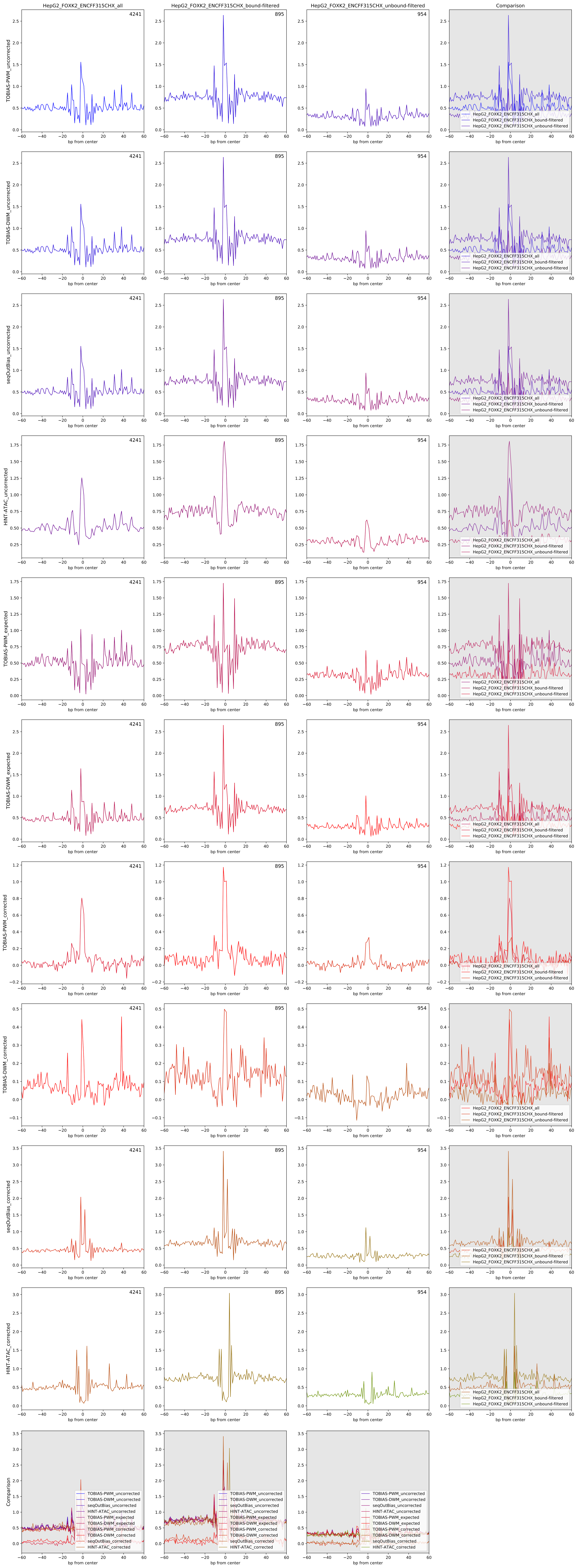

Aggregate footprints in HepG2 for TF GABPA (ChIP-seq ENCFF613KAB)

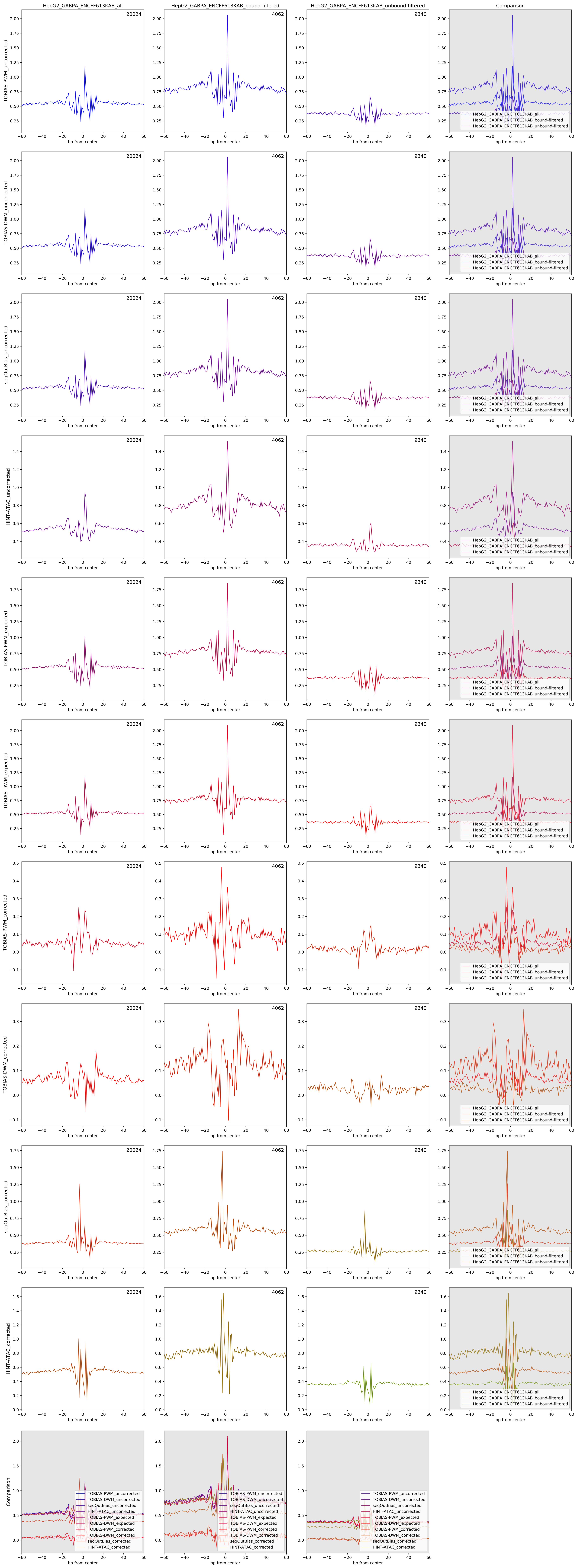

Aggregate footprints in HepG2 for TF GATA4 (ChIP-seq ENCFF097OXR)

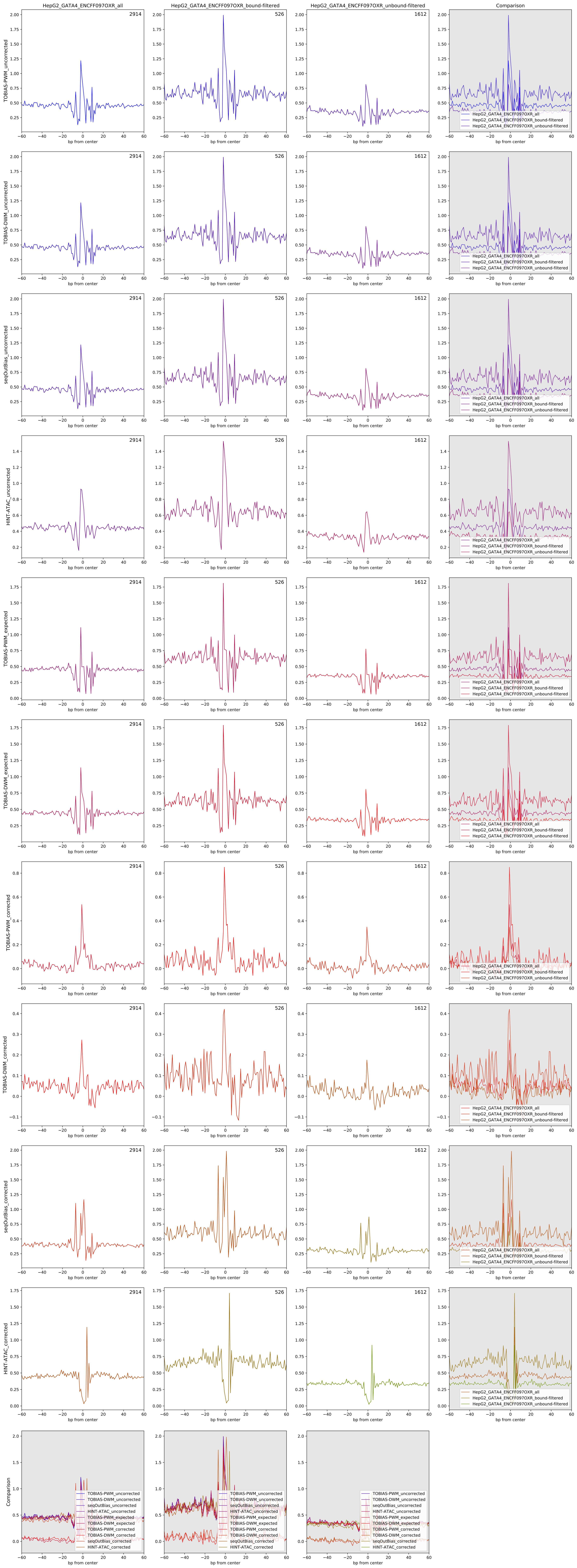

Aggregate footprints in HepG2 for TF GMEB2 (ChIP-seq ENCFF219KQD)

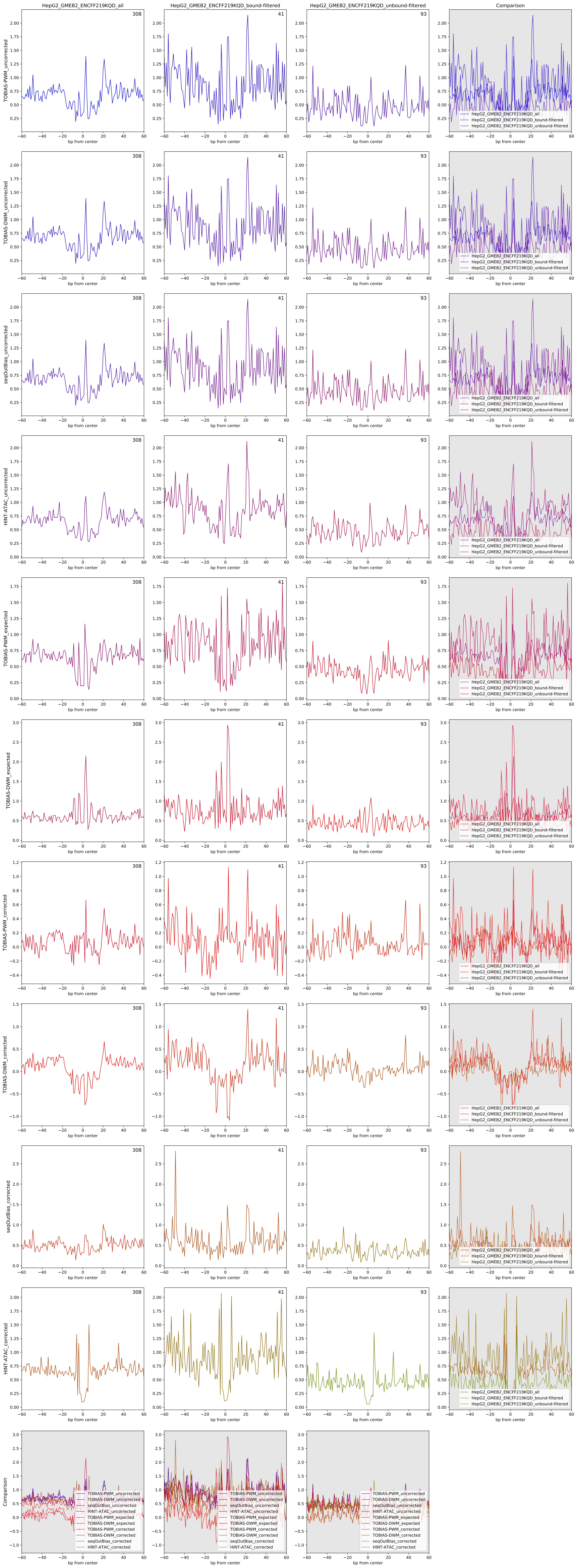

Aggregate footprints in HepG2 for TF HLF (ChIP-seq ENCFF685UQE)

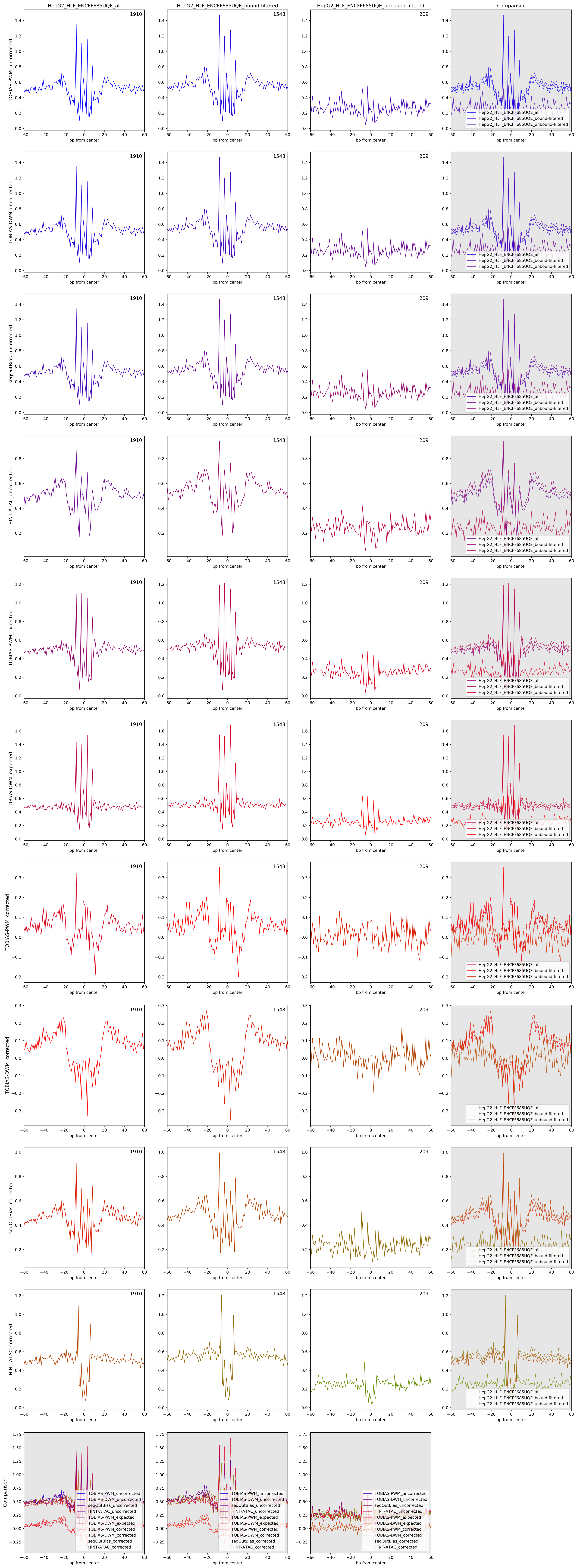

Aggregate footprints in HepG2 for TF HNF1A (ChIP-seq ENCFF800QTO)

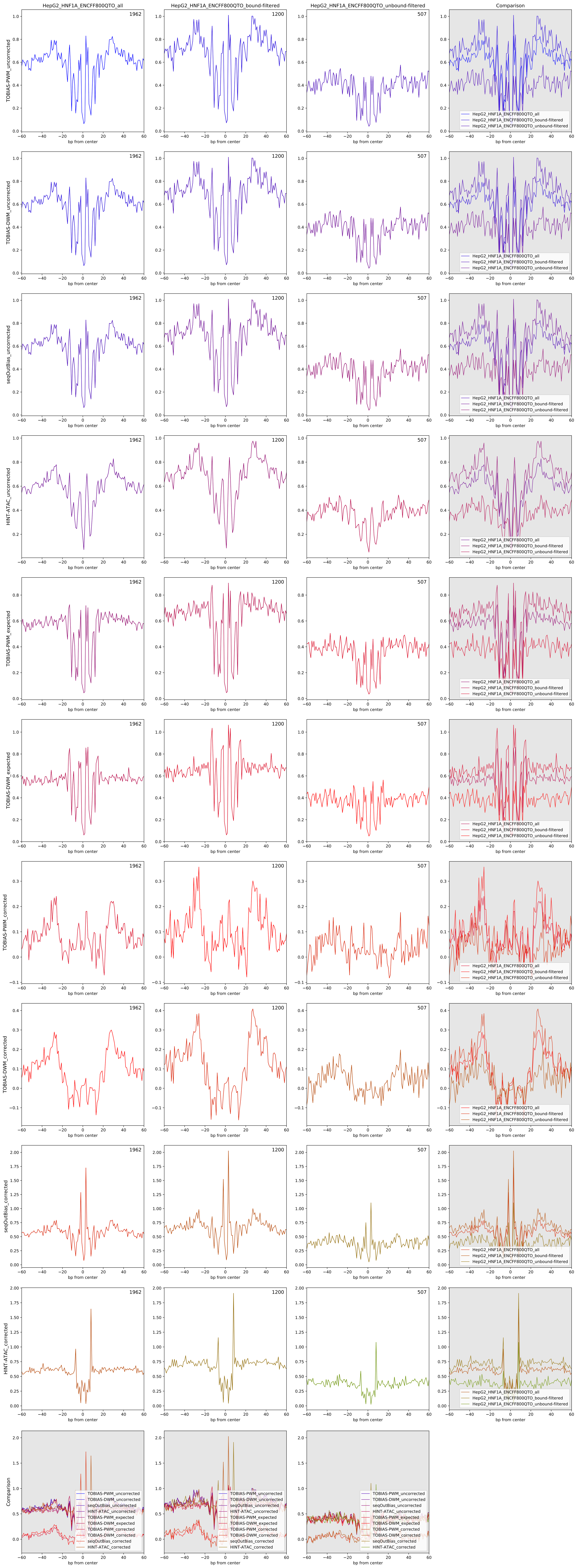

Aggregate footprints in HepG2 for TF HNF4A (ChIP-seq ENCFF072CXB)

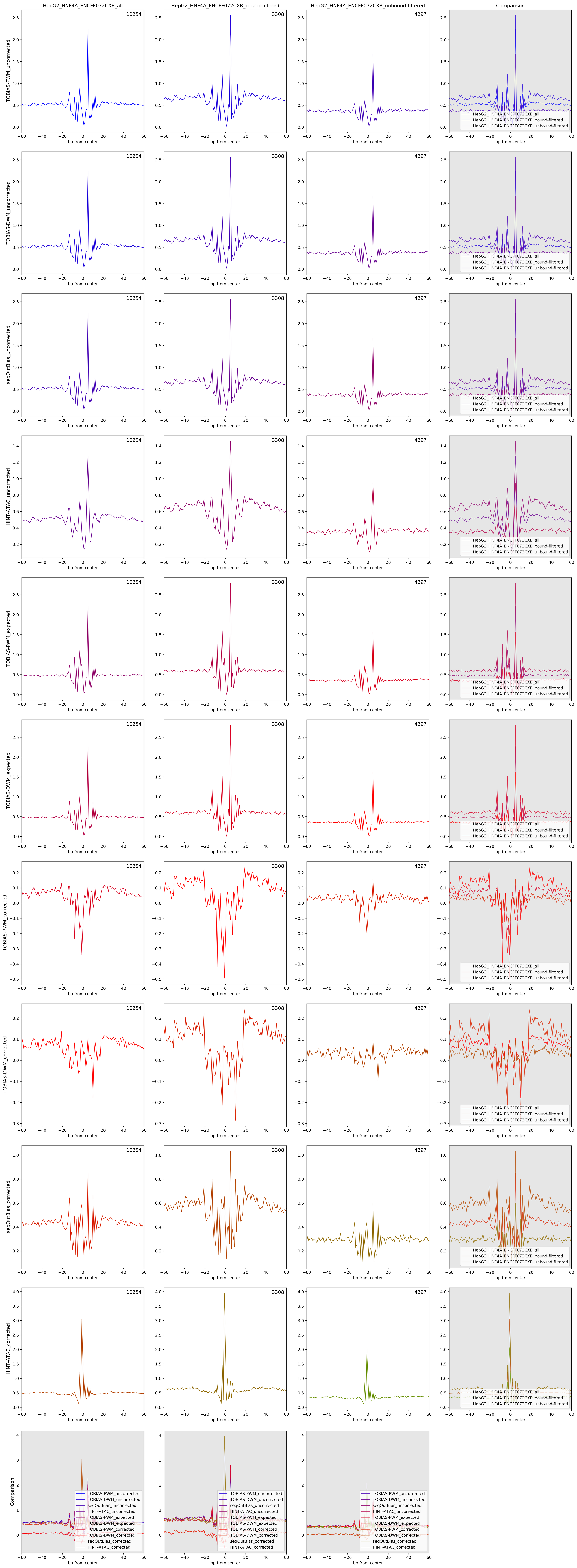

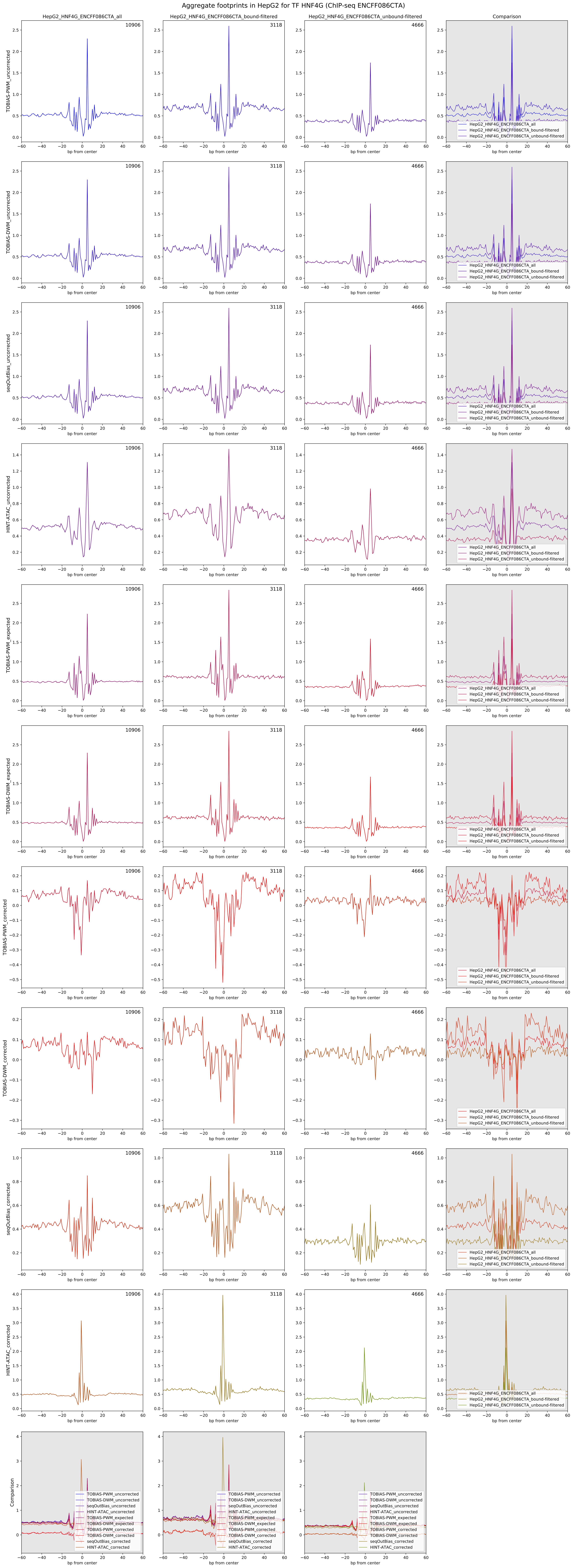

Aggregate footprints in HepG2 for TF HNF4G (ChIP-seq ENCFF086CTA)
HepG2_HNF4G_ENCFF086CTA_all
HepG2_HNF4G_ENCFF086CTA_bound-filtered
HepG2_HNF4G_ENCFF086CTA_unbound-filtered
Comparison
TOBIAS-PWM_uncorrected
TOBIAS-DWM_uncorrected
seqOutBias_uncorrected
HINT-ATAC_uncorrected
TOBIAS-PWM_expected
TOBIAS-DWM_expected
TOBIAS-PWM_corrected
TOBIAS-DWM_corrected
seqOutBias_corrected
HINT-ATAC_corrected
10906
3118
4666
bp from center

Aggregate footprints in HepG2 for TF JUN (ChIP-seq ENCFF331IUK)

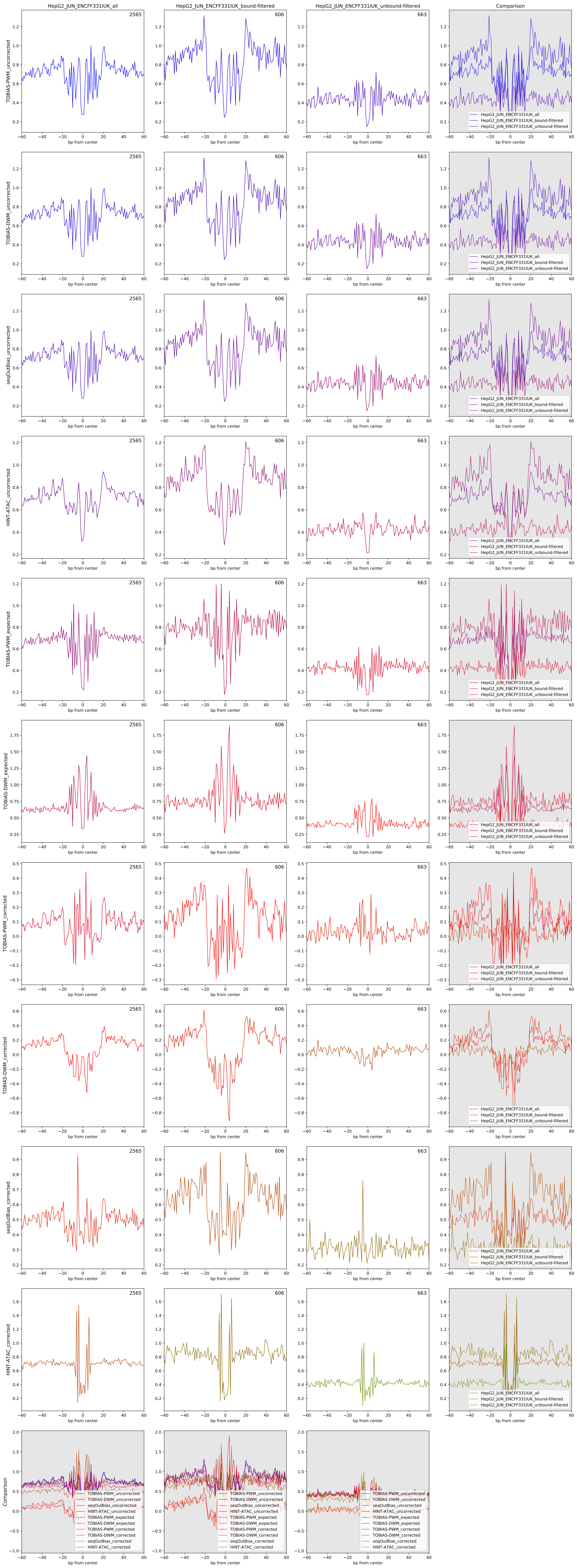

Aggregate footprints in HepG2 for TF JUND (ChIP-seq ENCFF430PEI)

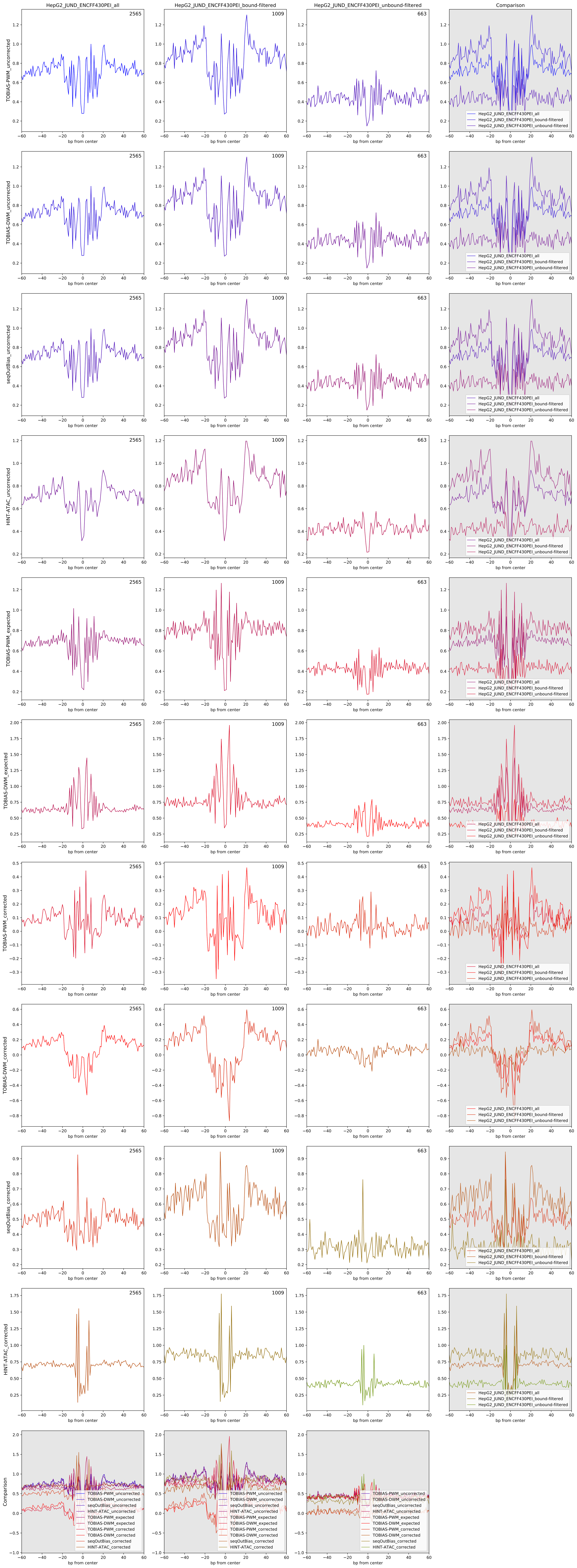

Aggregate footprints in HepG2 for TF MAFF (ChIP-seq ENCFF493TIR)

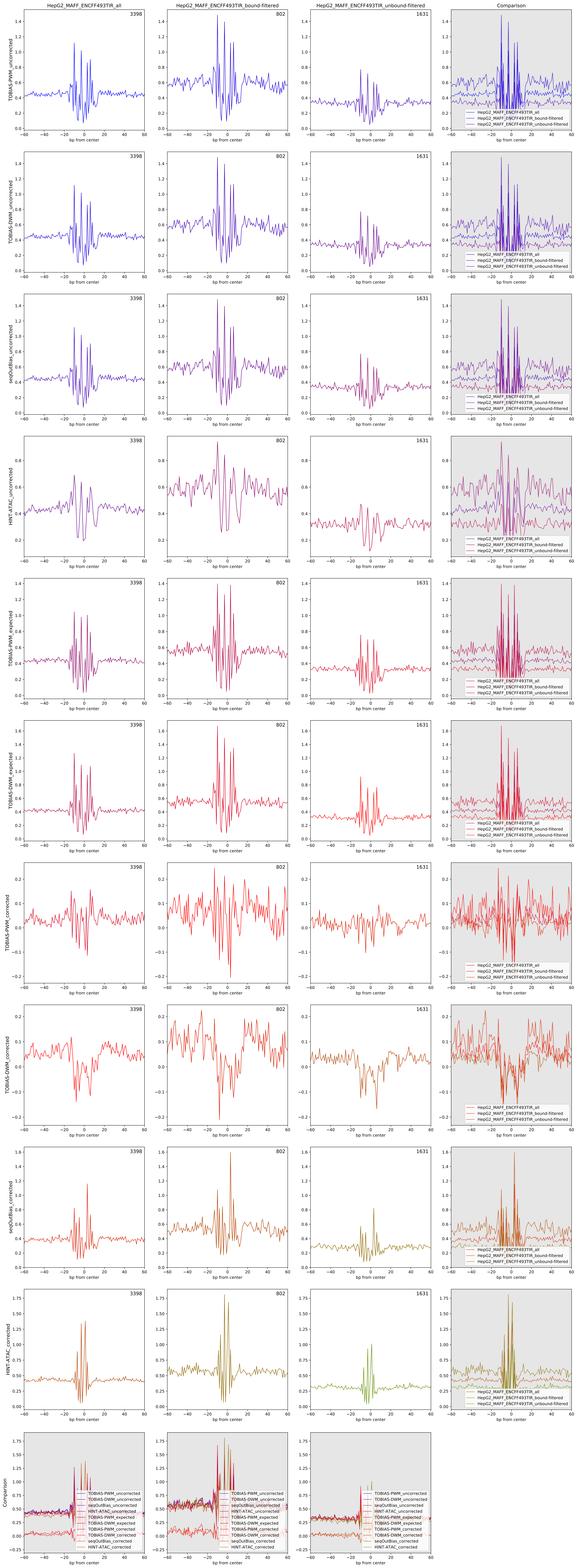

Aggregate footprints in HepG2 for TF MAFK (ChIP-seq ENCFF171OJF)

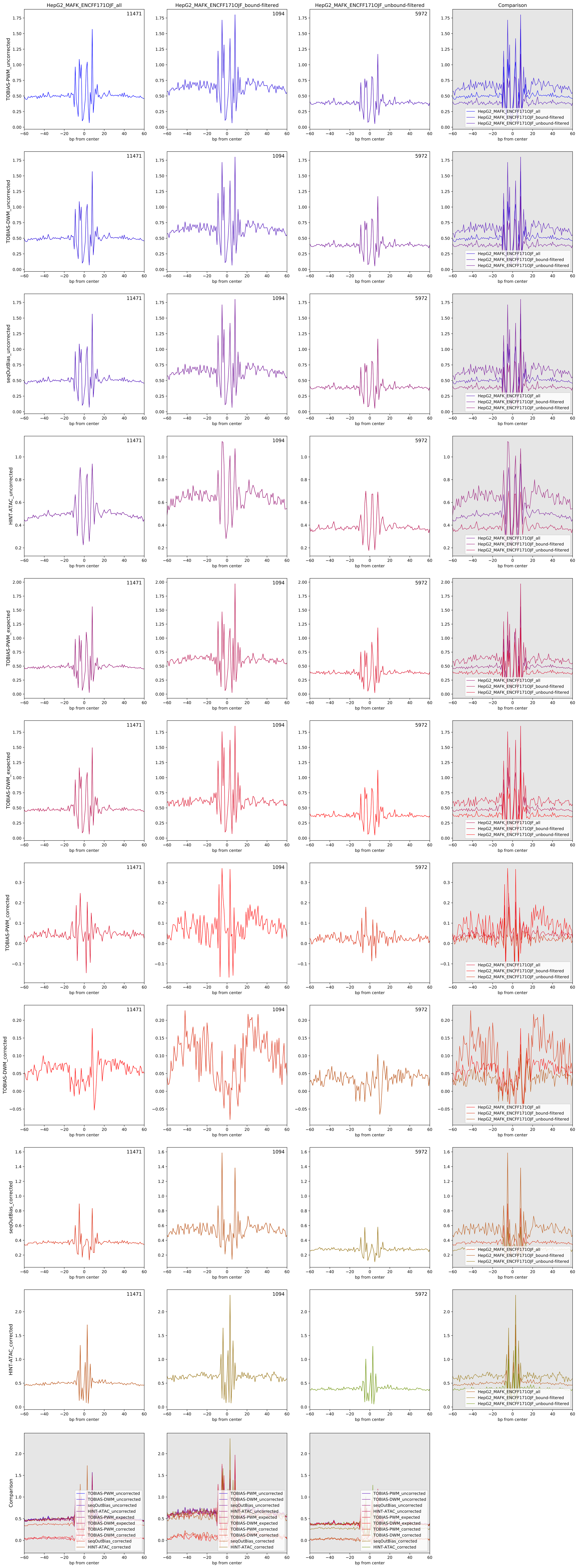

Aggregate footprints in HepG2 for TF MAX (ChIP-seq ENCFF140PUO)

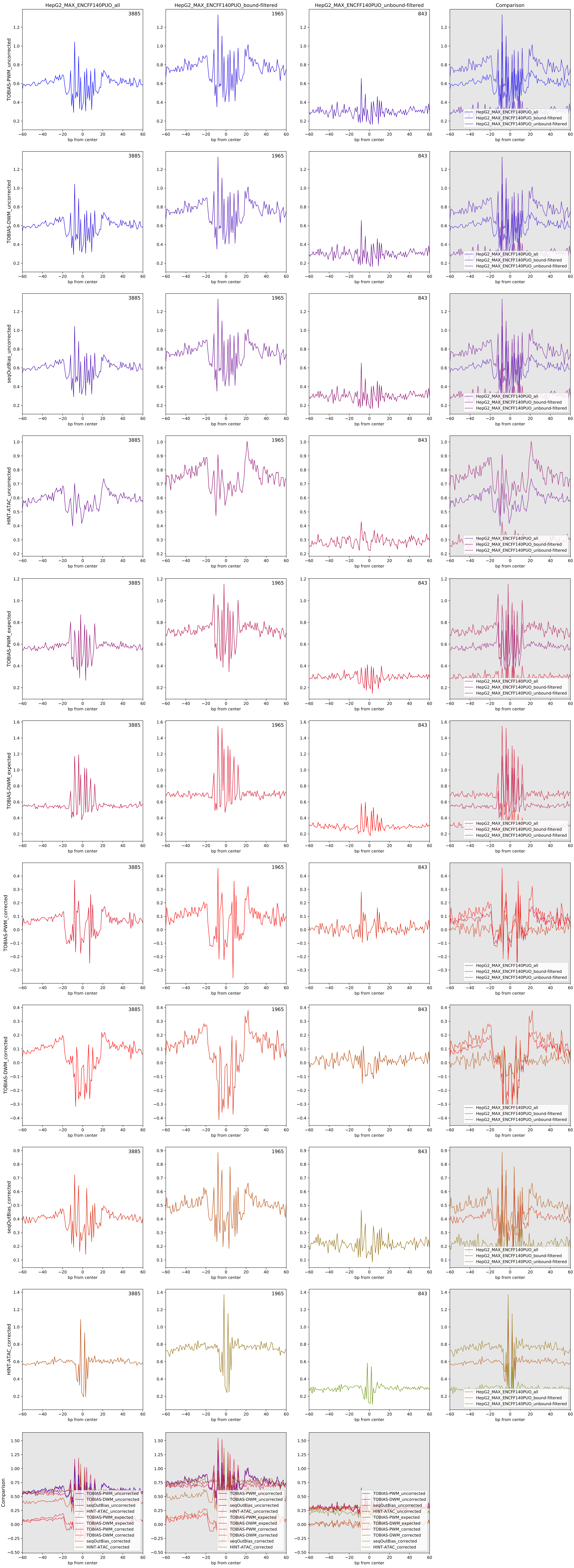

Aggregate footprints in HepG2 for TF MIXL1 (ChIP-seq ENCFF963OLI)

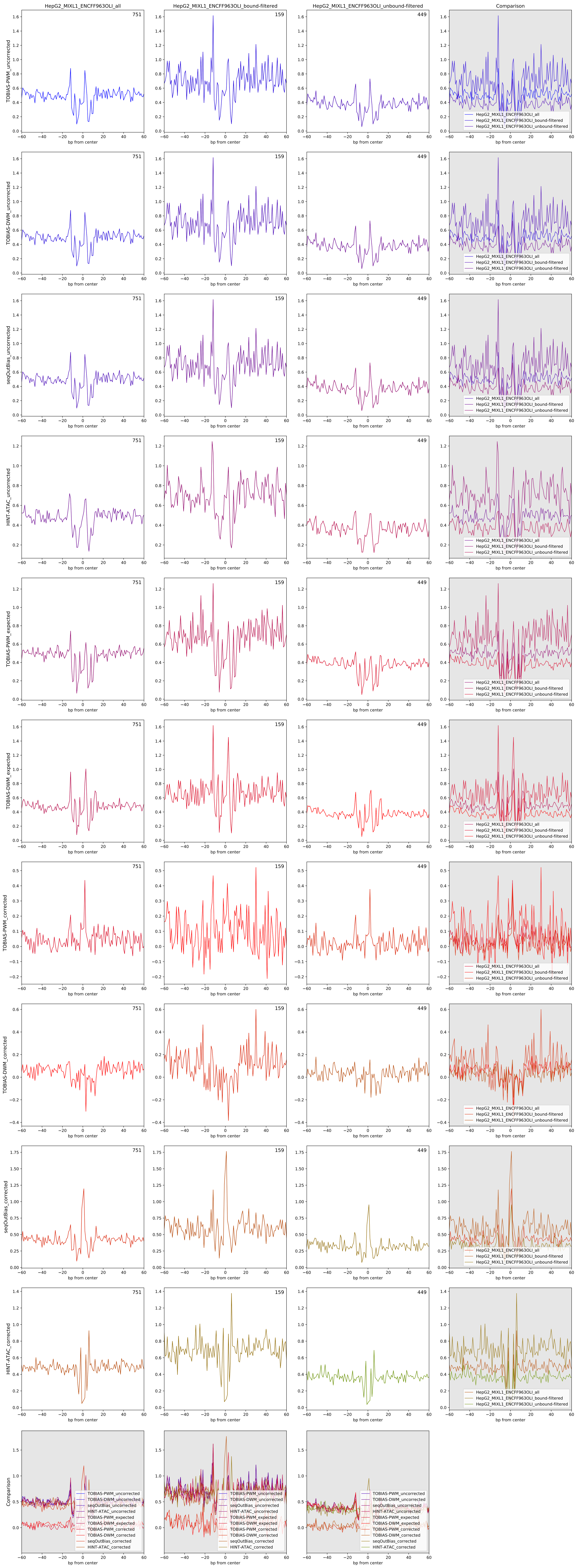

Aggregate footprints in HepG2 for TF MLX (ChIP-seq ENCFF232WMR)

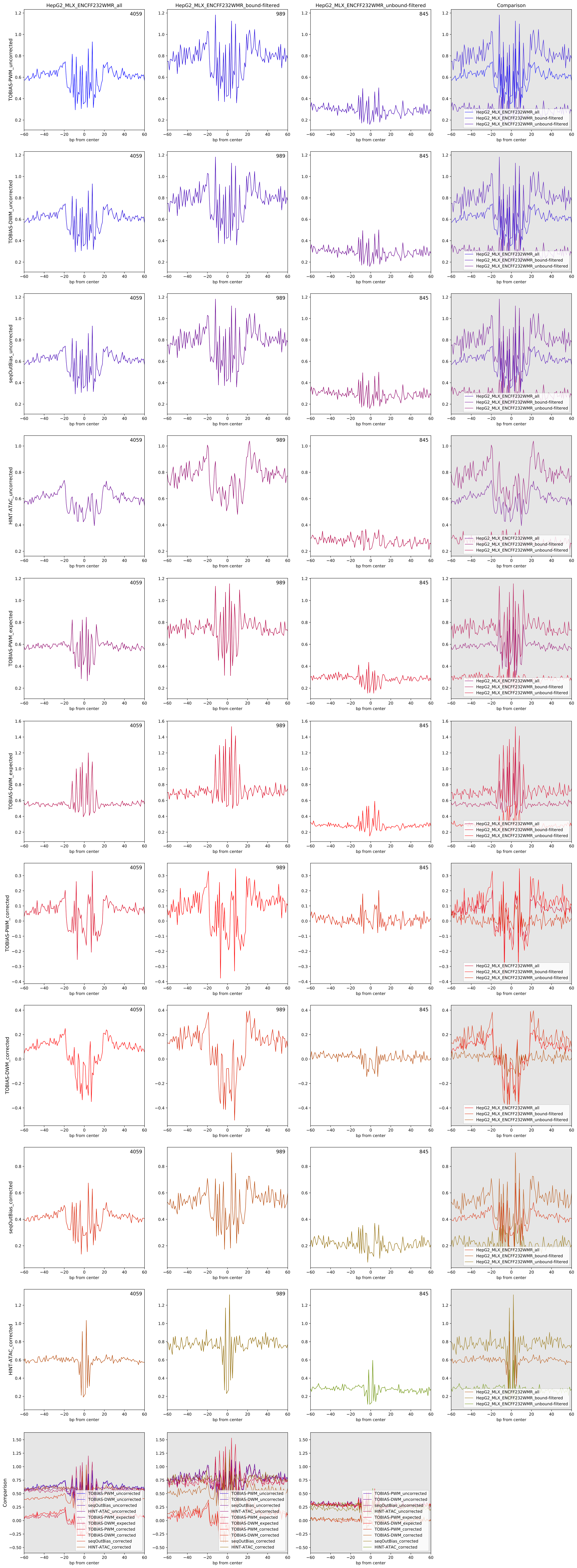

Aggregate footprints in HepG2 for TF MNT (ChIP-seq ENCFF562FMQ)

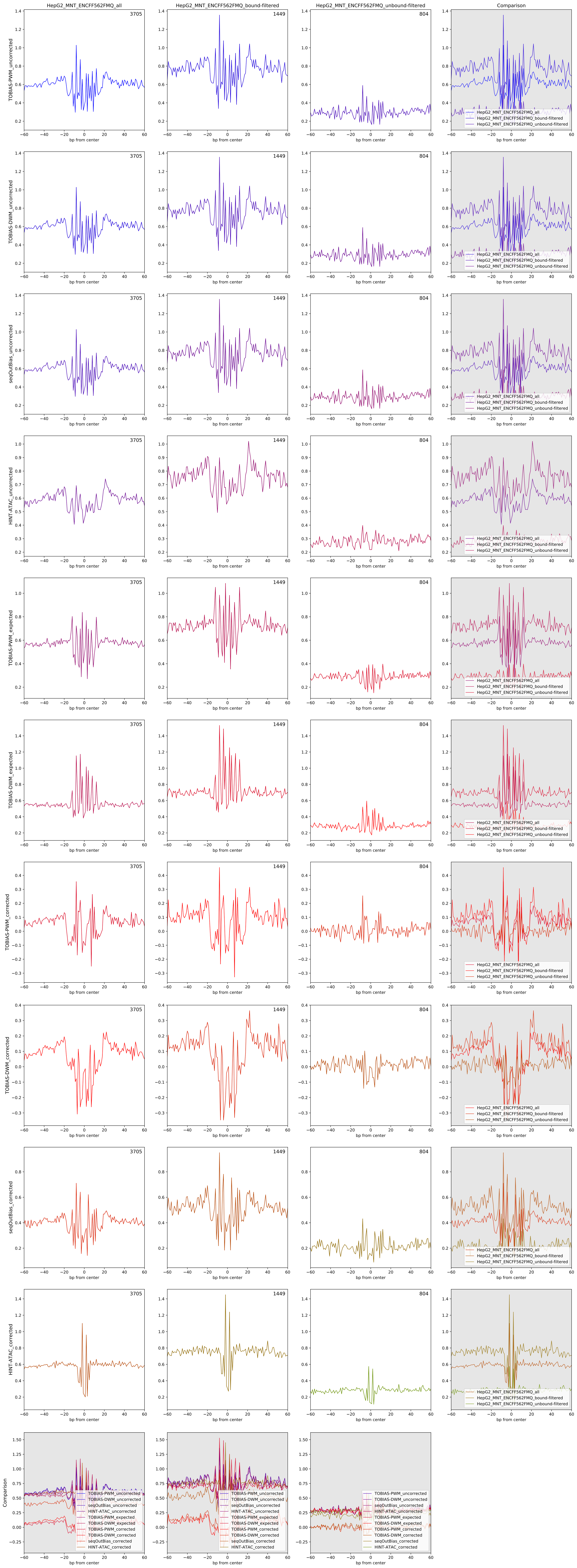

Aggregate footprints in HepG2 for TF NFE2L2 (ChIP-seq ENCFF882YLO)

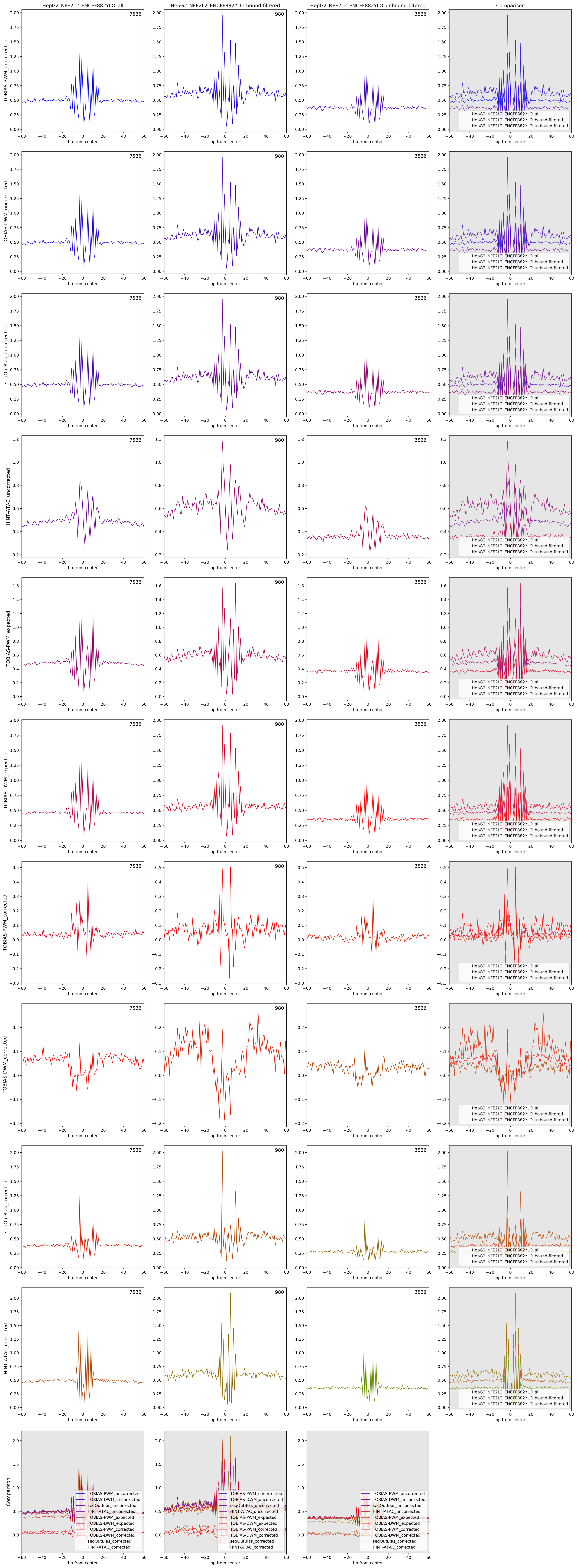

Aggregate footprints in HepG2 for TF NFIA (ChIP-seq ENCFF926VIT)

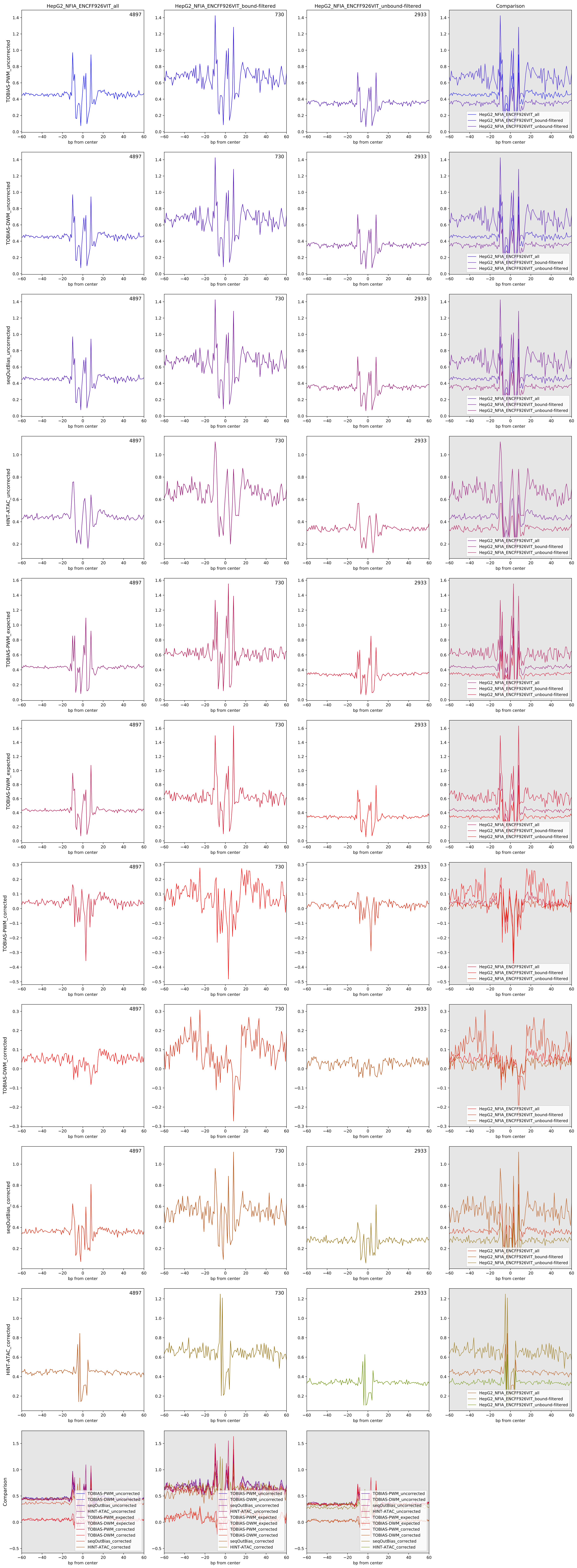

Aggregate footprints in HepG2 for TF NFIL3 (ChIP-seq ENCFF278ZNM)

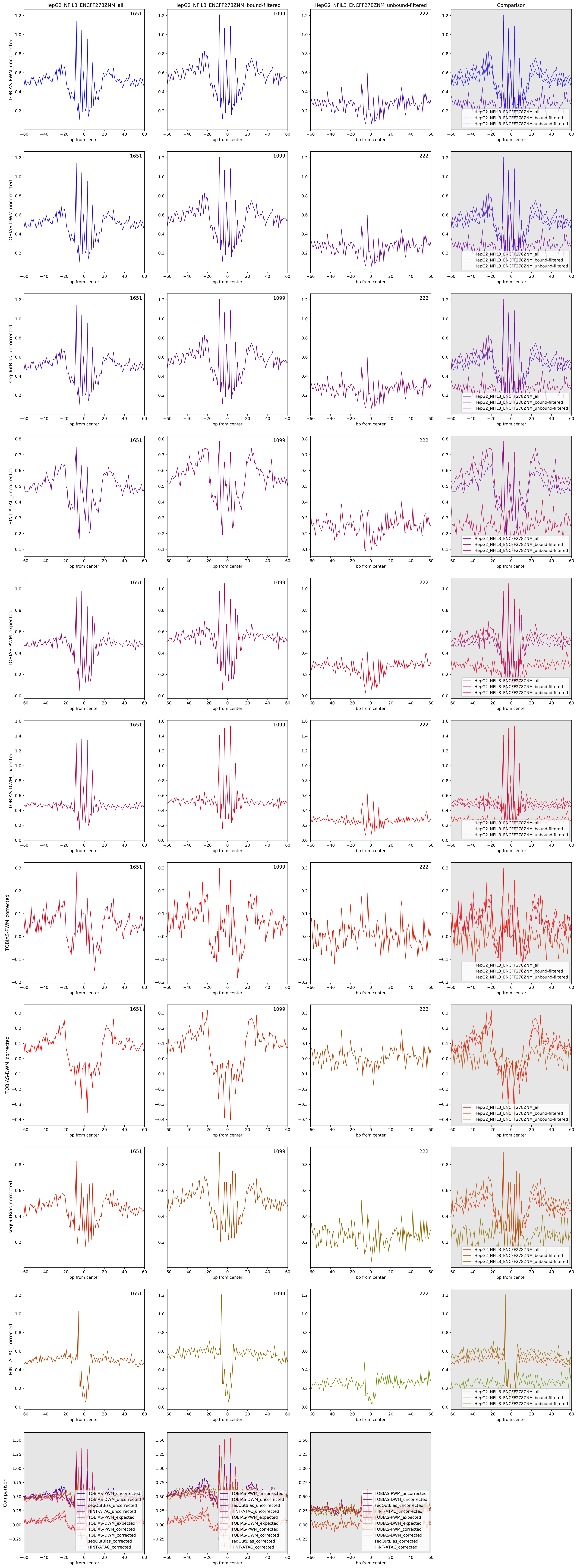

Aggregate footprints in HepG2 for TF NR2F1 (ChIP-seq ENCFF295CUB)

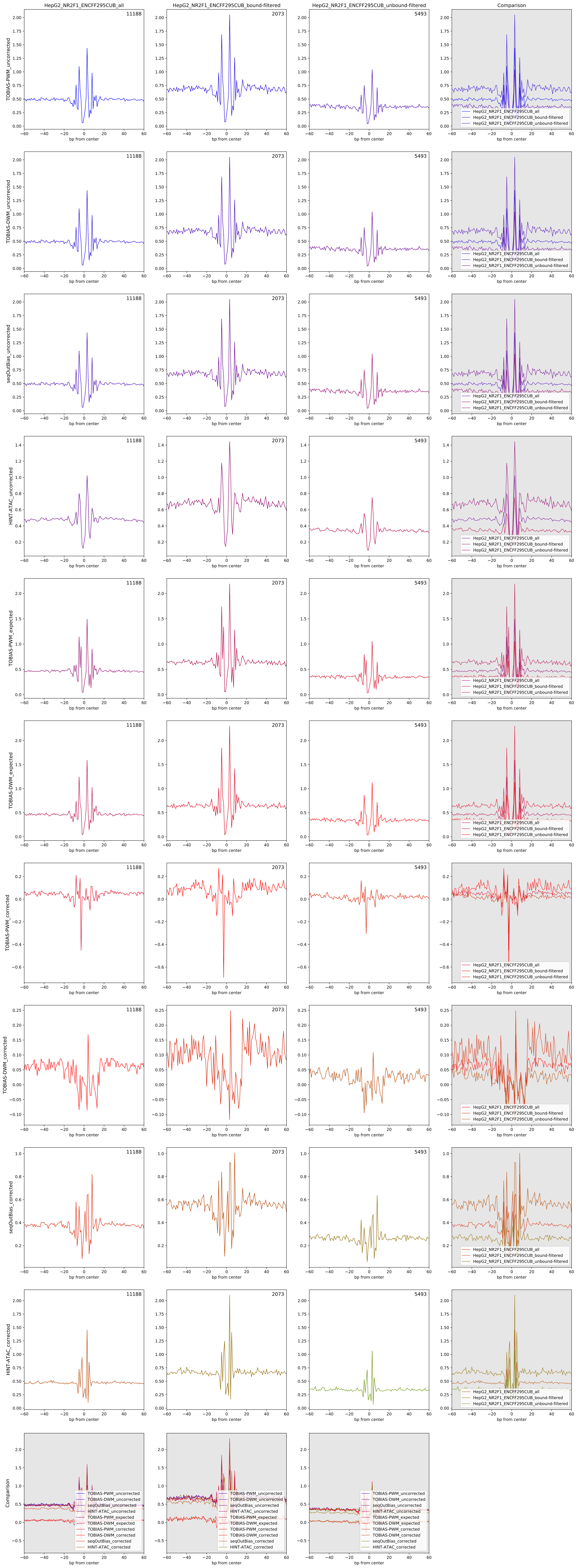

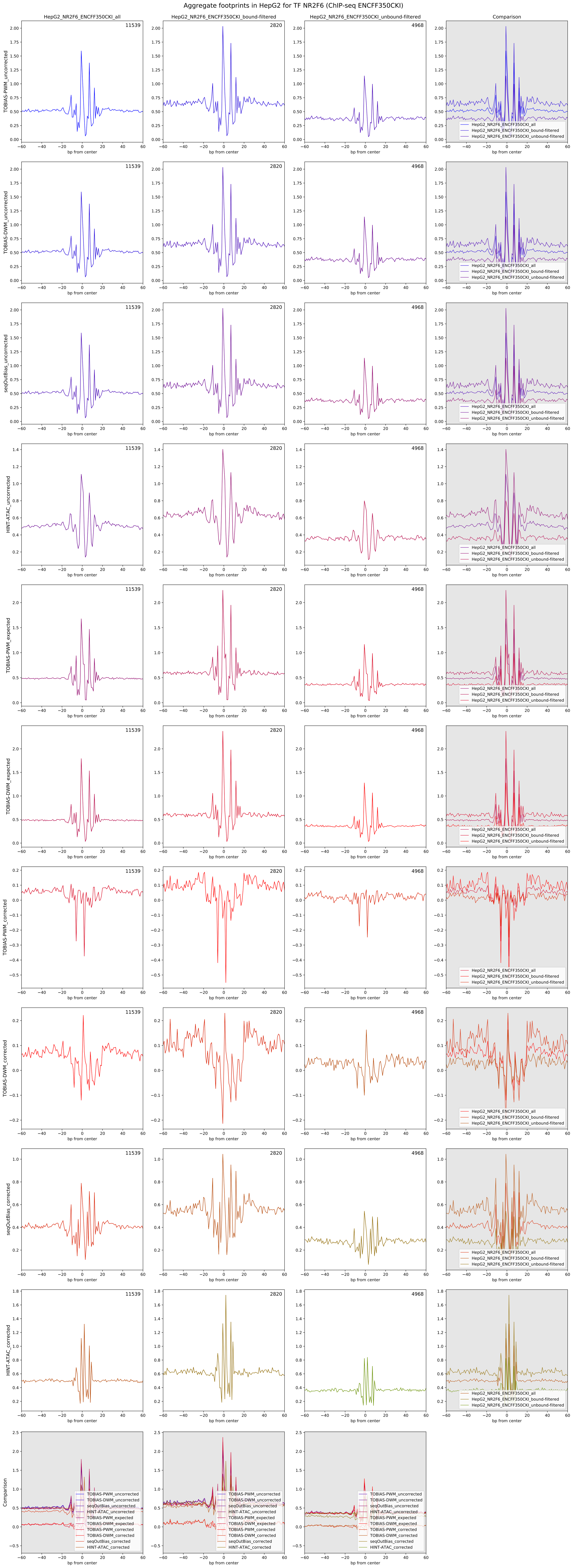

Aggregate footprints in HepG2 for TF NR2F6 (ChIP-seq ENCFF350CKI)
HepG2_NR2F6_ENCFF350CKI_all
HepG2_NR2F6_ENCFF350CKI_bound-filtered
HepG2_NR2F6_ENCFF350CKI_unbound-filtered
Comparison
11539
2820
4968
TOBIAS-PWM_uncorrected
TOBIAS-DWM_uncorrected
seqOutBias_uncorrected
HINT-ATAC_uncorrected
TOBIAS-PWM_expected
TOBIAS-DWM_expected
TOBIAS-PWM_corrected
TOBIAS-DWM_corrected
seqOutBias_corrected
HINT-ATAC_corrected
Comparison
bp from center

Aggregate footprints in HepG2 for TF NRF1 (ChIP-seq ENCFF313RFR)

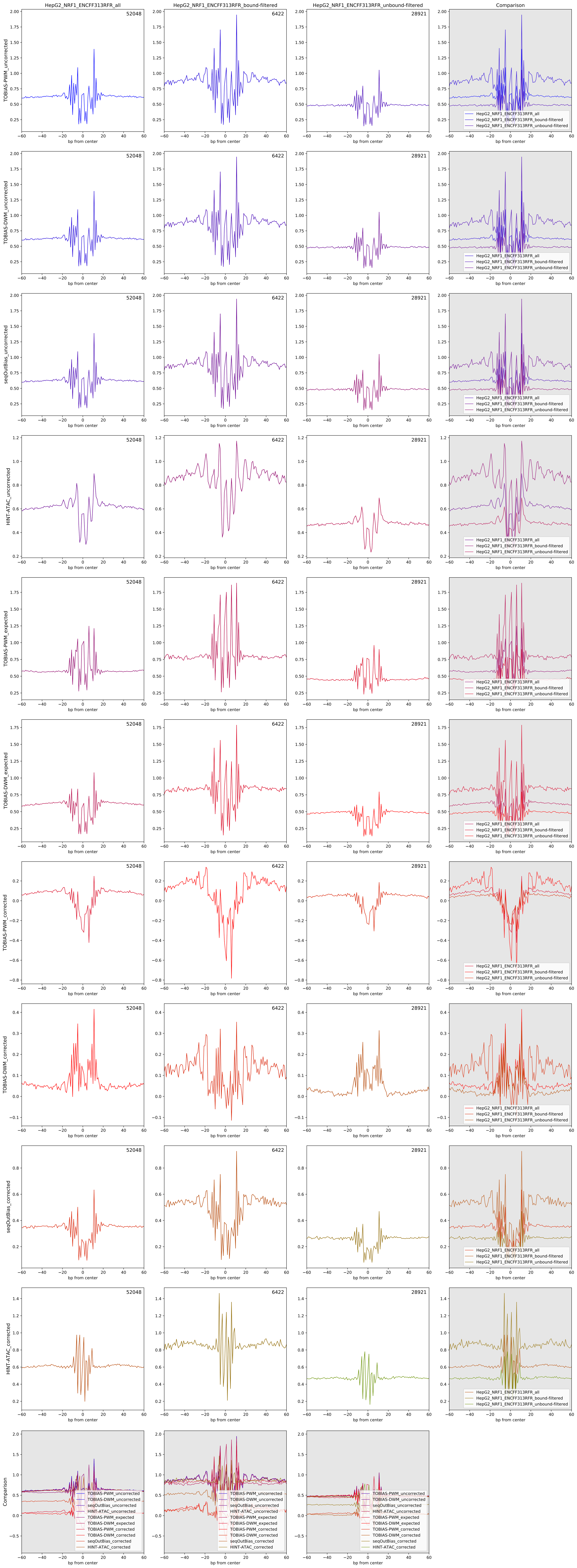

Aggregate footprints in HepG2 for TF PBX2 (ChIP-seq ENCFF709YWO)

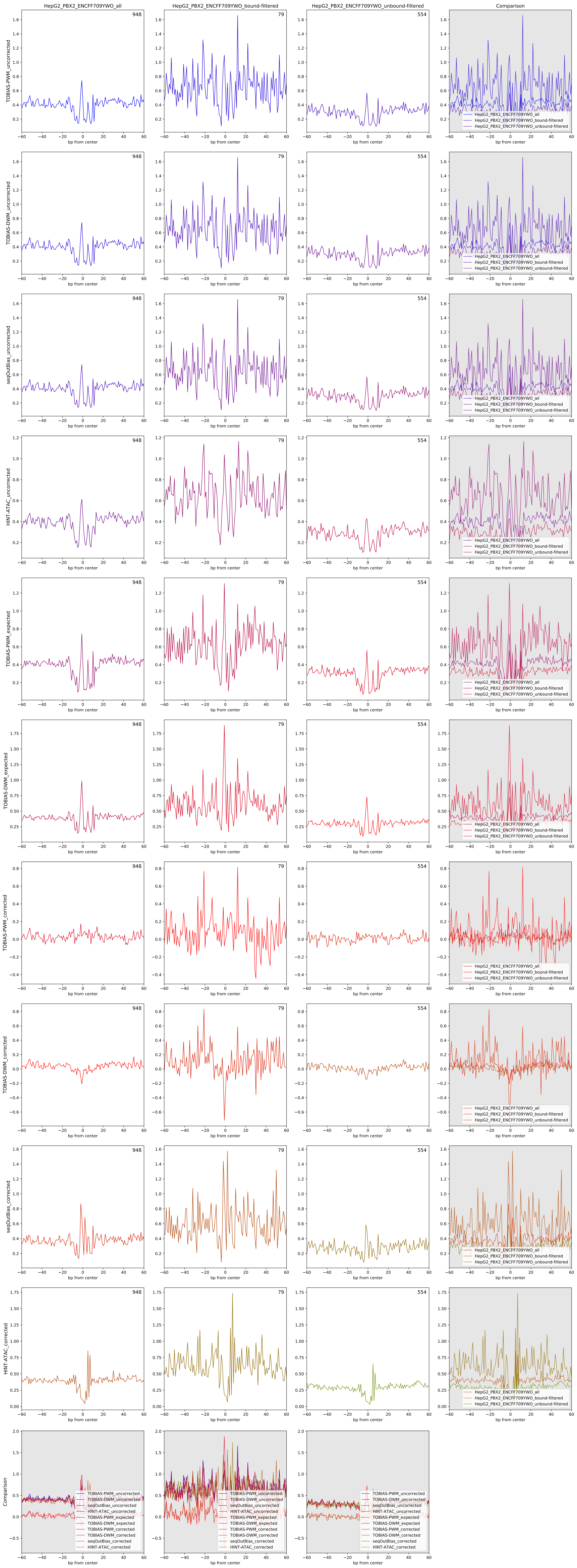

Aggregate footprints in HepG2 for TF PPARG (ChIP-seq ENCFF736PPV)

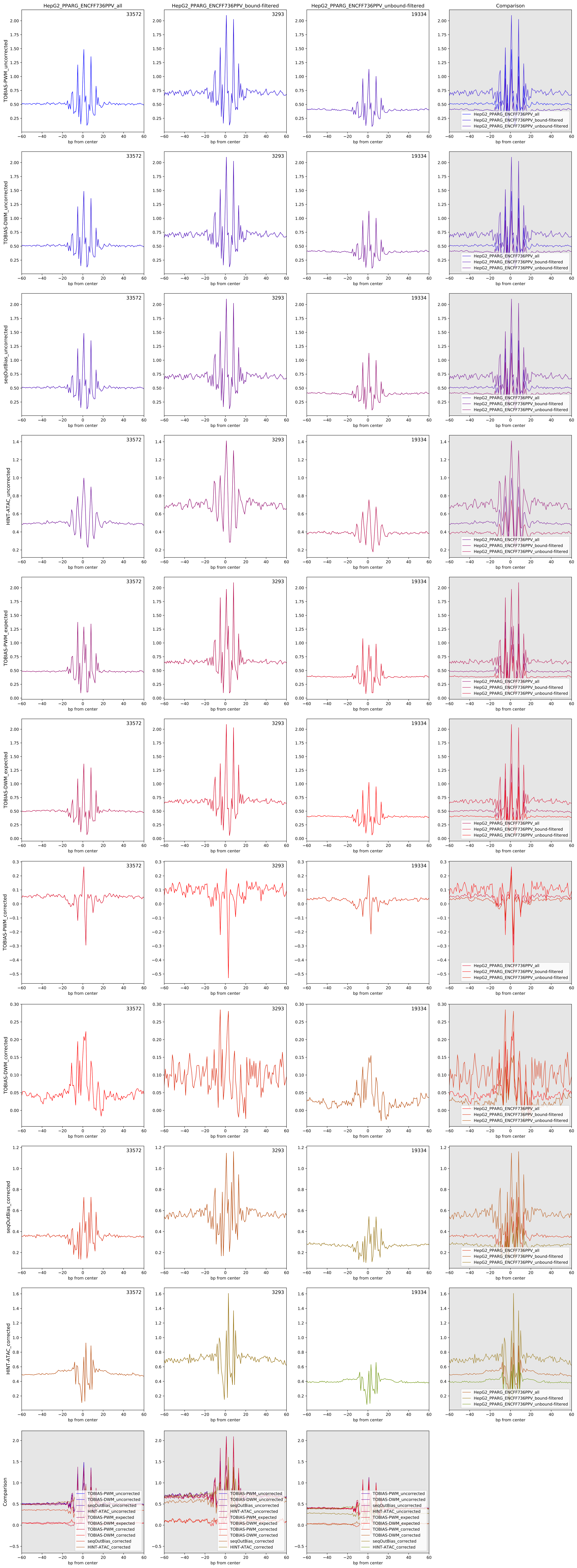

Aggregate footprints in HepG2 for TF RBPJ (ChIP-seq ENCFF153ODZ)

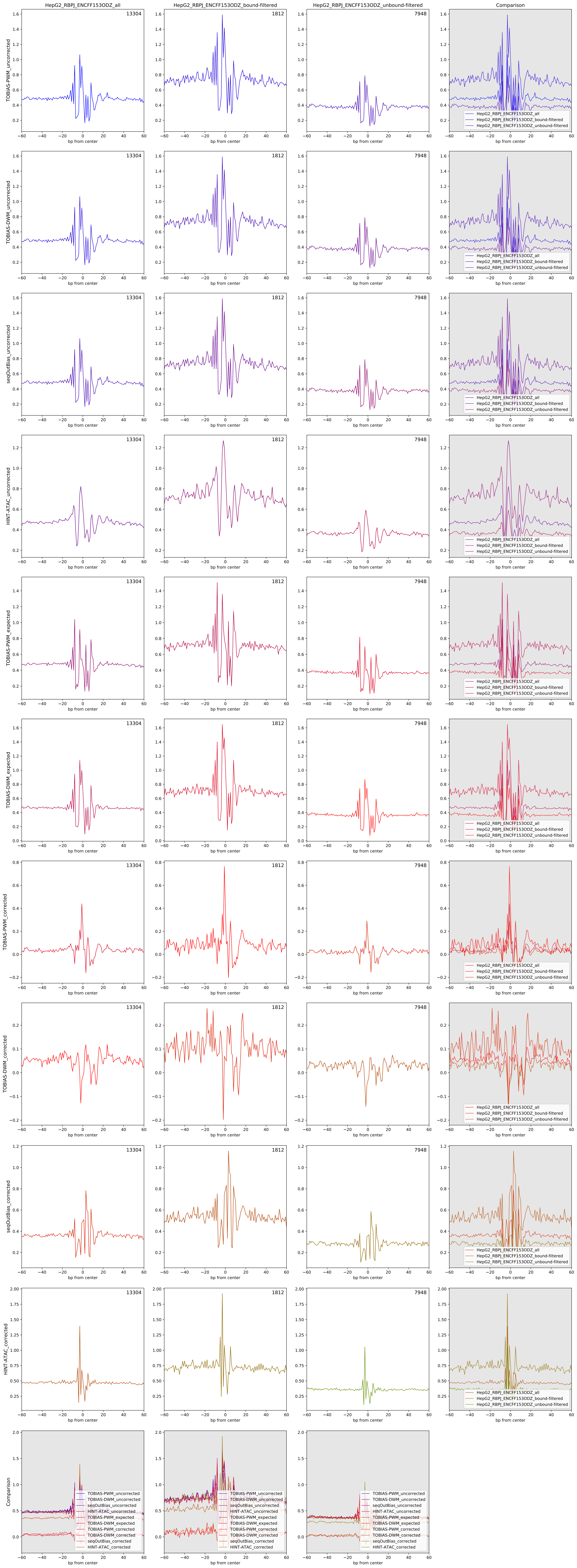

Aggregate footprints in HepG2 for TF REST (ChIP-seq ENCFF986RRJ)

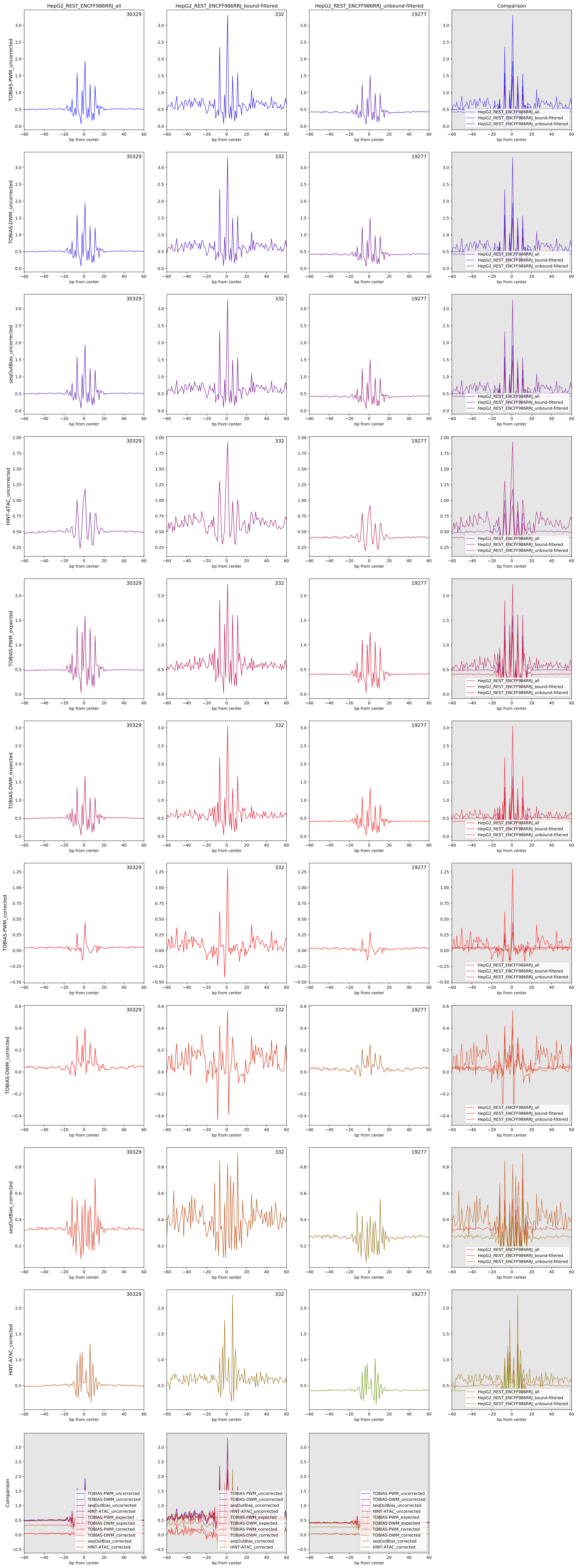

Aggregate footprints in HepG2 for TF RFX1 (ChIP-seq ENCFF788CJF)

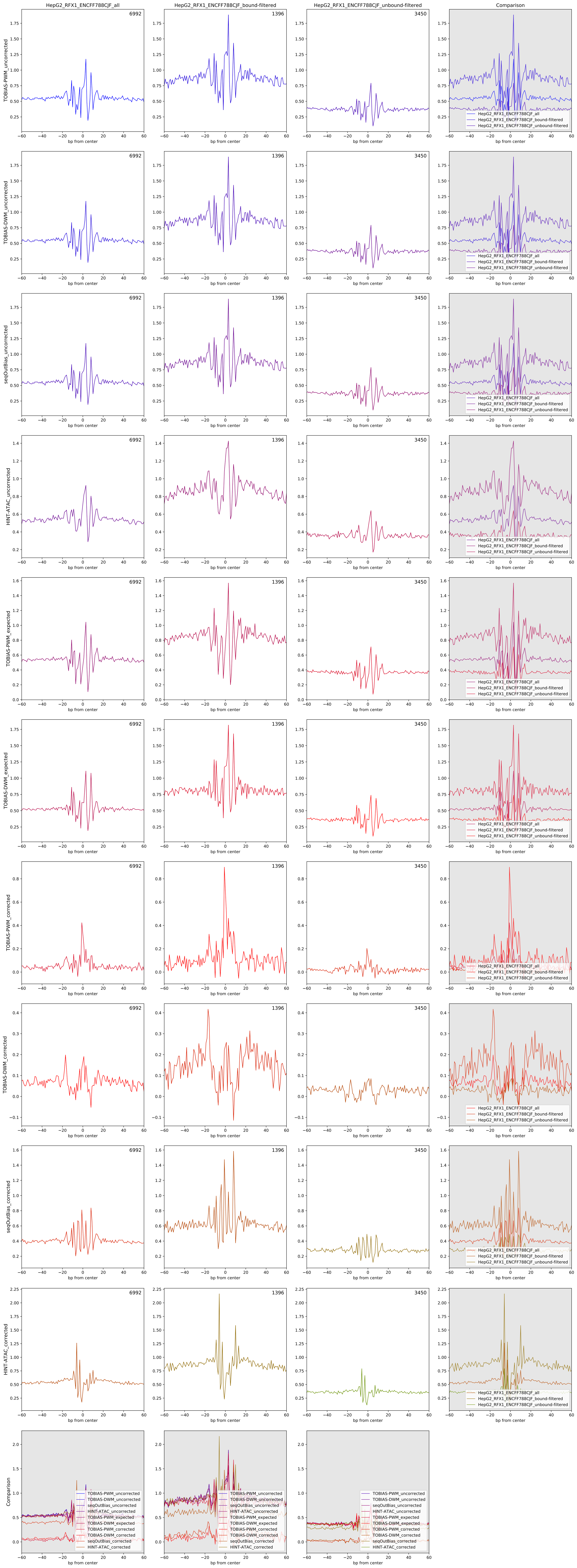

Aggregate footprints in HepG2 for TF RFX3 (ChIP-seq ENCFF431YWW)

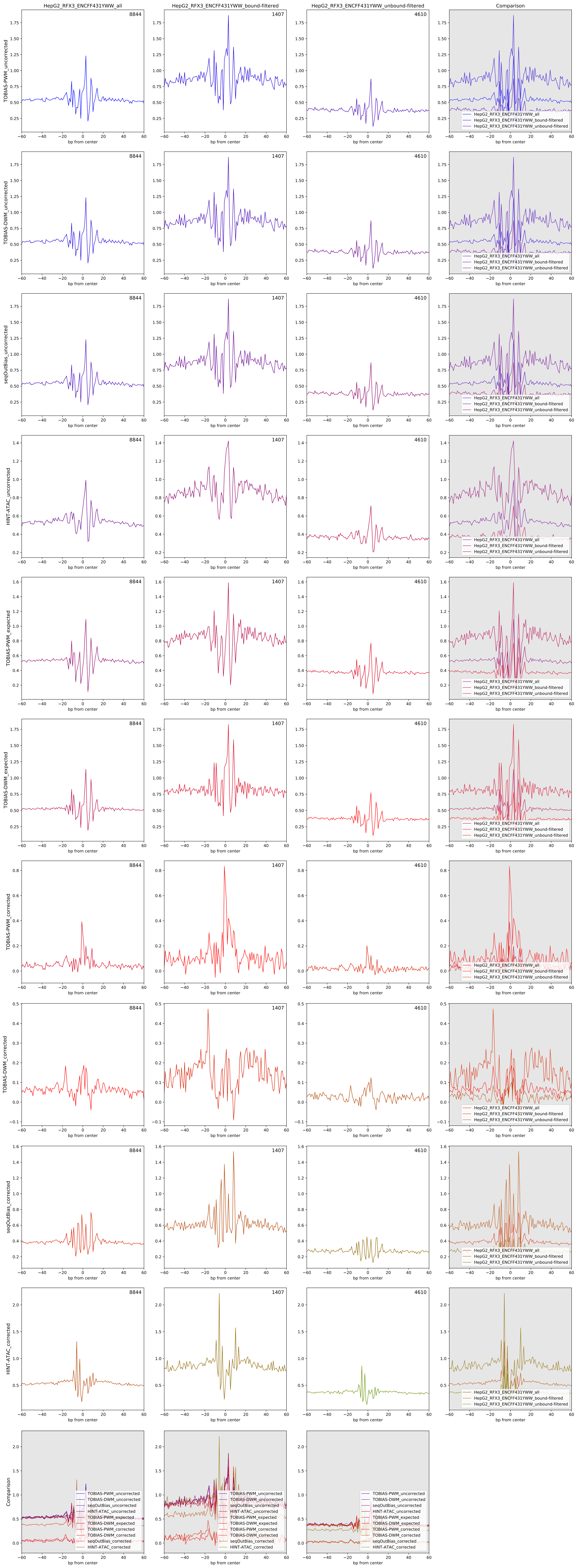

Aggregate footprints in HepG2 for TF RFX5 (ChIP-seq ENCFF059GWW)

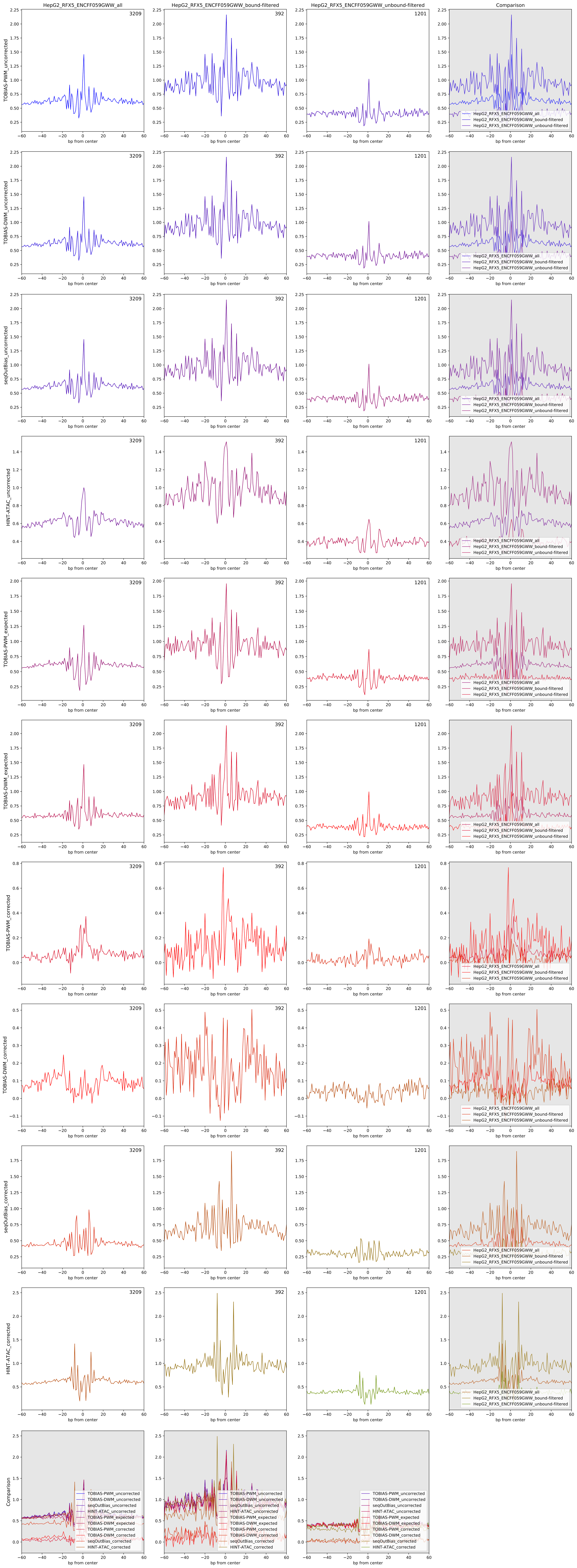

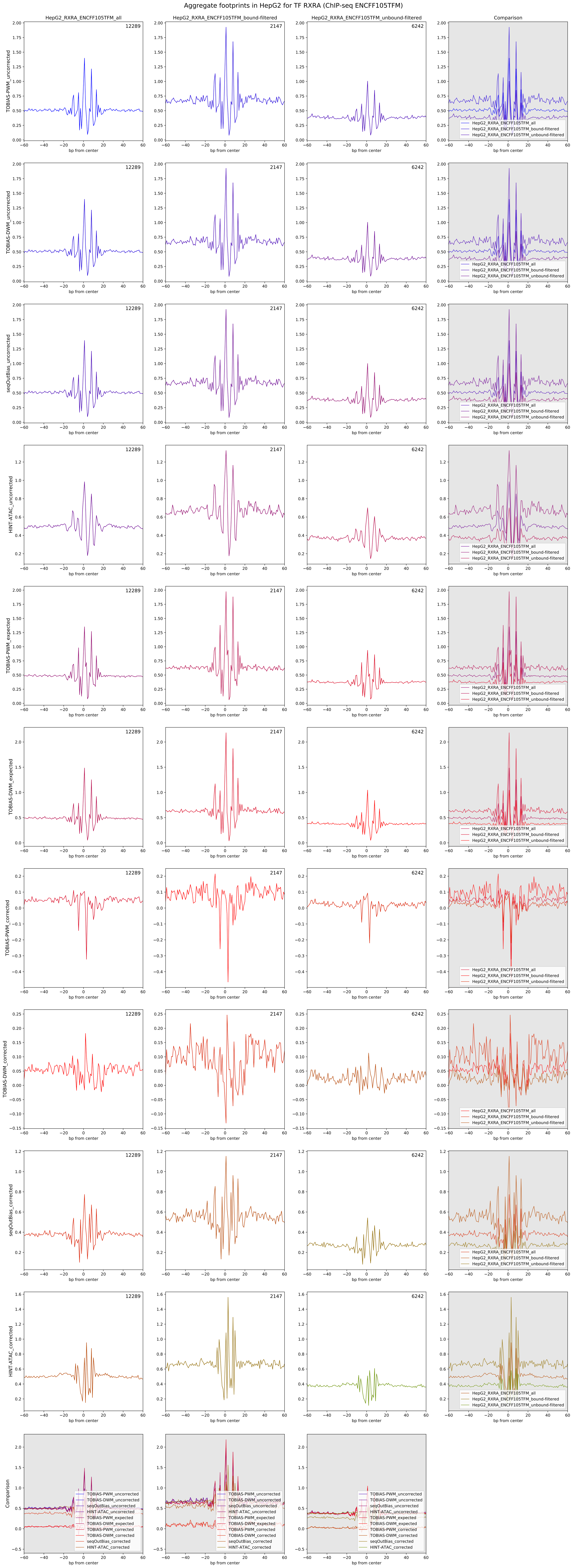
Aggregate footprints in HepG2 for TF RXRA (ChIP-seq ENCFF105TFM)
HepG2_RXRA_ENCFF105TFM_all
HepG2_RXRA_ENCFF105TFM_bound-filtered
HepG2_RXRA_ENCFF105TFM_unbound-filtered
Comparison
12289
2147
6242
TOBIAS-PWM_uncorrected
TOBIAS-DWM_uncorrected
seqOutBias_uncorrected
HINT-ATAC_uncorrected
TOBIAS-PWM_expected
TOBIAS-DWM_expected
TOBIAS-PWM_corrected
TOBIAS-DWM_corrected
seqOutBias_corrected
HINT-ATAC_corrected
Comparison
bp from center
HepG2_RXRA_ENCFF105TFM_all
HepG2_RXRA_ENCFF105TFM_bound-filtered
HepG2_RXRA_ENCFF105TFM_unbound-filtered
TOBIAS-PWM_uncorrected
TOBIAS-DWM_uncorrected
seqOutBias_uncorrected
HINT-ATAC_uncorrected
TOBIAS-PWM_expected
TOBIAS-DWM_expected
TOBIAS-PWM_corrected
TOBIAS-DWM_corrected
seqOutBias_corrected
HINT-ATAC_corrected

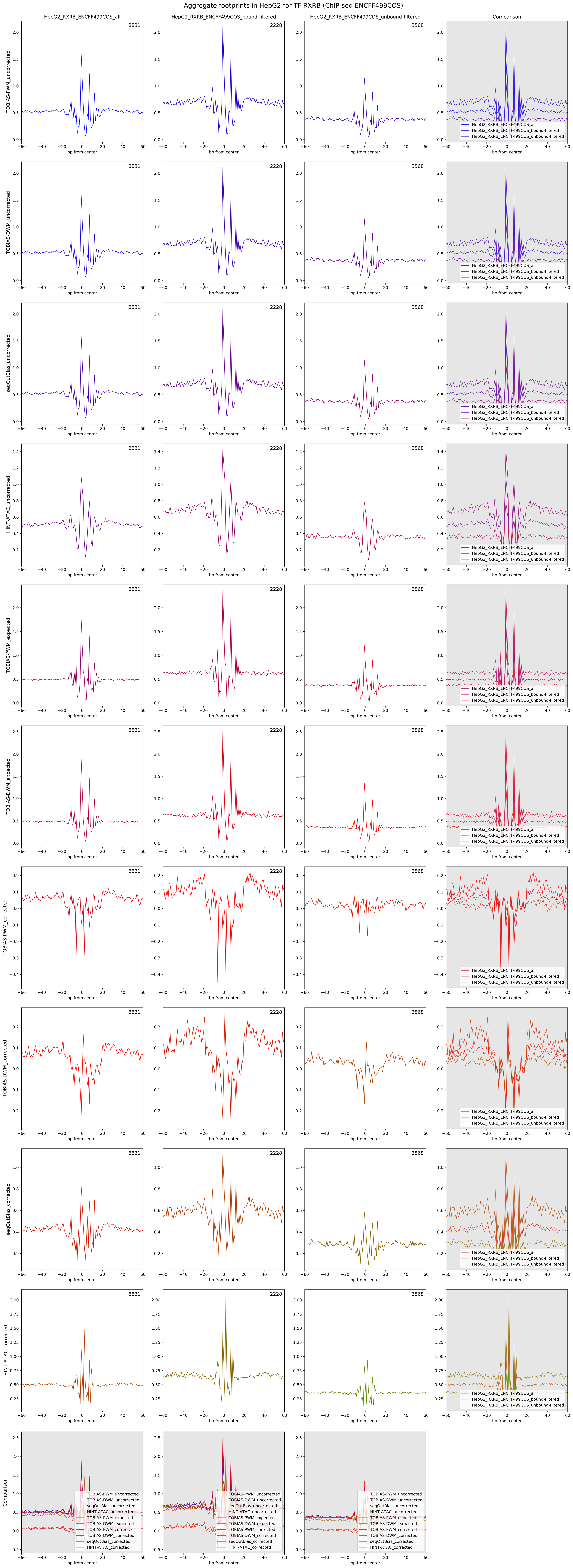
Aggregate footprints in HepG2 for TF RXRB (ChIP-seq ENCFF499COS)
HepG2_RXRB_ENCFF499COS_all
HepG2_RXRB_ENCFF499COS_bound-filtered
HepG2_RXRB_ENCFF499COS_unbound-filtered
Comparison
TOBIAS-PWM_uncorrected
TOBIAS-DWM_uncorrected
seqOutBias_uncorrected
HINT-ATAC_uncorrected
TOBIAS-PWM_expected
TOBIAS-DWM_expected
TOBIAS-PWM_corrected
TOBIAS-DWM_corrected
seqOutBias_corrected
HINT-ATAC_corrected
8831
2228
3568
bp from center

Aggregate footprints in HepG2 for TF SMAD4 (ChIP-seq ENCFF541EKJ)

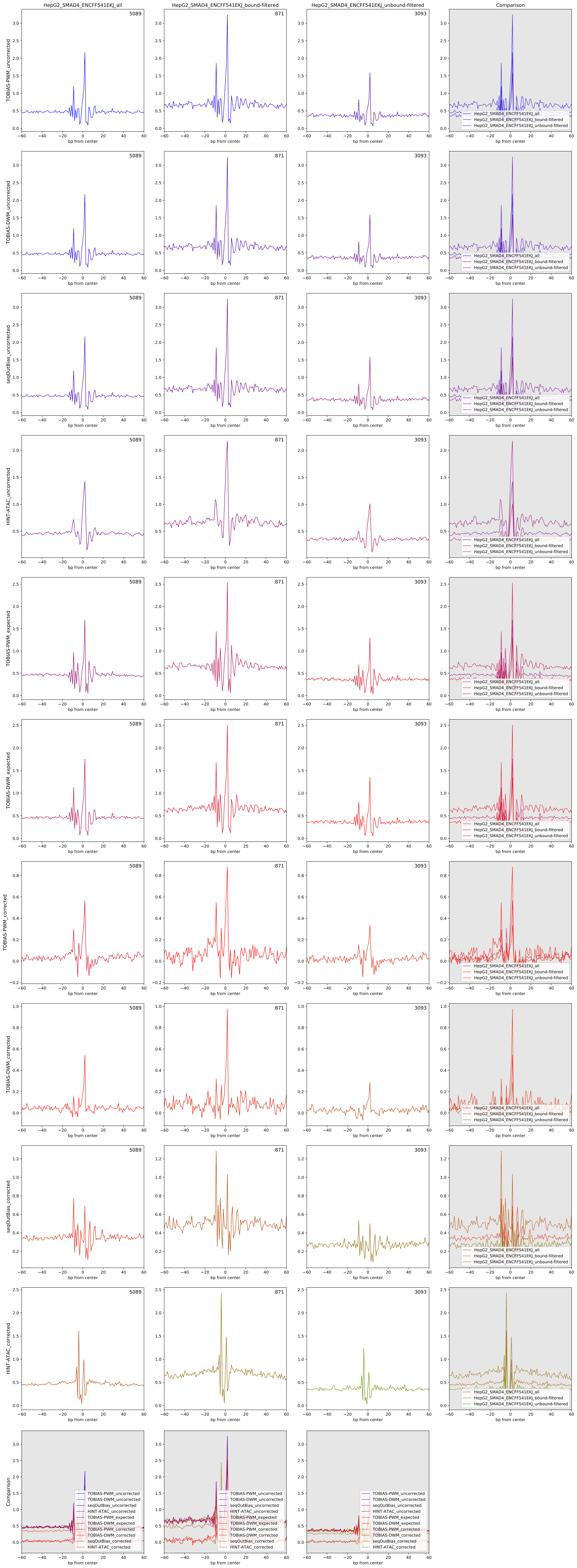

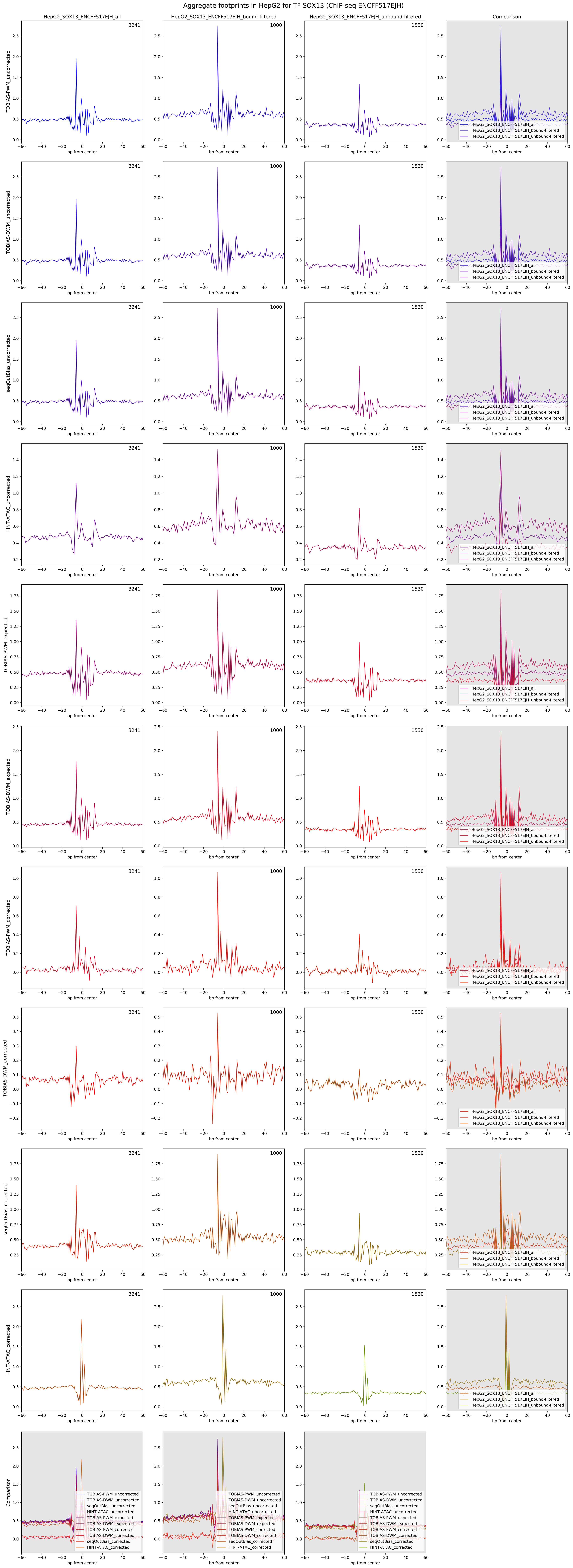
Aggregate footprints in HepG2 for TF SOX13 (ChIP-seq ENCFF517EJH)
HepG2_SOX13_ENCFF517EJH_all
HepG2_SOX13_ENCFF517EJH_bound-filtered
HepG2_SOX13_ENCFF517EJH_unbound-filtered
Comparison
TOBIAS-PWM_uncorrected
TOBIAS-DWM_uncorrected
seqOutBias_uncorrected
HINT-ATAC_uncorrected
TOBIAS-PWM_expected
TOBIAS-DWM_expected
TOBIAS-PWM_corrected
TOBIAS-DWM_corrected
seqOutBias_corrected
HINT-ATAC_corrected
3241
1000
1530
bp from center

Aggregate footprints in HepG2 for TF SOX5 (ChIP-seq ENCFF703VBH)

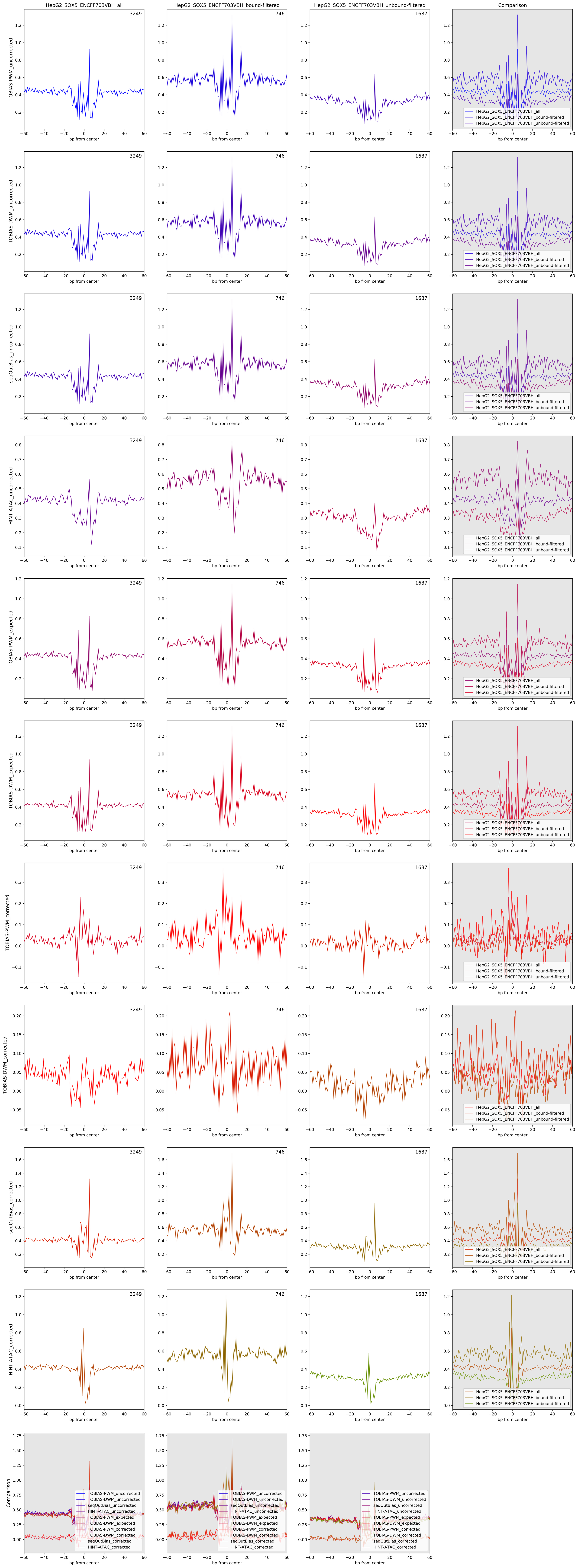

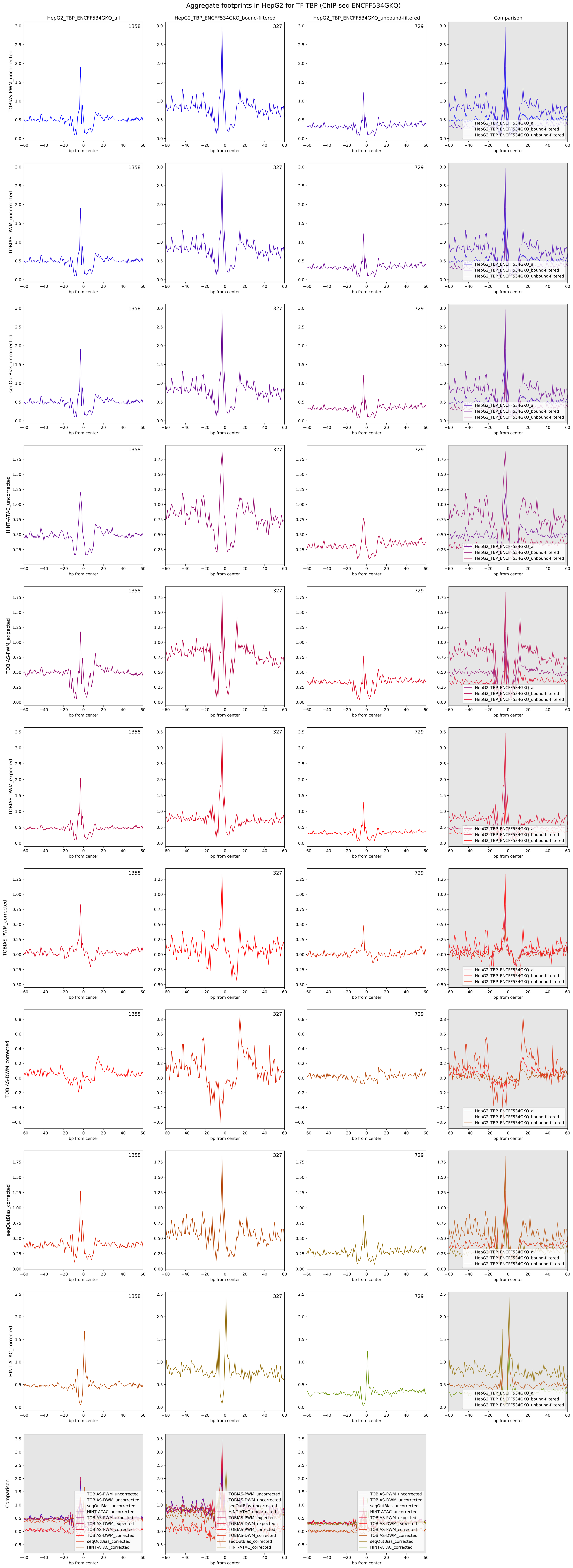
Aggregate footprints in HepG2 for TF TBP (ChIP-seq ENCFF534GKQ)
HepG2_TBP_ENCFF534GKQ_all
HepG2_TBP_ENCFF534GKQ_bound-filtered
HepG2_TBP_ENCFF534GKQ_unbound-filtered
Comparison
TOBIAS-PWM_uncorrected
TOBIAS-DWM_uncorrected
seqOutBias_uncorrected
HINT-ATAC_uncorrected
TOBIAS-PWM_expected
TOBIAS-DWM_expected
TOBIAS-PWM_corrected
TOBIAS-DWM_corrected
seqOutBias_corrected
HINT-ATAC_corrected
1358
327
729
bp from center

Aggregate footprints in HepG2 for TF TCF7 (ChIP-seq ENCFF928MIN)

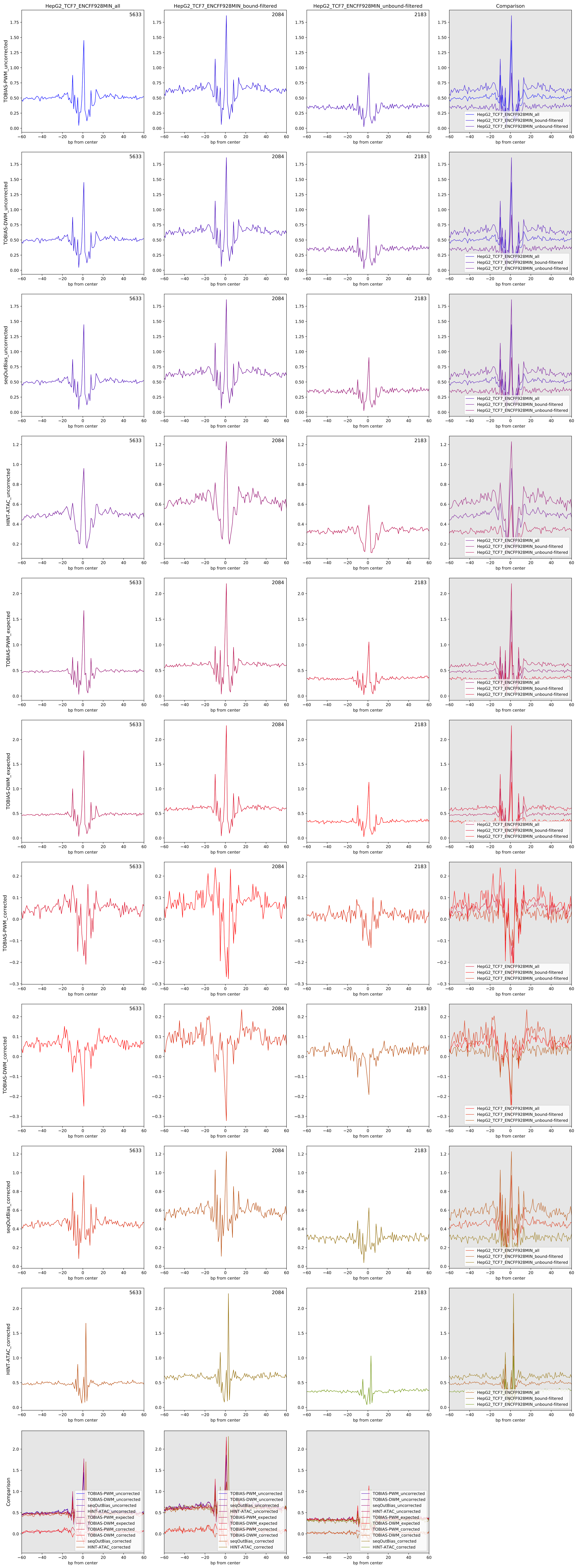

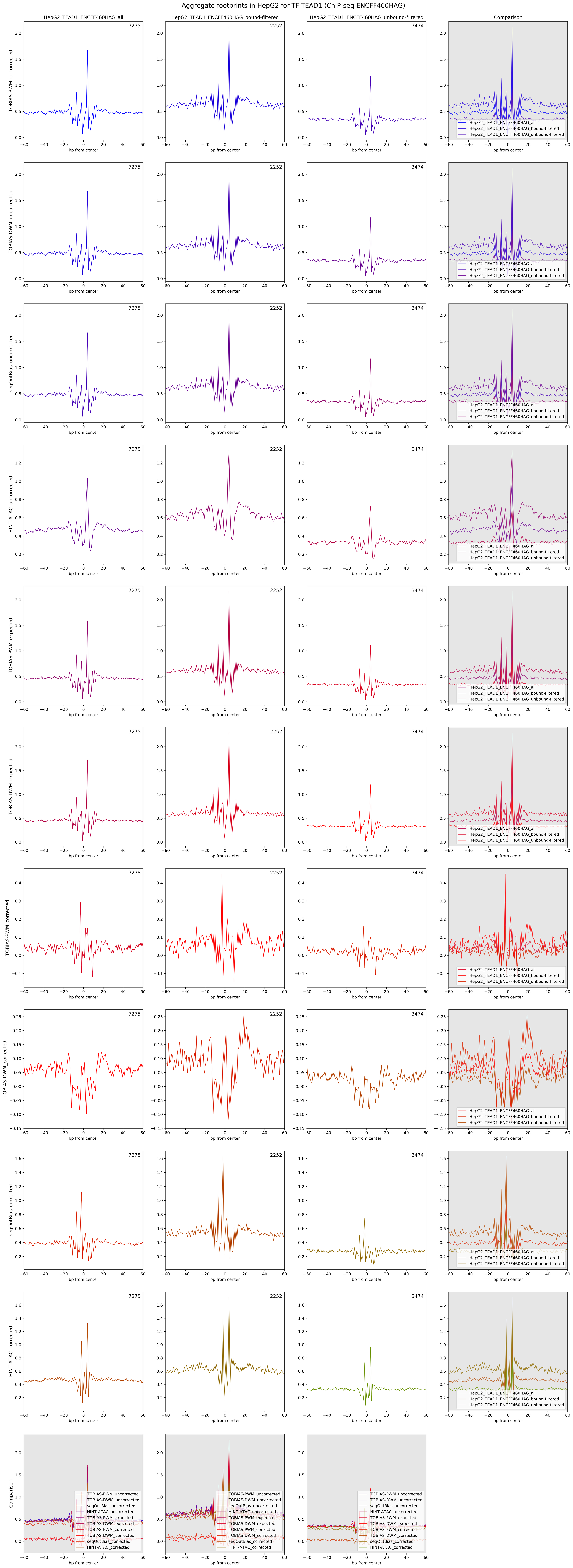

Aggregate footprints in HepG2 for TF TEAD1 (ChIP-seq ENCFF460HAG)
HepG2_TEAD1_ENCFF460HAG_all
HepG2_TEAD1_ENCFF460HAG_bound-filtered
HepG2_TEAD1_ENCFF460HAG_unbound-filtered
Comparison
TOBIAS-PWM_uncorrected
TOBIAS-DWM_uncorrected
seqOutBias_uncorrected
HINT-ATAC_uncorrected
TOBIAS-PWM_expected
TOBIAS-DWM_expected
TOBIAS-PWM_corrected
TOBIAS-DWM_corrected
seqOutBias_corrected
HINT-ATAC_corrected
7275
2252
3474
bp from center

Aggregate footprints in HepG2 for TF TEAD3 (ChIP-seq ENCFF314CDT)

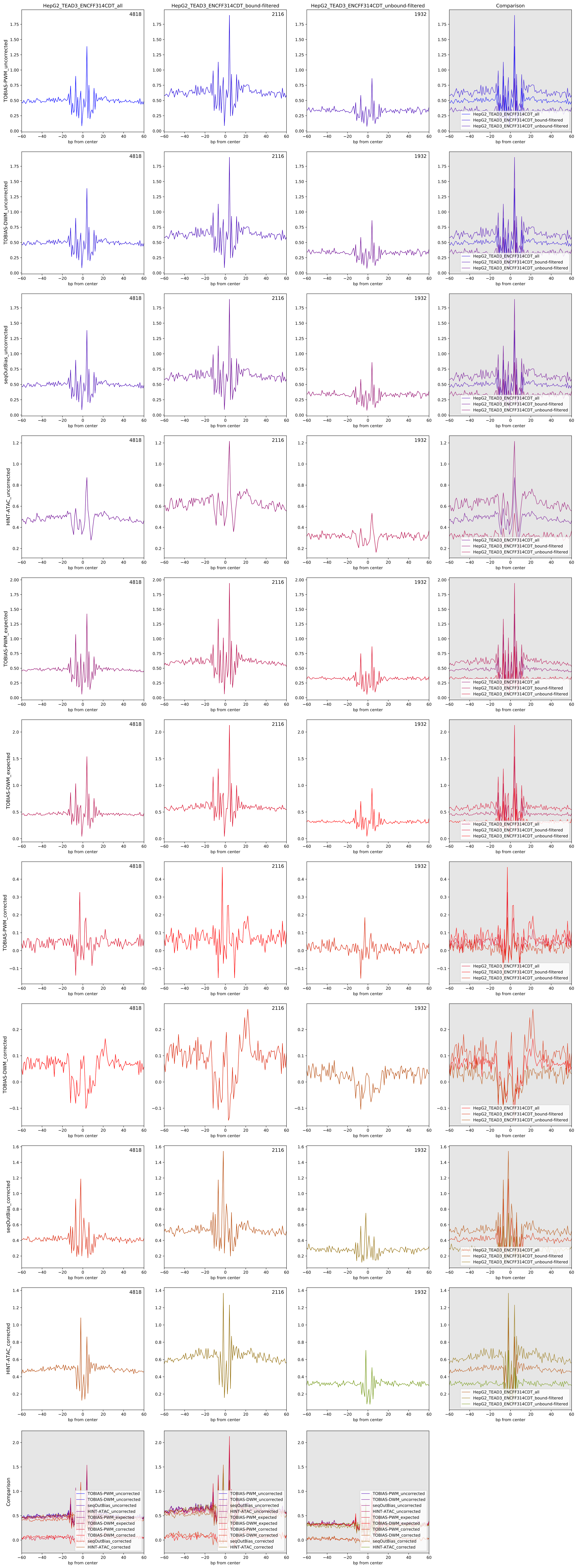

Aggregate footprints in HepG2 for TF TFAP4 (ChIP-seq ENCFF912SQI)

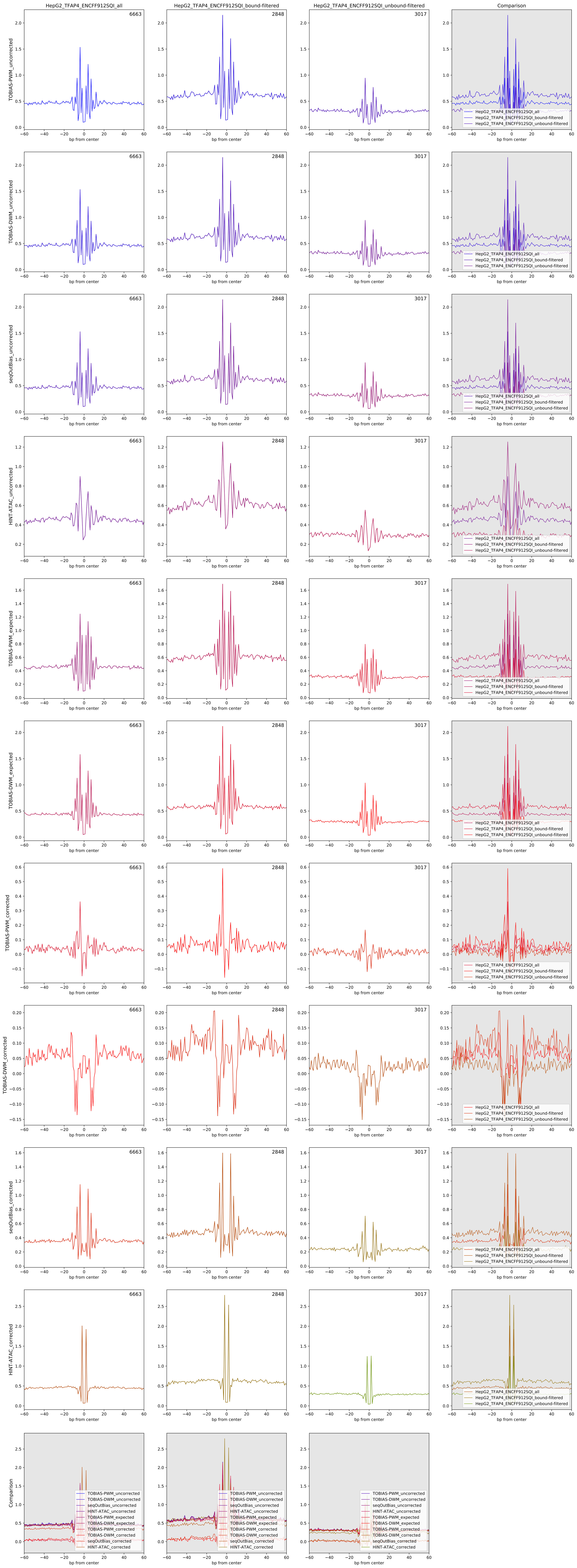

Aggregate footprints in HepG2 for TF TFDP1 (ChIP-seq ENCFF245GRN)

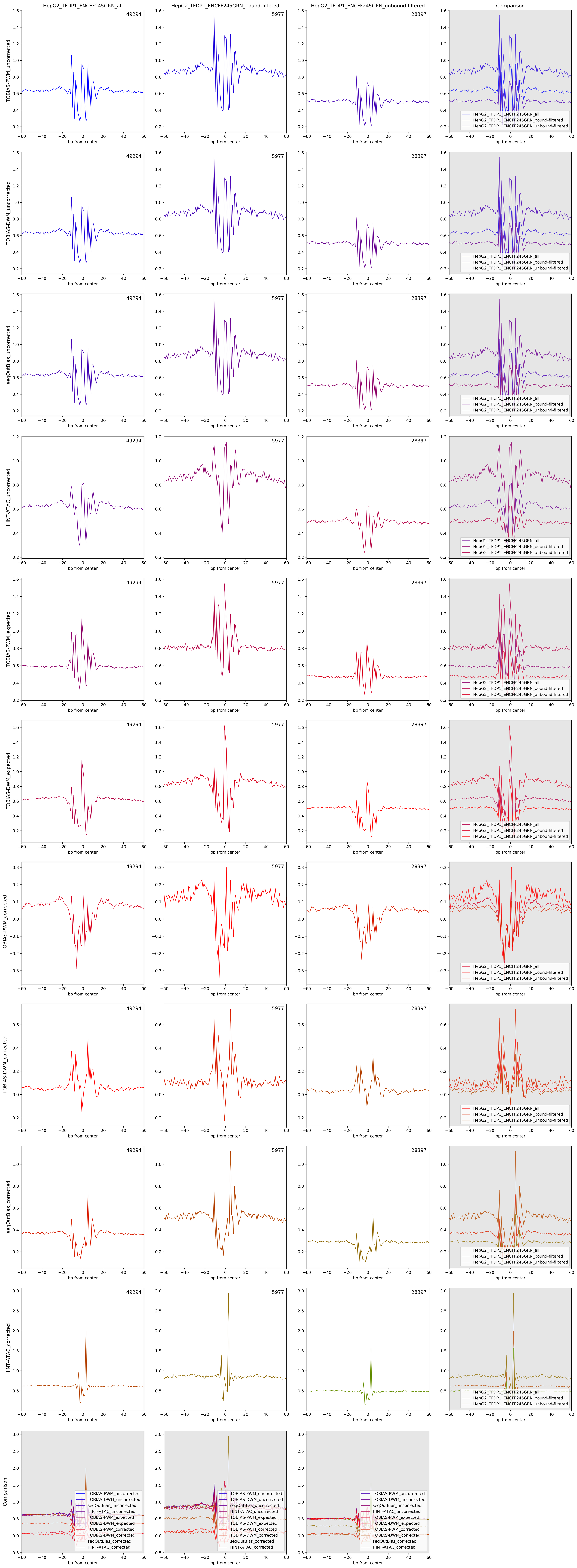

Aggregate footprints in HepG2 for TF TFE3 (ChIP-seq ENCFF277NOY)

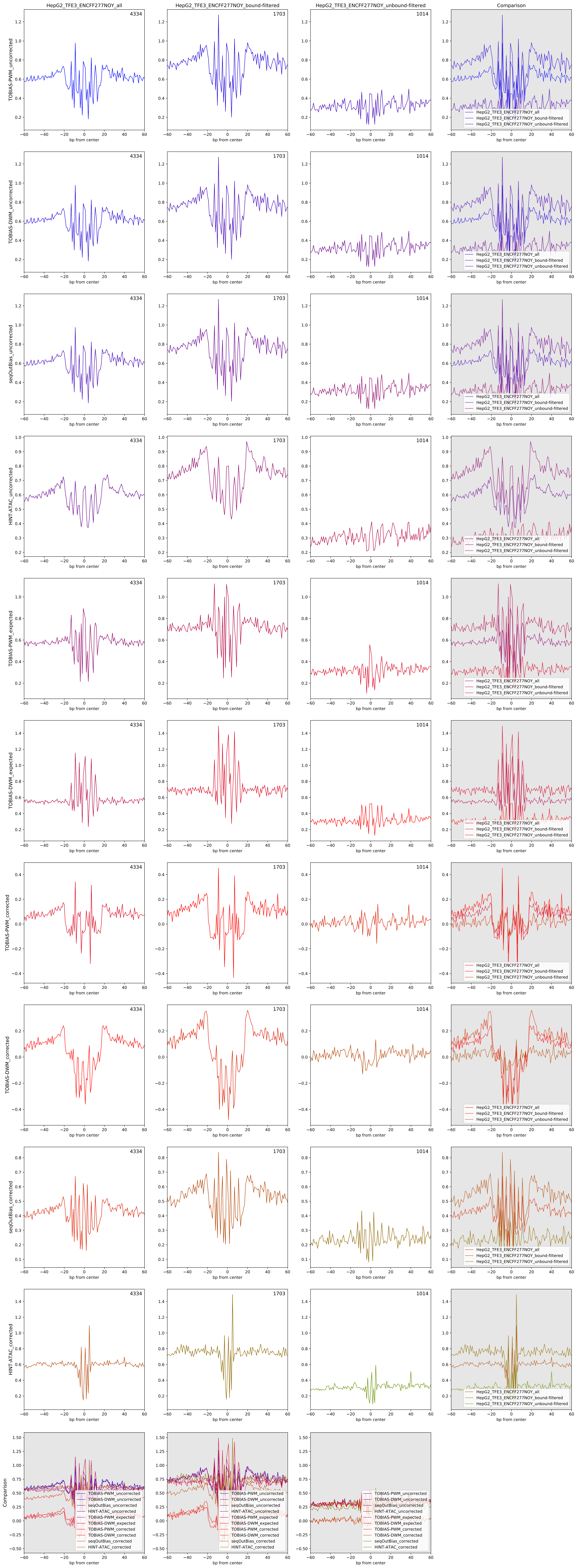

Aggregate footprints in HepG2 for TF TGIF2 (ChIP-seq ENCFF661WFA)

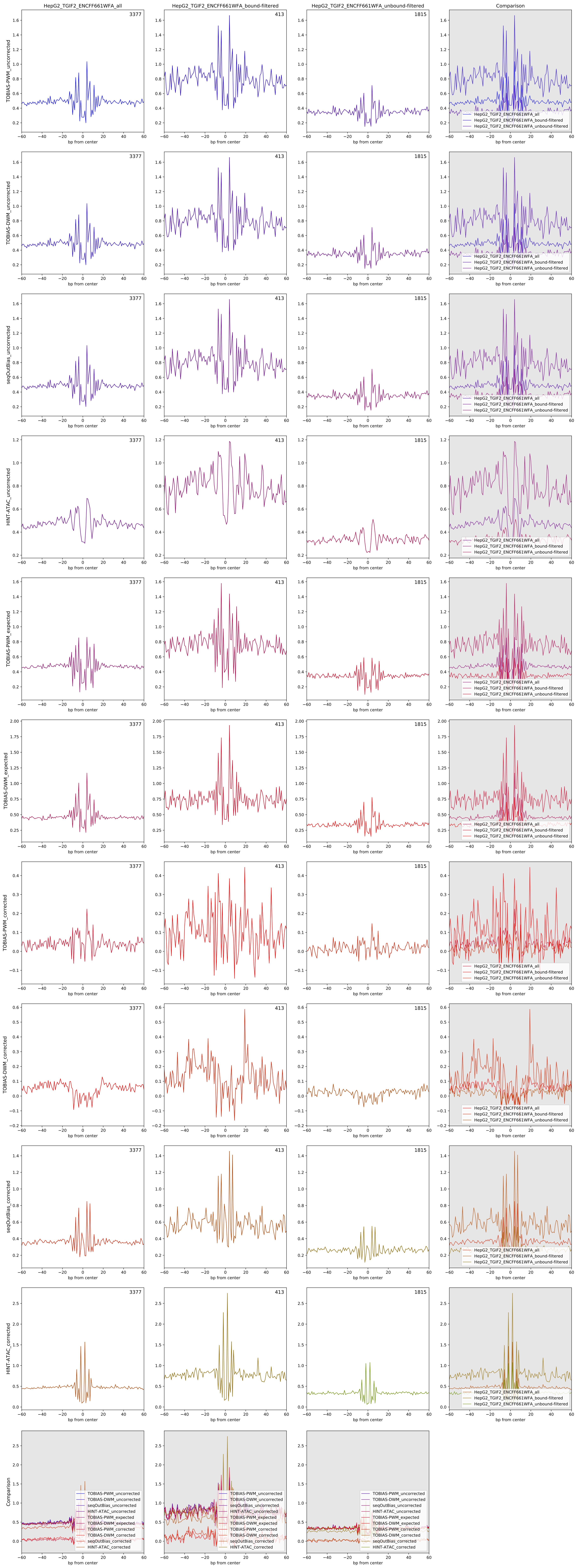

Aggregate footprints in HepG2 for TF USF1 (ChIP-seq ENCFF914IFQ)

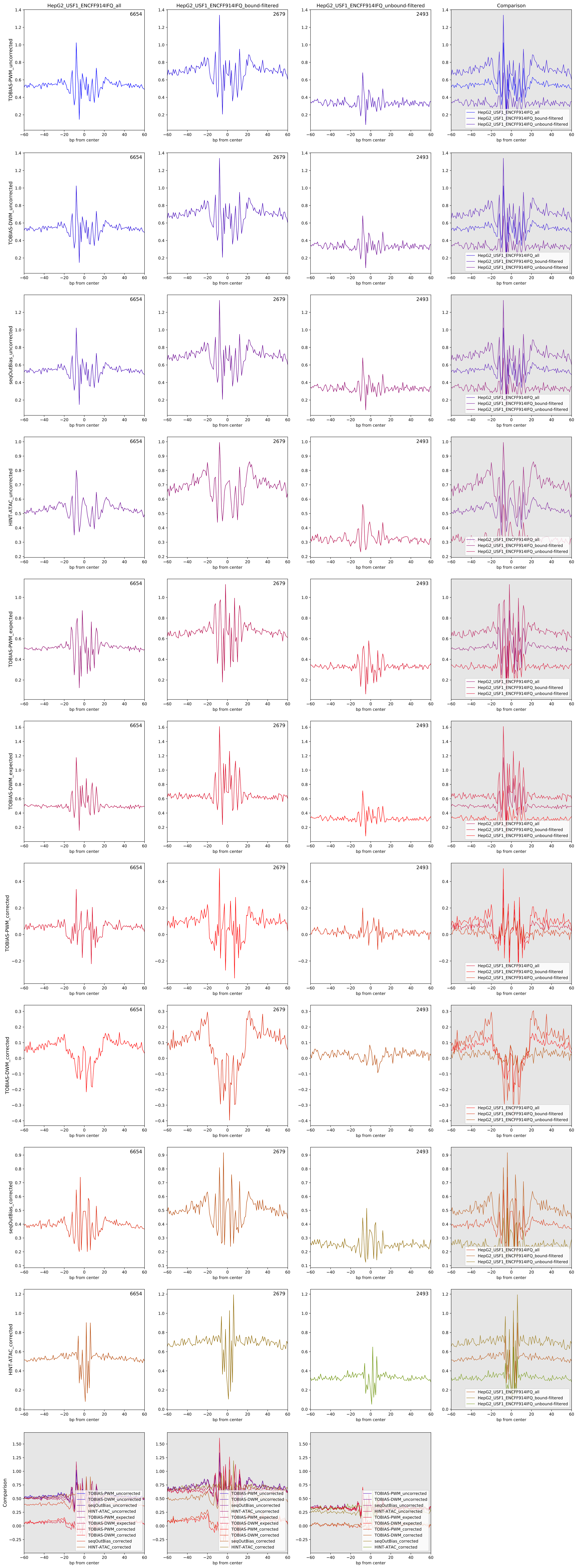

Aggregate footprints in HepG2 for TF YY1 (ChIP-seq ENCFF177YDT)

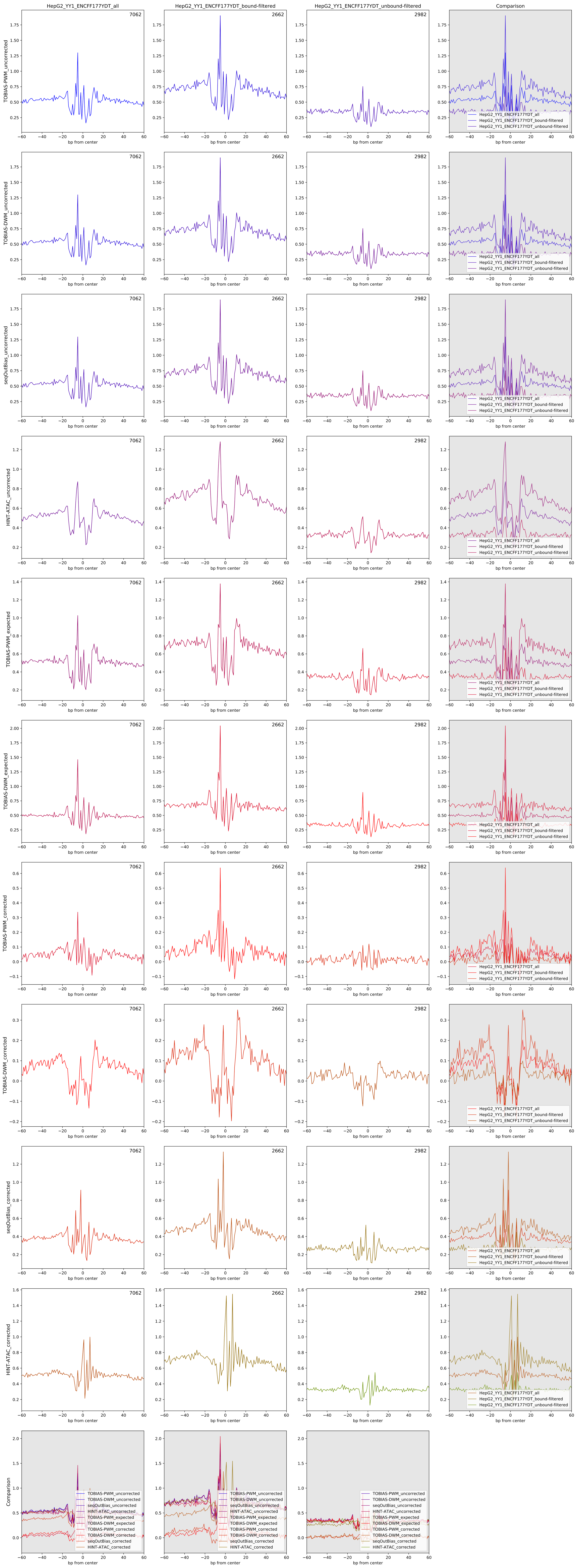

Aggregate footprints in HepG2 for TF ZBTB33 (ChIP-seq ENCFF943WRA)

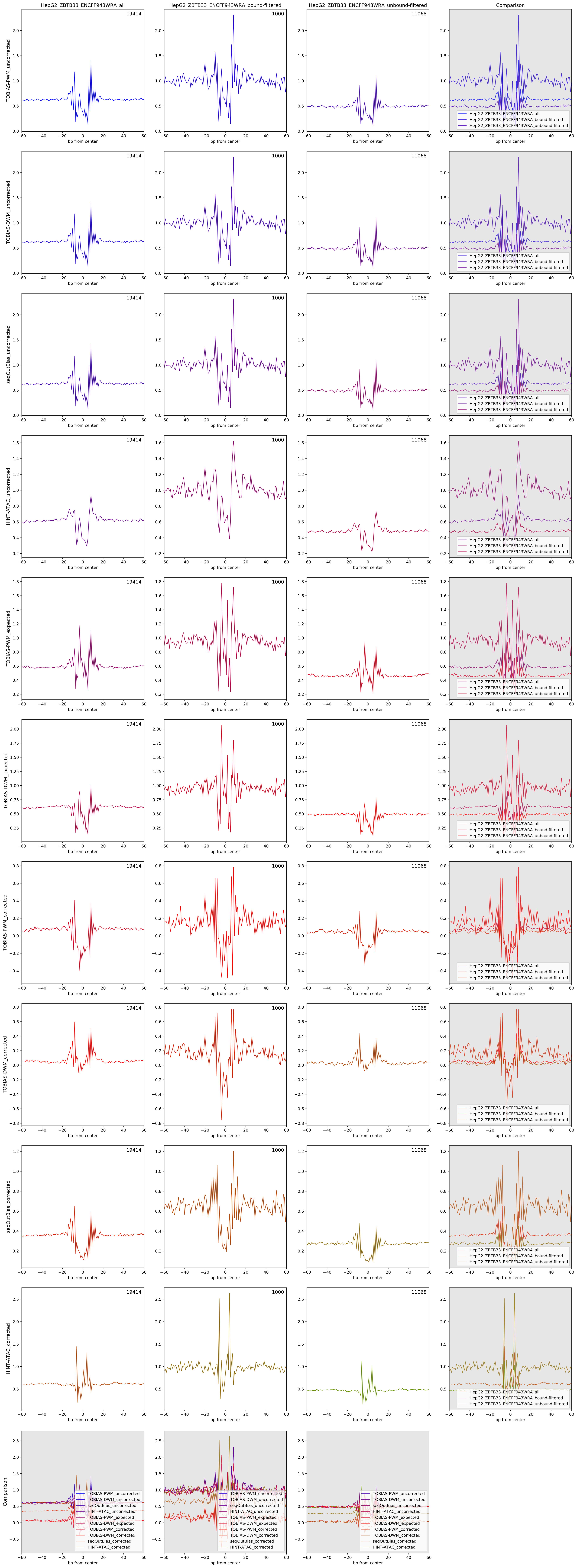

Aggregate footprints in HepG2 for TF ZNF24 (ChIP-seq ENCFF904QAD)

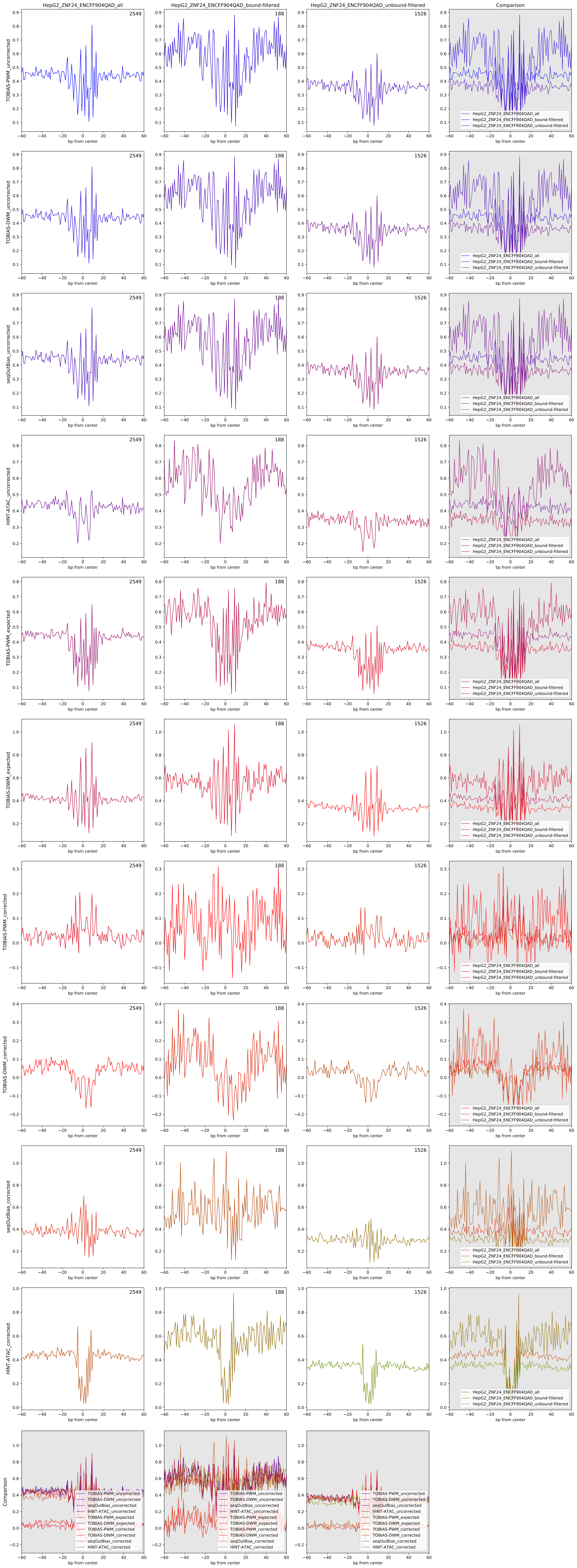

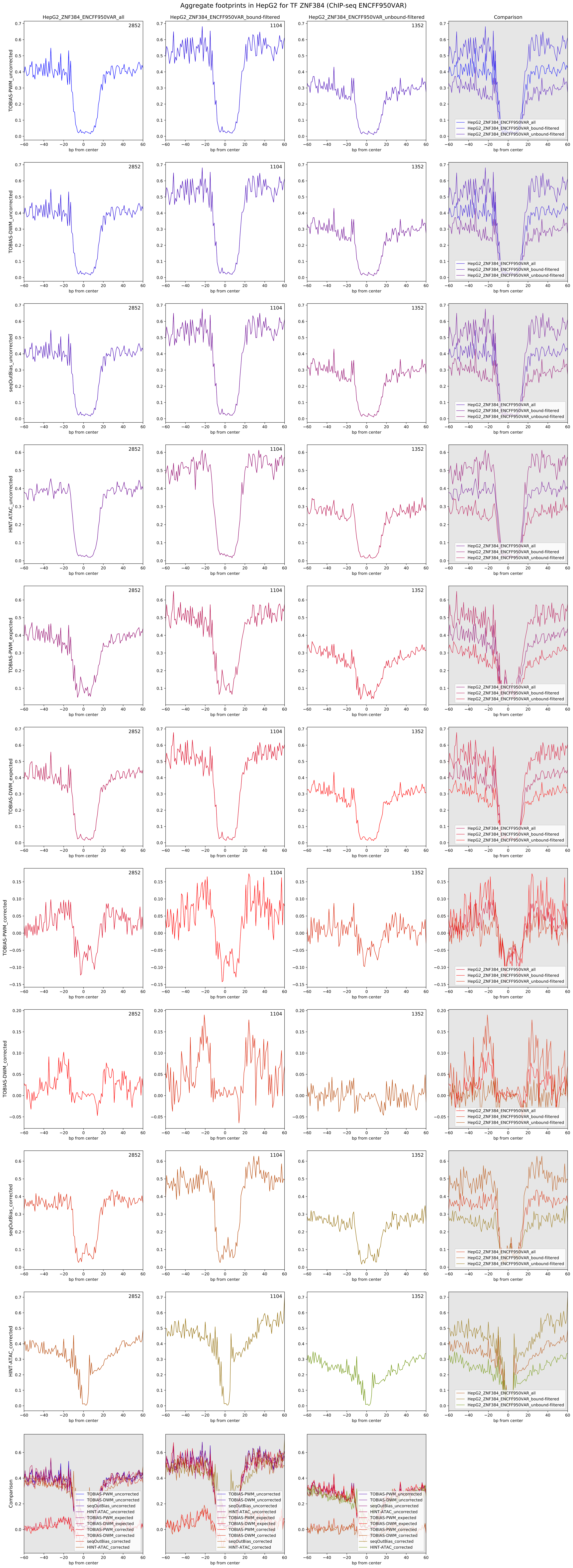

Aggregate footprints in HepG2 for TF ZNF384 (ChIP-seq ENCFF950VAR)
HepG2_ZNF384_ENCFF950VAR_all
HepG2_ZNF384_ENCFF950VAR_bound-filtered
HepG2_ZNF384_ENCFF950VAR_unbound-filtered
Comparison
TOBIAS-PWM_uncorrected
TOBIAS-DWM_uncorrected
seqOutBias_uncorrected
HINT-ATAC_uncorrected
TOBIAS-PWM_expected
TOBIAS-DWM_expected
TOBIAS-PWM_corrected
TOBIAS-DWM_corrected
seqOutBias_corrected
HINT-ATAC_corrected
2852
1104
1352
bp from center

Aggregate footprints in K562 for TF ATF1 (ChIP-seq ENCFF715PLB)

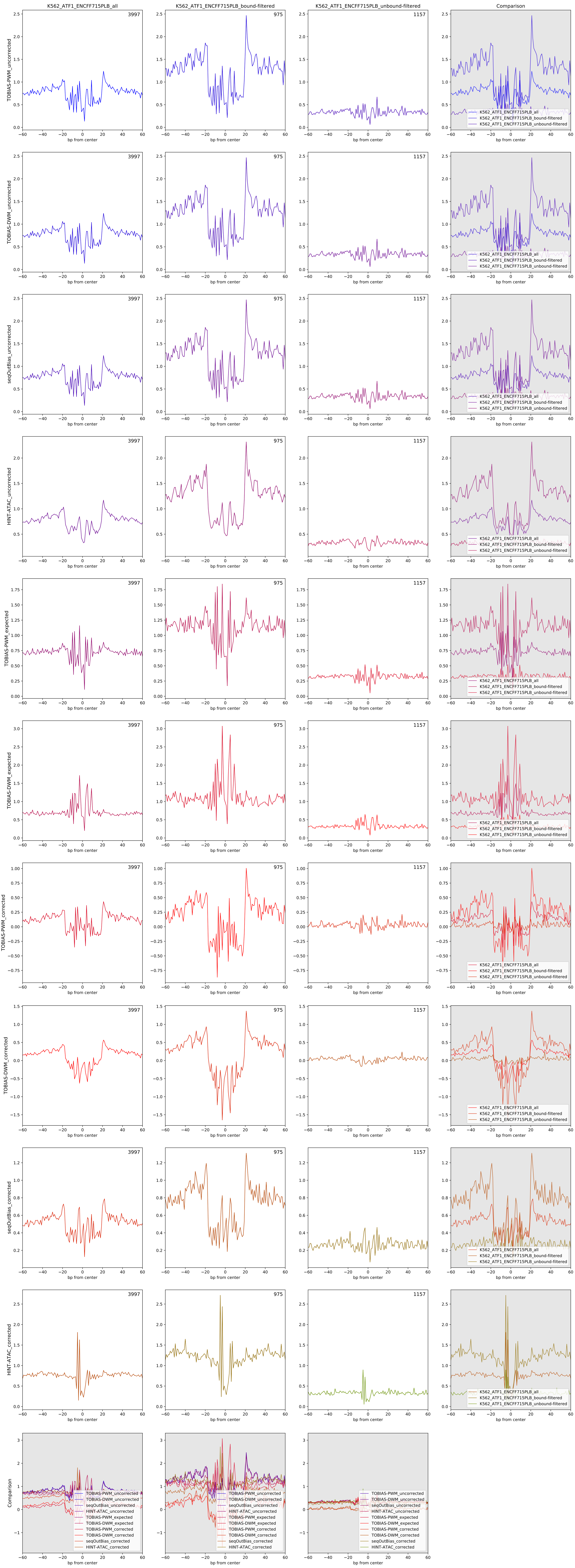

Aggregate footprints in K562 for TF ATF3 (ChIP-seq ENCFF937OKC)

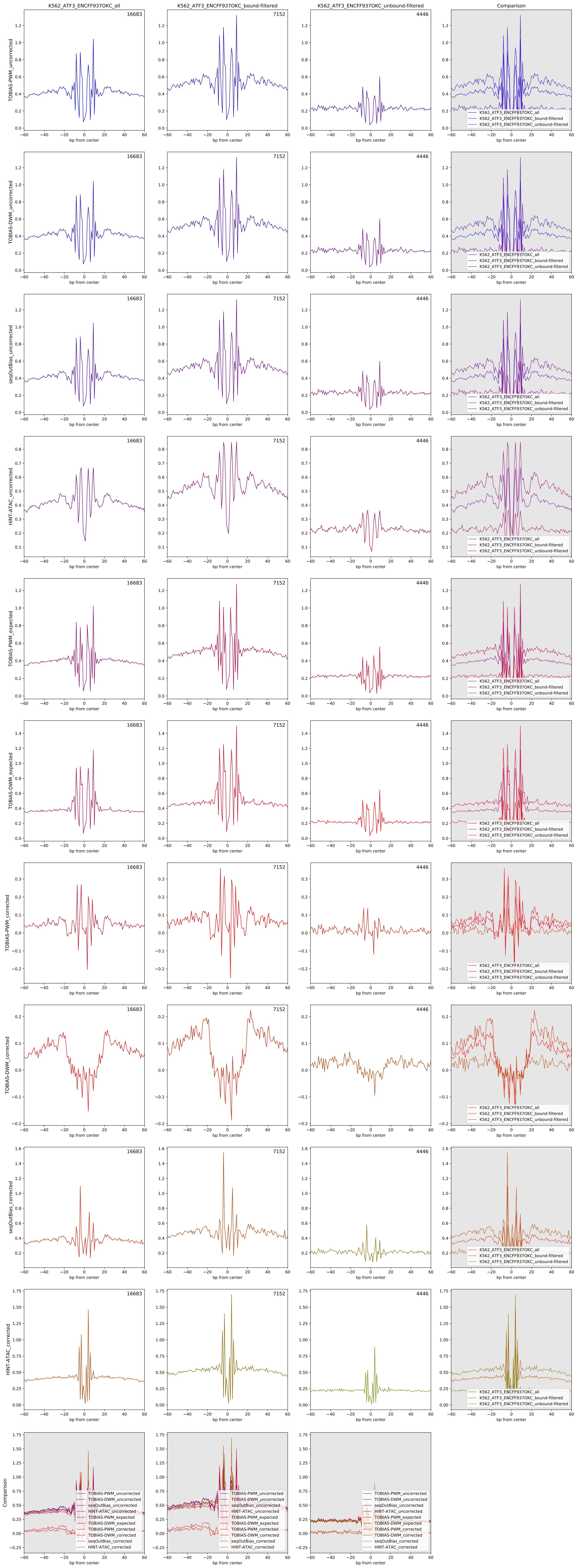

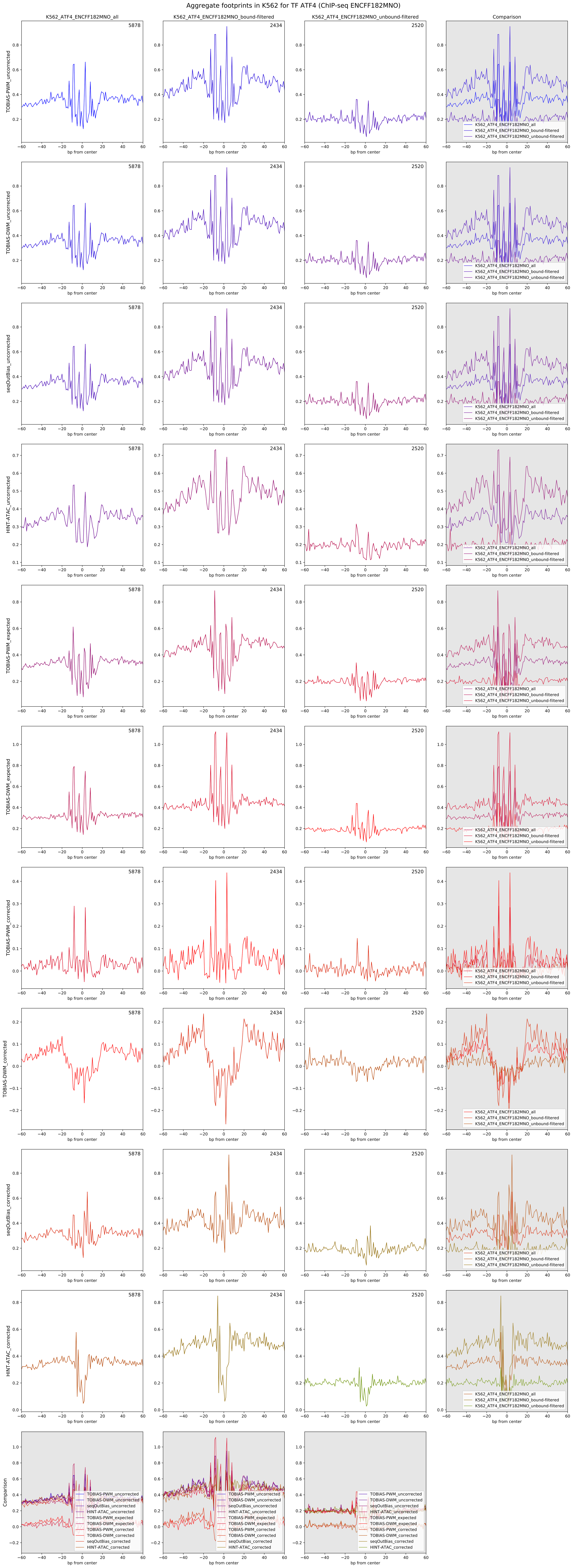

Aggregate footprints in K562 for TF ATF4 (ChIP-seq ENCFF182MNO)
K562_ATF4_ENCFF182MNO_all
K562_ATF4_ENCFF182MNO_bound-filtered
K562_ATF4_ENCFF182MNO_unbound-filtered
Comparison
TOBIAS-PWM_uncorrected
TOBIAS-DWM_uncorrected
seqOutBias_uncorrected
HINT-ATAC_uncorrected
TOBIAS-PWM_expected
TOBIAS-DWM_expected
TOBIAS-PWM_corrected
TOBIAS-DWM_corrected
seqOutBias_corrected
HINT-ATAC_corrected
5878
2434
2520
bp from center

Aggregate footprints in K562 for TF BACH1 (ChIP-seq ENCFF543FNN)

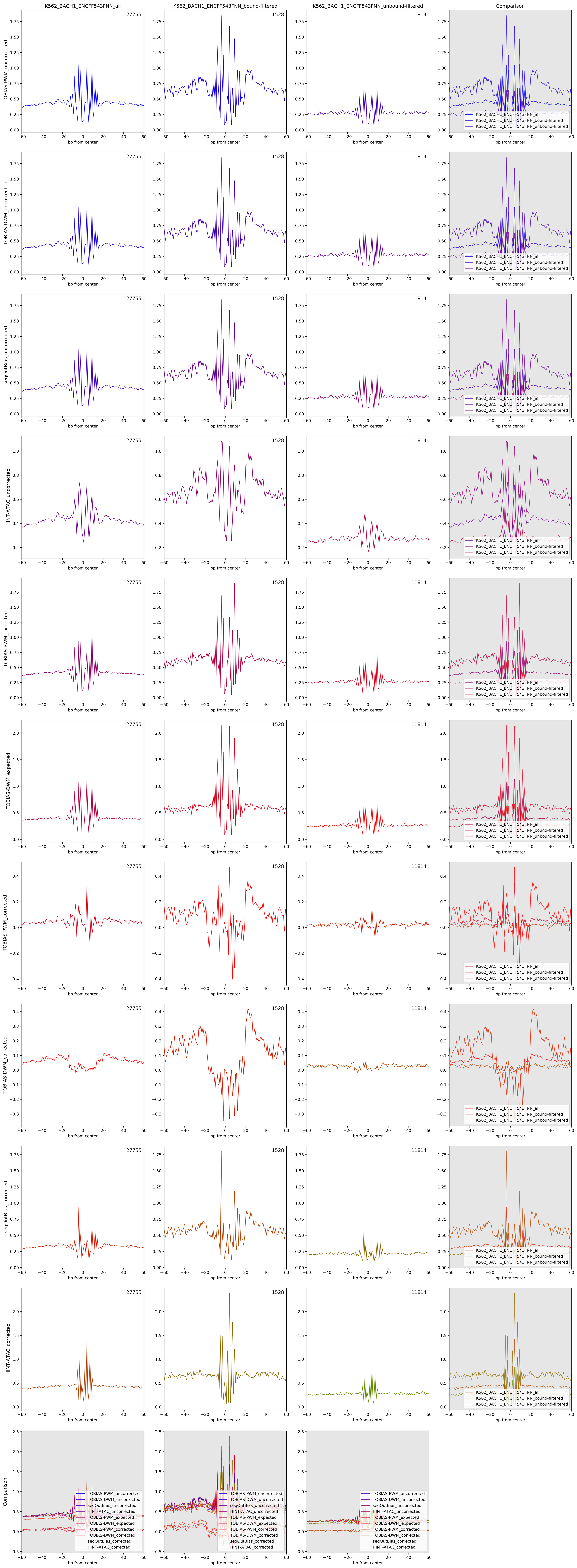

Aggregate footprints in K562 for TF BHLHE40 (ChIP-seq ENCFF477JTV)

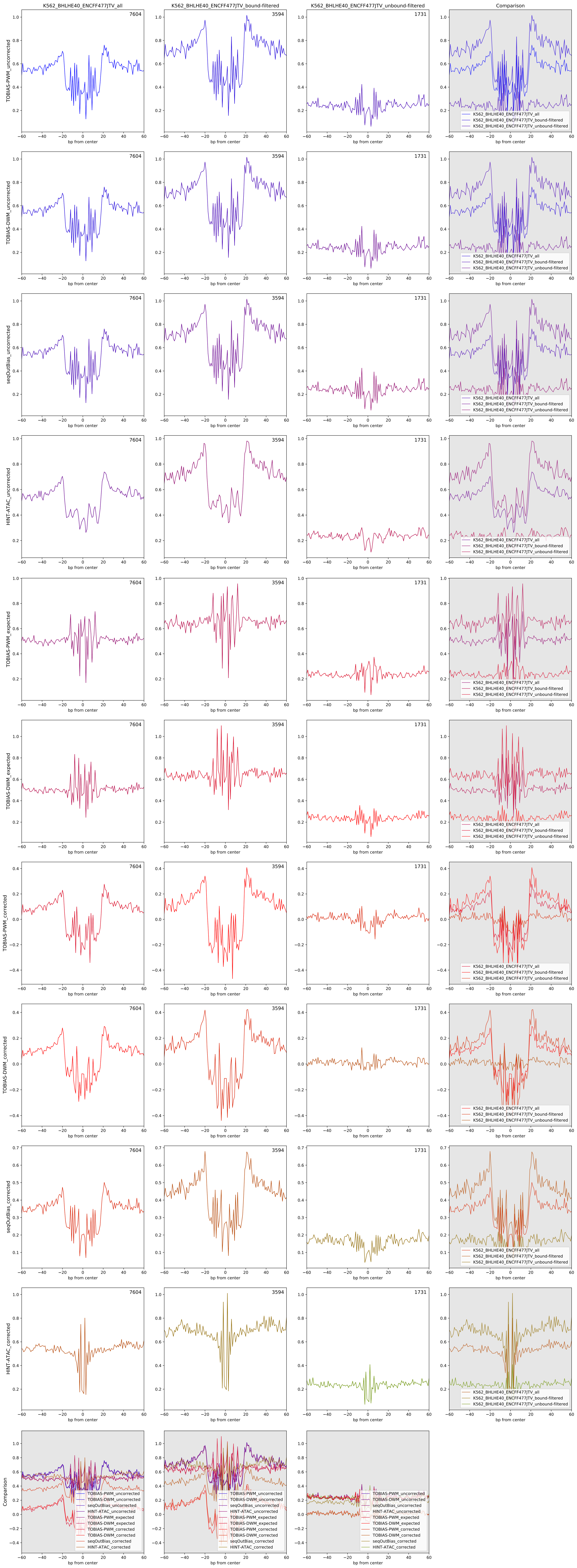

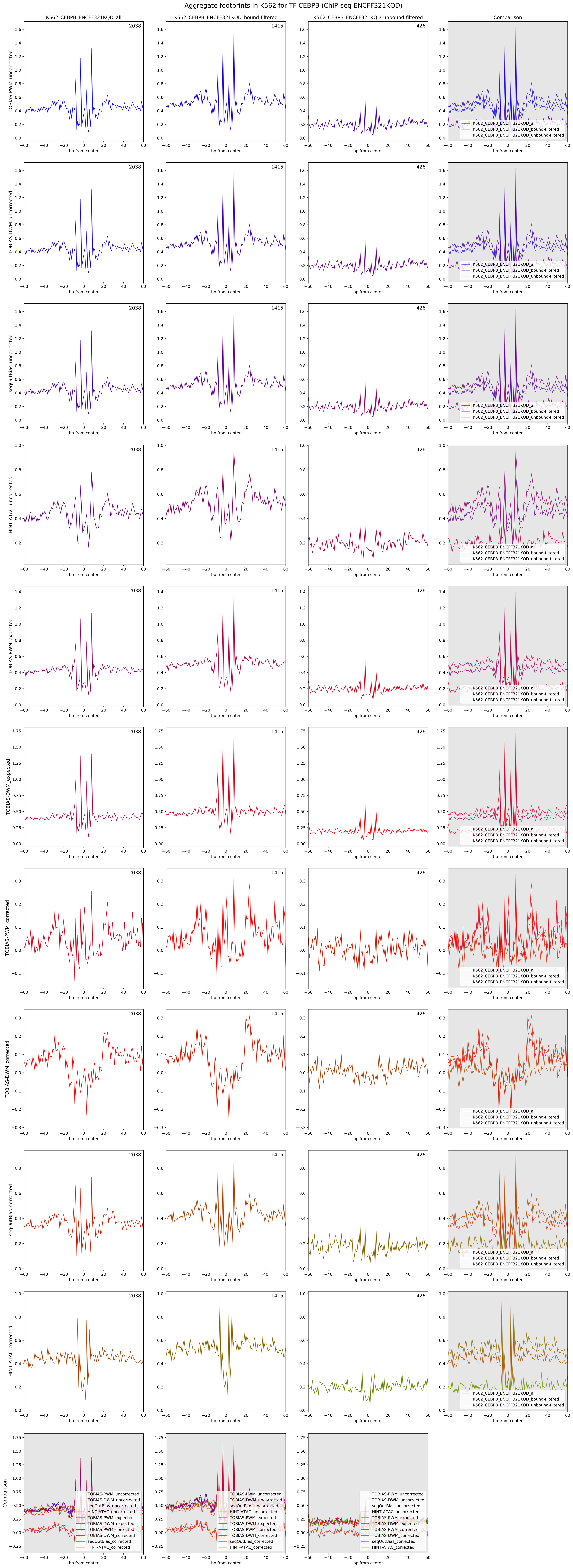
Aggregate footprints in K562 for TF CEBPB (ChIP-seq ENCFF321KQD)
K562_CEBPB_ENCFF321KQD_all
K562_CEBPB_ENCFF321KQD_bound-filtered
K562_CEBPB_ENCFF321KQD_unbound-filtered
Comparison
TOBIAS-PWM_uncorrected
TOBIAS-DWM_uncorrected
seqOutBias_uncorrected
HINT-ATAC_uncorrected
TOBIAS-PWM_expected
TOBIAS-DWM_expected
TOBIAS-PWM_corrected
TOBIAS-DWM_corrected
seqOutBias_corrected
HINT-ATAC_corrected
2038
1415
426
bp from center

Aggregate footprints in K562 for TF CEBPG (ChIP-seq ENCFF797MRW)

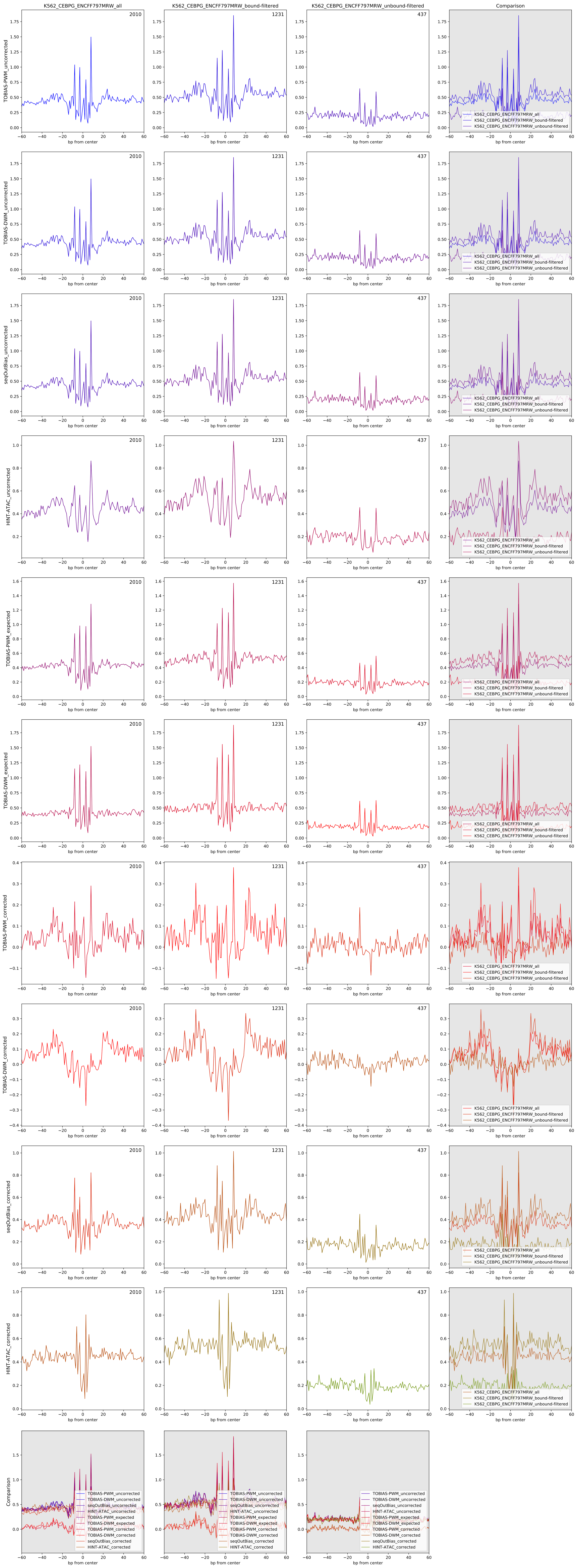

Aggregate footprints in K562 for TF CREB3L1 (ChIP-seq ENCFF566HGU)

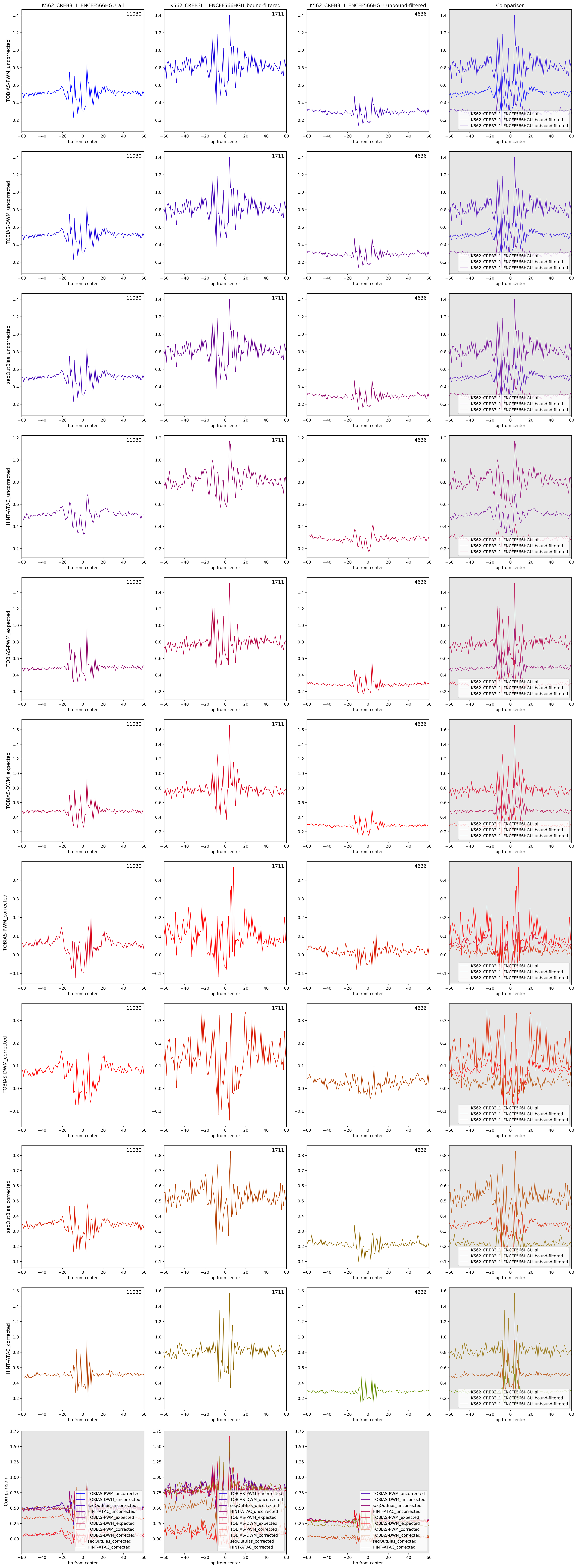

Aggregate footprints in K562 for TF CREM (ChIP-seq ENCFF021XJN)

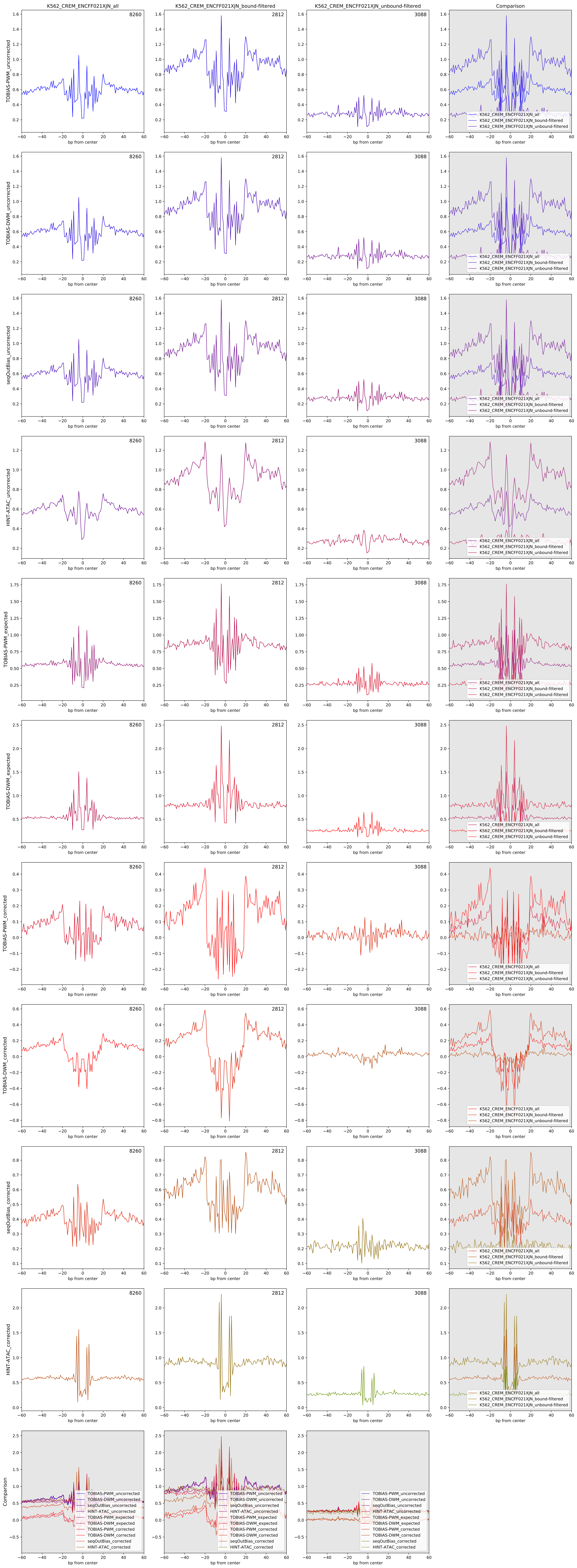

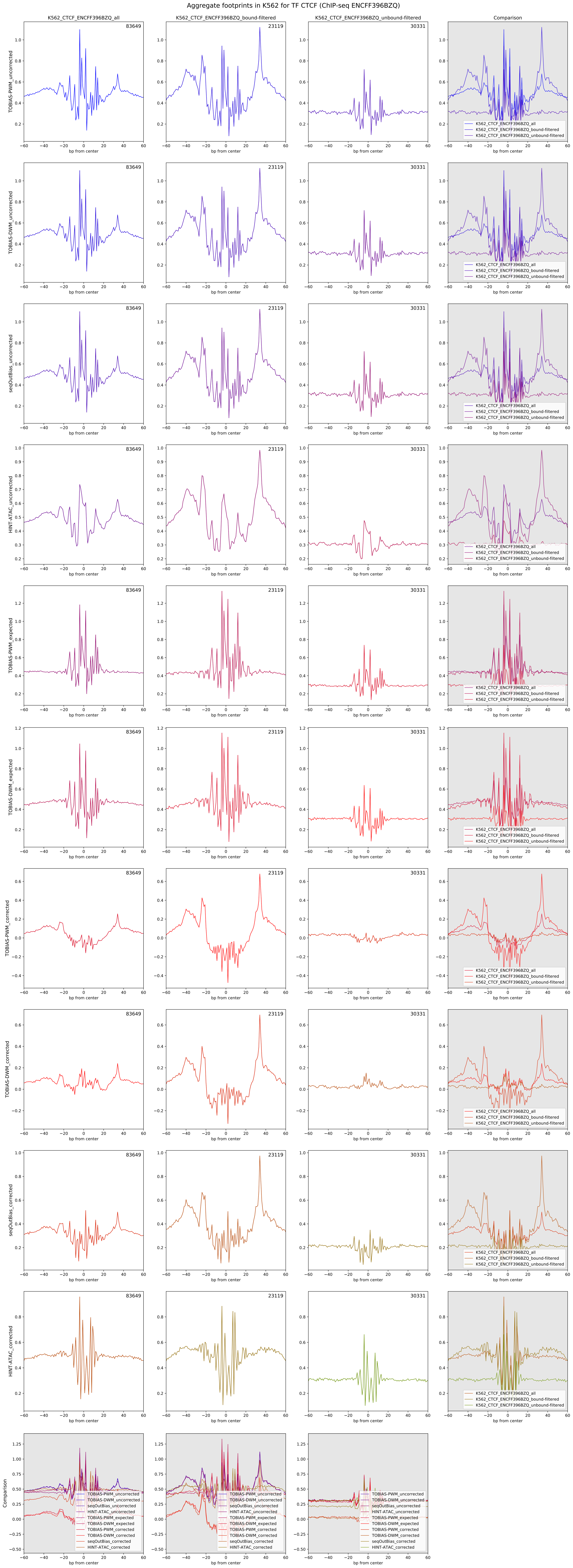

Aggregate footprints in K562 for TF CTCF (ChIP-seq ENCFF396BZQ)
K562_CTCF_ENCFF396BZQ_all
K562_CTCF_ENCFF396BZQ_bound-filtered
K562_CTCF_ENCFF396BZQ_unbound-filtered
Comparison
TOBIAS-PWM_uncorrected
TOBIAS-DWM_uncorrected
seqOutBias_uncorrected
HINT-ATAC_uncorrected
TOBIAS-PWM_expected
TOBIAS-DWM_expected
TOBIAS-PWM_corrected
TOBIAS-DWM_corrected
seqOutBias_corrected
HINT-ATAC_corrected
83649
23119
30331
bp from center

Aggregate footprints in K562 for TF CTCFL (ChIP-seq ENCFF678TFE)

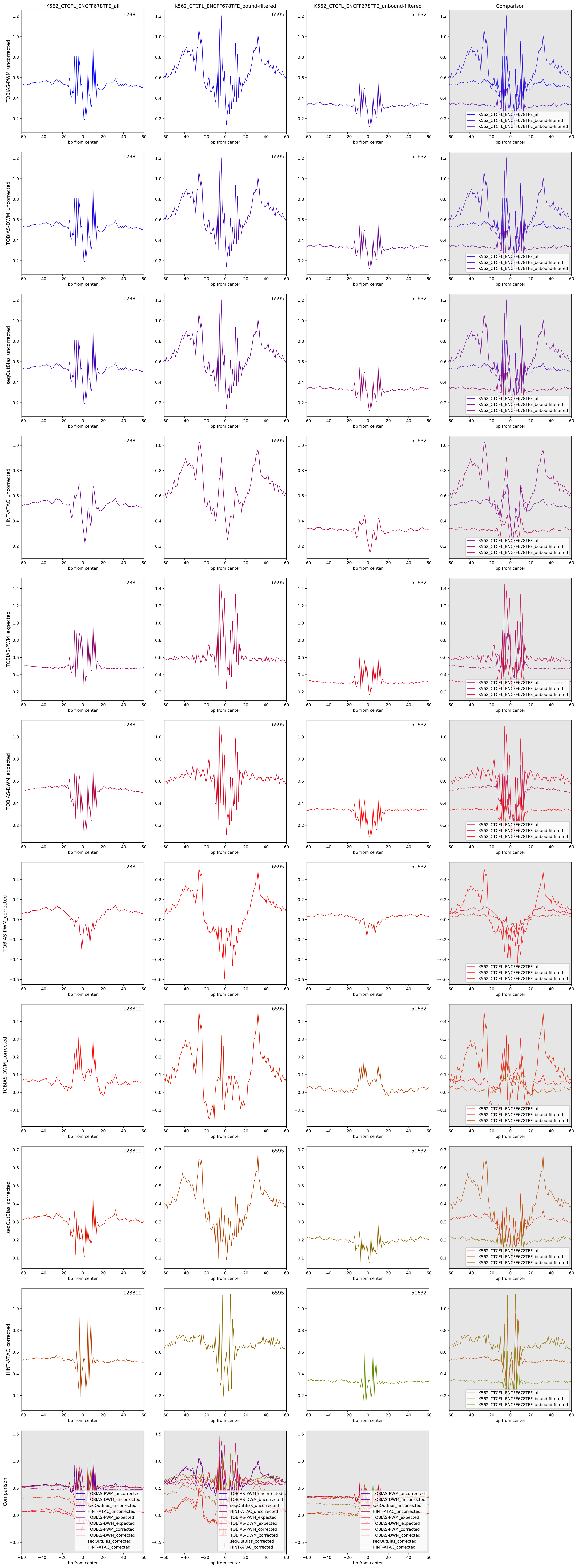

Aggregate footprints in K562 for TF CUX1 (ChIP-seq ENCFF556HMX)

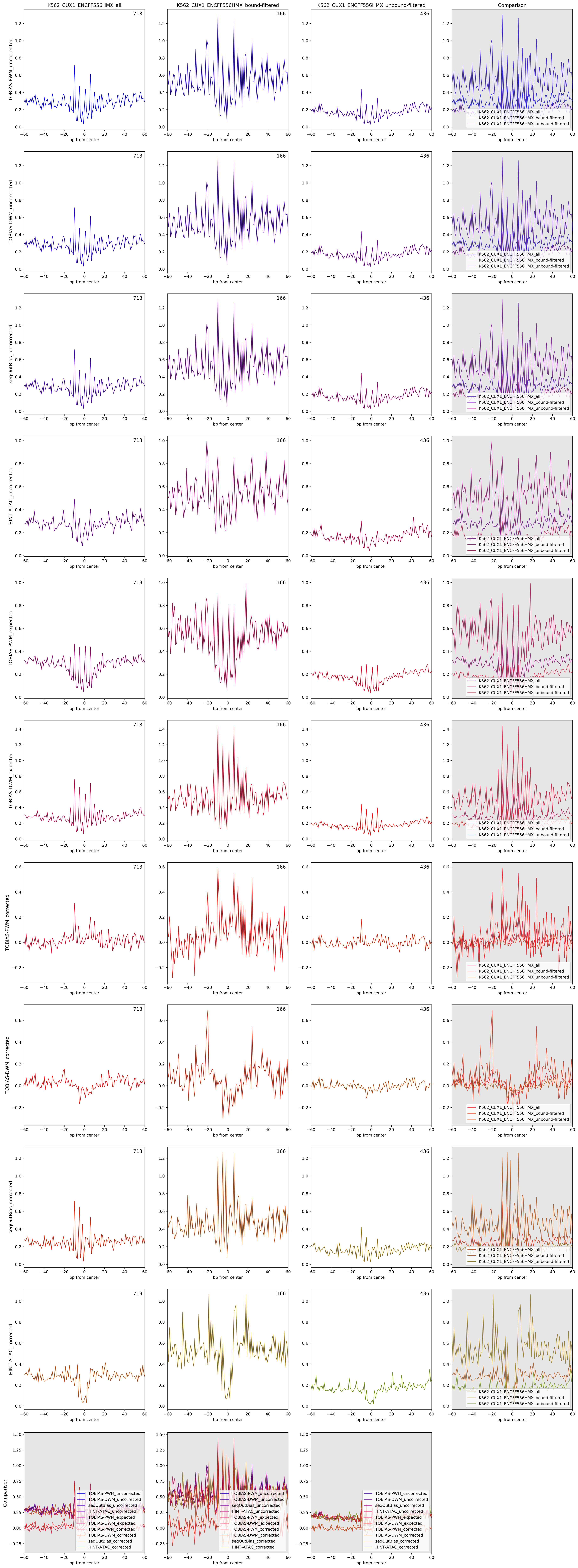

Aggregate footprints in K562 for TF E2F1 (ChIP-seq ENCFF445VTT)

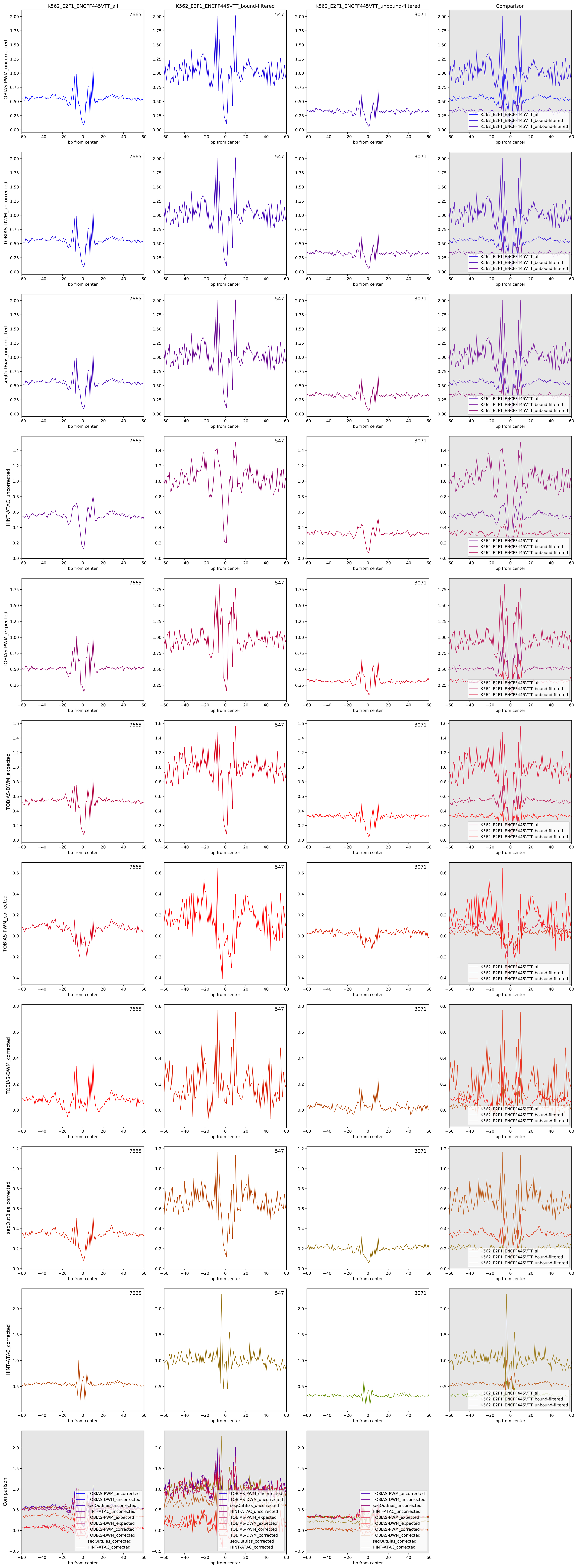

Aggregate footprints in K562 for TF E2F4 (ChIP-seq ENCFF225TLP)

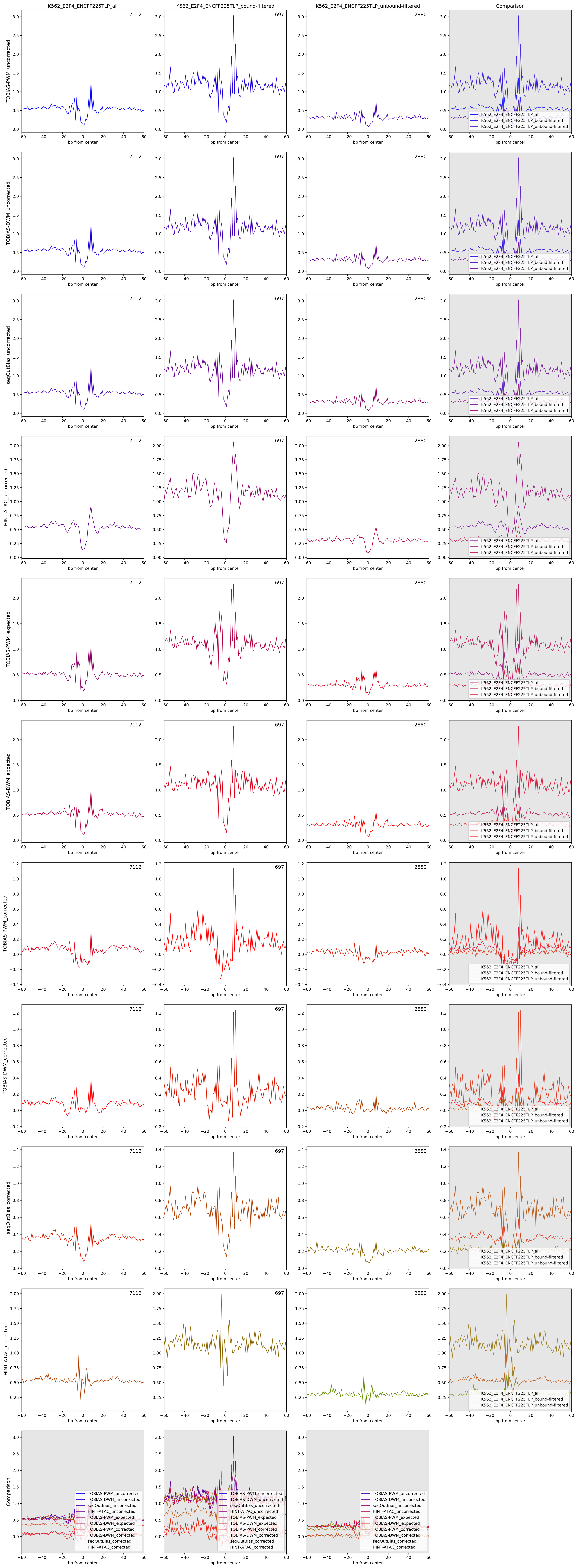

Aggregate footprints in K562 for TF E2F6 (ChIP-seq ENCFF533GSH)

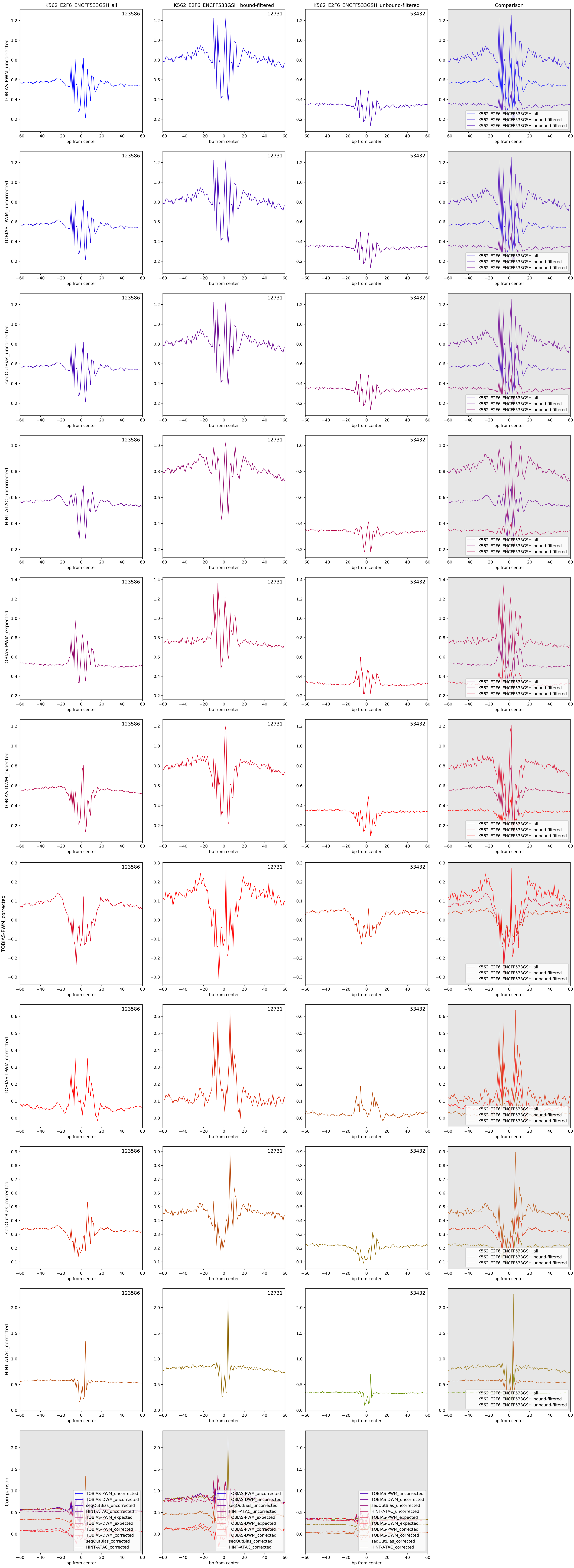

Aggregate footprints in K562 for TF E2F8 (ChIP-seq ENCFF171WWF)

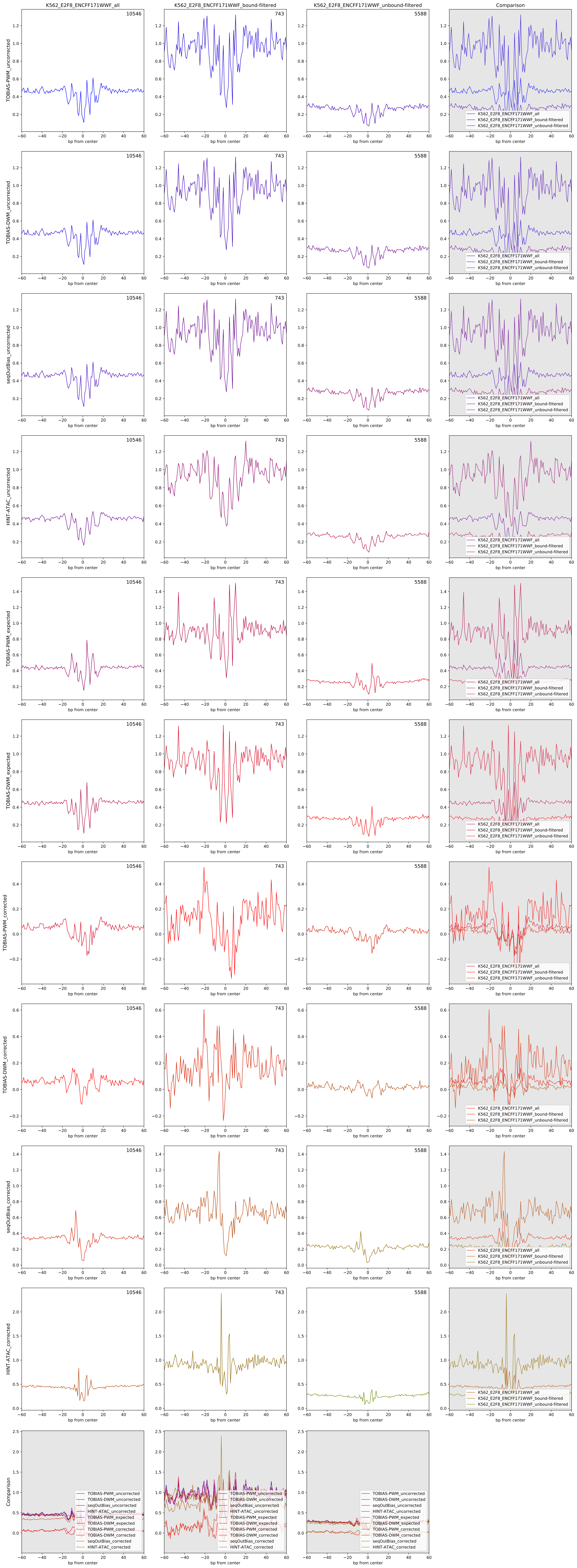

Aggregate footprints in K562 for TF EGR1 (ChIP-seq ENCFF561OGS)

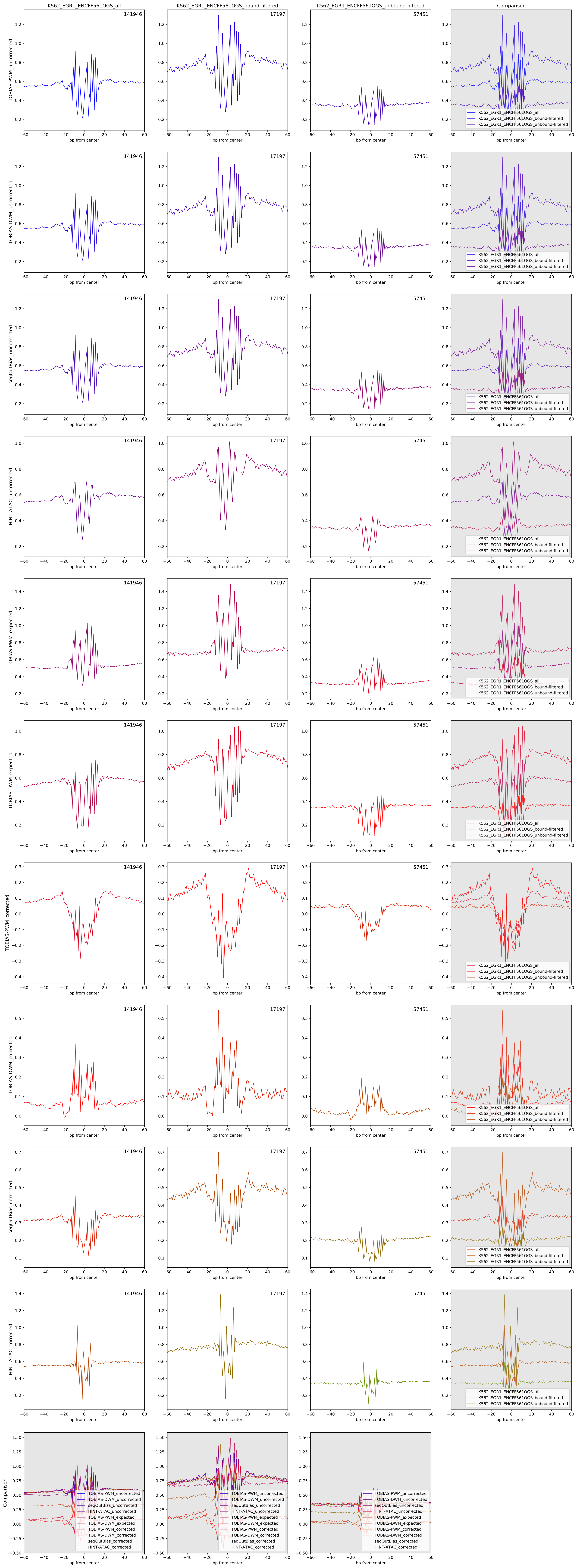

Aggregate footprints in K562 for TF ELF1 (ChIP-seq ENCFF463GCH)

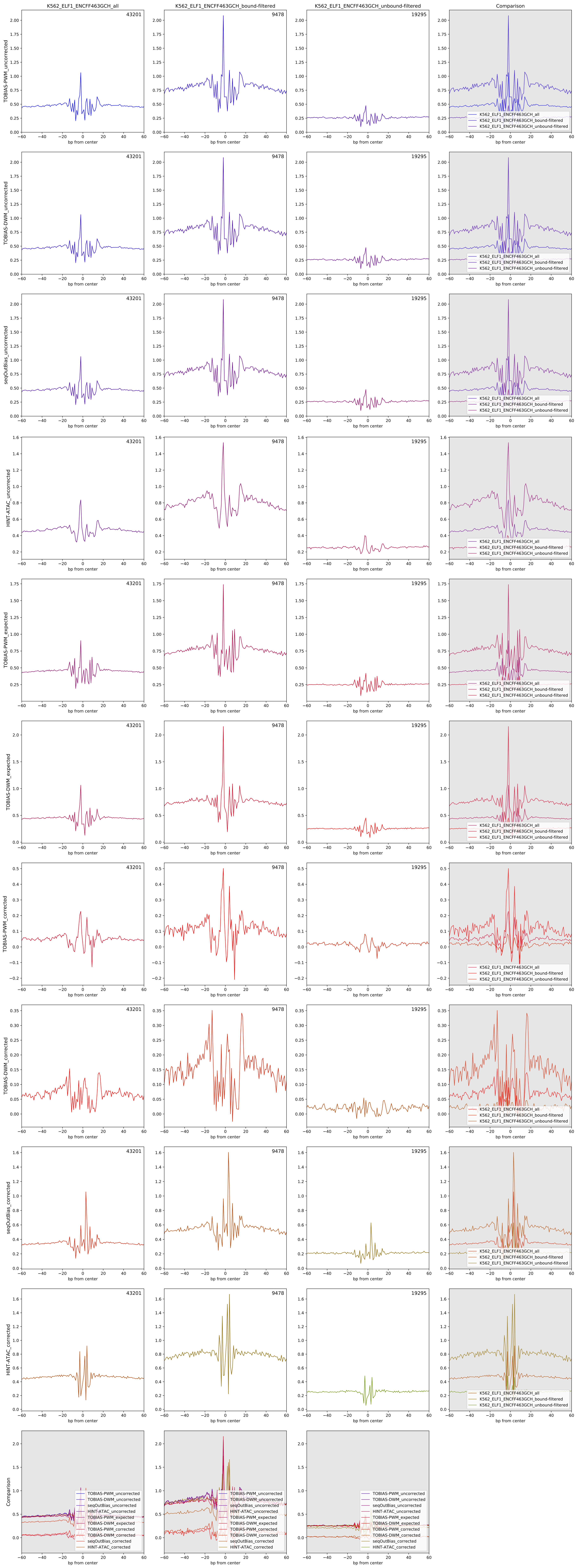

Aggregate footprints in K562 for TF ELF4 (ChIP-seq ENCFF539SXG)

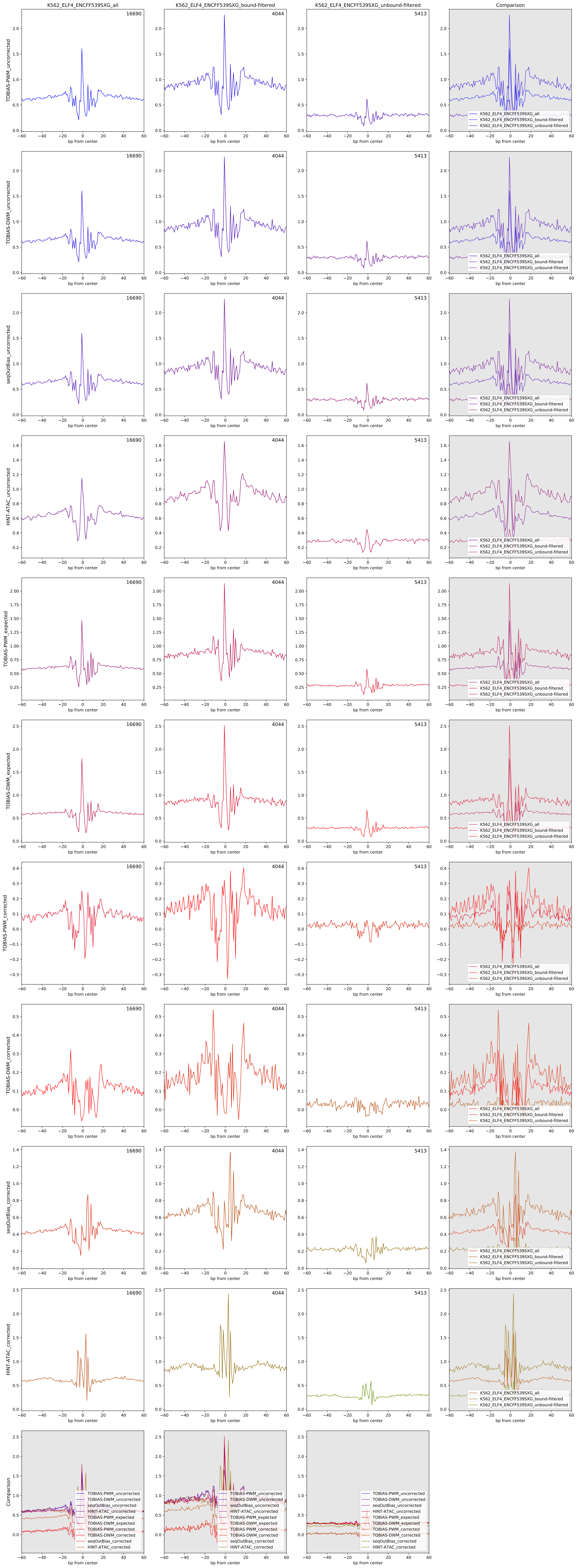

Aggregate footprints in K562 for TF ELK1 (ChIP-seq ENCFF119SCQ)

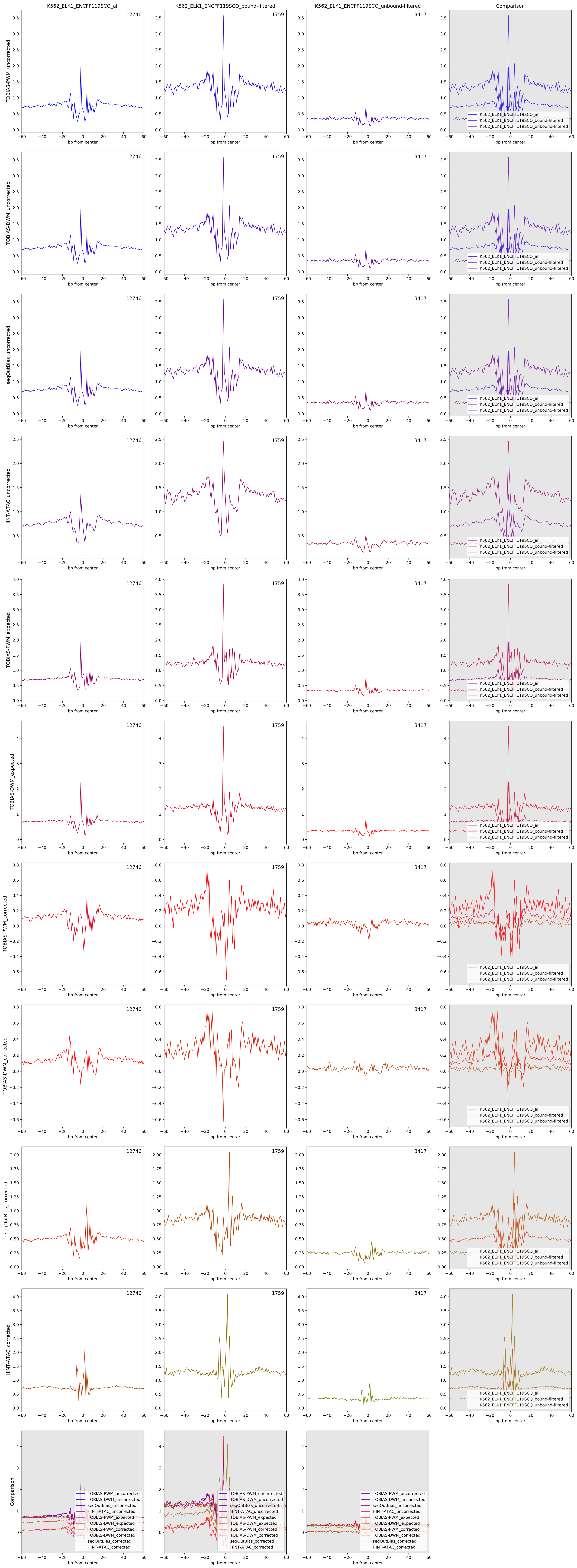

Aggregate footprints in K562 for TF ESRRA (ChIP-seq ENCFF592GWM)

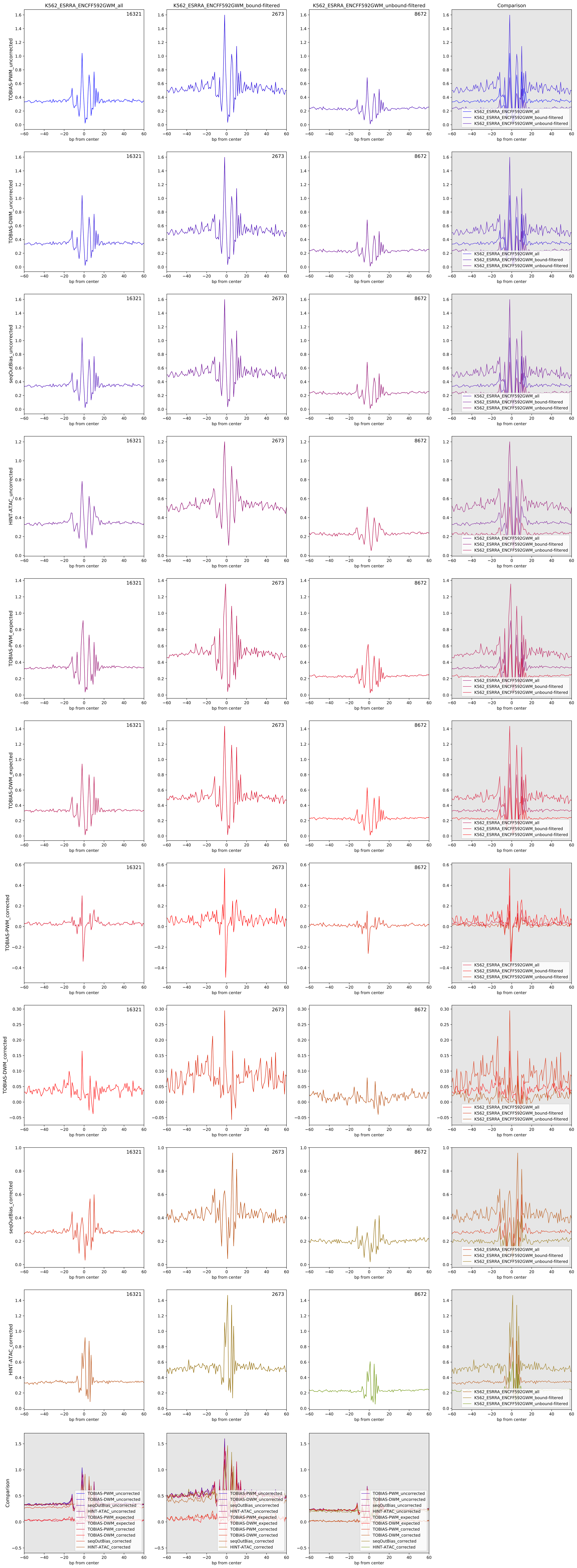

Aggregate footprints in K562 for TF ETV1 (ChIP-seq ENCFF804KSB)

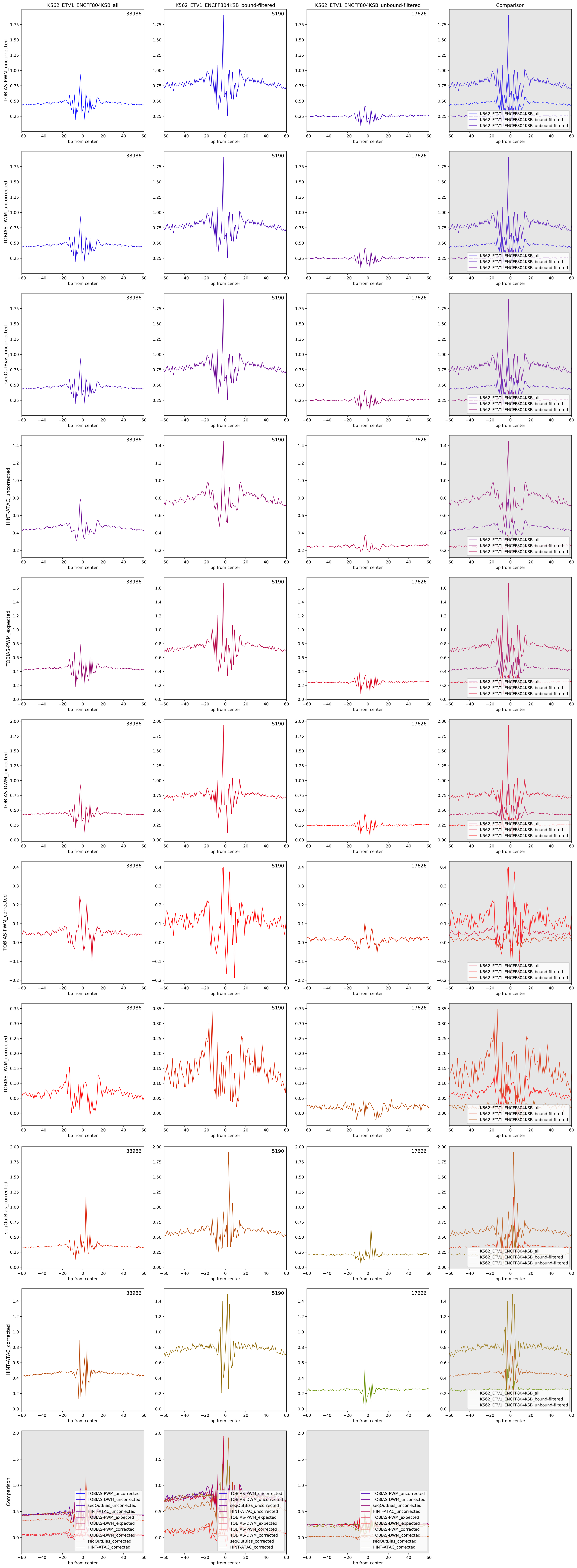

Aggregate footprints in K562 for TF ETV6 (ChIP-seq ENCFF426GSY)

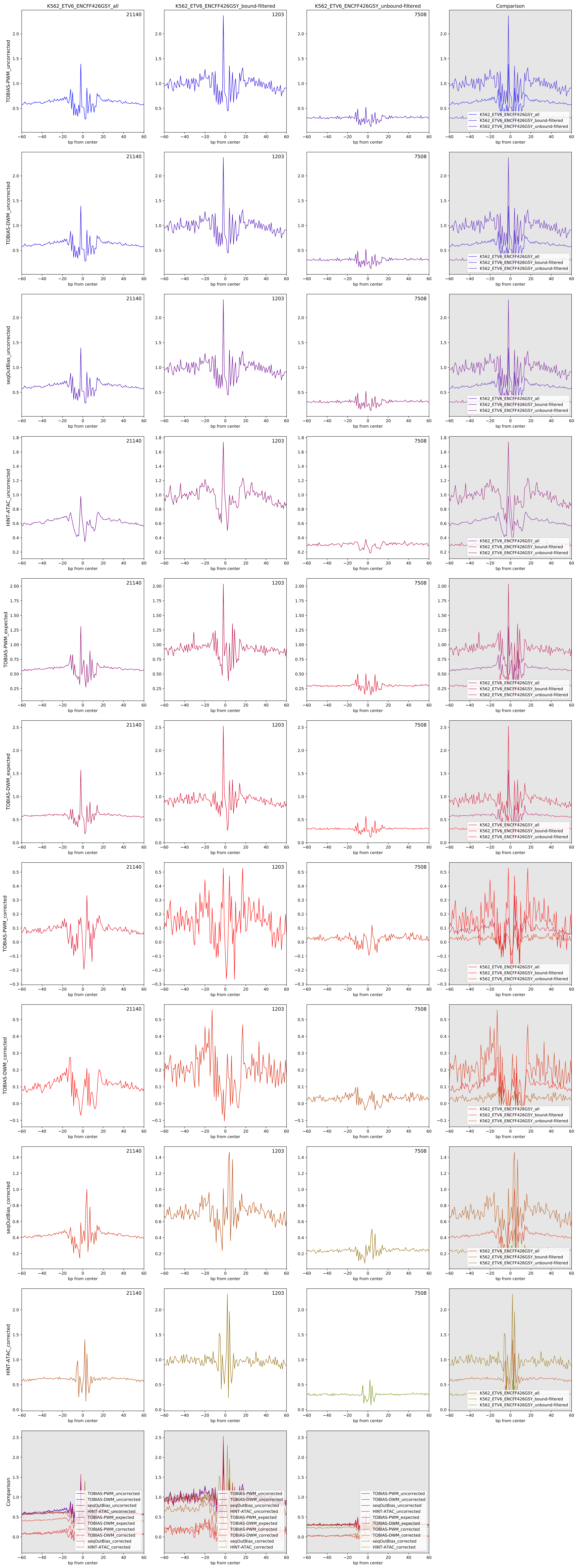

Aggregate footprints in K562 for TF FOSL1 (ChIP-seq ENCFF151FYK)

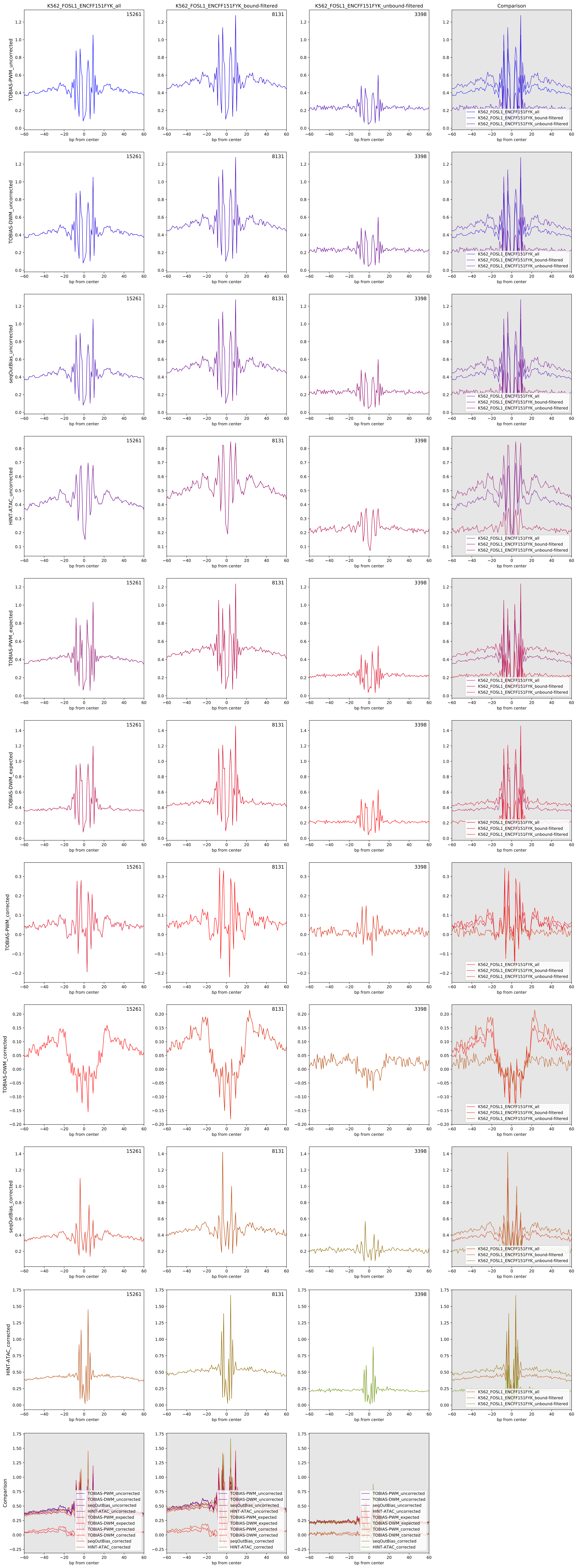

Aggregate footprints in K562 for TF FOXA1 (ChIP-seq ENCFF765NAN)

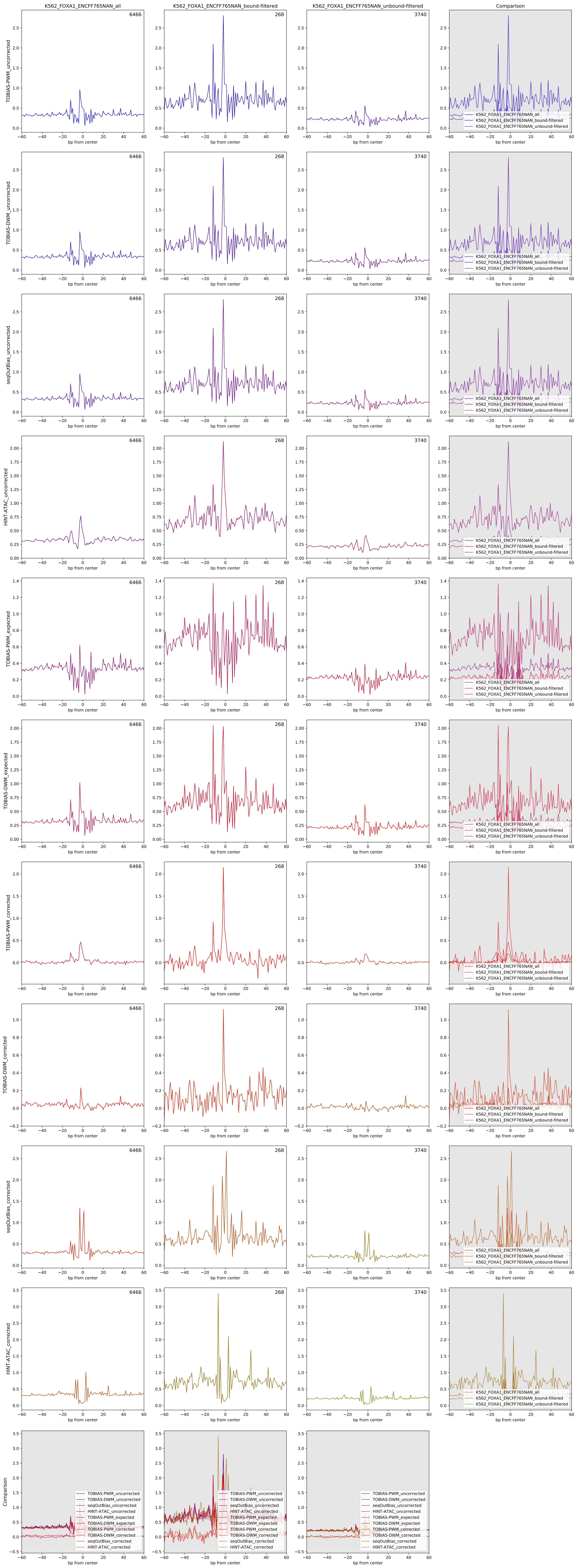

Aggregate footprints in K562 for TF FOXJ2 (ChIP-seq ENCFF175IUD)

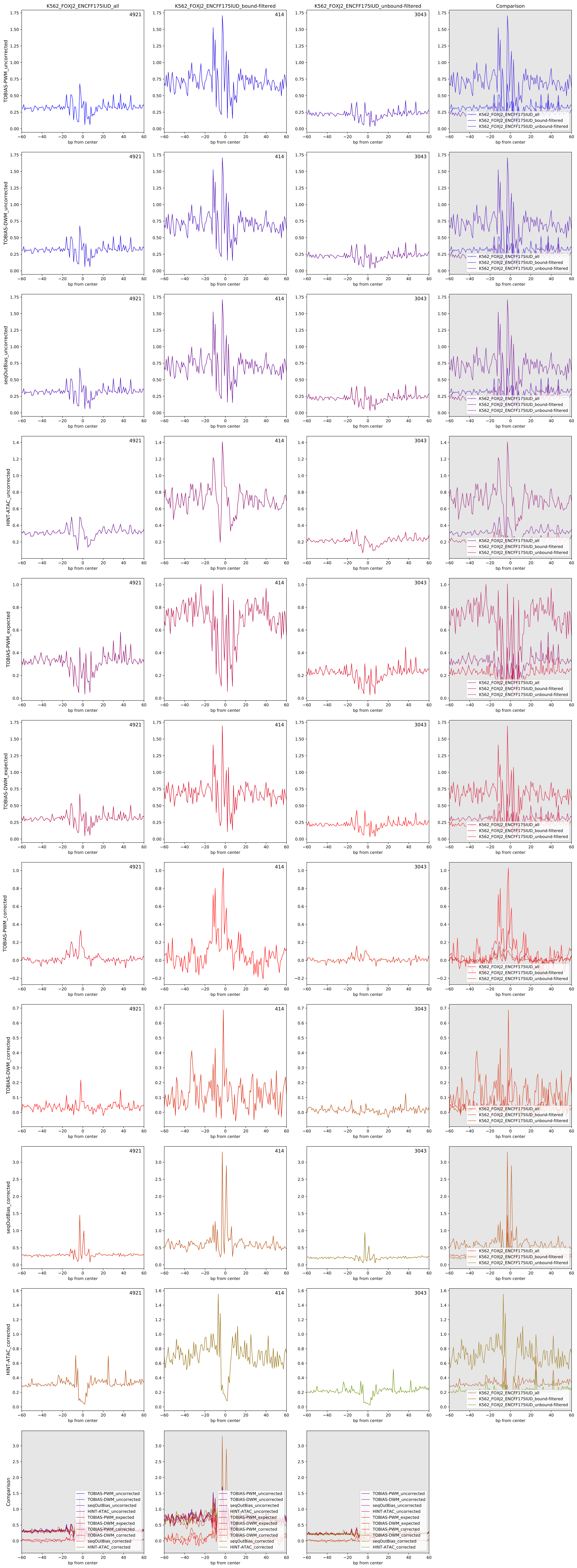

Aggregate footprints in K562 for TF FOXK2 (ChIP-seq ENCFF066CWG)

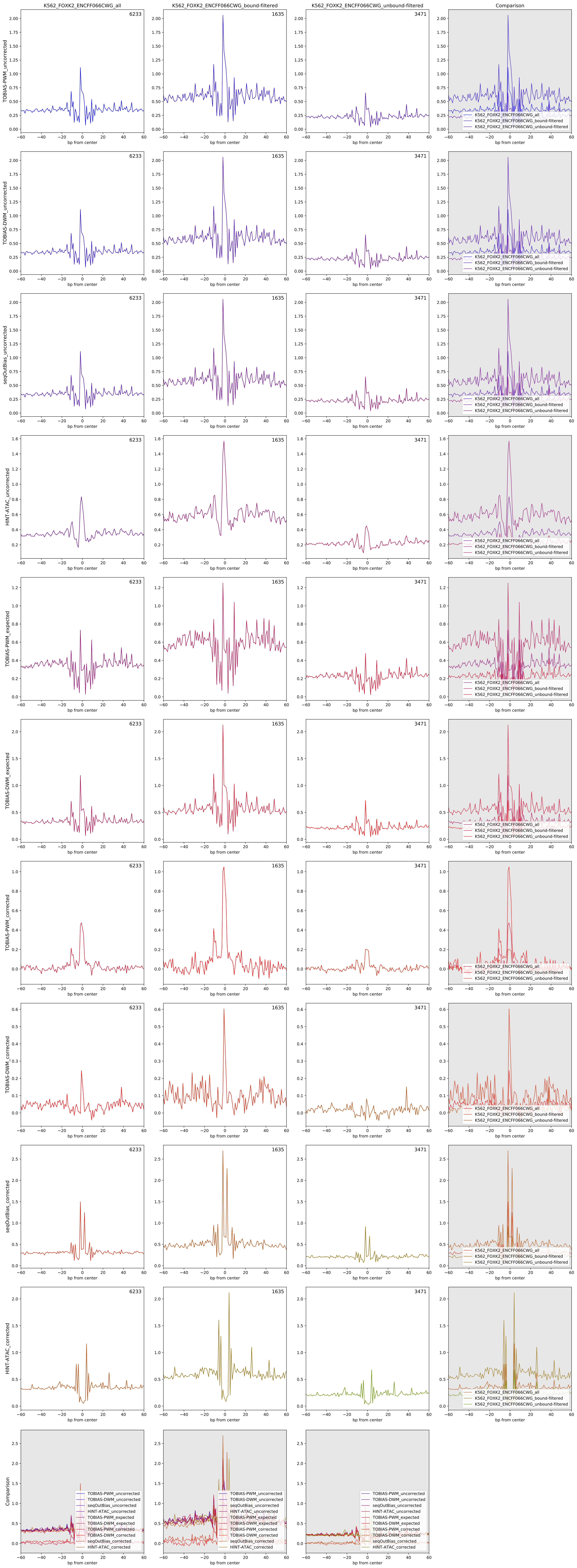

Aggregate footprints in K562 for TF GABPA (ChIP-seq ENCFF535BMV)

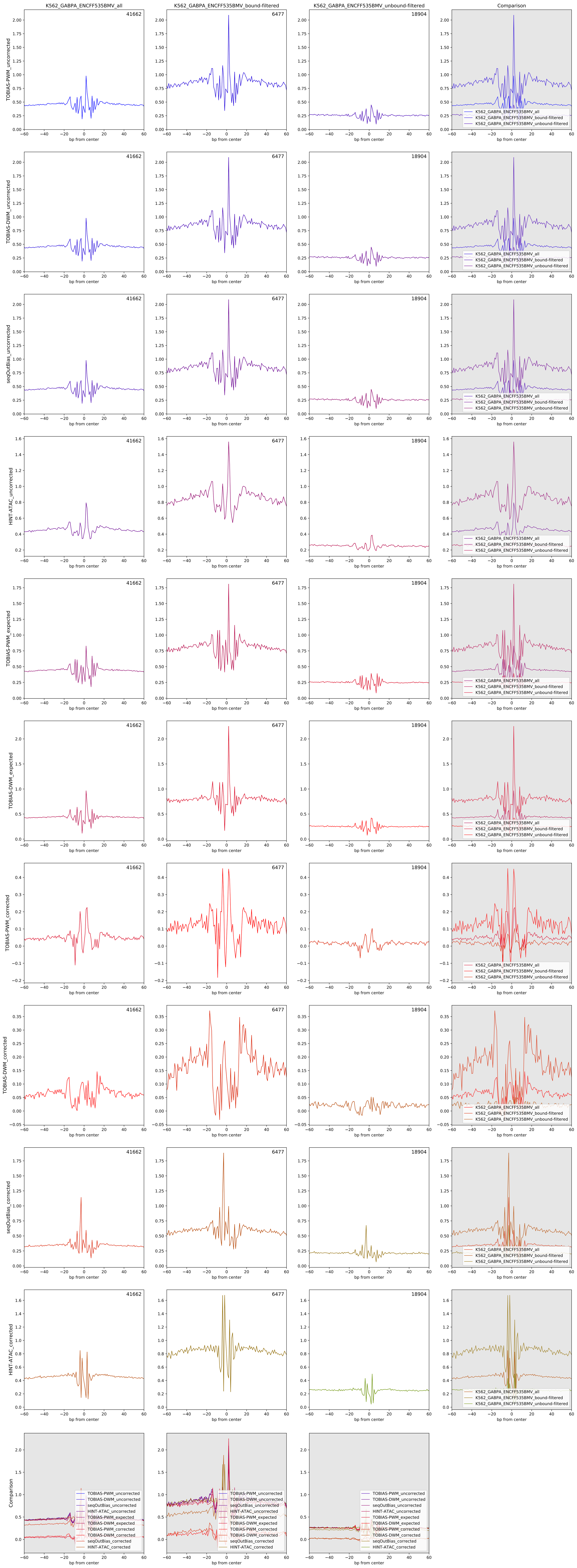

Aggregate footprints in K562 for TF GATA1 (ChIP-seq ENCFF148JKK)

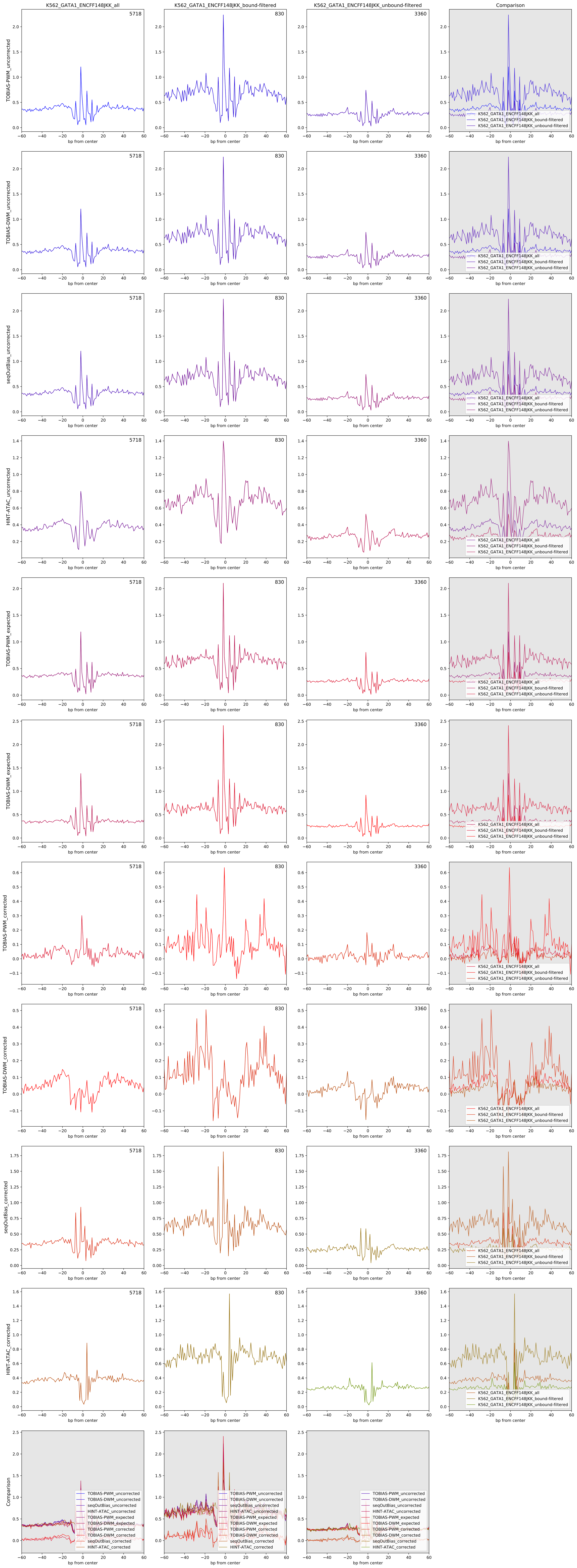

Aggregate footprints in K562 for TF GATA2 (ChIP-seq ENCFF173TXA)

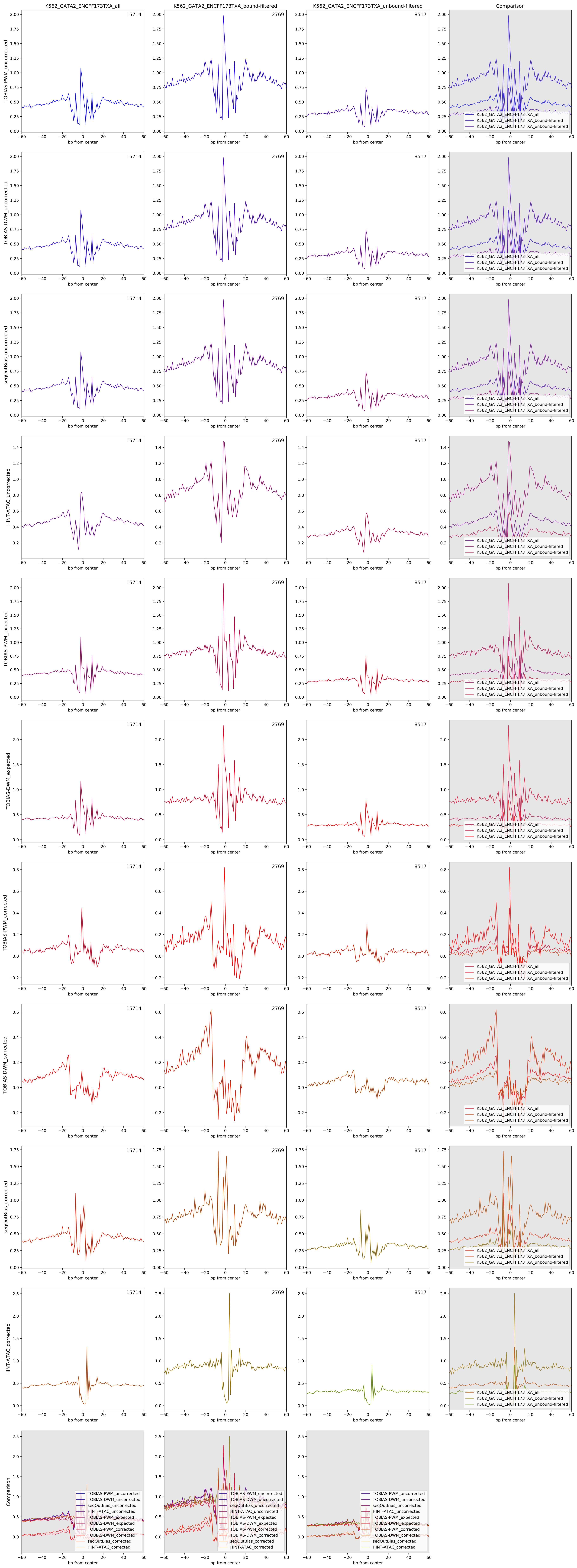

Aggregate footprints in K562 for TF HES1 (ChIP-seq ENCFF010OOE)

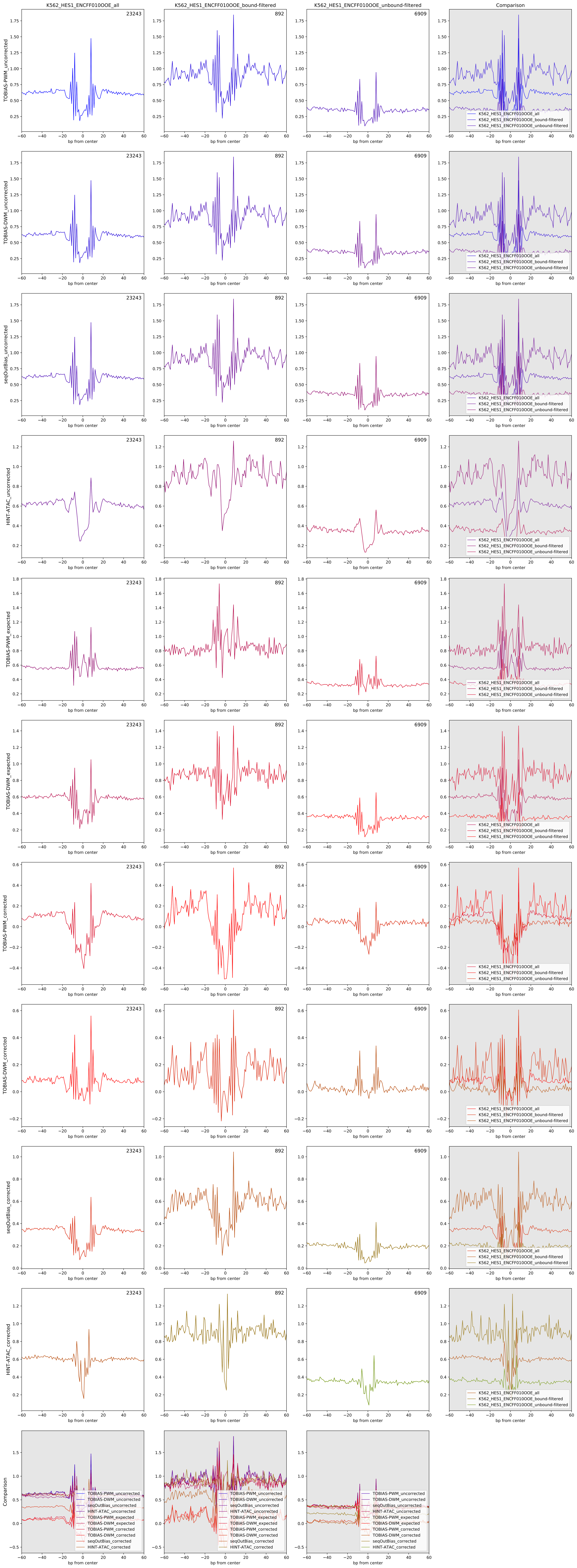

Aggregate footprints in K562 for TF HINFP (ChIP-seq ENCFF558VAL)

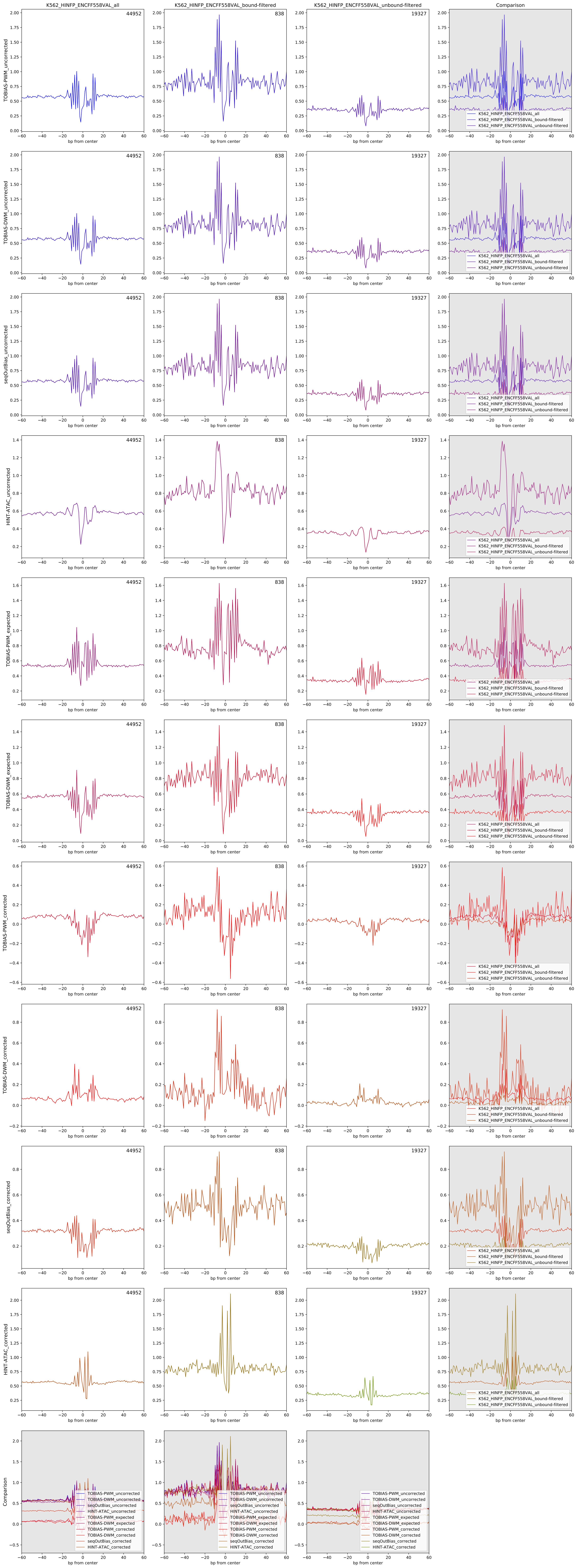

Aggregate footprints in K562 for TF HMBOX1 (ChIP-seq ENCFF718DFX)

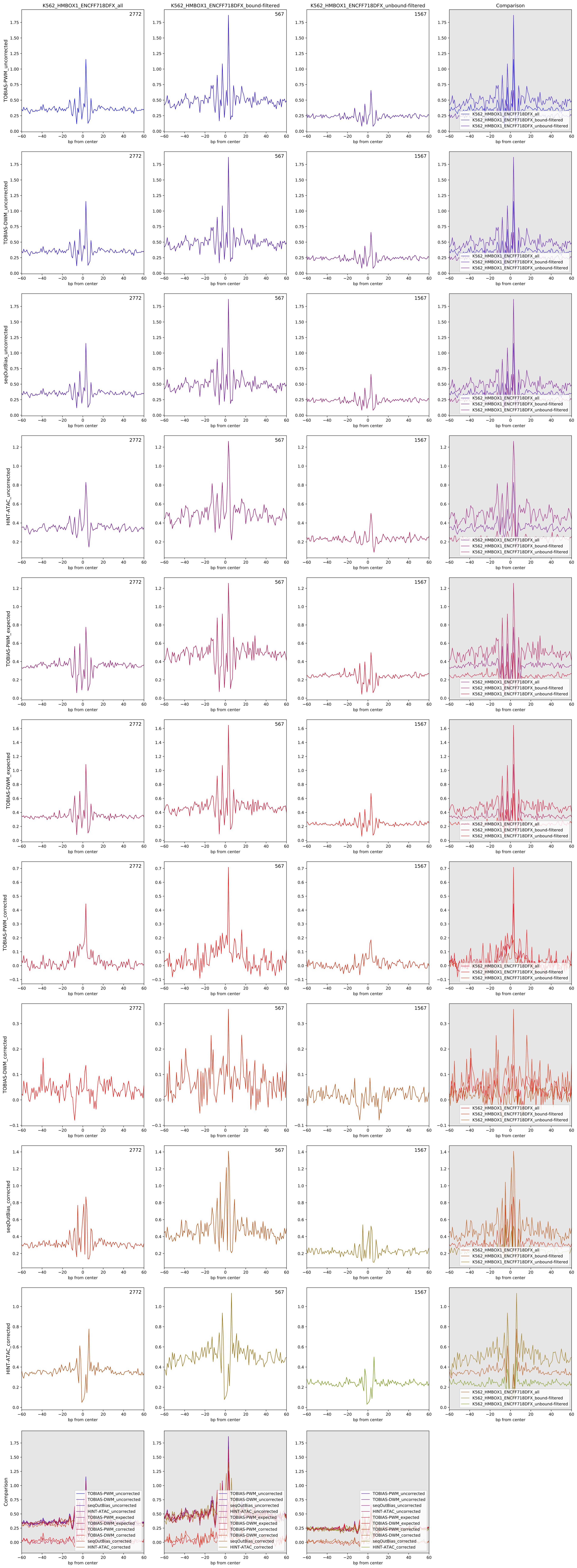

Aggregate footprints in K562 for TF IRF1 (ChIP-seq ENCFF203LRV)

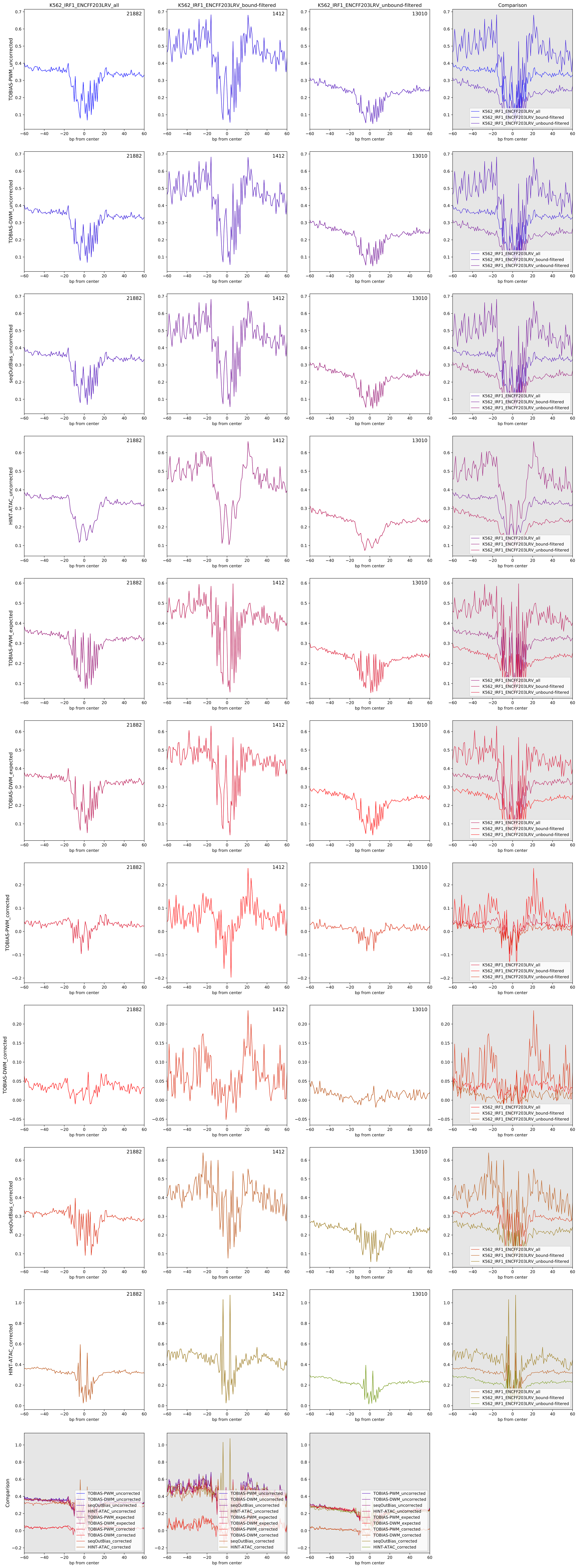

Aggregate footprints in K562 for TF IRF2 (ChIP-seq ENCFF886EVL)

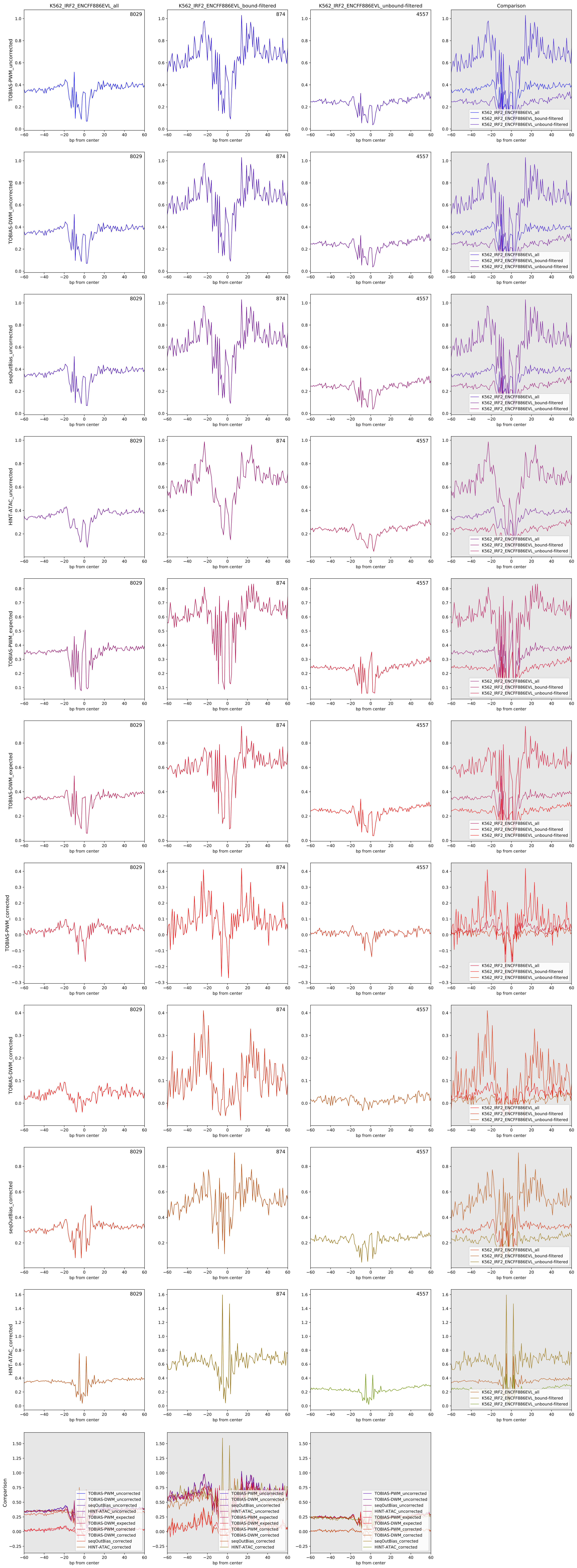

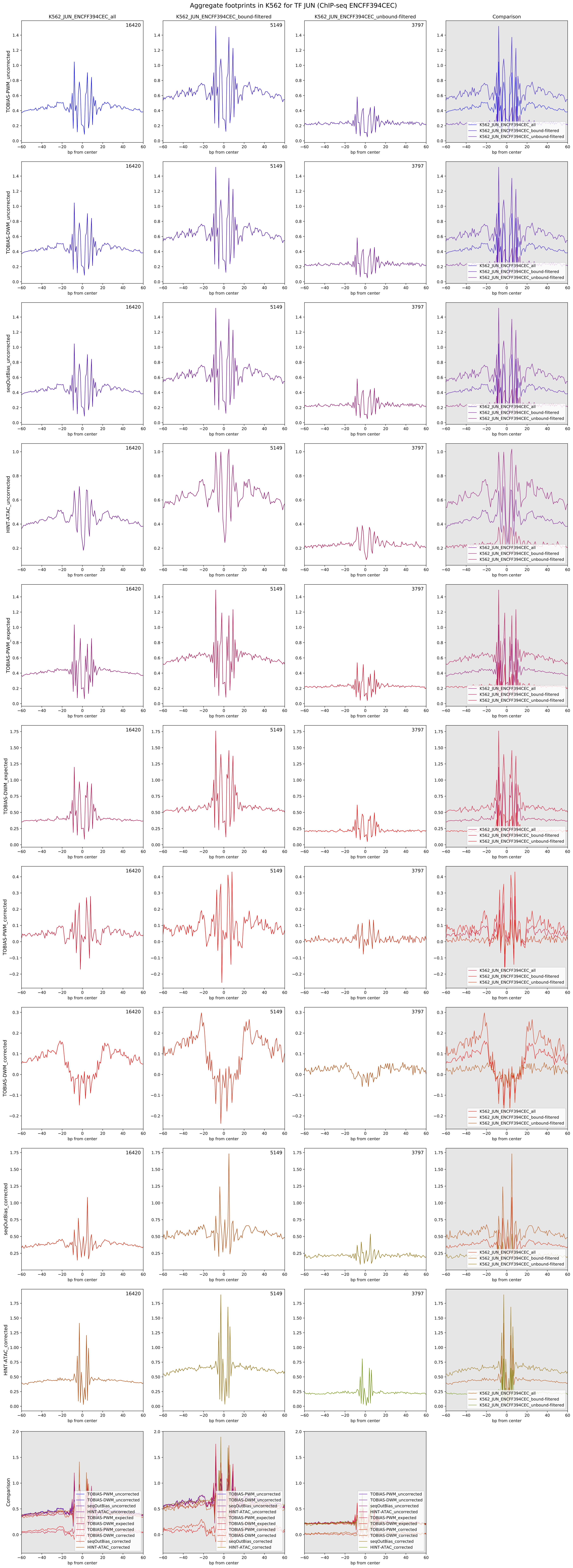
Aggregate footprints in K562 for TF JUN (ChIP-seq ENCFF394CEC)
K562_JUN_ENCFF394CEC_all
K562_JUN_ENCFF394CEC_bound-filtered
K562_JUN_ENCFF394CEC_unbound-filtered
Comparison
16420
5149
3797
TOBIAS-PWM_uncorrected
TOBIAS-DWM_uncorrected
seqOutBias_uncorrected
HINT-ATAC_uncorrected
TOBIAS-PWM_expected
TOBIAS-DWM_expected
TOBIAS-PWM_corrected
TOBIAS-DWM_corrected
seqOutBias_corrected
HINT-ATAC_corrected
bp from center

Aggregate footprints in K562 for TF JUND (ChIP-seq ENCFF213EYD)

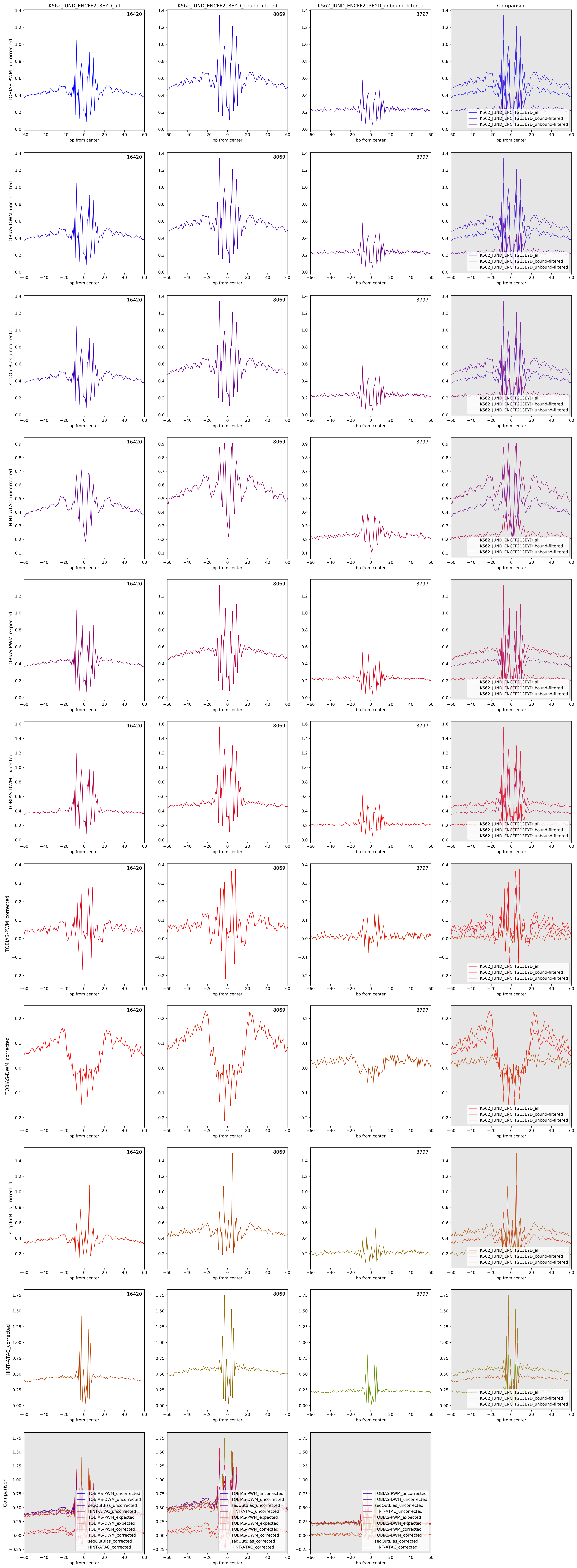

Aggregate footprints in K562 for TF KLF1 (ChIP-seq ENCFF287GDT)

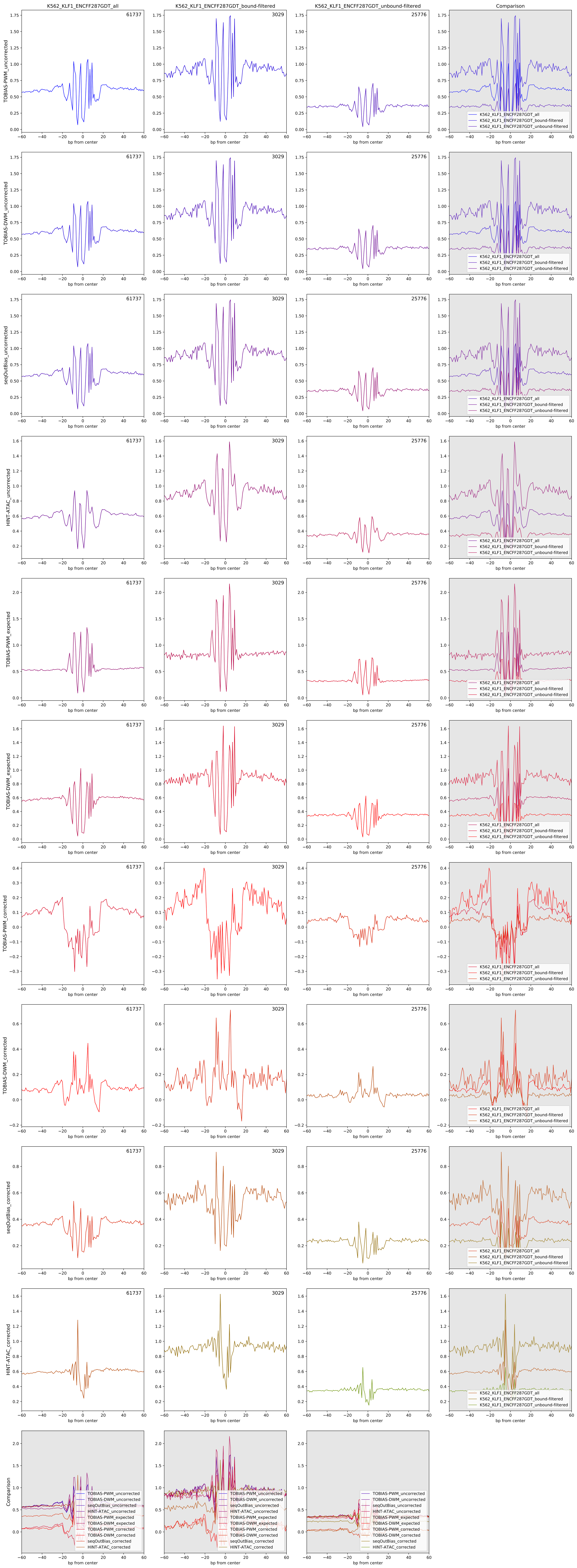

Aggregate footprints in K562 for TF KLF13 (ChIP-seq ENCFF381GEK)

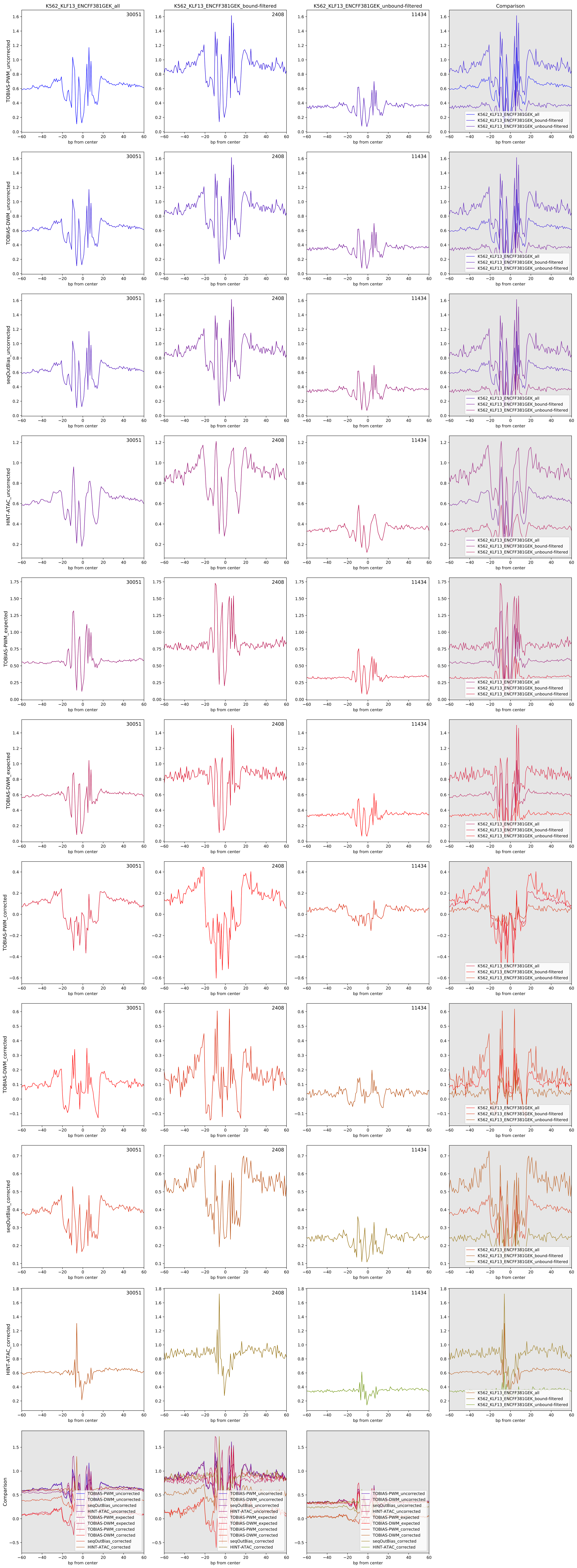

Aggregate footprints in K562 for TF LEF1 (ChIP-seq ENCFF697VRJ)

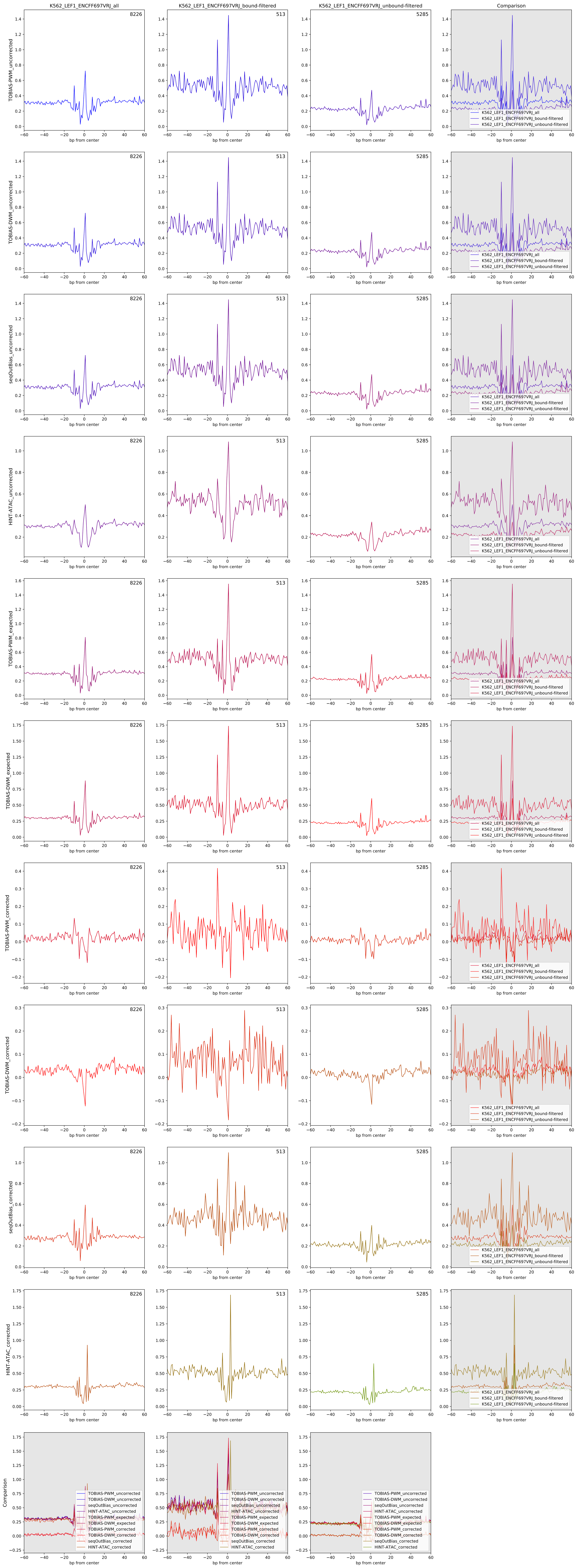

Aggregate footprints in K562 for TF MAFF (ChIP-seq ENCFF498MGH)

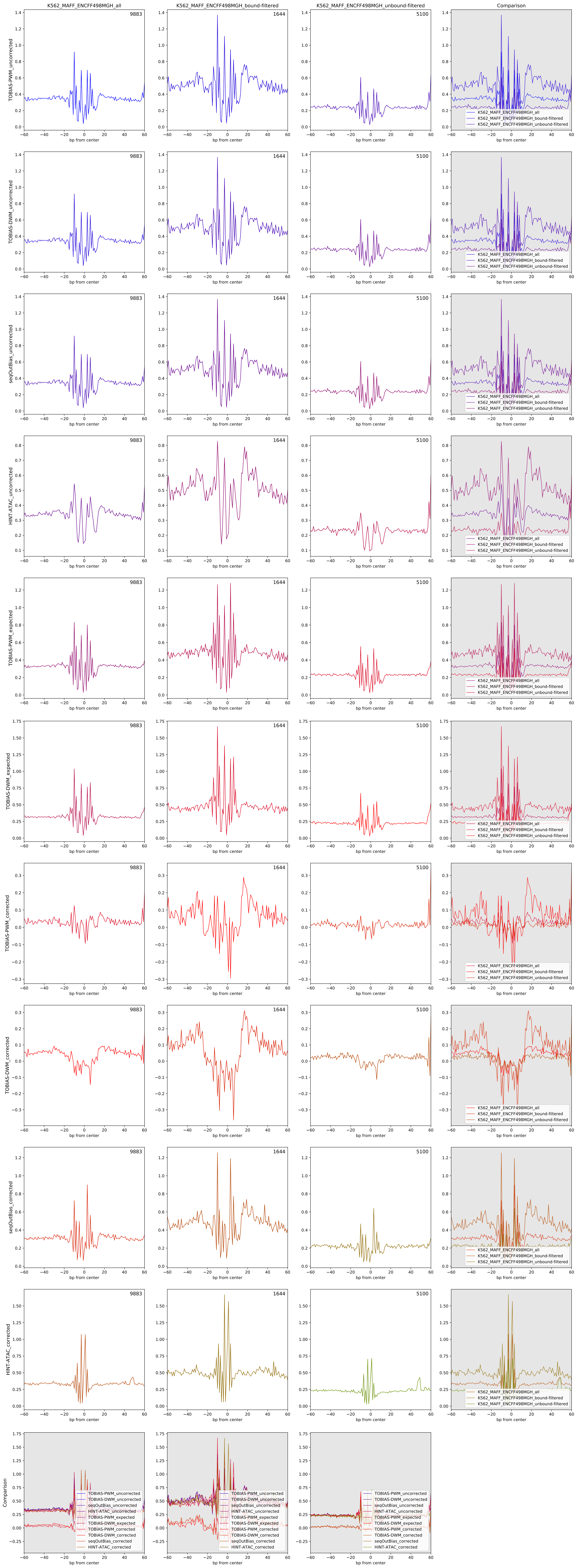

Aggregate footprints in K562 for TF MAFG (ChIP-seq ENCFF393CCU)

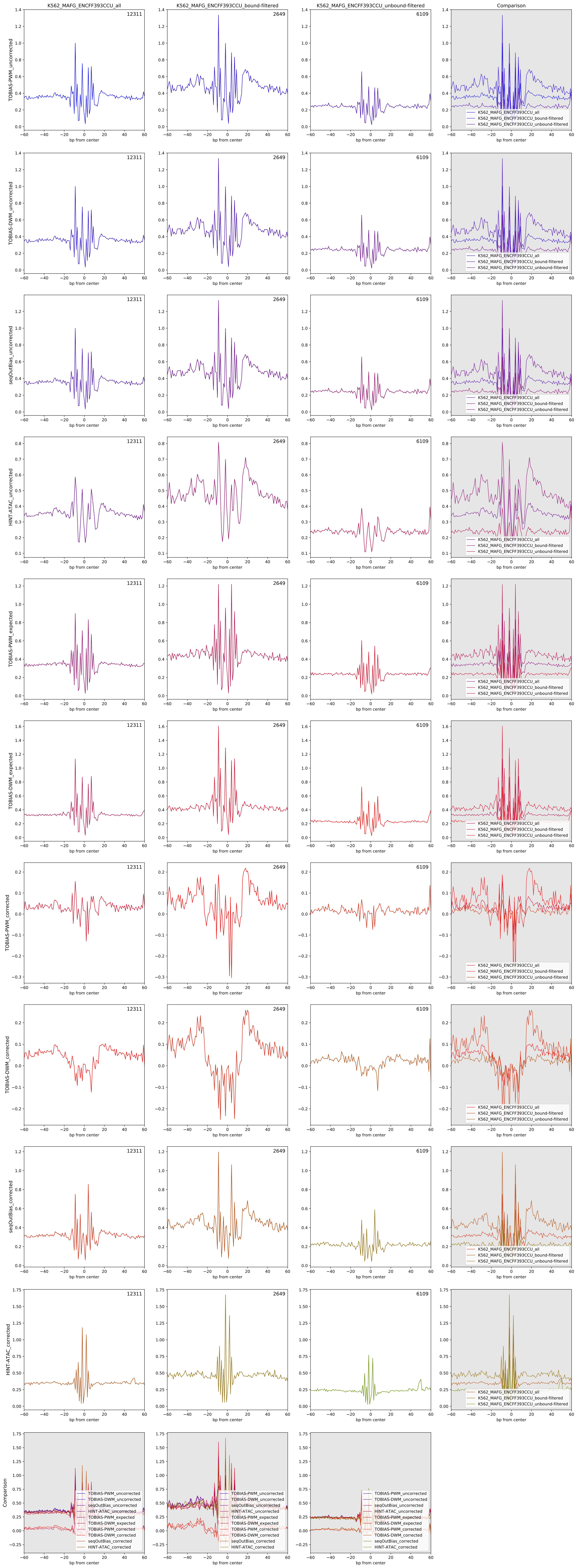

Aggregate footprints in K562 for TF MAFK (ChIP-seq ENCFF893SCL)

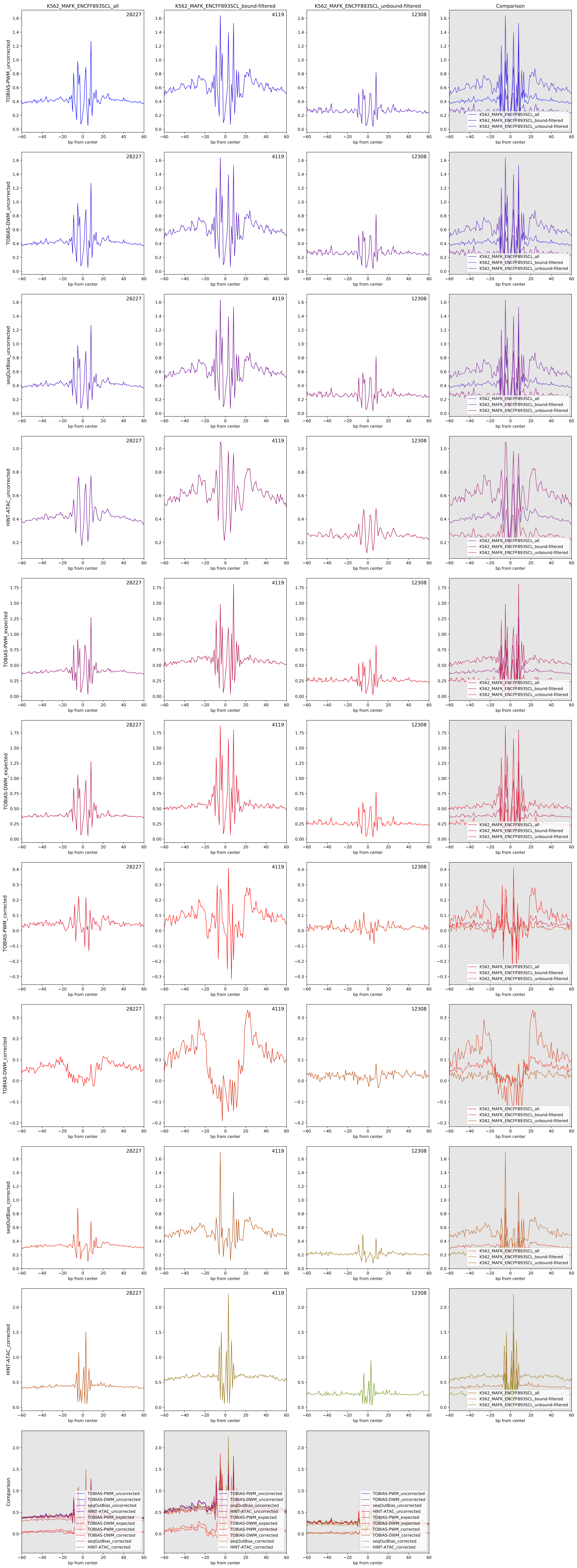

Aggregate footprints in K562 for TF MAX (ChIP-seq ENCFF900NVQ)

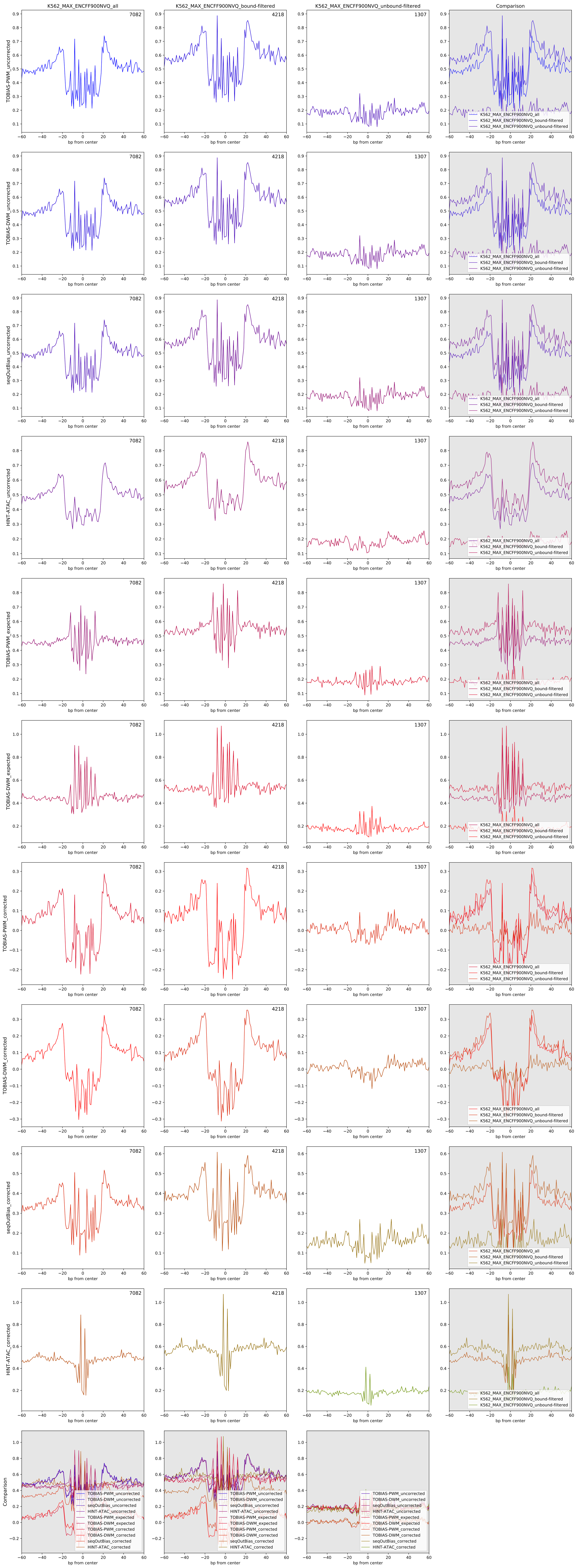

Aggregate footprints in K562 for TF MEF2A (ChIP-seq ENCFF310SMW)

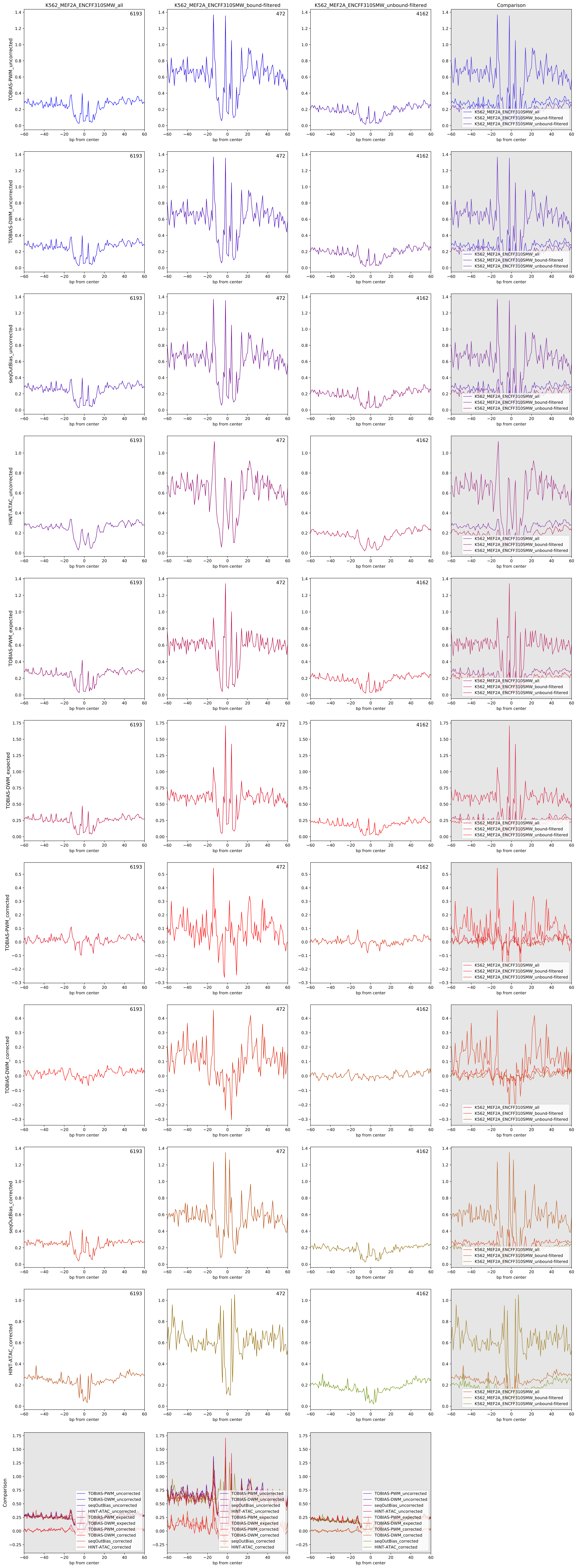

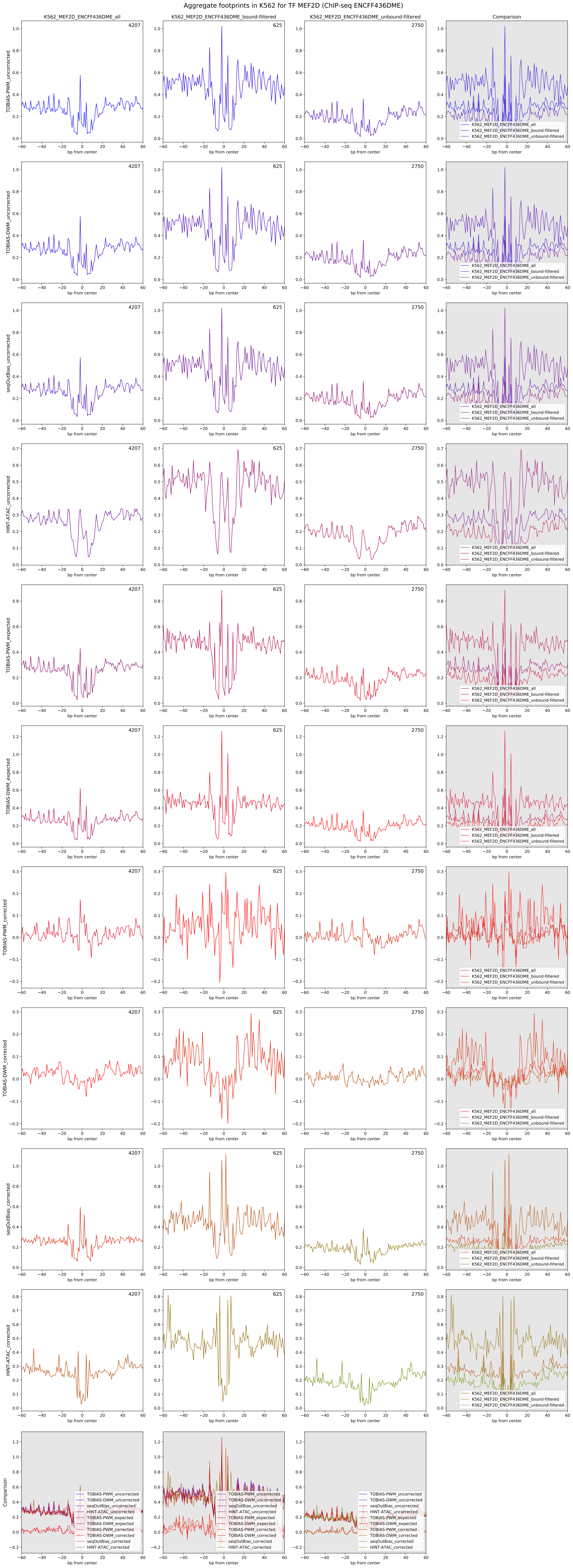
Aggregate footprints in K562 for TF MEF2D (ChIP-seq ENCFF436DME)
K562_MEF2D_ENCFF436DME_all
K562_MEF2D_ENCFF436DME_bound-filtered
K562_MEF2D_ENCFF436DME_unbound-filtered
Comparison
TOBIAS-PWM_uncorrected
TOBIAS-DWM_uncorrected
seqOutBias_uncorrected
HINT-ATAC_uncorrected
TOBIAS-PWM_expected
TOBIAS-DWM_expected
TOBIAS-PWM_corrected
TOBIAS-DWM_corrected
seqOutBias_corrected
HINT-ATAC_corrected
4207
625
2750
bp from center

Aggregate footprints in K562 for TF MEIS2 (ChIP-seq ENCFF937UEE)

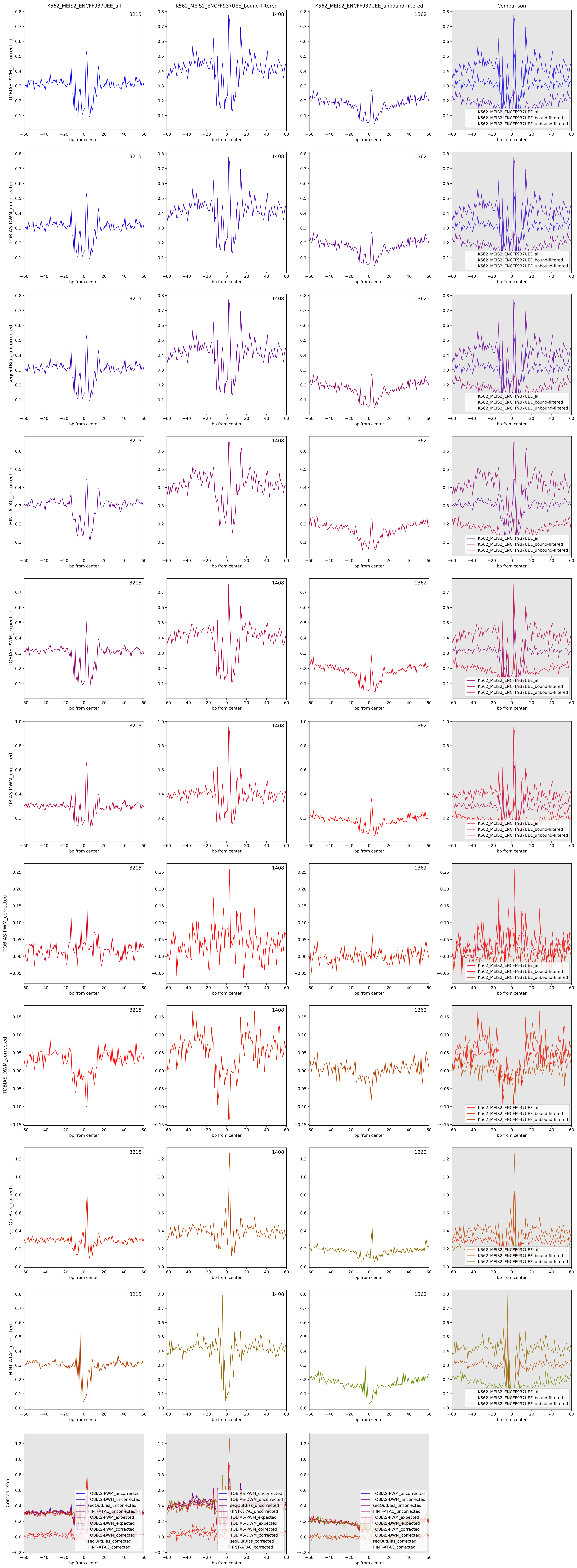

Aggregate footprints in K562 for TF MGA (ChIP-seq ENCFF525MPI)

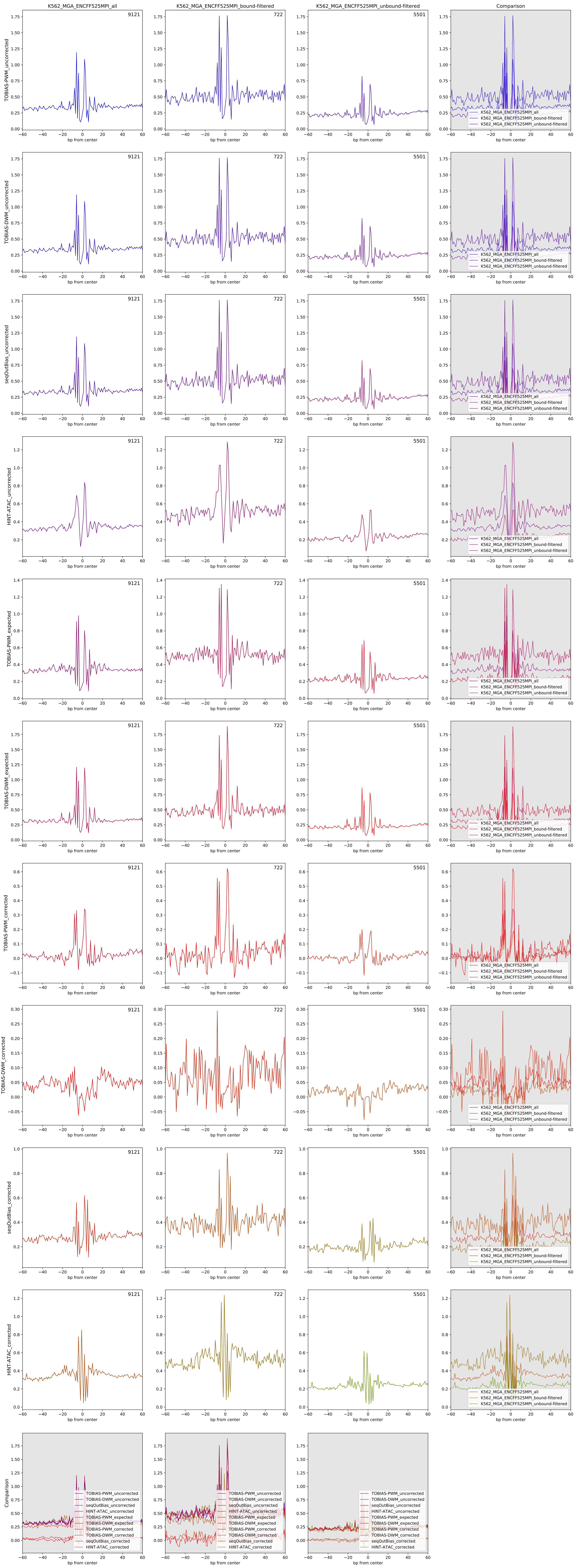

Aggregate footprints in K562 for TF MITF (ChIP-seq ENCFF262TMM)

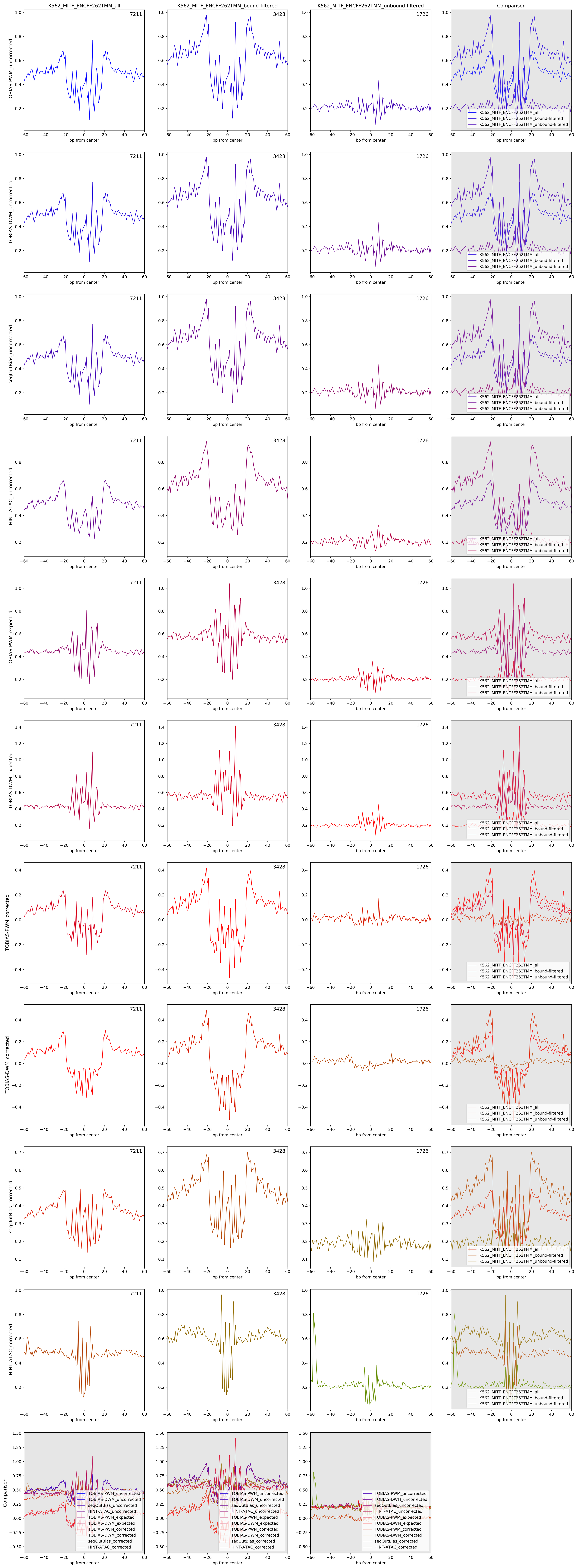

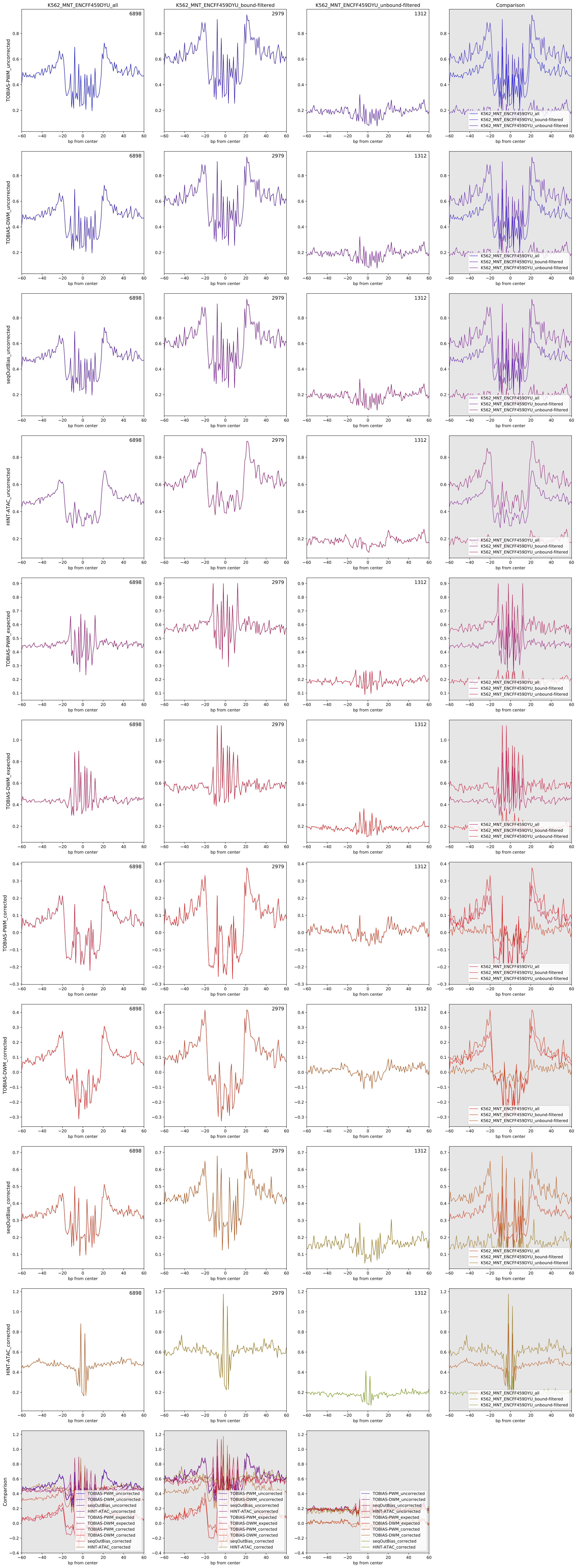
Aggregate footprints in K562 for TF MNT (ChIP-seq ENCFF459DYU)
K562_MNT_ENCFF459DYU_all
K562_MNT_ENCFF459DYU_bound-filtered
K562_MNT_ENCFF459DYU_unbound-filtered
Comparison
TOBIAS-PWM_uncorrected
TOBIAS-DWM_uncorrected
seqOutBias_uncorrected
HINT-ATAC_uncorrected
TOBIAS-PWM_expected
TOBIAS-DWM_expected
TOBIAS-PWM_corrected
TOBIAS-DWM_corrected
seqOutBias_corrected
HINT-ATAC_corrected
6898
2979
1312
bp from center

Aggregate footprints in K562 for TF MXI1 (ChIP-seq ENCFF243QTL)

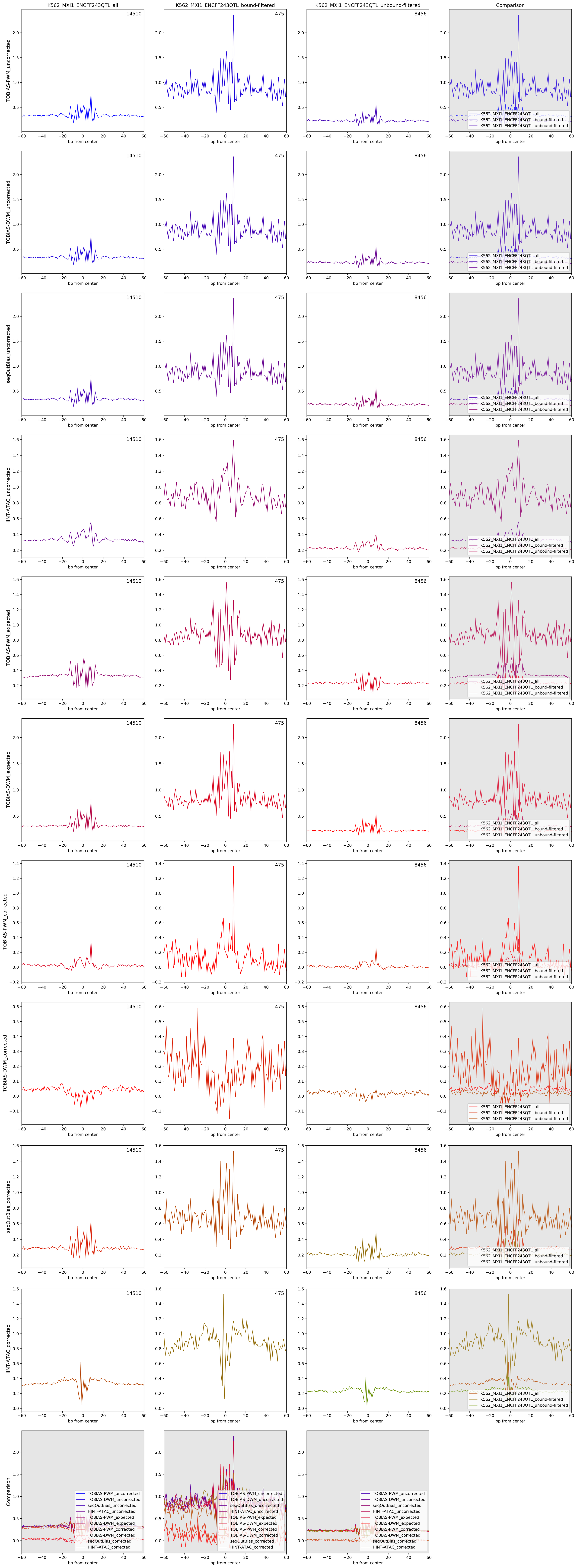

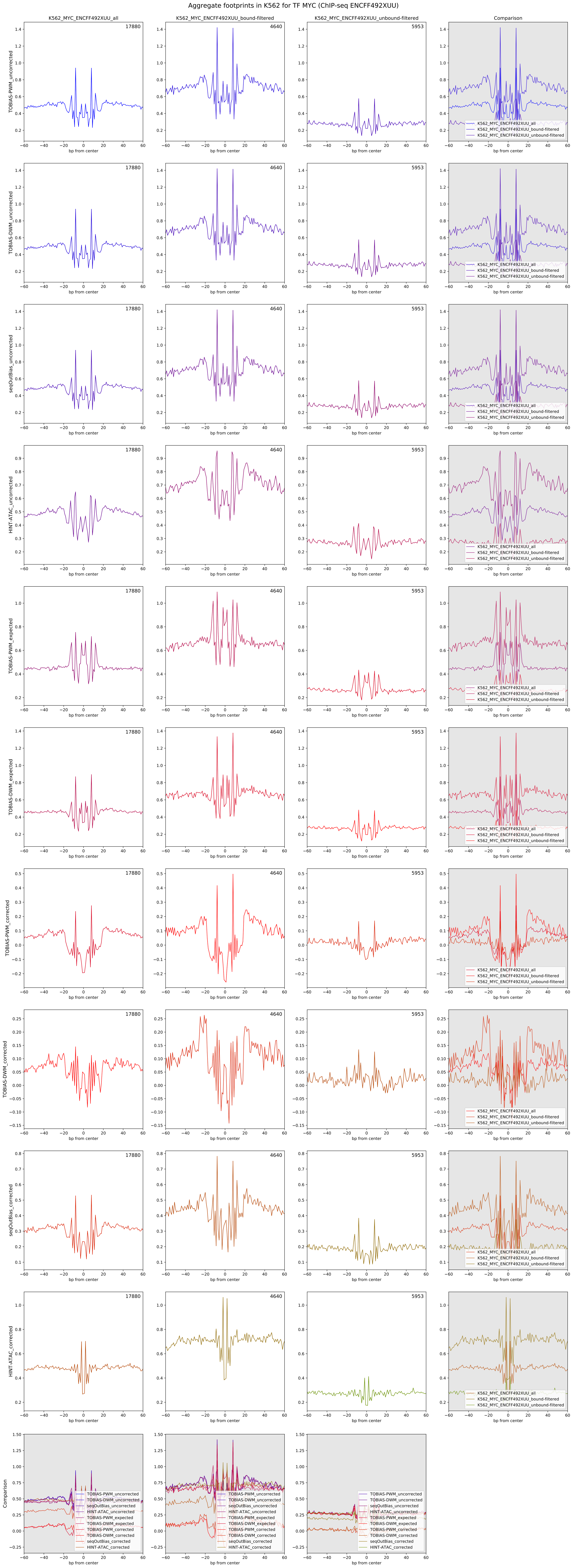
Aggregate footprints in K562 for TF MYC (ChIP-seq ENCFF492XUU)
K562_MYC_ENCFF492XUU_all
K562_MYC_ENCFF492XUU_bound-filtered
K562_MYC_ENCFF492XUU_unbound-filtered
Comparison
TOBIAS-PWM_uncorrected
TOBIAS-DWM_uncorrected
seqOutBias_uncorrected
HINT-ATAC_uncorrected
TOBIAS-PWM_expected
TOBIAS-DWM_expected
TOBIAS-PWM_corrected
TOBIAS-DWM_corrected
seqOutBias_corrected
HINT-ATAC_corrected
Comparison
17880
4640
5953
bp from center
K562_MYC_ENCFF492XUU_all
K562_MYC_ENCFF492XUU_bound-filtered
K562_MYC_ENCFF492XUU_unbound-filtered
TOBIAS-PWM_uncorrected
TOBIAS-DWM_uncorrected
seqOutBias_uncorrected
HINT-ATAC_uncorrected
TOBIAS-PWM_expected
TOBIAS-DWM_expected
TOBIAS-PWM_corrected
TOBIAS-DWM_corrected
seqOutBias_corrected
HINT-ATAC_corrected

Aggregate footprints in K562 for TF NFE2 (ChIP-seq ENCFF286HTI)

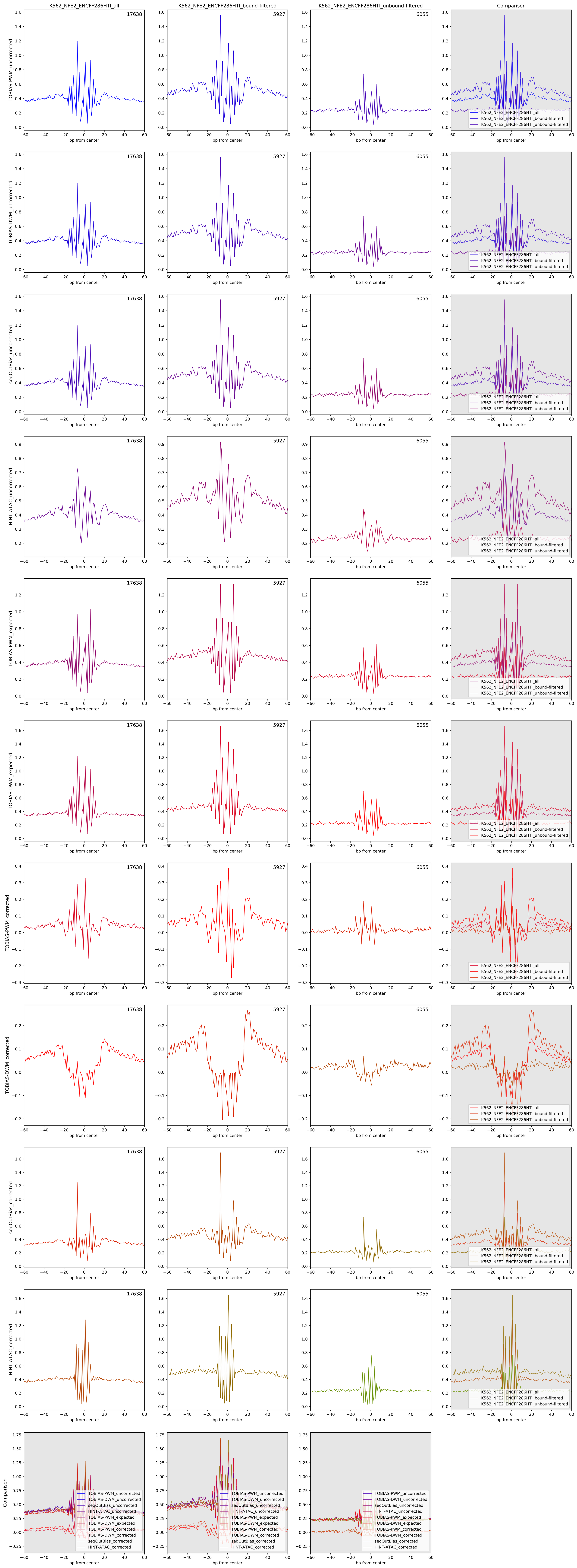

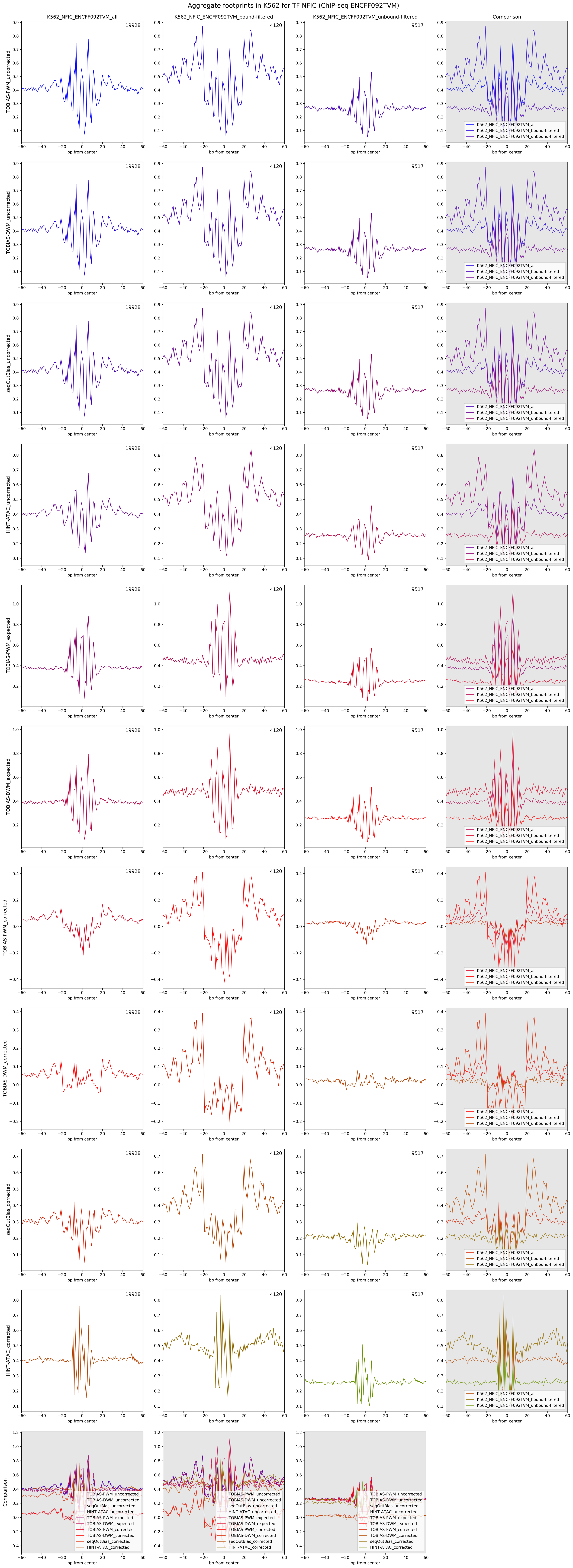

Aggregate footprints in K562 for TF NFIC (ChIP-seq ENCFF092TVM)
K562_NFIC_ENCFF092TVM_all
K562_NFIC_ENCFF092TVM_bound-filtered
K562_NFIC_ENCFF092TVM_unbound-filtered
Comparison
TOBIAS-PWM_uncorrected
TOBIAS-DWM_uncorrected
seqOutBias_uncorrected
HINT-ATAC_uncorrected
TOBIAS-PWM_expected
TOBIAS-DWM_expected
TOBIAS-PWM_corrected
TOBIAS-DWM_corrected
seqOutBias_corrected
HINT-ATAC_corrected
19928
4120
9517
bp from center

Aggregate footprints in K562 for TF NFYA (ChIP-seq ENCFF302CPE)

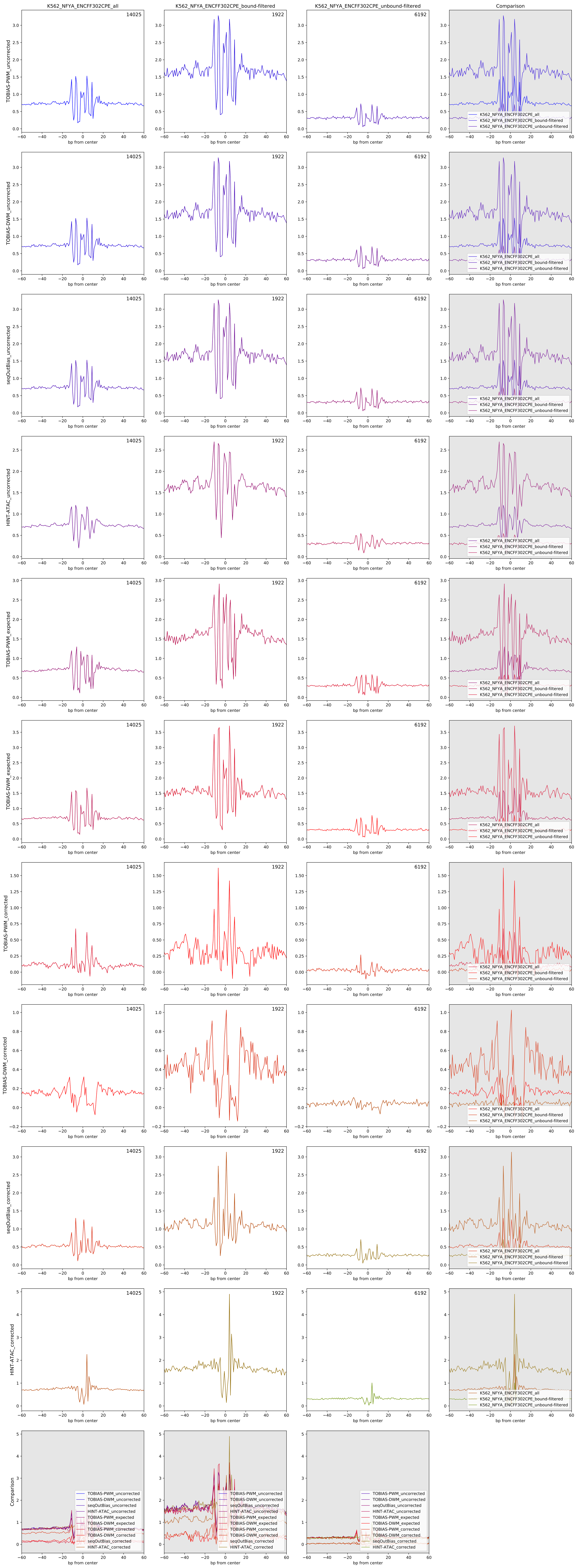

Aggregate footprints in K562 for TF NFYB (ChIP-seq ENCFF009NXK)

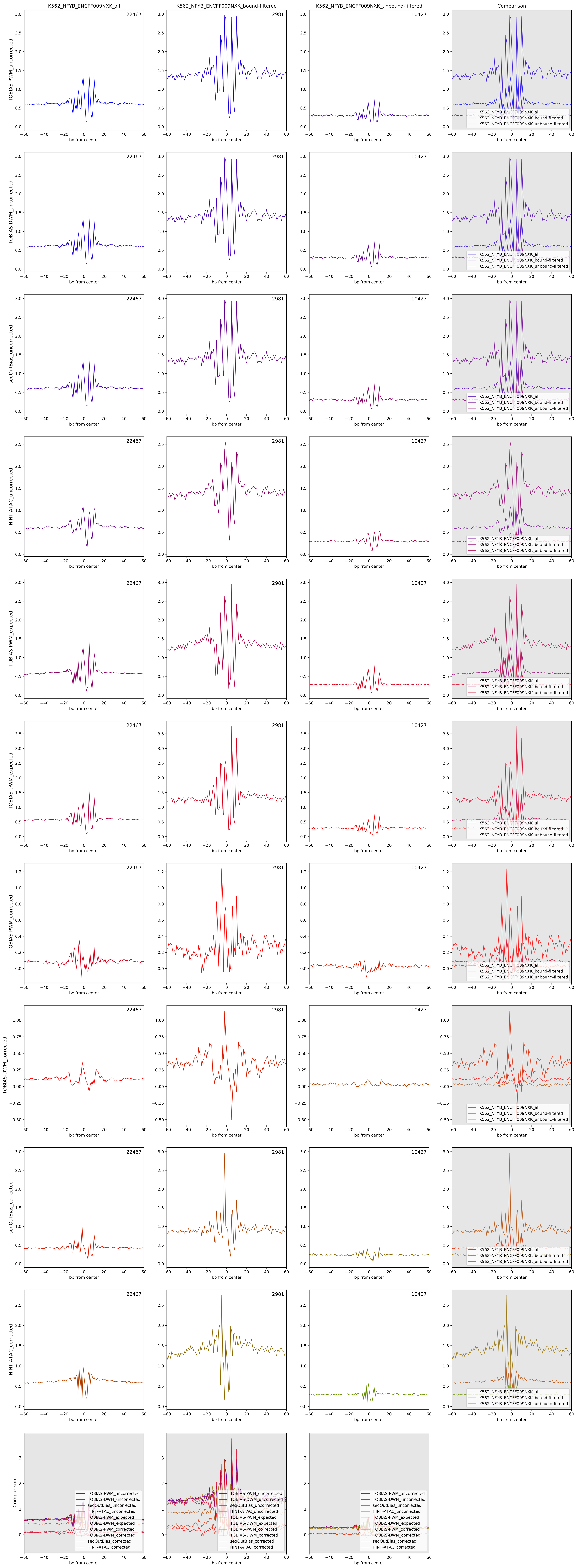

Aggregate footprints in K562 for TF NR2C2 (ChIP-seq ENCFF789AXP)

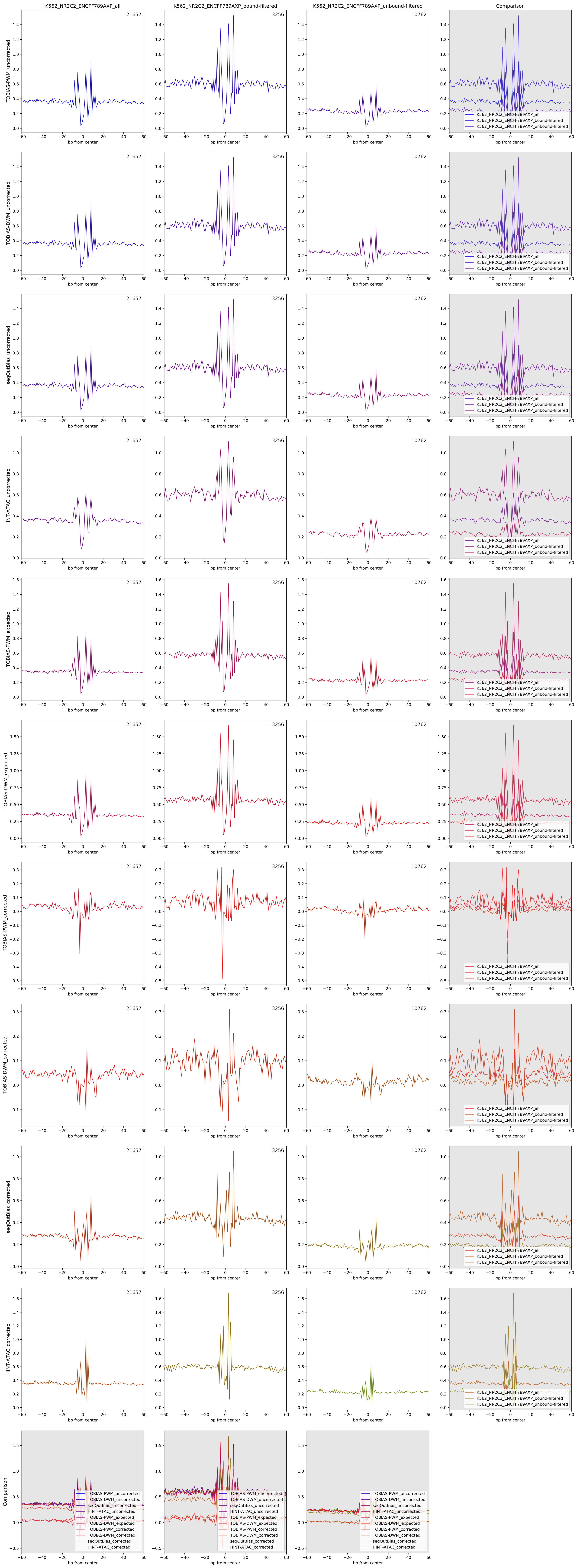

Aggregate footprints in K562 for TF NR2F1 (ChIP-seq ENCFF363IQN)

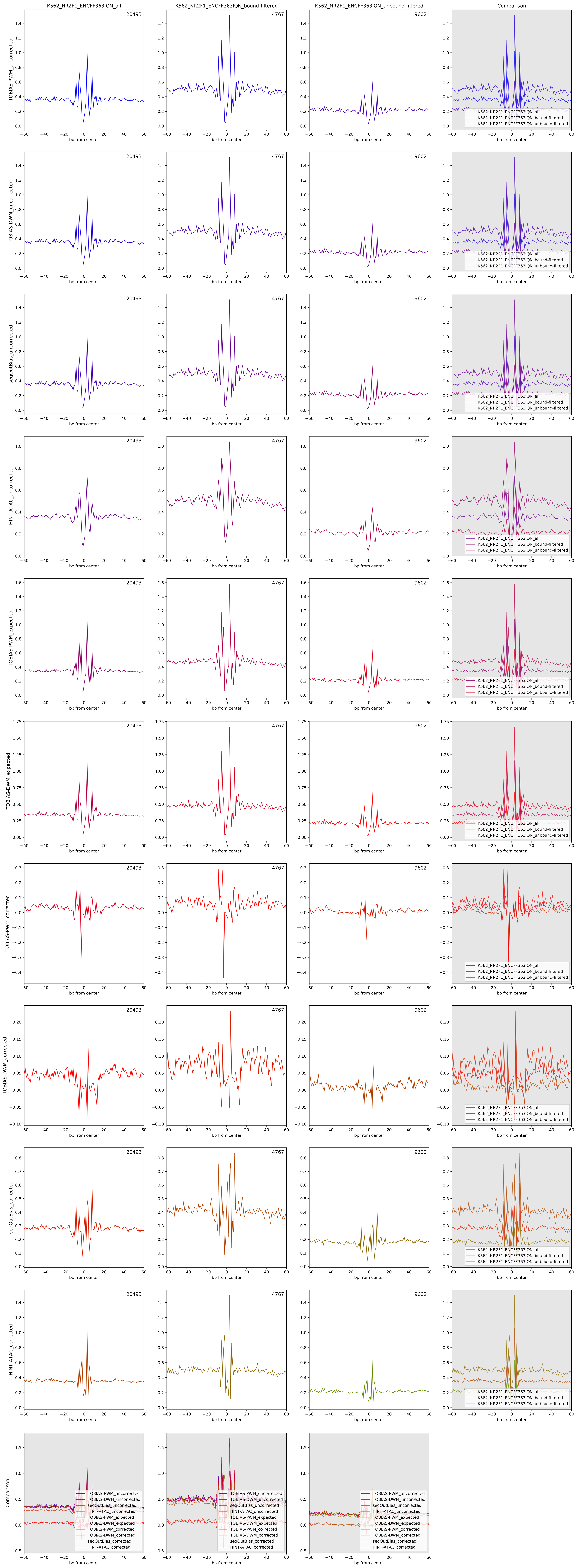

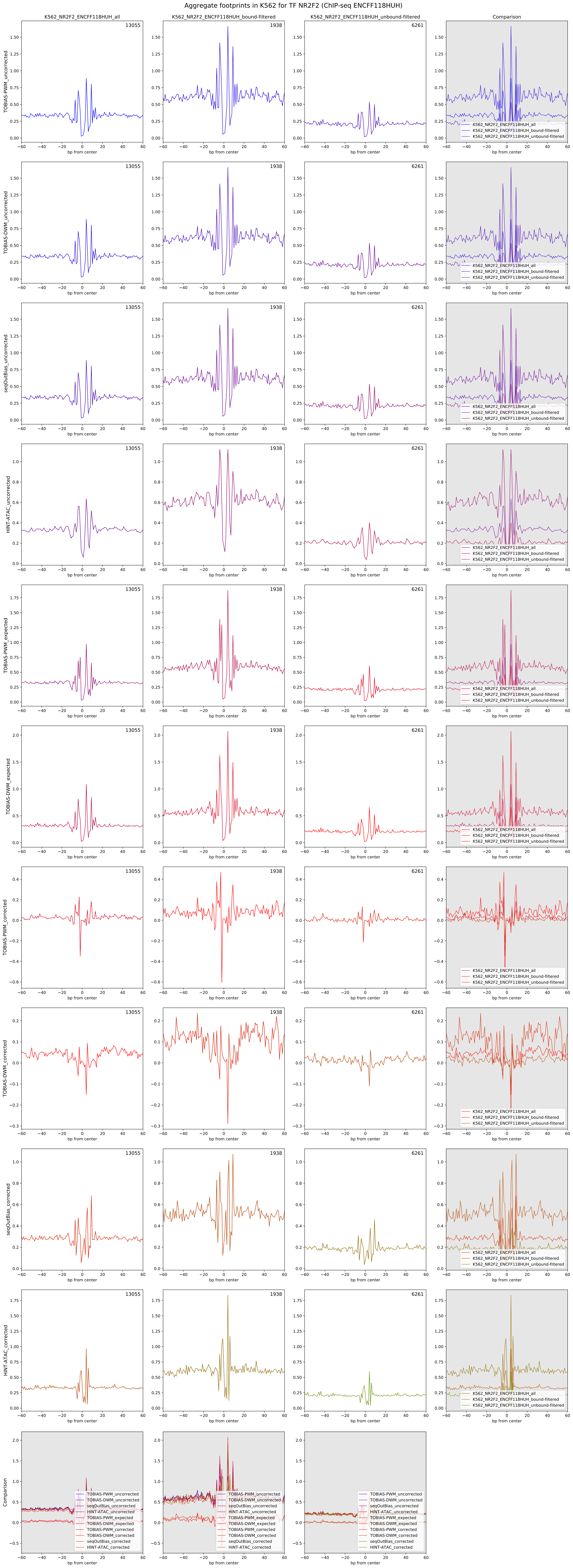

Aggregate footprints in K562 for TF NR2F2 (ChIP-seq ENCFF118HUH)
K562_NR2F2_ENCFF118HUH_all
K562_NR2F2_ENCFF118HUH_bound-filtered
K562_NR2F2_ENCFF118HUH_unbound-filtered
Comparison
TOBIAS-PWM_uncorrected
TOBIAS-DWM_uncorrected
seqOutBias_uncorrected
HINT-ATAC_uncorrected
TOBIAS-PWM_expected
TOBIAS-DWM_expected
TOBIAS-PWM_corrected
TOBIAS-DWM_corrected
seqOutBias_corrected
HINT-ATAC_corrected
13055
1938
6261
bp from center

Aggregate footprints in K562 for TF NR2F6 (ChIP-seq ENCFF194VBK)

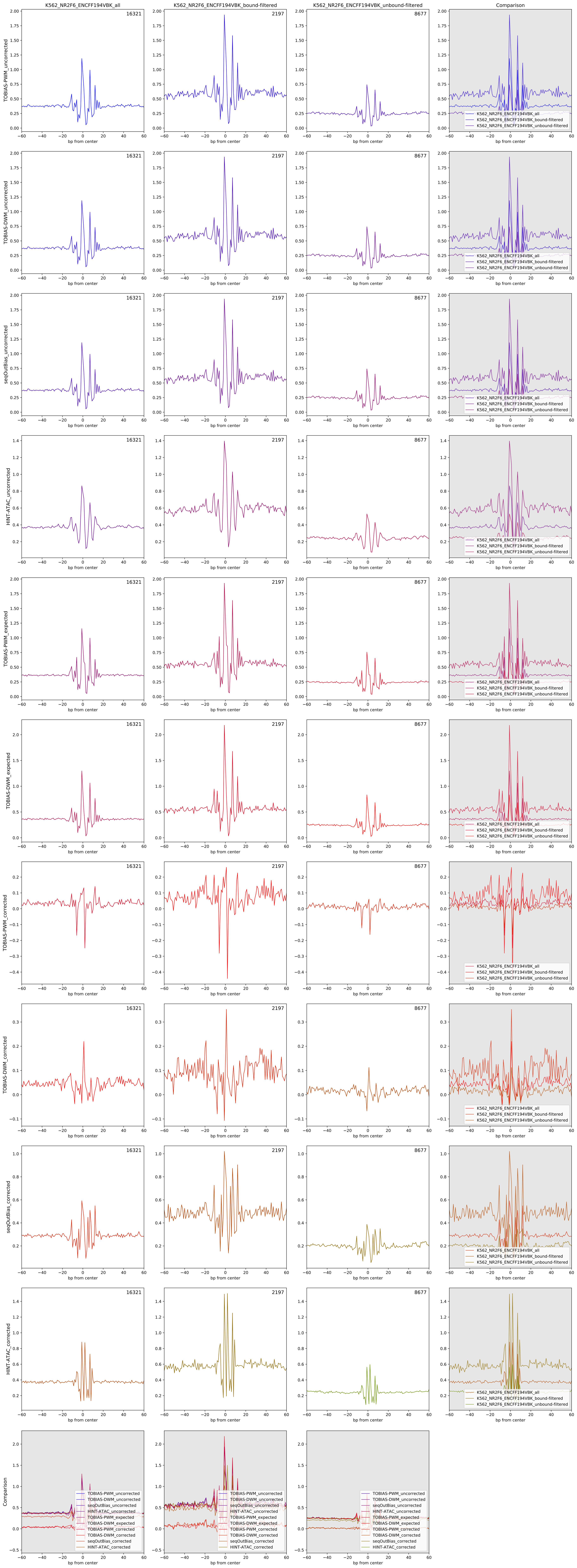

Aggregate footprints in K562 for TF NRF1 (ChIP-seq ENCFF626VDA)

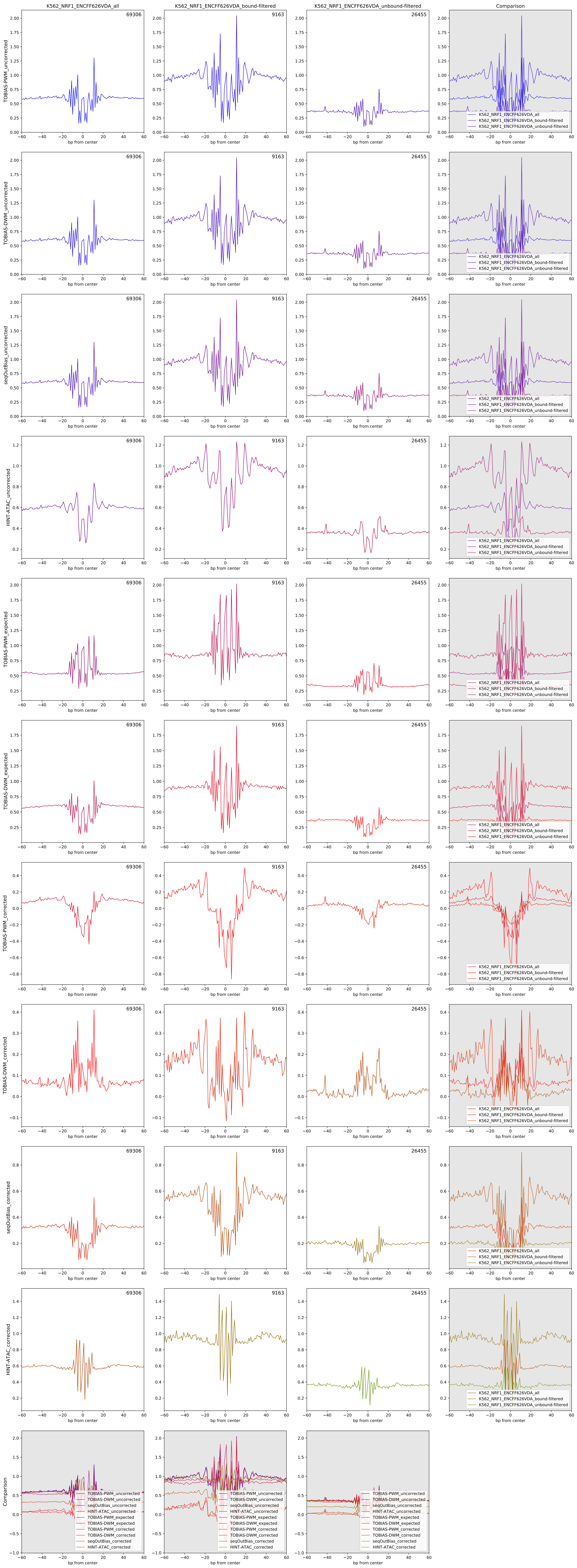

Aggregate footprints in K562 for TF PBX2 (ChIP-seq ENCFF685CMQ)

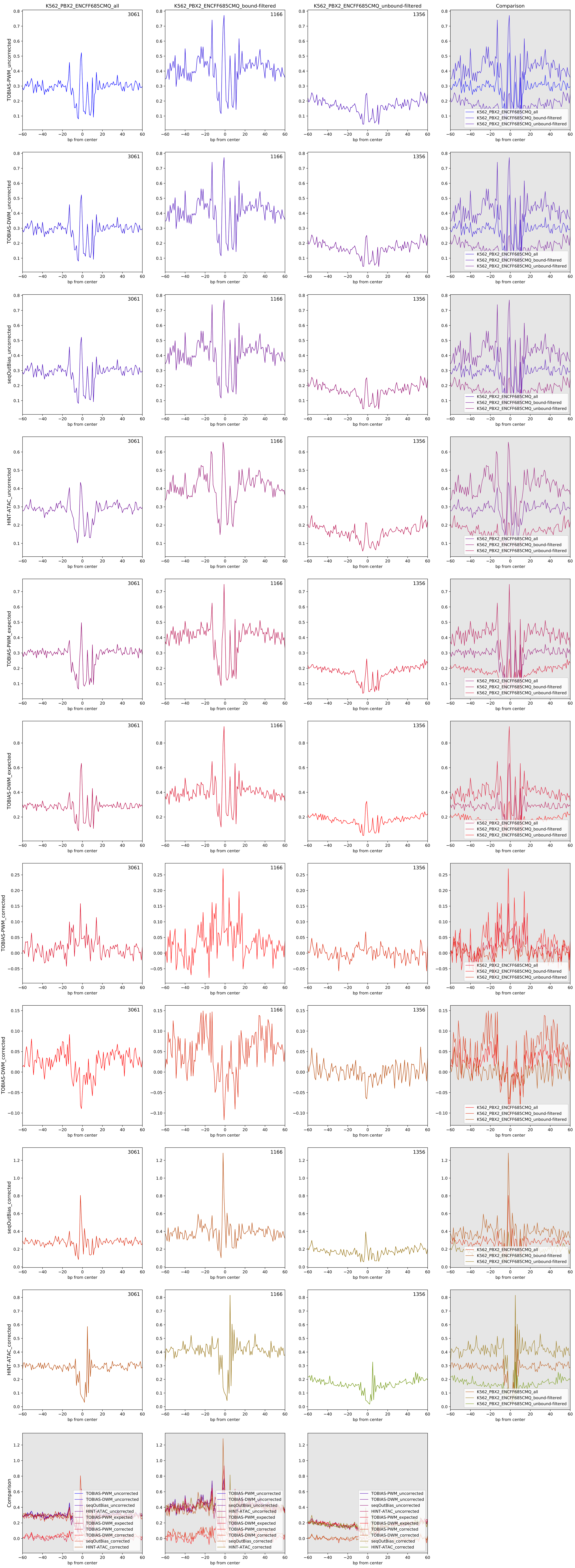

Aggregate footprints in K562 for TF PKNOX1 (ChIP-seq ENCFF062VBB)

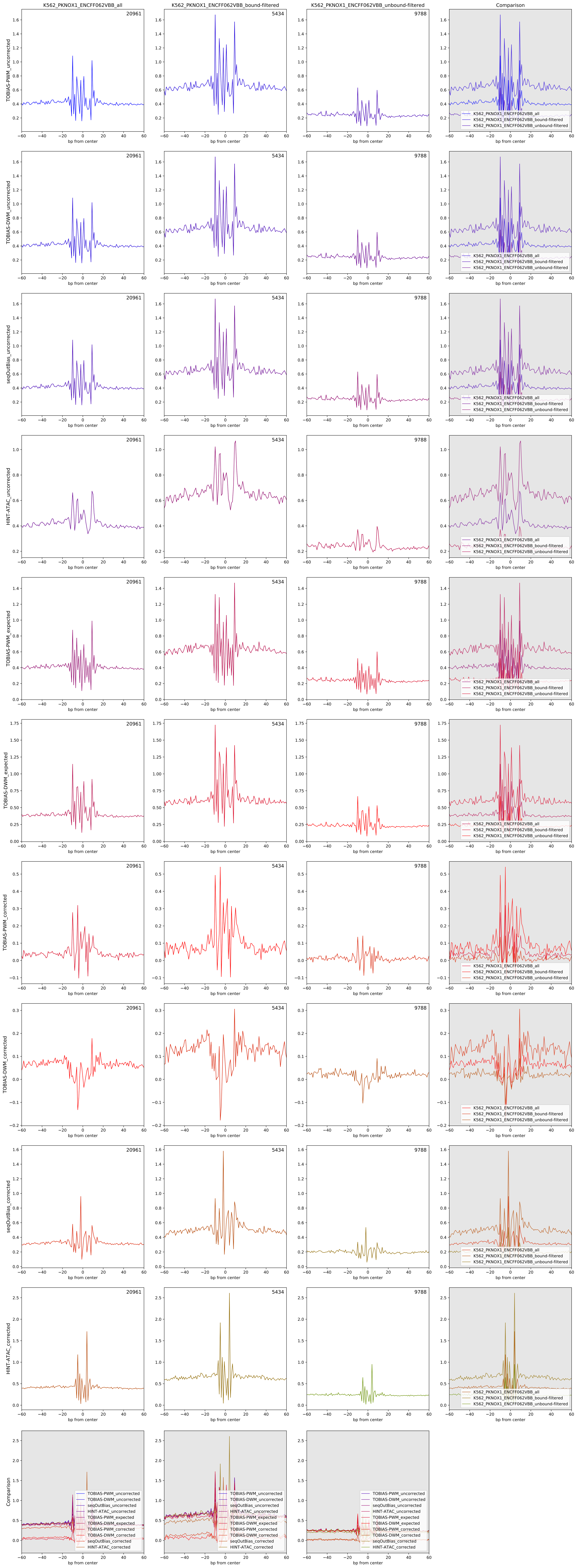

Aggregate footprints in K562 for TF REST (ChIP-seq ENCFF023ZUW)

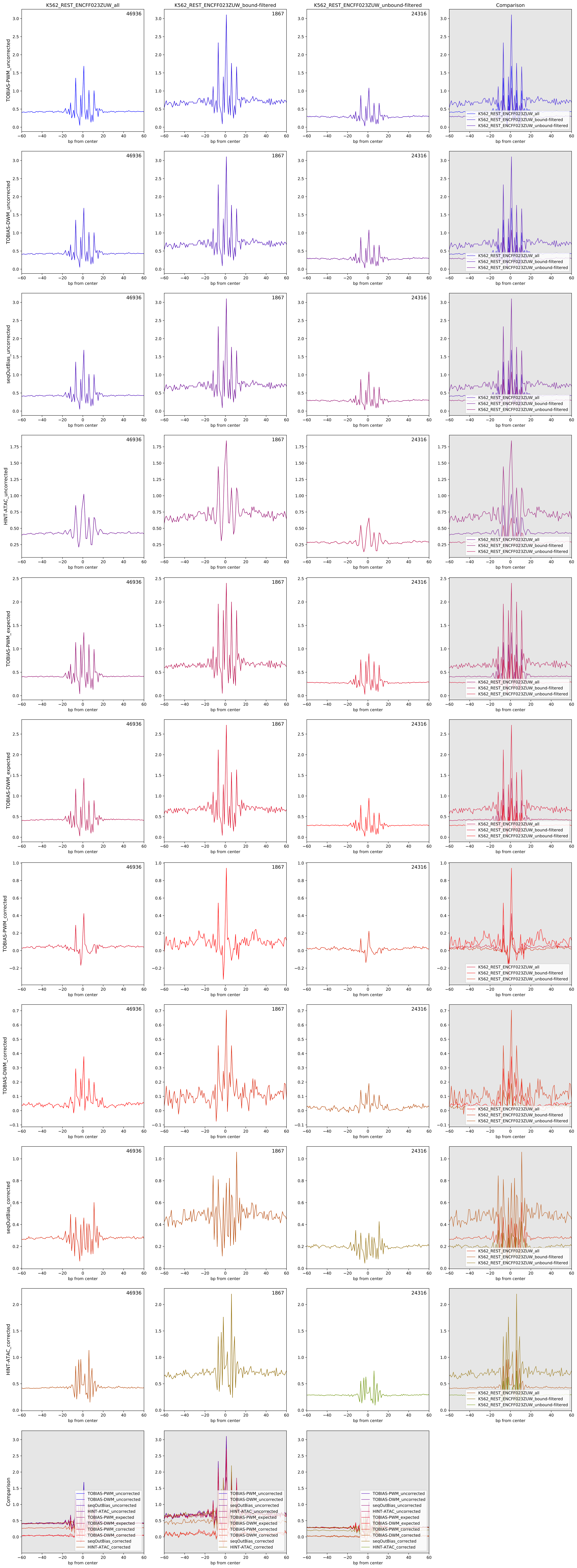

Aggregate footprints in K562 for TF RFX1 (ChIP-seq ENCFF193PVX)

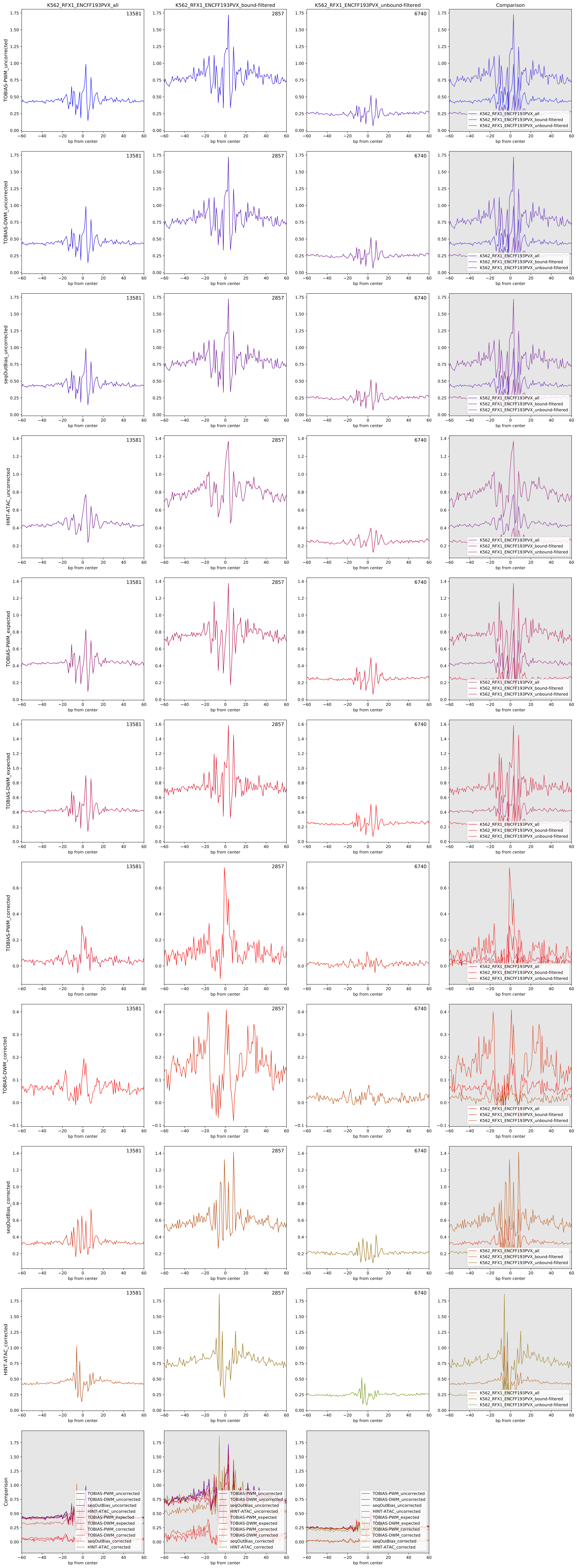

Aggregate footprints in K562 for TF RFX5 (ChIP-seq ENCFF201YKU)

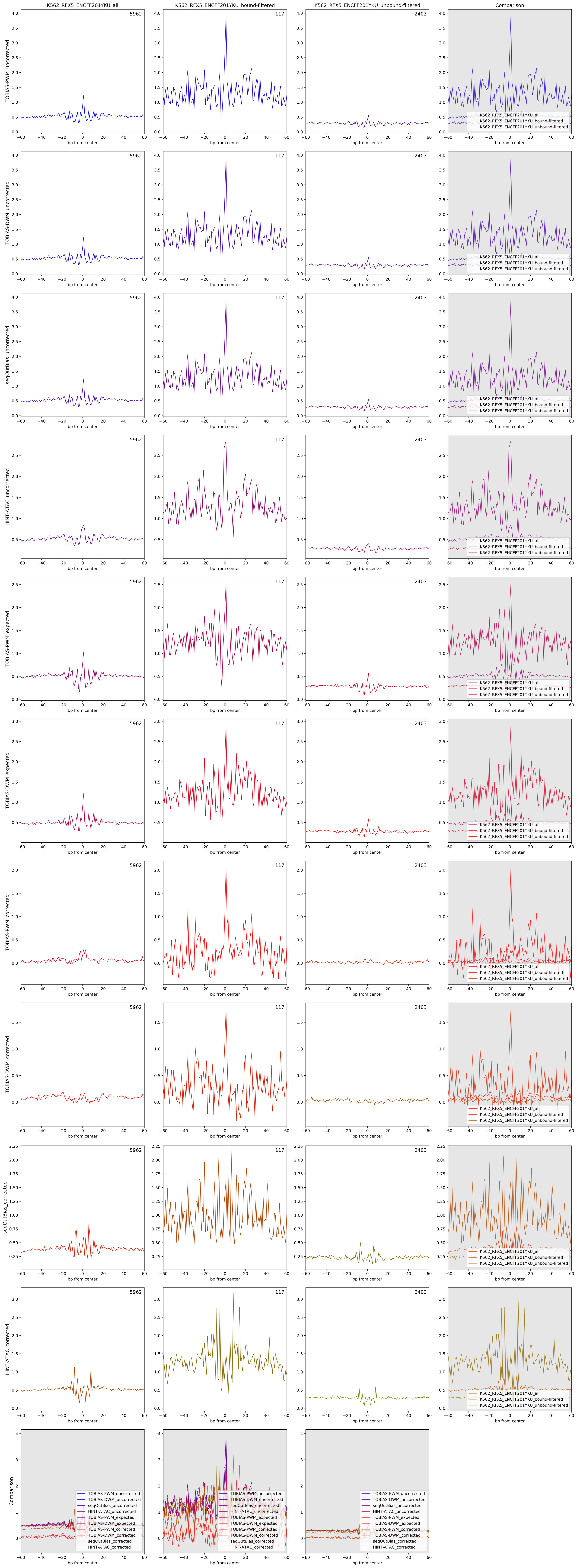

Aggregate footprints in K562 for TF RUNX1 (ChIP-seq ENCFF545WXN)

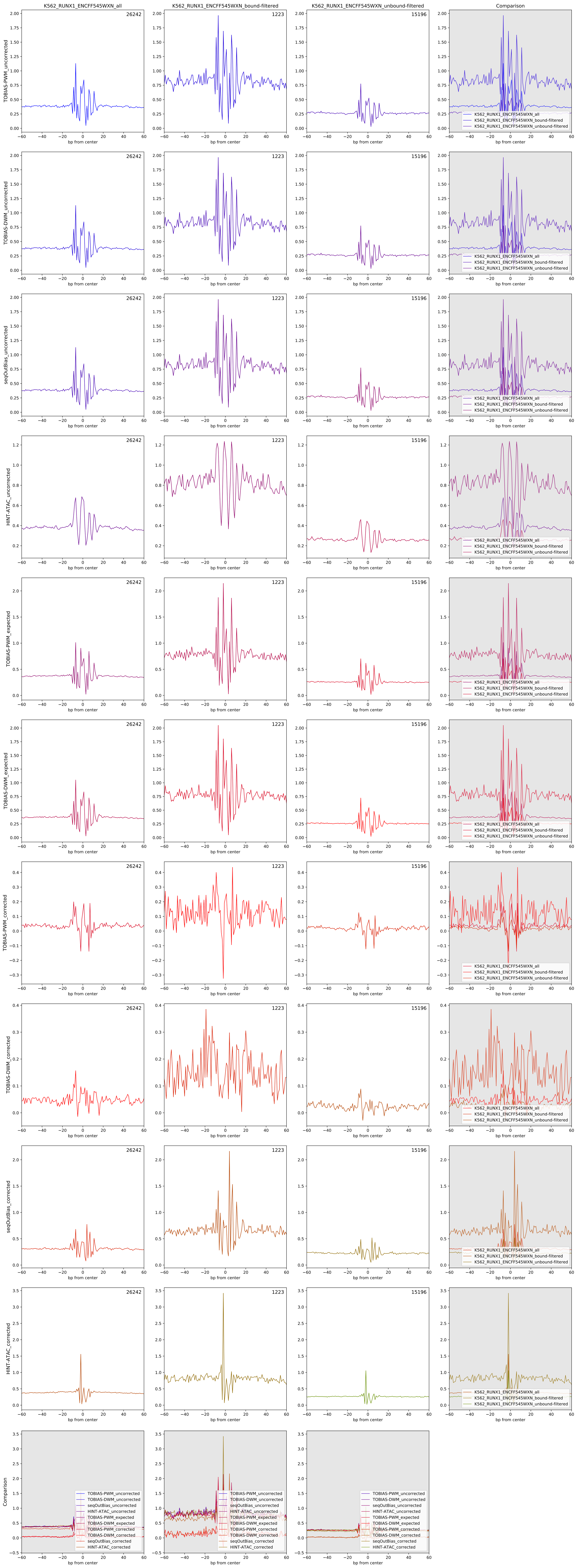

Aggregate footprints in K562 for TF SOX6 (ChIP-seq ENCFF431STY)

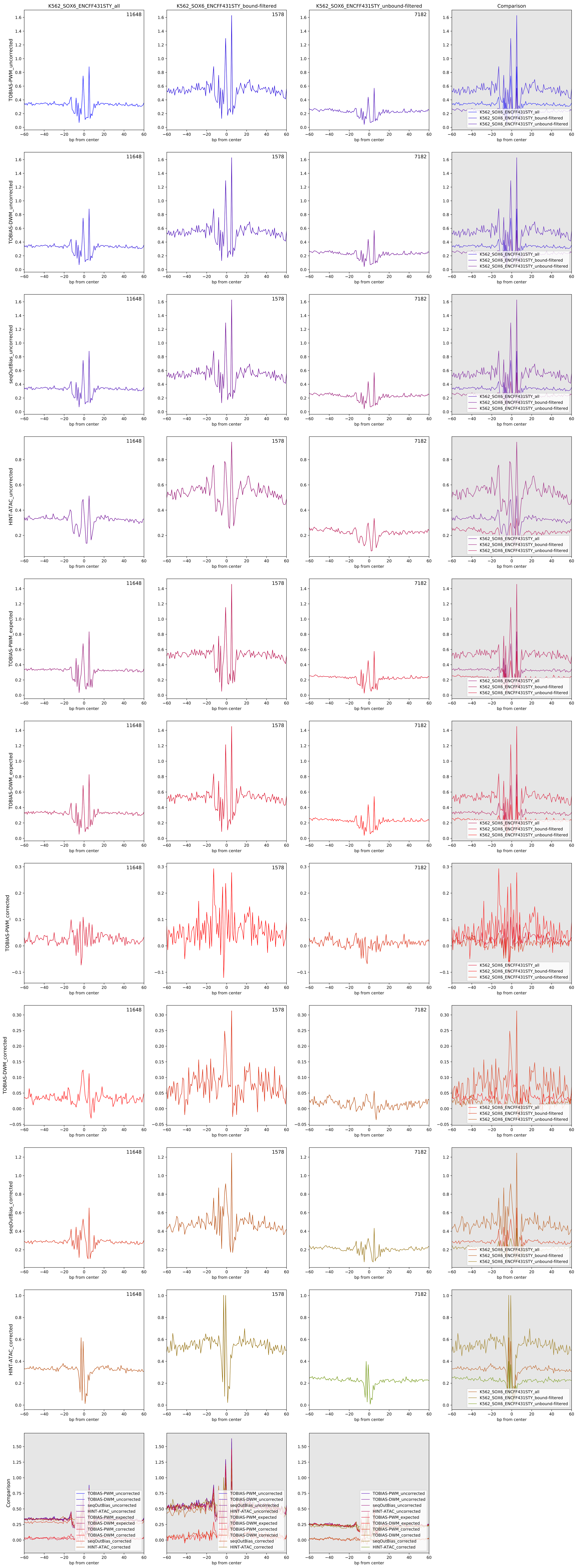

Aggregate footprints in K562 for TF SP1 (ChIP-seq ENCFF452LDK)

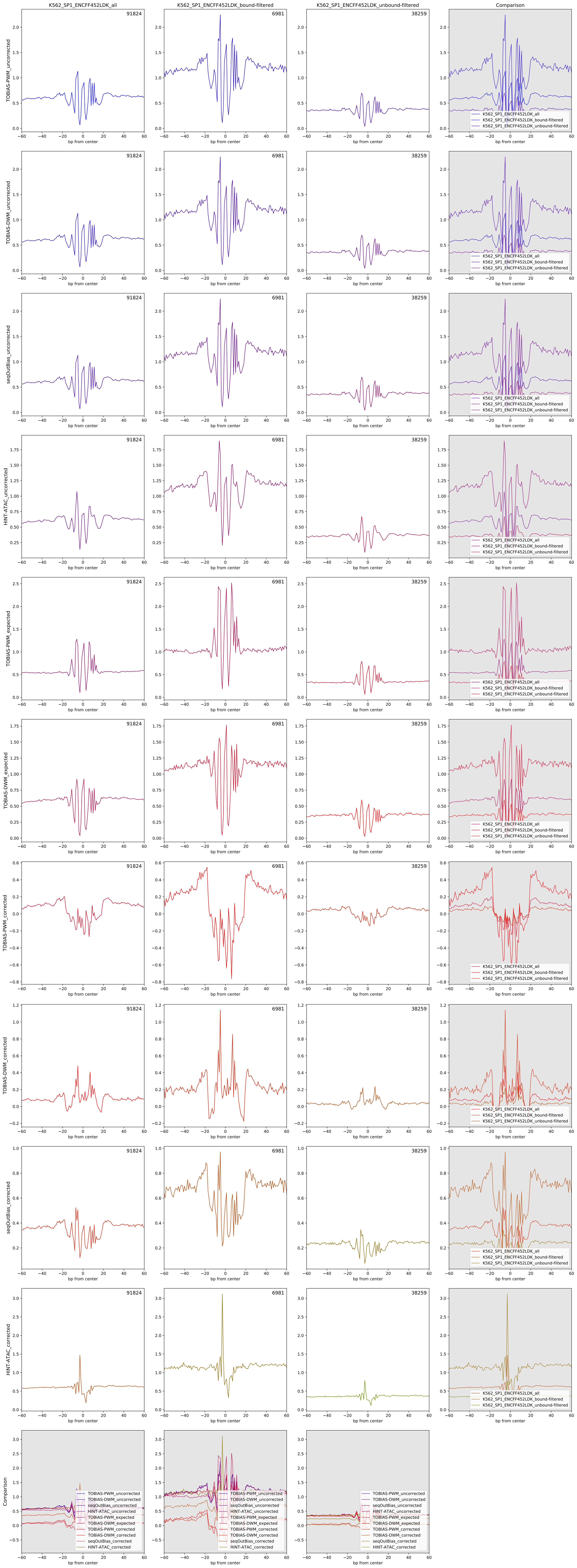

Aggregate footprints in K562 for TF SREBF1 (ChIP-seq ENCFF777MYW)

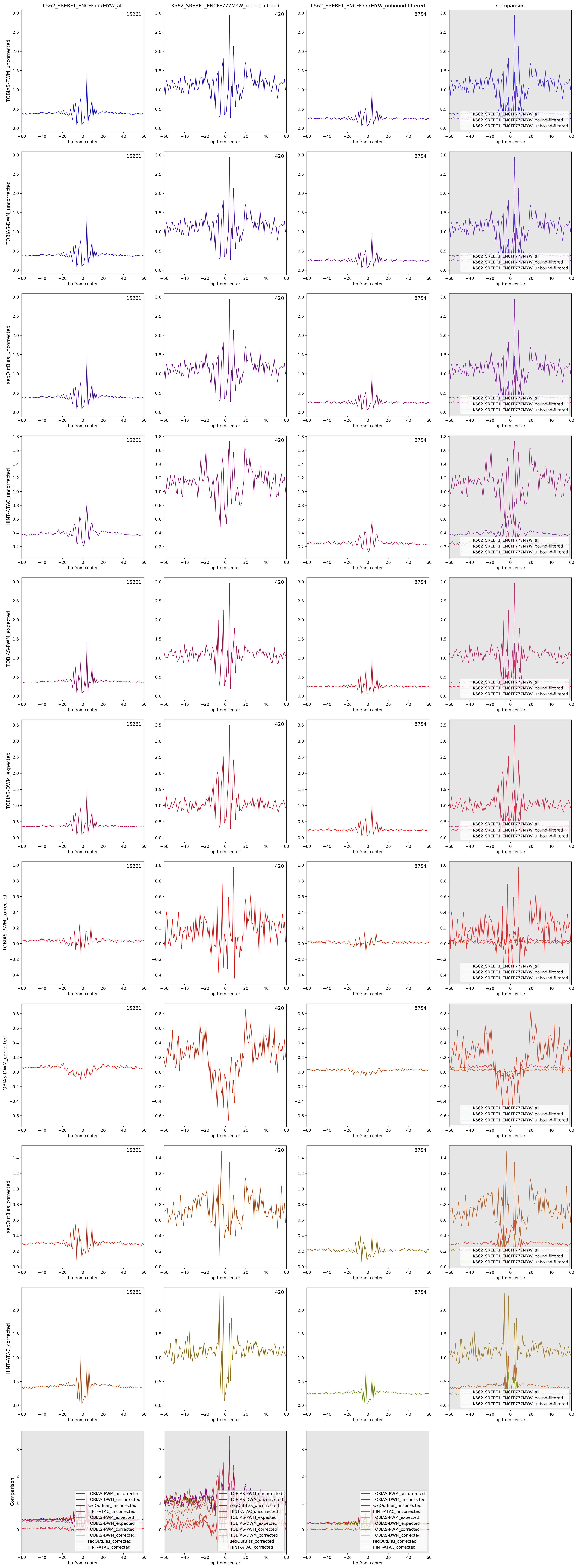

Aggregate footprints in K562 for TF STAT5A (ChIP-seq ENCFF517IXK)

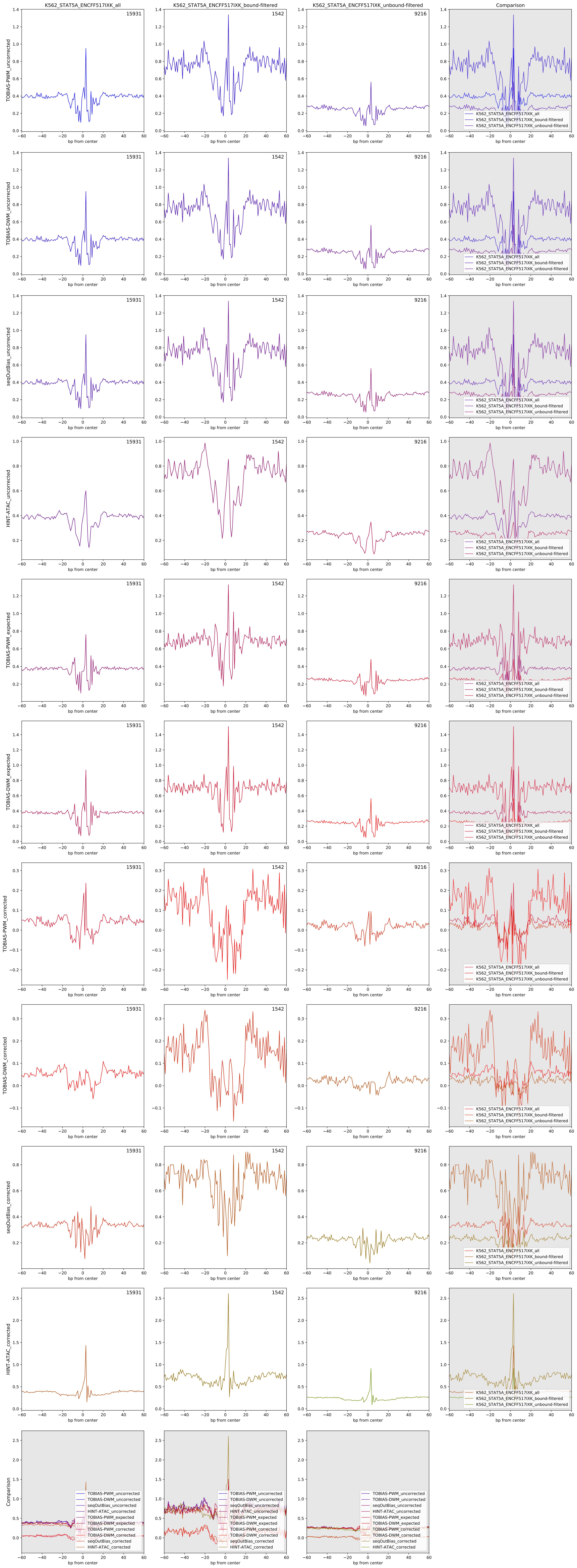

Aggregate footprints in K562 for TF TBP (ChIP-seq ENCFF370YGS)

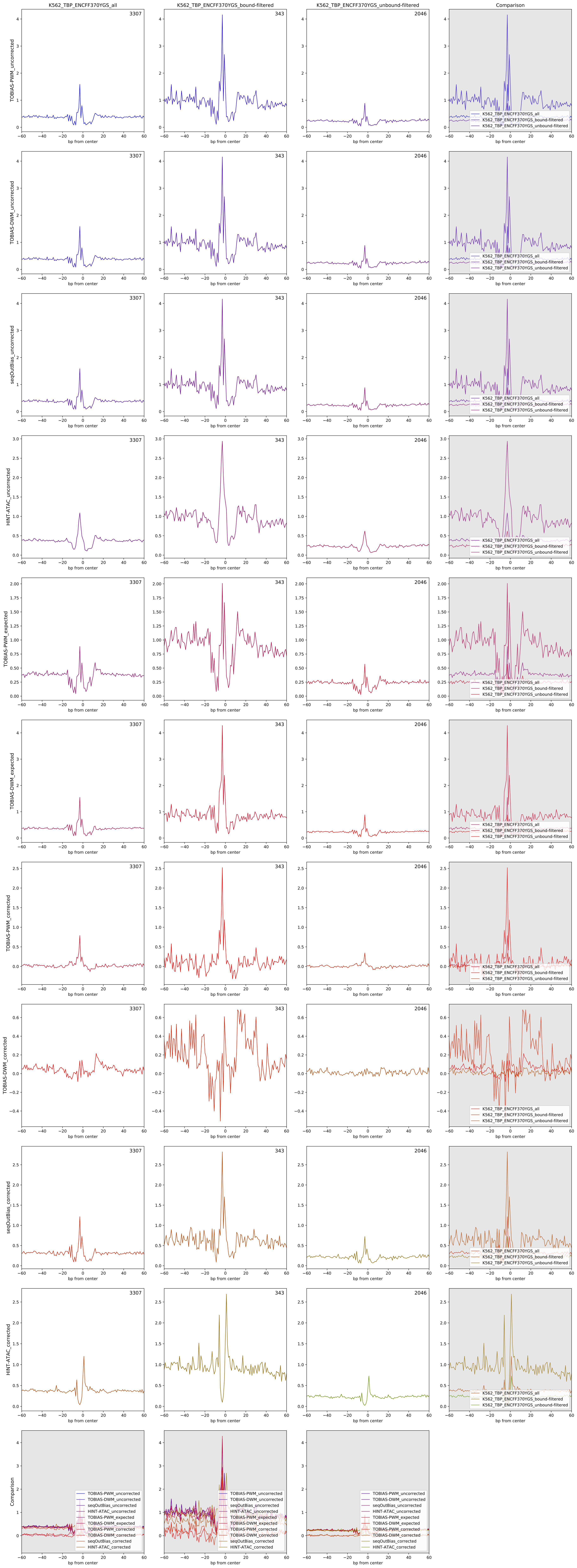

Aggregate footprints in K562 for TF TCF12 (ChIP-seq ENCFF912LXU)

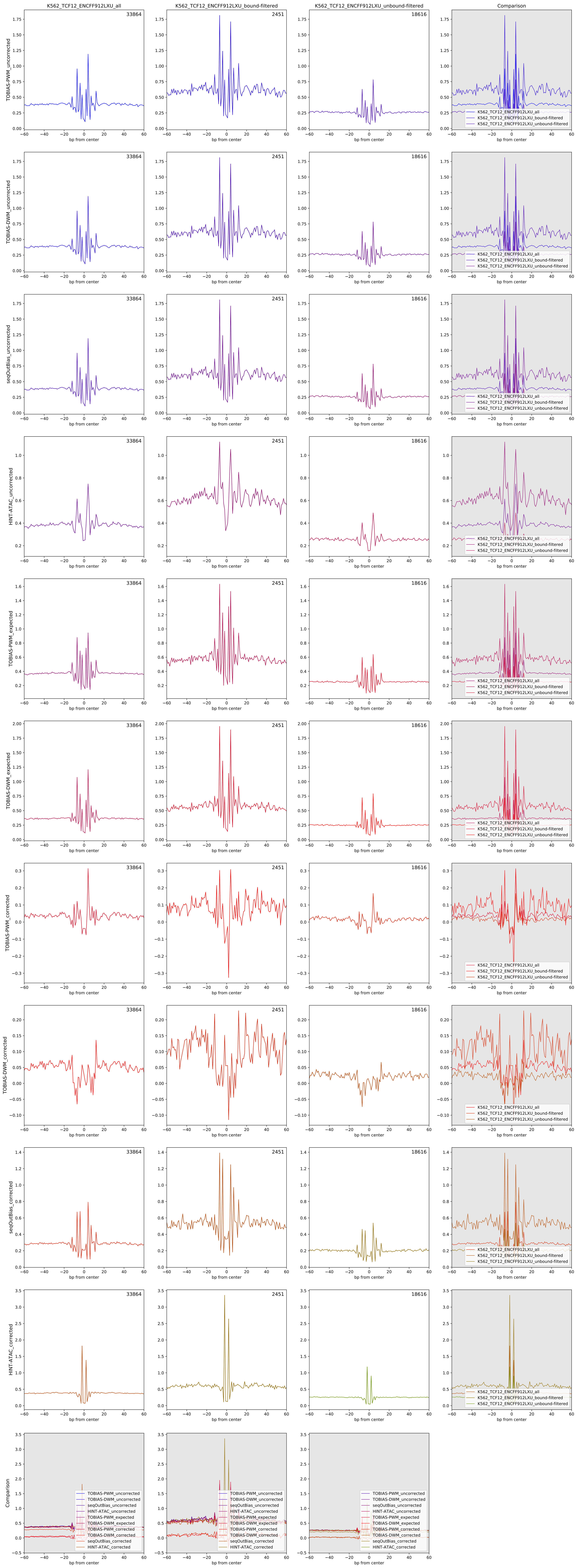

Aggregate footprints in K562 for TF TCF7 (ChIP-seq ENCFF512IAI)

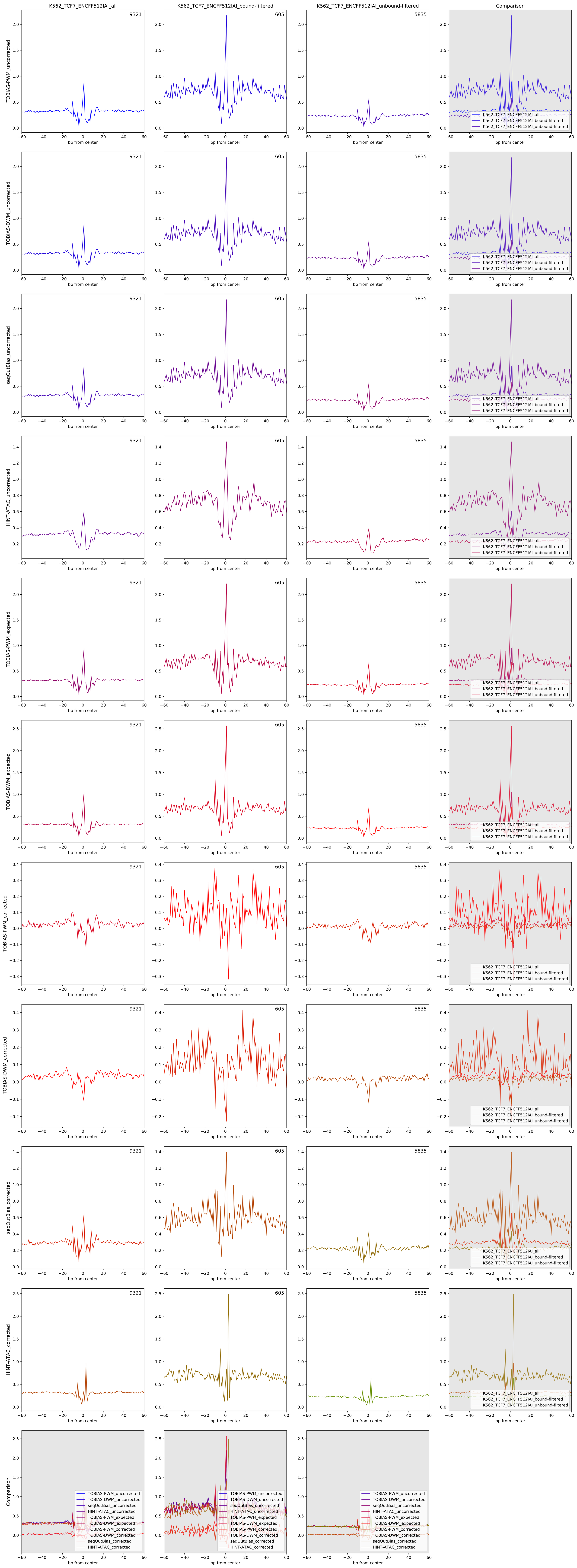

Aggregate footprints in K562 for TF TCF7L2 (ChIP-seq ENCFF556YFF)

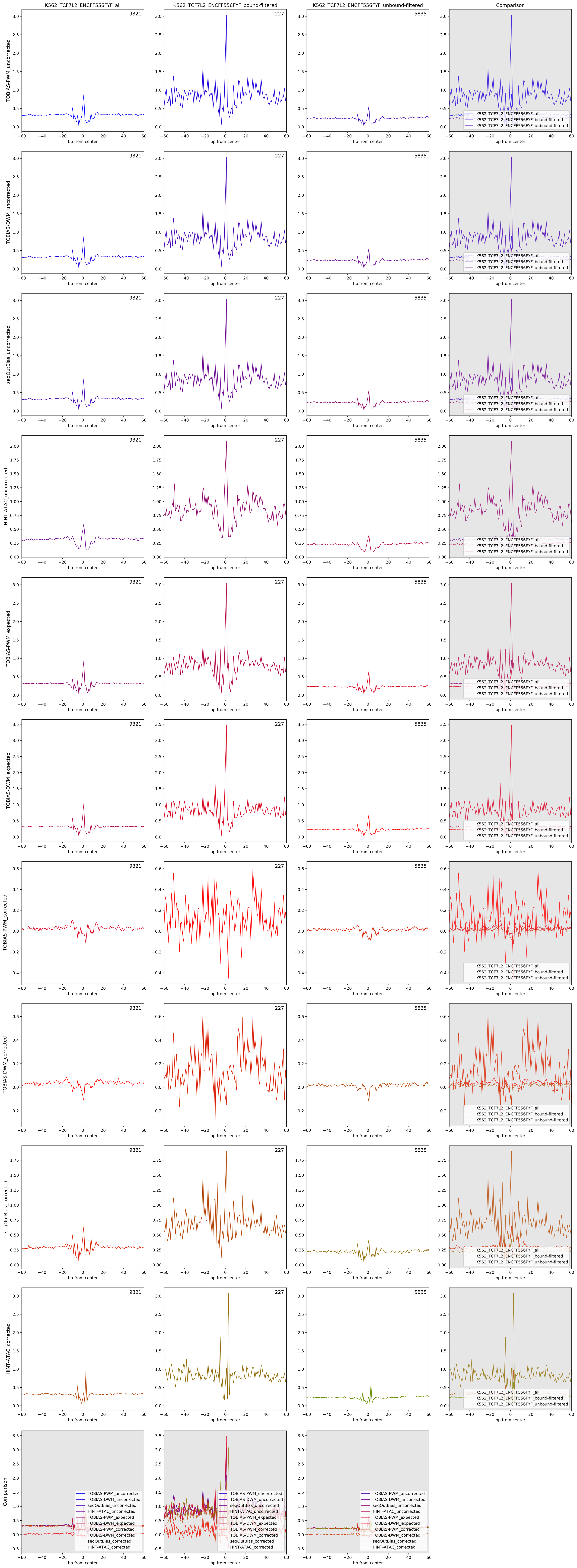

Aggregate footprints in K562 for TF TEAD2 (ChIP-seq ENCFF563LGJ)

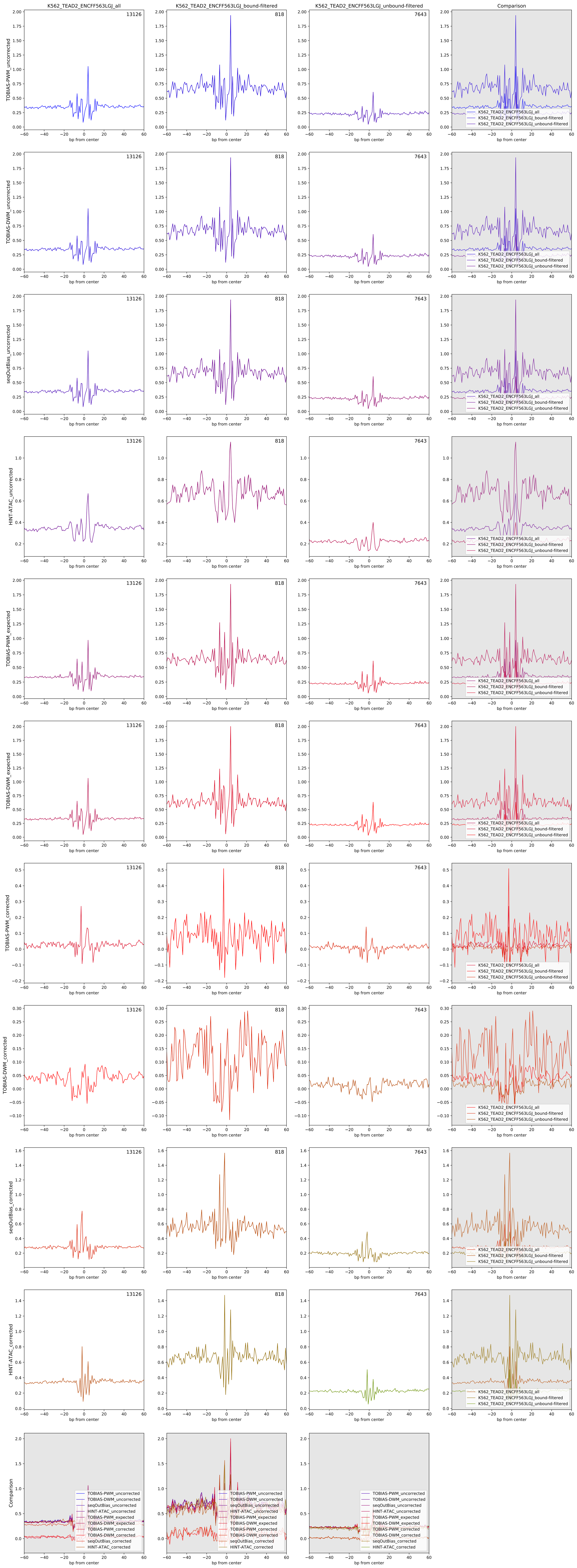

Aggregate footprints in K562 for TF TEAD4 (ChIP-seq ENCFF547MLB)

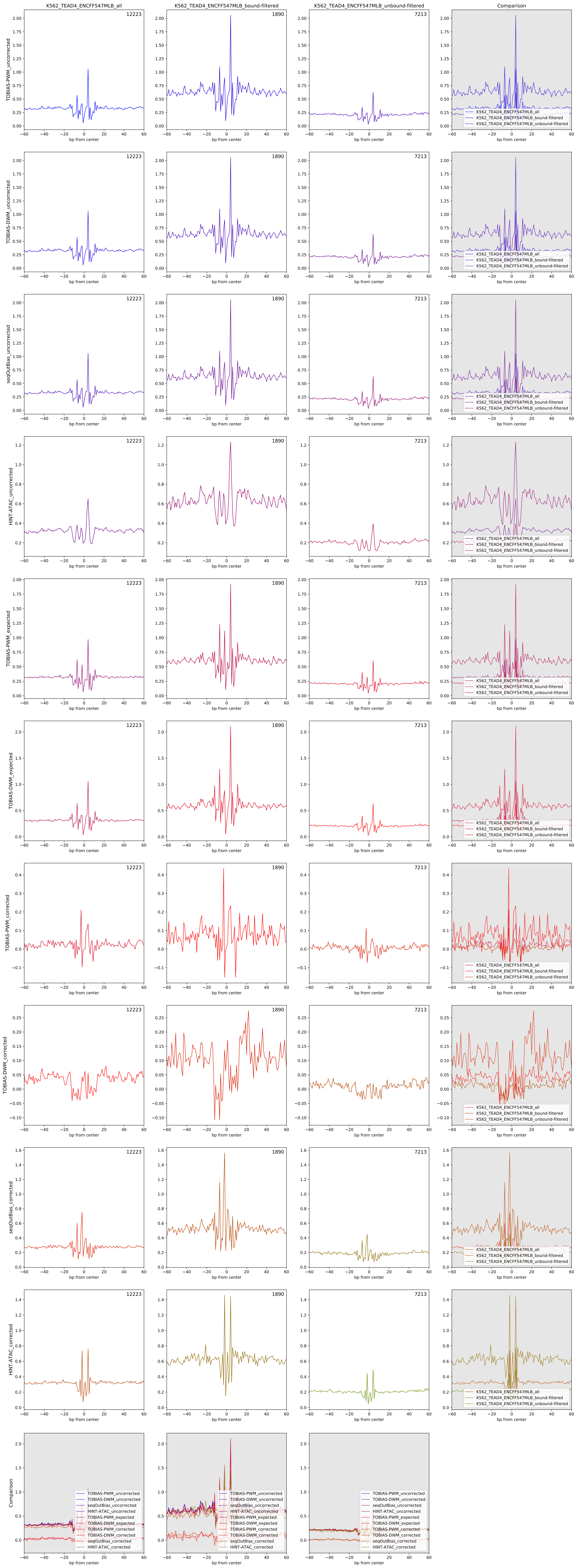

Aggregate footprints in K562 for TF TFDP1 (ChIP-seq ENCFF354QYM)

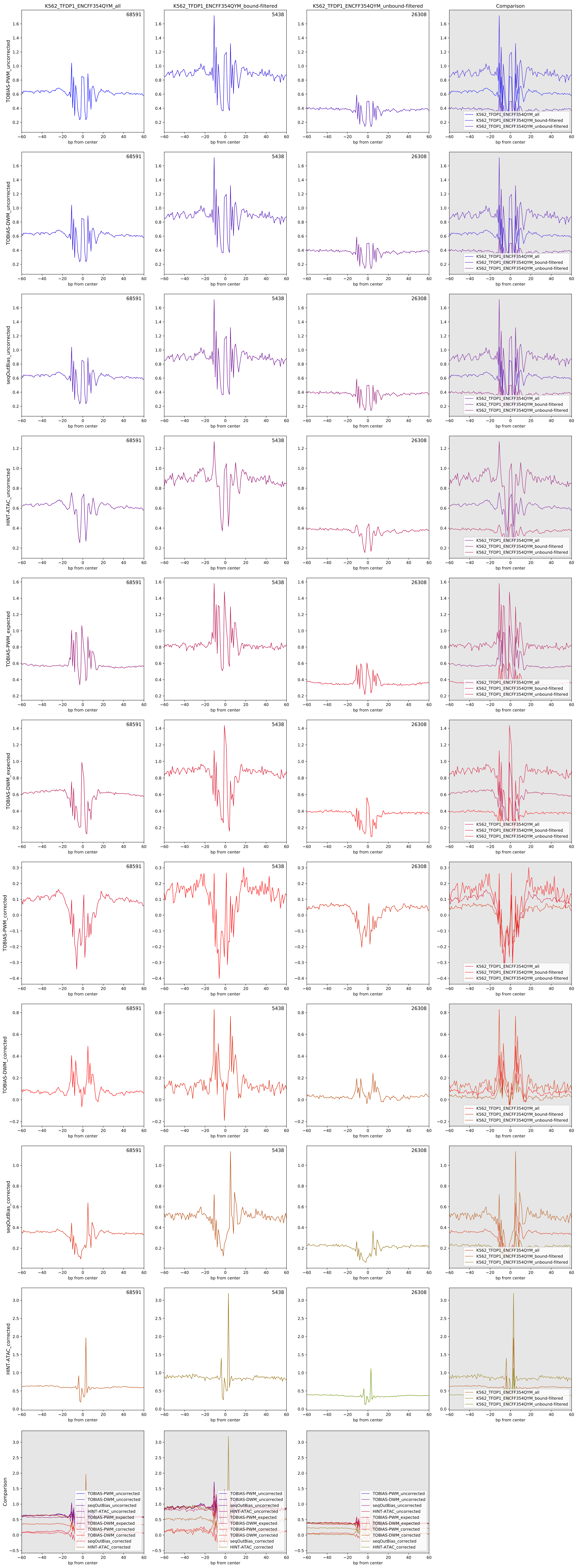

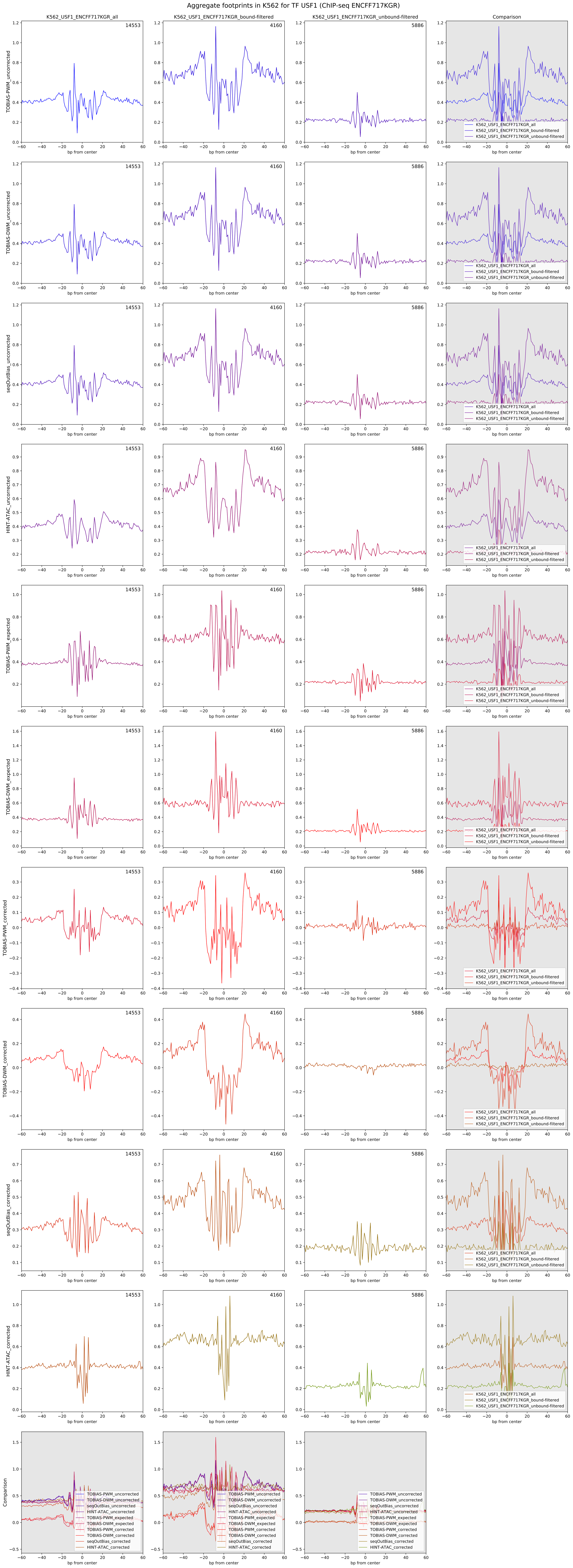
Aggregate footprints in K562 for TF USF1 (ChIP-seq ENCFF717KGR)
K562_USF1_ENCFF717KGR_all
K562_USF1_ENCFF717KGR_bound-filtered
K562_USF1_ENCFF717KGR_unbound-filtered
Comparison
TOBIAS-PWM_uncorrected
TOBIAS-DWM_uncorrected
seqOutBias_uncorrected
HINT-ATAC_uncorrected
TOBIAS-PWM_expected
TOBIAS-DWM_expected
TOBIAS-PWM_corrected
TOBIAS-DWM_corrected
seqOutBias_corrected
HINT-ATAC_corrected
14553
4160
5886
bp from center

Aggregate footprints in K562 for TF USF2 (ChIP-seq ENCFF208VCB)

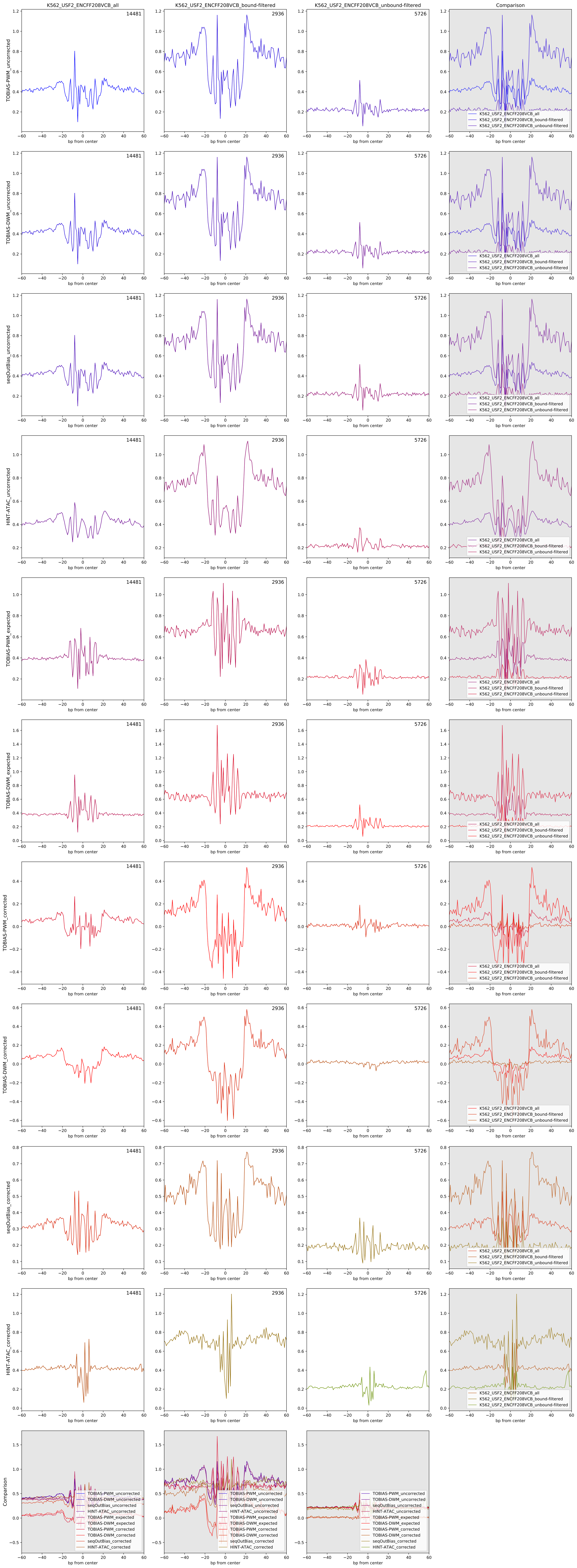

Aggregate footprints in K562 for TF YY1 (ChIP-seq ENCFF024TJO)

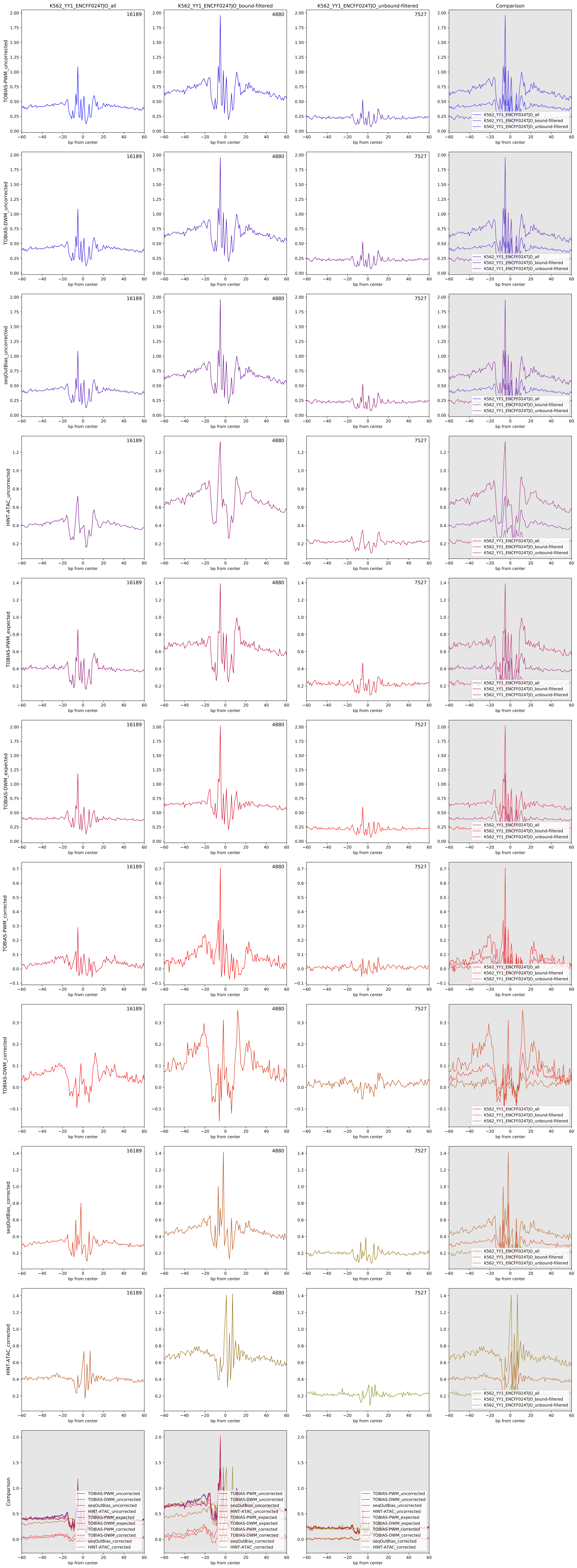

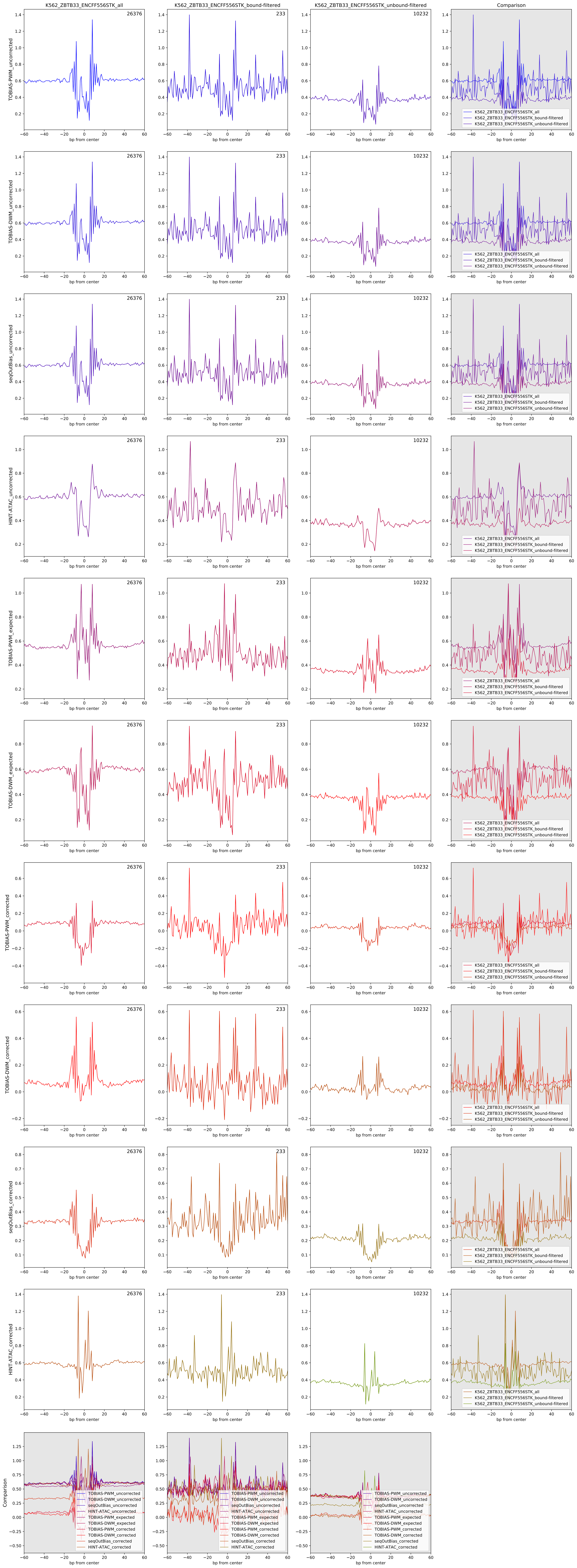

Aggregate footprints in K562 for TF ZBTB33 (ChIP-seq ENCFF556STK)
K562_ZBTB33_ENCFF556STK_all
K562_ZBTB33_ENCFF556STK_bound-filtered
K562_ZBTB33_ENCFF556STK_unbound-filtered
Comparison
TOBIAS-PWM_uncorrected
TOBIAS-DWM_uncorrected
seqOutBias_uncorrected
HINT-ATAC_uncorrected
TOBIAS-PWM_expected
TOBIAS-DWM_expected
TOBIAS-PWM_corrected
TOBIAS-DWM_corrected
seqOutBias_corrected
HINT-ATAC_corrected
26376
233
10232
bp from center

Aggregate footprints in K562 for TF ZNF143 (ChIP-seq ENCFF700GZI)

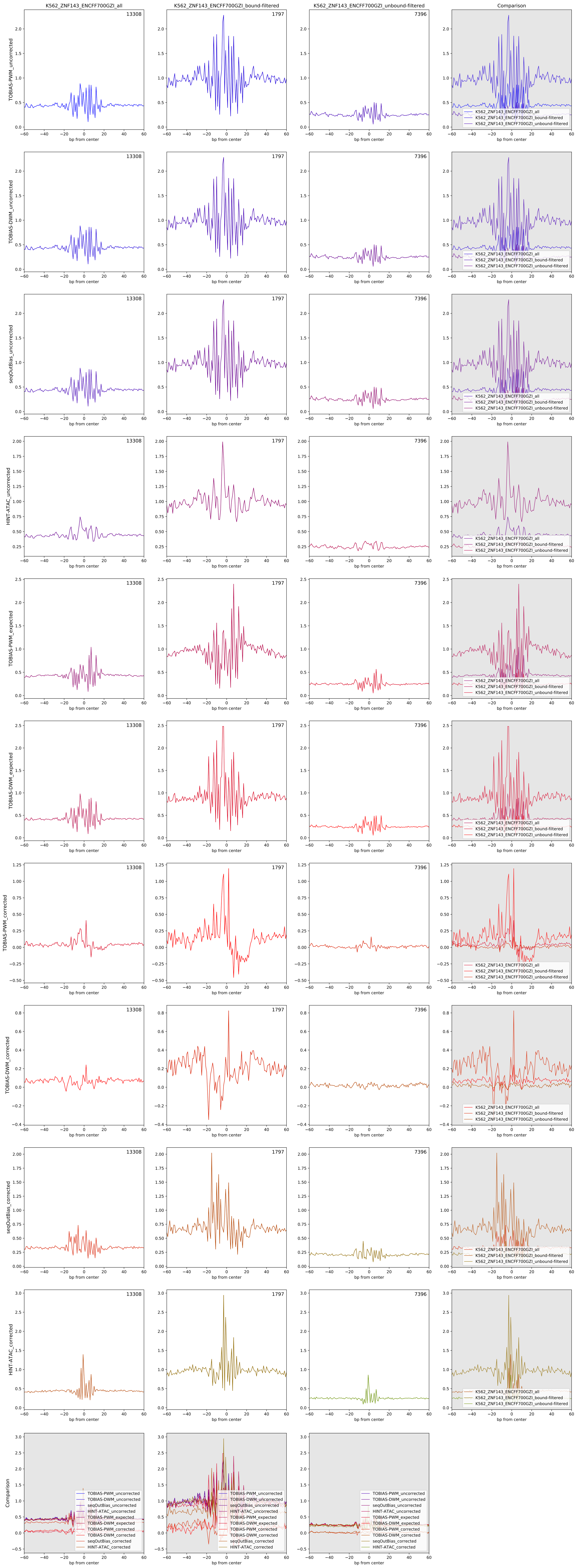

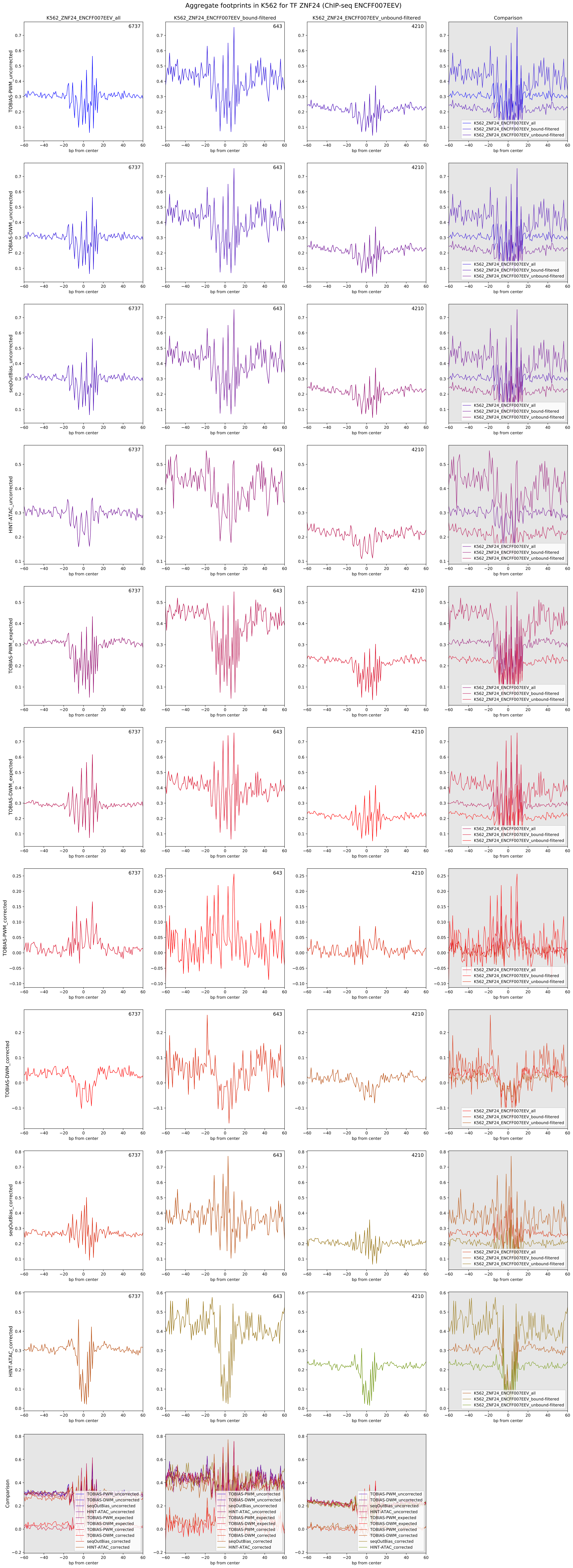

Aggregate footprints in K562 for TF ZNF24 (ChIP-seq ENCFF007EEV)
K562_ZNF24_ENCFF007EEV_all
K562_ZNF24_ENCFF007EEV_bound-filtered
K562_ZNF24_ENCFF007EEV_unbound-filtered
Comparison
TOBIAS-PWM_uncorrected
TOBIAS-DWM_uncorrected
seqOutBias_uncorrected
HINT-ATAC_uncorrected
TOBIAS-PWM_expected
TOBIAS-DWM_expected
TOBIAS-PWM_corrected
TOBIAS-DWM_corrected
seqOutBias_corrected
HINT-ATAC_corrected
6737
643
4210
bp from center

Aggregate footprints in K562 for TF ZNF263 (ChIP-seq ENCFF579ETM)

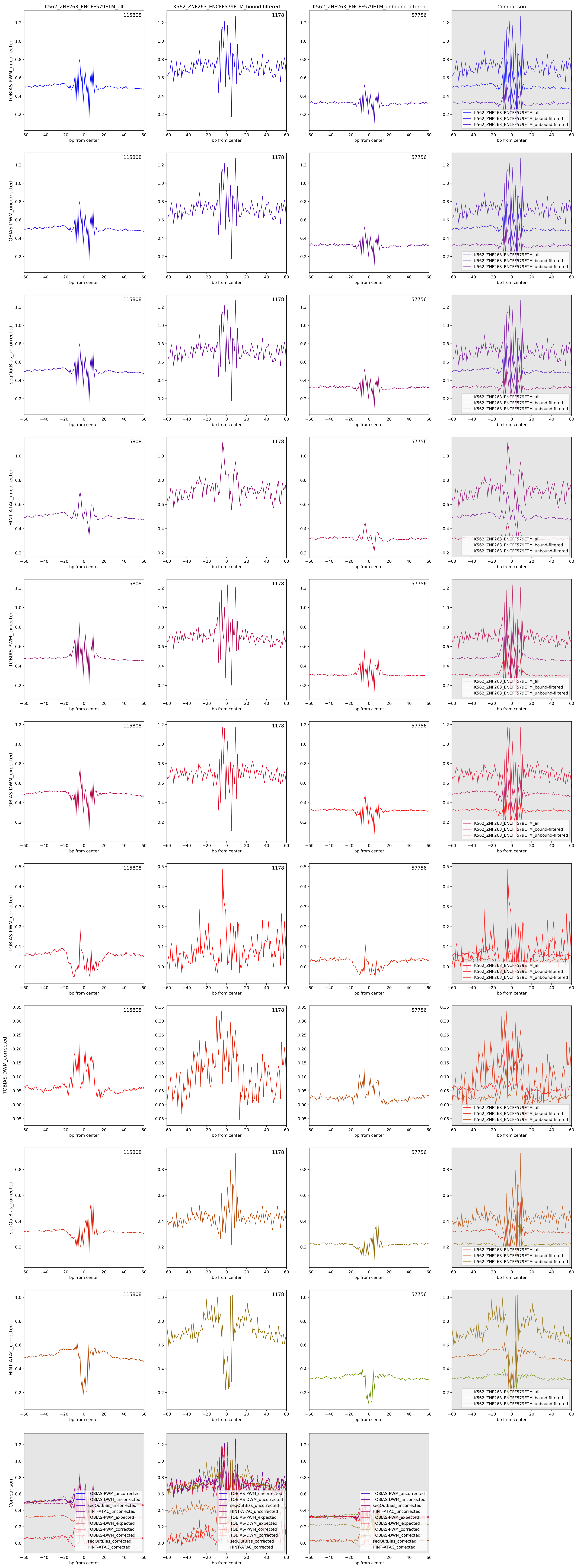

Aggregate footprints in K562 for TF ZNF384 (ChIP-seq ENCFF106YXG)

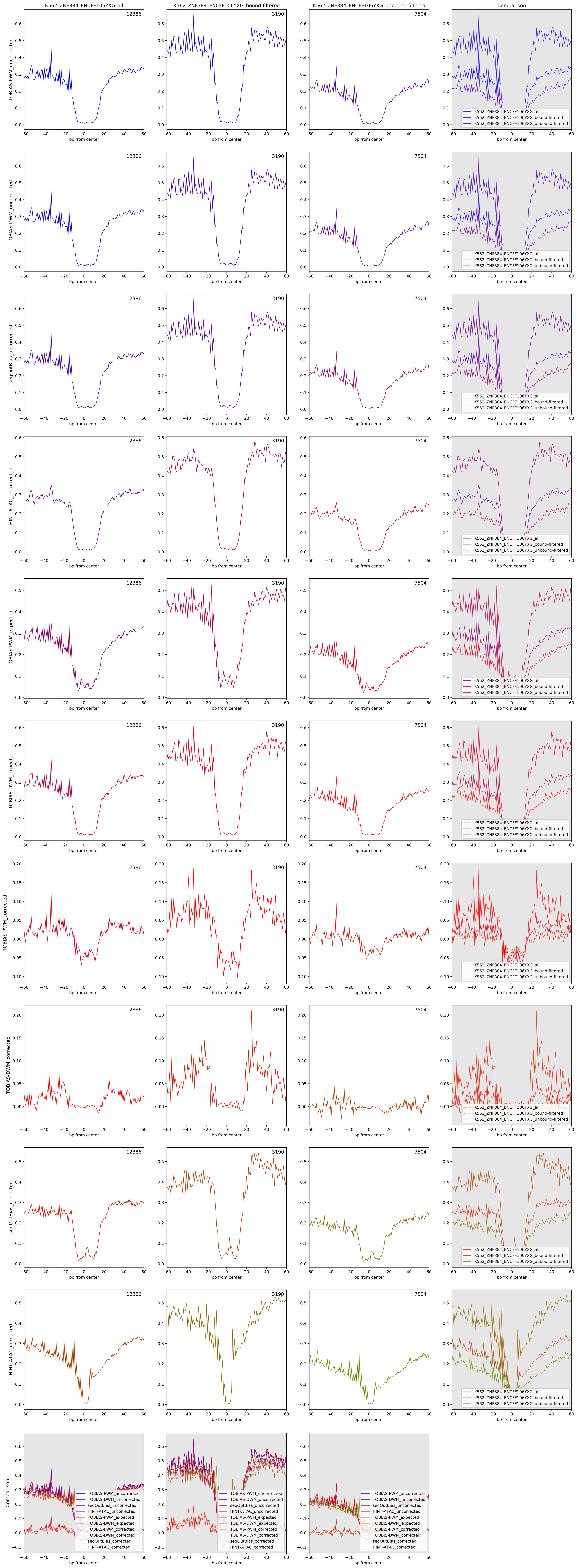

Aggregate footprints in K562 for TF ZNF740 (ChIP-seq ENCFF301FKQ)

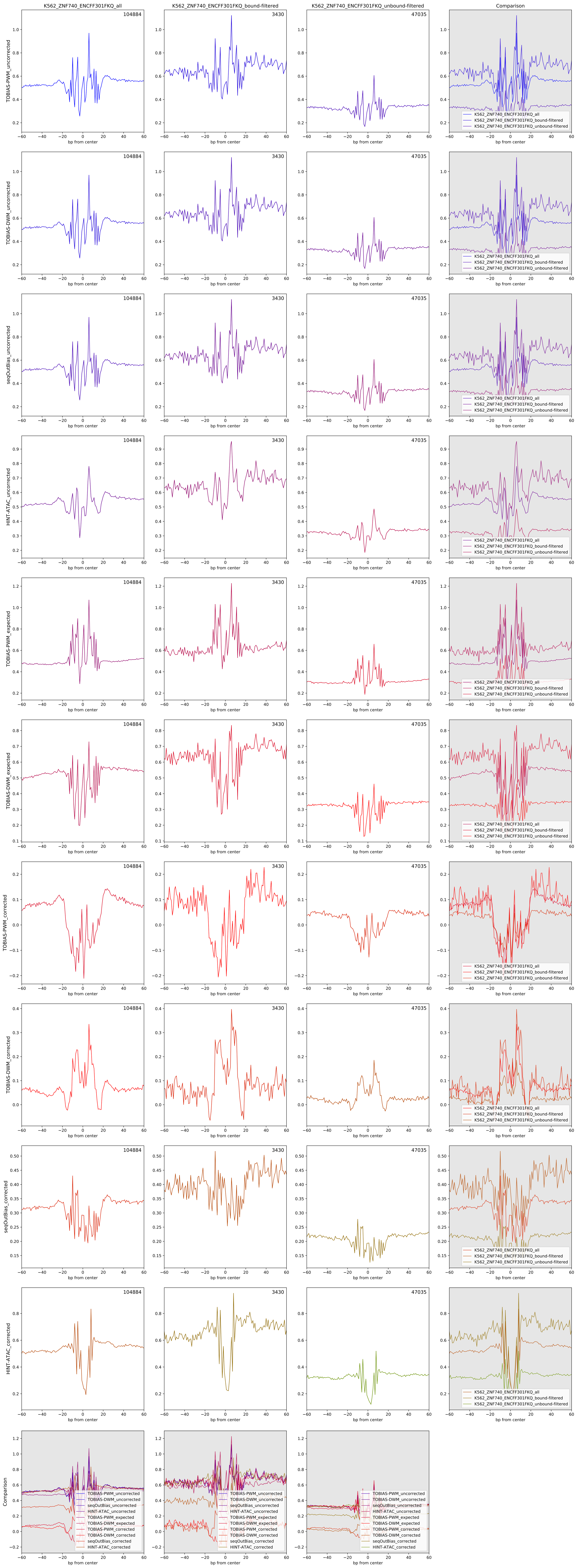

Supplement: Supplementary file 4 — Supplementary Data 1 [file 41467_2020_18035_MOESM4_ESM.pdf]
